# The role of continental shelf bathymetry in shaping marine range shifts in the face of climate change

**DOI:** 10.1111/gcb.16276

**Published:** 2022-06-13

**Authors:** Zoë J. Kitchel, Hailey M. Conrad, Rebecca L. Selden, Malin L. Pinsky

**Affiliations:** ^1^ Ecology and Evolution Graduate Program Rutgers University New Brunswick New Jersey USA; ^2^ Department of Fish and Wildlife Conservation Blacksburg Virginia USA; ^3^ Biological Sciences Wellesley College Wellesley Massachusetts USA; ^4^ Department of Ecology, Evolution, and Natural Resources Rutgers University New Brunswick New Jersey USA

**Keywords:** continental shelf, depth, habitat gain, habitat loss, latitude, species area relationship, species distributions

## Abstract

As a consequence of anthropogenic climate change, marine species on continental shelves around the world are rapidly shifting deeper and poleward. However, whether these shifts deeper and poleward will allow species to access more, less, or equivalent amounts of continental shelf area and associated critical habitats remains unclear. By examining the proportion of seabed area at a range of depths for each large marine ecosystem (LME), we found that shelf area declined monotonically for 19% of LMEs examined. However, the majority exhibited a greater proportion of shelf area in mid‐depths or across several depth ranges. By comparing continental shelf area across 2° latitudinal bands, we found that all coastlines exhibit multiple instances of shelf area expansion and contraction, which have the potential to promote or restrict poleward movement of marine species. Along most coastlines, overall shelf habitat increases or exhibits no significant change moving towards the poles. The exception is the Southern West Pacific, which experiences an overall loss of area with increasing latitude. Changes in continental shelf area availability across latitudes and depths are likely to affect the number of species local ecosystems can support. These geometric analyses help identify regions of conservation priority and ecological communities most likely to face attrition or expansion due to variations in available area

## Graphical abstract

Continental shelves provide critical habitat for a diverse array of marine species. There is no general rule for how availability of these essential habitats vary across spaceinstead, there is tremendous variability in continental shelf area across latitudes and depths that species will encounter while seeking more suitable environmental conditions. We highlight regions where we may expect increased area to facilitate species range shifts and where we expect decreased area to restrict species range shifts.

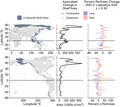

## 1 INTRODUCTION

Many species in terrestrial and aquatic systems are shifting where they live in response to climate change (Lenoir & Svenning, 2015). Marine species are particularly sensitive to temperature changes associated with climate change, in part because they have evolved in the relatively stable thermal conditions characteristic of the ocean (Pinsky et al., 2019). This high sensitivity, coupled with higher dispersal potential and limited biogeographical barriers have led marine species to track isotherms poleward six times faster than their terrestrial counterparts (Lenoir et al., 2020). In addition, there is evidence that marine species are moving deeper to maintain their thermal niche (Dulvy et al., 2008; Perry et al., 2005; Pinsky et al., 2013; Poloczanska et al., 2016).

As species undergo range shifts, they also experience changes in the availability and quality of habitat (Platts et al., 2019). Sufficient habitat area is critical for population viability, and subsequently, for successful range shifts (Opdam & Wascher, 2004). The number of individuals a habitat can support often scales with the size of the habitat (Alzate et al., 2019; Halpern et al., 2005). Larger habitat areas provide more opportunities for establishment and growth in the case of sessile individuals, and more opportunities for foraging for more mobile individuals (Bender et al., 1998; Griffen & Drake, 2008; MacArthur & Wilson, 1967). Many species rely on metapopulation structure across space to maintain populations large enough to avoid inbreeding depression and buffer against the risk of extinction due to demographic stochasticity and disturbances (Hanski et al., 1996; Kuparinen et al., 2014). Larger habitat areas also tend to support higher overall species richness because of increased heterogeneity and reduced likelihood of extinction (Cornell & Karlson, 2000; MacArthur & Wilson, 1967). Although the types and quality of habitats associated with continental shelves vary, shelf width can help predict the risk of extirpation for some marine species (Yan et al., 2021).

Continental shelves support productive, complex, and economically and culturally important marine ecosystems (Amoroso et al., 2018; Bell, 2009; Buhl‐Mortensen et al., 2012; Gomes et al., 2018; Smith & Brown, 2002). These essential habitats often exhibit high nutrient availability due to freshwater inputs and upwelling (specifically in eastern boundary ecosystems) (Chen et al., 2000; García‐Reyes et al., 2015). The relatively shallow waters (typically less than 200 m) permit light to penetrate the water column through to the substrate, promoting primary production in the form of plant and algal growth (Duarte, 1991; Kahng et al., 2019). On the continental shelf, depth and seafloor area are key components of suitable habitat. Unique biogenic and geologic structures that provide habitat and refuge supporting diverse and productive ecosystems are often limited to the continental shelf (Buhl‐Mortensen et al., 2012; Malatesta & Auster, 1999; Nagelkerken et al., 2000; Townsend et al., 2004). Many marine species are restricted to living on the continental shelf due to metabolic tolerances, and their reliance on primary production occurring within the photic zone (Brown & Thatje, 2015; Mestre et al., 2013; Smith & Brown, 2002). It is unlikely that marine species will successfully establish off of the continental shelf even as species shift into deeper waters (Dulvy et al., 2008). In support of this limitation on range shifts, studies have revealed distinct shelf, slope, and abyssal plain species assemblages, and even distinctive clustering within these larger ocean zones (Brandt et al., 2007; Fujita et al., 1995; Pearcy et al., 1982; Rocha et al., 2018).

Marine species face heterogeneity in shelf area as they move poleward and deeper to track temperature isotherms. The width of the continental shelf ranges from 778 km in the Weddell Sea of Antarctica to 11 km within the Mediterranean and Black Seas (Harris et al., 2014). The shelves exhibit high variability in structure across latitudes and depths, often bisected by deep canyons and channels (Heezen et al., 1964; Lastras et al., 2011). Additionally, shelf characteristics vary by plate activity within each ocean basin. Passive shelves including those of the Arctic Ocean and the Northern and Southern Atlantic Ocean are on average nearly three times wider than active shelves largely concentrated in the Pacific (Harris et al., 2014). The stark contrast between the high number of extinctions in the late Ordovician when compared to the Cenozoic despite similar changes in climate was partially driven by a lack of contiguous continental shelf area which can facilitate range shifts to more suitable climates (Finnegan et al., 2016; Sheehan, 2001). How the availability of shelf area will change as species shift due to modern climate change, however, has yet to be examined.

Similar variations in habitat availability are faced by terrestrial species as they shift in latitude and elevation. In some cases, corridors such as protected areas can facilitate poleward movement, while in other cases, obstacles such as rivers or human altered landscapes can restrict movement (Beier, 2012; Jha & Kremen, 2013; Thomas, 2010). Shifts of marine species into deeper waters have been mirrored by terrestrial species shifting to higher elevations (Freeman, Lee‐Yaw, et al., 2018; Freeman, Scholer, et al., 2018; Vitasse et al., 2021). For both marine and terrestrial communities, available area is not synonymous with high quality habitat. Carrying capacity can vary with substrate, food web structure, precipitation patterns, water characteristics, and human impacts (Amoroso et al., 2018; Myers et al., 2001; Nagelkerken et al., 2000). Still, larger areas are more likely to provide useful habitats to species (Grantham et al., 2020; Mortelliti et al., 2010; Stuhldreher & Fartmann, 2014). Despite the prevailing assumption that montane surface area decreases with elevation and therefore that species will lose habitat as they track temperature upslope, topographic analyses have revealed that the relationship between habitat area and elevation differs by mountain range. For the majority of mountain ranges, surface area does not decrease monotonically as species move upslope, and in a few select ranges, species will find the largest areas of suitable habitat at the highest elevations (Elsen & Tingley, 2015). No comparable analysis has yet been conducted on regional continental shelf area as a function of ocean depth or latitude. Globally, habitat area decreases from a maximum at sea level to a minimum near 750 m depth, at which point habitat area increases towards a second peak in area at the abyssal plain (Eakins & Sharman, 2012). However, patterns in shelf area across depth and latitude at the regional scale—most relevant to marine organisms undergoing range shifts—have not yet been described.

The goal of this study is to assess the changes in continental shelf area that species will face as they make range shifts into deeper depths and higher latitudes. We evaluate the regional changes in shelf area availability across depths and latitudes in the Pacific, Atlantic, and Indian Oceans. These bathymetric analyses highlight key areas where populations and communities may be enhanced or constrained by shelf area.

## 2 METHODS

### 2.1 Shelf area by depth within large marine ecosystems

First, we assessed how continental shelf availability varies across depth at the scale of large marine ecosystems (LMEs). Because of their distinct bathymetry, hydrology, productivity, and trophic interactions, LMEs are a useful regional unit to assess the extent and impact of depth shifts (Sherman & Alexander, 1986; Sherman & Duda, 1999). We obtained LME delineations from the ScienceBase Catalog of the United States Geological Survey (Sherman et al., 2017). We used the continental shelf definition from the Blue Habitats web portal (Harris et al., 2014), which included all submerged area adjacent to land and islands from the low water mark to the point where the slope increased markedly beyond a slope of 1:2000 and towards ocean depths (IHO, 2008). We note that LMEs contain areas deeper than the continental shelf, which is on average ~200 m. Conversely, not all continental shelf regions are included by LMEs. Therefore, the LME shapefiles were trimmed to focus on continental shelf area within LMEs for the depth analyses. Continental shelf areas excluded from LME designation were left out of the depth analyses.

We used the Bedrock ETOPO1 one arc‐minute global digital elevation model dataset to extract bathymetry for the continental shelf regions within LMEs (Amante & Eakins, 2009). The Blue Habitat shelf delineation primarily includes shallow shelf regions, but we excluded any areas with depths below 2000 m to eliminate any misclassifications (0.01% of grid cells). We characterized the distribution of shelf area at depth by plotting hypsometric curves for each LME. Because of the deep bathymetry of the Central Arctic (LME 64) and the unique isolation of the Antarctic (LME 61), both polar LMEs were excluded from the analyses. Area in km^2^ at each 1 m depth bin was calculated using the area function of the raster package implemented in R (Hijmans, 2021; R Core Team, 2021). We verified that calculating area from projected polygons did not meaningfully change results (Figures S1–S3).

**FIGURE 1:**
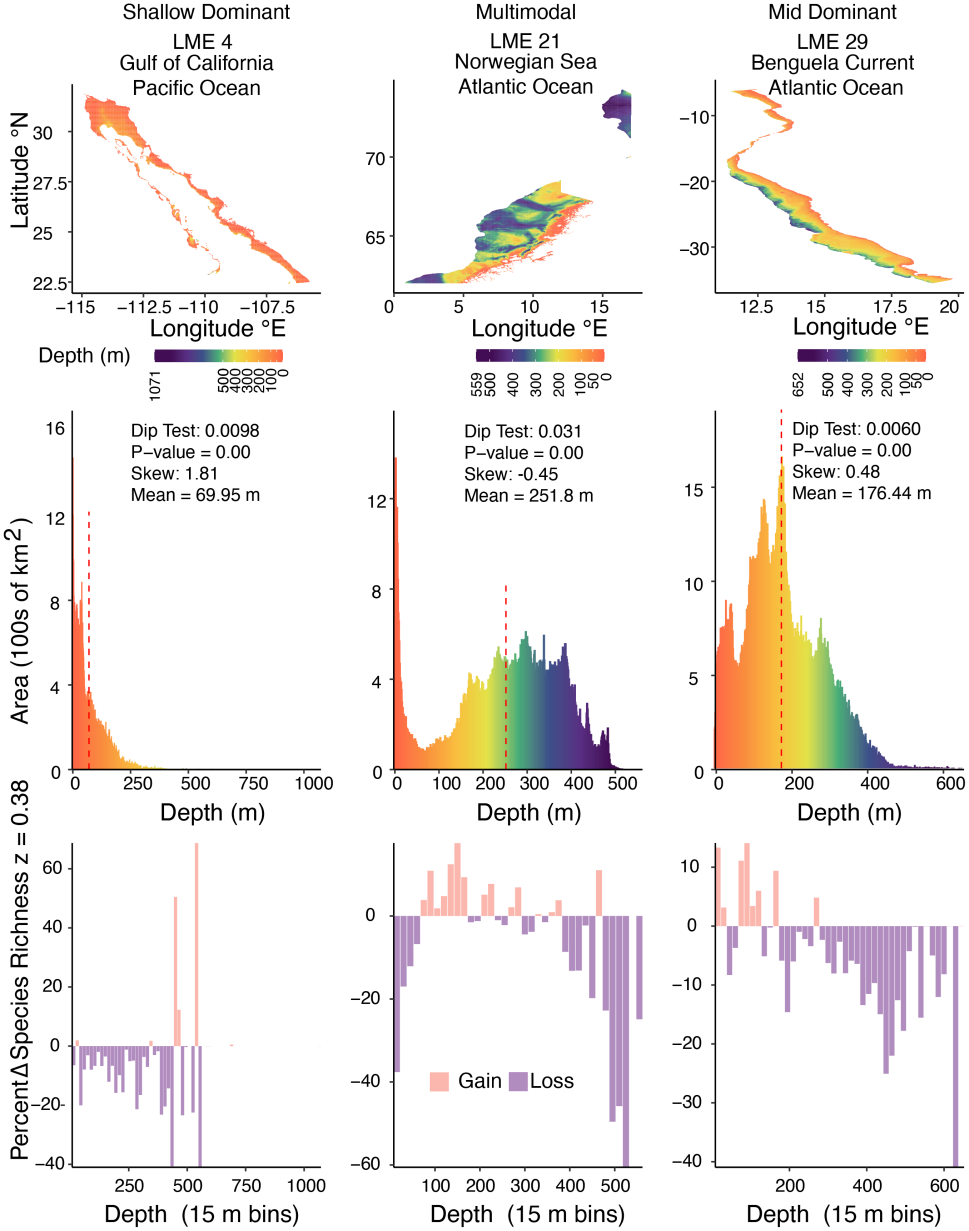
Example classifications for how shelf area availability changes with depth within a LME. Depth distribution map (above), hypsometric curve (middle), and SAR richness predictions (below). Deep Dominant and Uniform are not shown because no LMEs were assigned these types. Color represents depth (above, middle), and the vertical dashed red bar on the hypsometric curves (middle) indicates mean depth for the LME. Predicted percent change in species richness was calculated for a 15 m depth shift using an average SAR with an exponent of *z* = 0.38 (bottom). See Figures S5, S6, and S10b for depth maps, hypsometric curves and change in species richness for all 64 LMEs, and see Figure S10a,c for SAR calculations using the minimum and maximum exponent values for marine fish. LME, large marine ecosystem; SAR, species area relationships.

We classified the depth distribution of LMEs into five categories based on the skew, modality, and uniformity of the hypsometric curve: Shallow‐Dominant, Mid‐Dominant, Deep‐Dominant, Uniform, and Multimodal (Figure 1). Curves for which we were unable to reject the null hypothesis of a uniform distribution using the Kolmogorov–Smirnov test (*p*‐value >.05) were classified as uniform (Kolmogorov, 1933; Smirnov, 1939). When uniformity was rejected, Hartigan's dip test was implemented in R to assess modality (Hartigan & Hartigan, 1985; Maechler, 2021). Curves with a dip test statistic greater than 0.01 and *p*‐value <.05 were categorized as Multimodal. All curves that did not meet the criteria for Multimodal or Uniform were categorized based on skew. Curves with skew values less than −1 (high left skew) were assigned to Deep‐Dominant, between −1 and 1 to Mid‐Dominant, and greater than 1 (high right skew) to Shallow‐Dominant. We also assessed the sensitivity of classifications to a less conservative skew using threshold values of −0.5 and 0.5 for Deep‐Dominant and Shallow‐Dominant designations.

### 2.2 Shelf area across latitude

To assess the changes in seafloor area experienced by species shifting poleward, we joined together the continental shelf components of LMEs along each continental boundary. We analyzed changes in shelf area availability from low to high latitudes for continental shelves of the West Pacific, East Pacific, West Atlantic, East Atlantic, West Indian, and East Indian oceans. For coastlines not contained within LMEs (i.e. southern coast of Sumatra and Papua New Guinea), we supplemented the LME‐restricted ETOPO1 bathymetric rasters with ETOPO1 bathymetric rasters trimmed to continental shelf areas within FAO major marine fishing areas (FAO, 2019; Area 57 assigned to East Indian and Area 71 assigned to West Pacific). Large islands were kept in the analyses if they were a part of a mainland LME (e.g. Madagascar), but excluded if they were an individual LME (e.g. New Zealand). For reasons described in the previous section, the Central Arctic and Antarctic LMEs were excluded from these analyses. Again, we used the Blue Habitat shelf delineation to restrict analyses to the continental shelf. We calculated area in km^2^ of the continental shelf for 2° latitudinal bins using the area function of the raster package implemented in R (Hijmans, 2021; R Core Team, 2021). We again verified that calculating area from projected polygons did not meaningfully change results (Figures S1–S3). Additionally, we calculated the percent change in seafloor area from each bin to the next poleward bin. The 2° latitudinal bin size is representative of the average range shift for marine species over a 40 year period (Lenoir et al., 2020). The International Union for the Conservation of Nature classifies species that have experienced a 50% loss in habitat or population as Vulnerable to Extinction (IUCN Standards and Petitions Committee, 2019), therefore we identified locations where there was either doubling (expansion) or halving (contraction) in seafloor area from one 2° latitudinal bin to the next poleward bin. Finally, to assess the overall pattern in shelf area availability over latitude, we regressed the continental shelf areas of each bin against the bin mid‐latitudes and extracted the slope and the *p*‐value of the resulting linear model.

### 2.3 Estimating percent changes in species richness using species area relationships

To calculate the potential percent change in species richness associated with a given change in continental shelf area, we used the mean of a range of exponents for species area relationships (SAR) developed for fishes across ecosystems based on the power‐law *S = C* × *A* ^*z*^ where *A* is area and the mean exponent *z* = 0.38 (Levin et al., 2009)*. C* is a constant based on the units of area, and therefore assigned a value of one for these analyses as we were more concerned with changes in richness than absolute richness. Assuming all species underwent a latitudinal shift of 2° (the approximate expected latitudinal shift over four decades; Lenoir et al., 2020) or a depth shift of 15 m (the approximate expected depth shift over four decades; Dulvy et al., 2008), we calculated the anticipated percent change in species richness communities will experience as they move into the neighboring poleward latitudinal bin in each coastline or the next depth bin in each LME. Species richness calculations based on the power‐law are highly sensitive to the *z* value or the slope of the log–log relationship of richness ~ area. We expect this parameter to vary by taxa, location, and study methodology. Therefore, we used an average value from the literature in the main text but replicated analyses for percent change in species richness using the minimum (0.175) and maximum (0.62) reported *z* value for marine fish in the Supplement for comparison (Figures S7–S9, S10a,c).

## 3 RESULTS

### 3.1 Available shelf area across depth within LMEs

**FIGURE 2:**
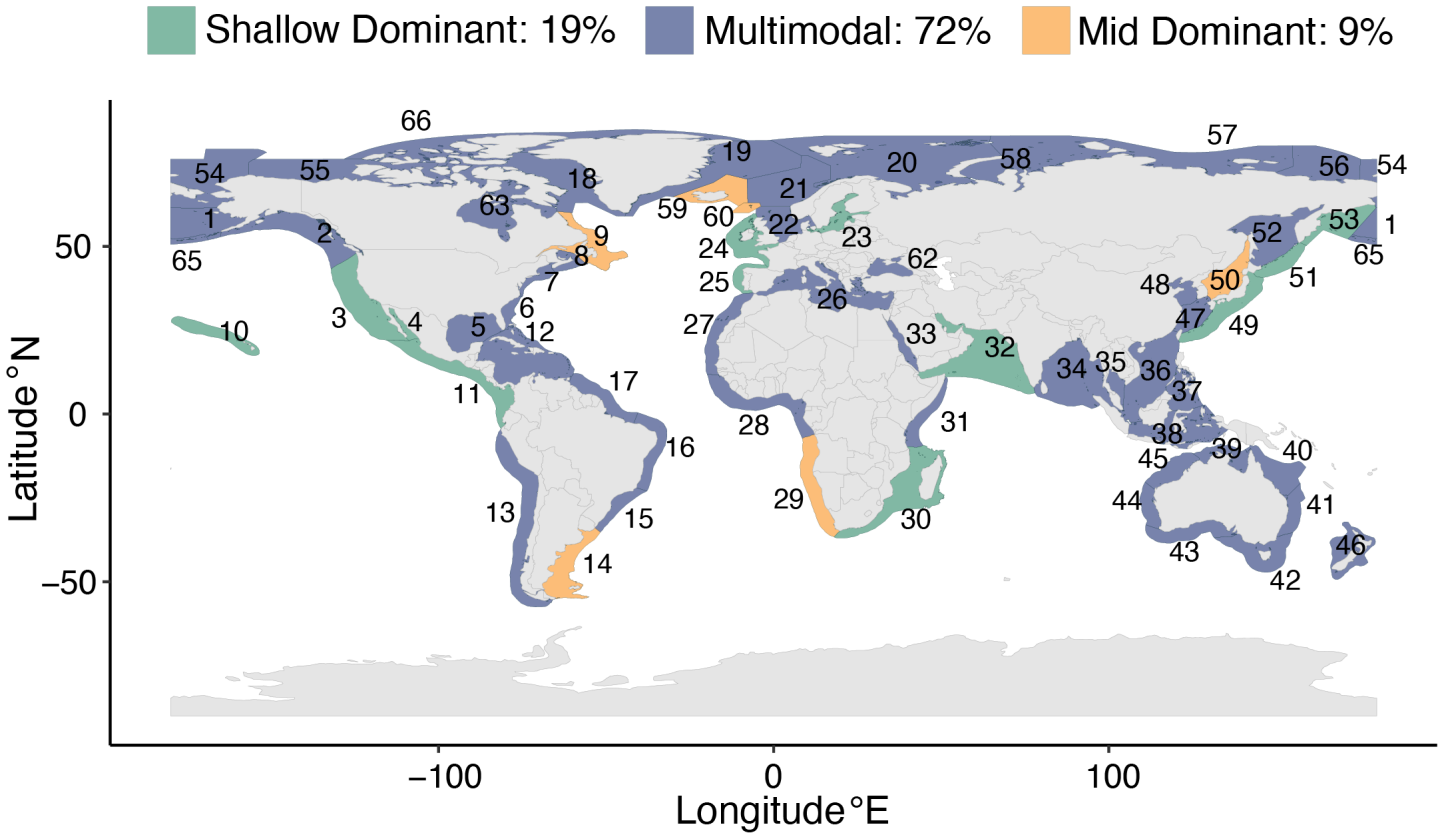
World map with 64 LMEs colored by depth distribution classification. No LMEs were classified as Deep Dominant or Uniform. See Figure S5 for LME names. See Figure S4 for alternative classifications with less conservative skew cutoffs. LME, large marine ecosystem.

Overall, 19% of LMEs were classified as Shallow‐Dominant, 9% were classified as Mid‐Dominant, and 72% were classified as Multimodal (Figures 1 and 2). No LMEs were classified as Deep‐Dominant or Uniform. Classifications were relatively insensitive to skew threshold. When a moderate skew threshold of >0.5 or <−0.5 was applied, 5% of LMEs were reclassified from Shallow‐Dominant to Mid‐Dominant (Figure S4). In response to a 15 m shift deeper, individual assemblages (defined as 15 m depth bins) experience a change in shelf area ranging from an increase of 105,000 km^2^ in part of the East Siberian Sea (LME 56) to a decrease of 230,000 km^2^ in part of the Northern Bering–Chukchi Seas (LME 54; Figures S5 and S6).

### 3.2 Available shelf area across latitude

**FIGURE 3:**
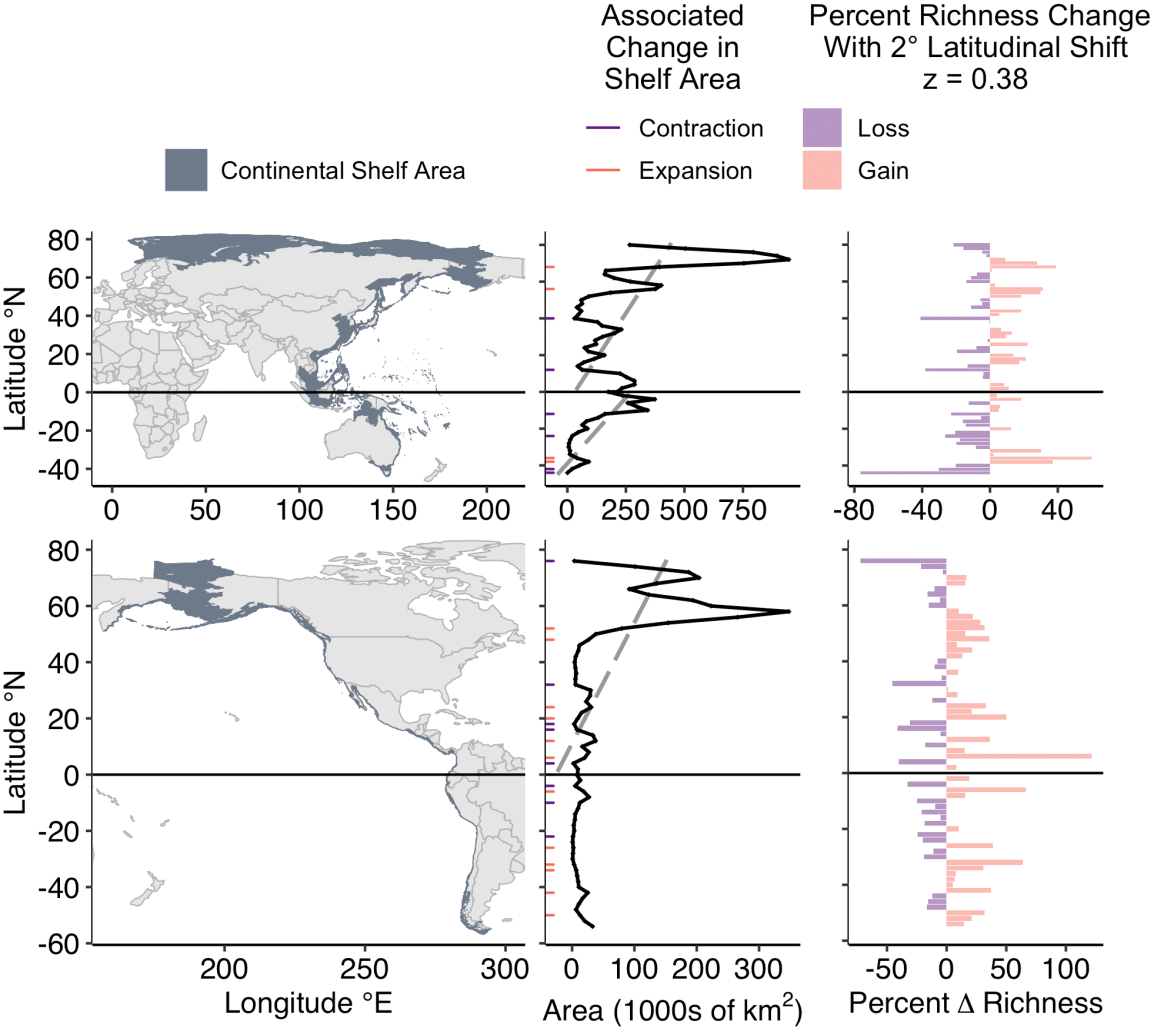
Continental shelf area availability by latitude along the western (upper) and eastern (lower) Pacific Ocean basin. Left panels show distribution of shelf area along coastlines of the Pacific. Middle panels show shelf area availability within 2° latitudinal bins in 1000s of km^2^. Poleward shifts that involve at least a halving of area (contraction) or a doubling of area (expansion) of continental shelf area are highlighted in purple and orange, respectively. Grey dashed line represents the best fit linear model for area versus latitude where the coefficient is significant (*p* < .05). Right panels show the predicted losses (purple) and gains (orange) in species richness from a 2° latitudinal shift using a SAR with an exponent of *z* = 0.38, representative of the mean for marine fish. See Figure S7 for SAR calculations using the minimum and maximum exponent values for marine fish. SAR, species area relationships.

**FIGURE 4:**
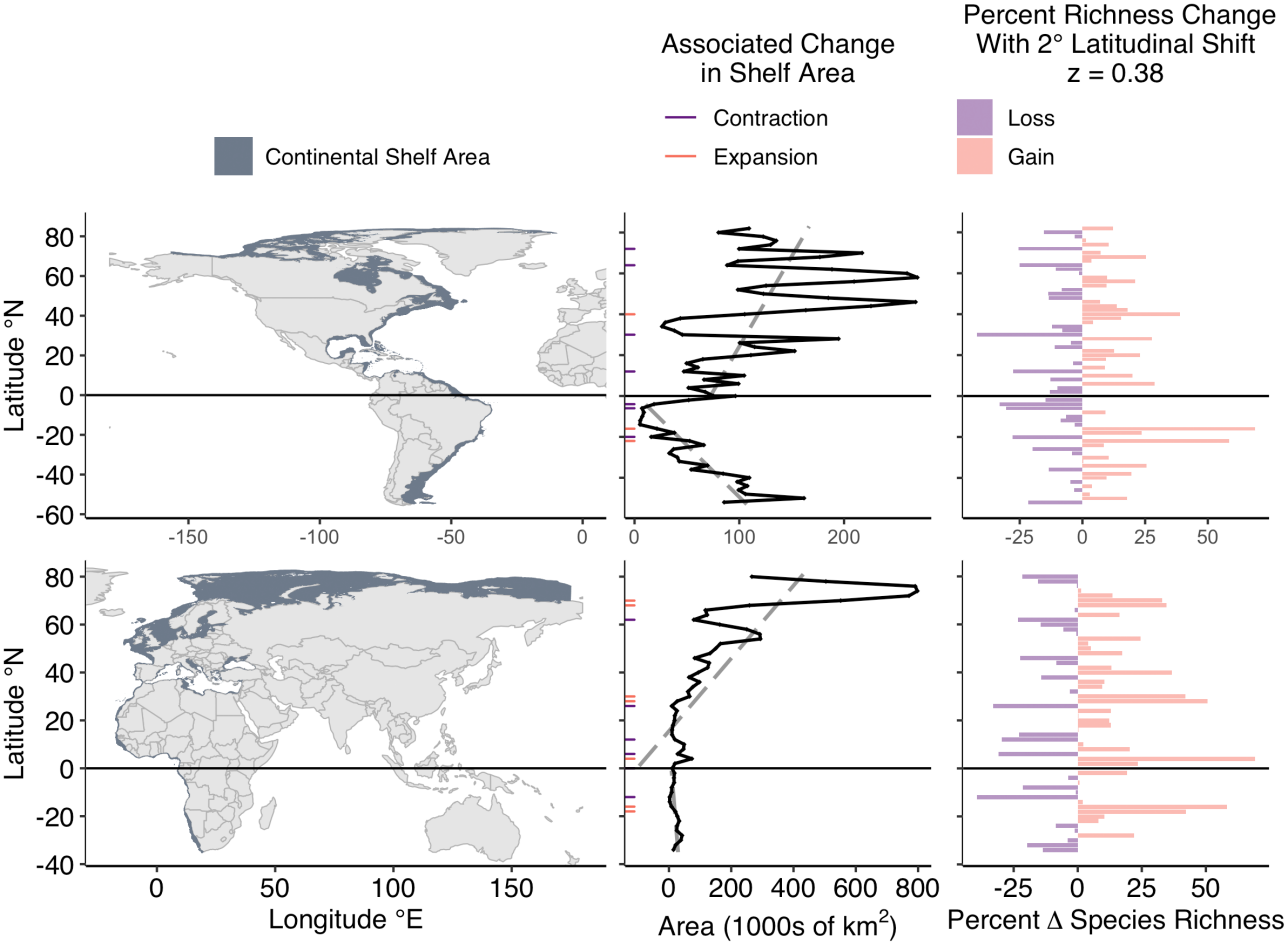
Continental shelf area availability by latitude along the western (upper) and eastern (lower) Atlantic Ocean basin. See Figure S8 for SAR calculations using the minimum and maximum exponent values for marine fish. Otherwise, see legend for Figure 3. SAR, species area relationships.

**FIGURE 5:**
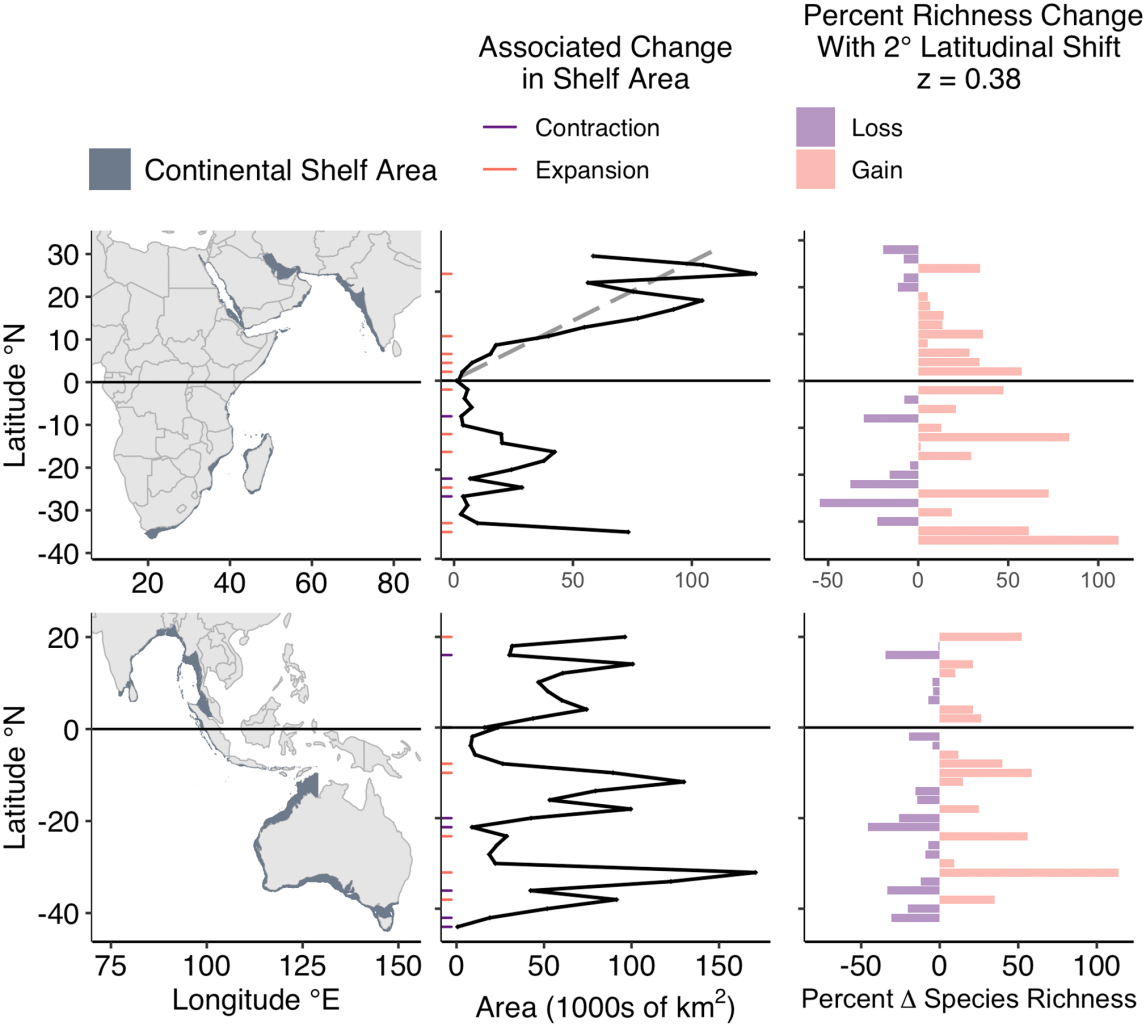
Continental shelf area availability by latitude along the western (upper) and eastern (lower) Indian Ocean basin. See Figure S9 for SAR calculations using the minimum and maximum exponent values for marine fish. Otherwise see legend for Figure 3. SAR, species area relationships.

**FIGURE 6:**
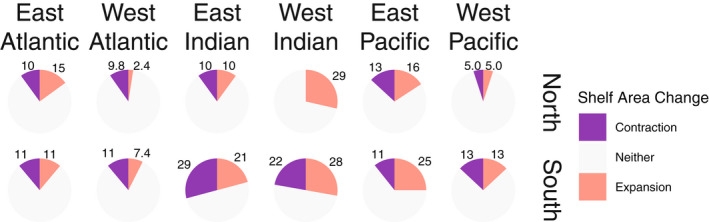
Proportion of 2° poleward shifts associated with a substantial contraction (purple) or expansion (orange) of continental shelf area for east and west coastlines of the three focal ocean basins. Poleward shifts that would experience between a halving or doubling of area are shown in grey.

Continental shelf availability varied with latitude across all six contiguous coastlines. Most coastlines exhibited instances of contraction (halving of shelf area) and all exhibited instances of expansion (doubling of shelf area) associated with 2° poleward shifts (Figures 3, 4, 5). Contractions were proportionally most common along the Southern East Indian coastline (29% of poleward shifts), and least common in the Northern West Indian coastline (no contractions; Table 1 and Figure 6). Expansions were proportionally most common along the Northern West Indian coastline (29% of poleward shifts), and least common along the Northern West Atlantic coastline (2.4% of poleward shifts; Table 1 and Figure 6).

**TABLE 1:** Percent of total 2° latitudinal bin shifts that experienced a doubling (expansion) or halving (contraction) in area

Coastline	Hemisphere	% of 2° shifts contractions	% of 2° shifts expansions	Coefficient km^2^/1° latitude	*p*‐value
East Atlantic	Northern	10	15	6600	3.6 × 10^−8^
East Atlantic	Southern	11	11	610	.014
East Indian	Northern	10	10	1310	.31
East Indian	Southern	29	21	−420	.49
East Pacific	Northern	13	16	2350	3.2 × 10^−5^
East Pacific	Southern	11	25	180	.10
West Atlantic	Northern	10	2.4	1130	.0070
West Atlantic	Southern	11	7.4	1980	4.0 × 10^−6^
West Indian	Northern	0	29	4030	.00011
West Indian	Southern	22	28	700	.11
West Pacific	Northern	5.0	5.0	5090	.00031
West Pacific	Southern	13	13	−7290	2.3 × 10^−6^

*Note*: Coefficient and *p*‐value of linear model for area versus latitude is also reported for each hemisphere‐region combination.

The majority of coastlines exhibited significant relationships between continental shelf area and latitude (Table 1; Figures 3, 4, 5). On average, shelf area decreased towards the poles along the coastline of the Southern West Pacific. In contrast, shelf area increased towards the poles along coastlines of the Northern West Pacific, the Northern East Pacific, the Southern West Atlantic, the Northern West Indian, the Northern East Atlantic, the Northern West Atlantic, and the Southern East Atlantic. No significant relationship between latitude and continental shelf area was found along the coastlines of the Southern West Indian, Southern East Indian, Northern East Indian, or Southern East Pacific (Table 1).

### 3.3 Expected percent change in species richness

Defining an assemblage as the species within a 2° latitudinal band that then shifts 2° poleward and ignoring shifts into the Southern Ocean, SAR predicted percent changes ranging from a 76% decrease in richness for part of the Southern East Indian and the Southern West Pacific to a 122% increase in richness for part of the Northern East Pacific (Figures 3 and 5). Varying the *z* value in the power‐law of the SAR impacted the magnitude of percent change in richness, but not the overall patterns (Table S1 & Figures S7–S9).

Defining an assemblage as the species within a 15 m depth band that then shifts 15 m deeper, SAR predicted percent changes ranging from a 78% decline in richness for part of the Northeast US (LME 7) and a 119% increase in richness for part of the West Bering Sea (LME 53) (Figure S10b). Again, varying the *z* value in the power‐law of the SAR impacted the magnitude of species gains and losses, but not the overall patterns (Table S2 & Figure S10a,c).

## 4 DISCUSSION

Continental shelf area is a limiting resource for a diverse array of marine organisms that depend on shallow and structured zones often with high productivity and biodiversity (Buhl‐Mortensen et al., 2012; García‐Reyes et al., 2015; Townsend et al., 2004). As species shift deeper and poleward in response to climate change, we expect the continental shelf available to them to change depending on regional bathymetry. Shelf area serves as a first‐degree constraint on successful range shifts, but to our knowledge, this is the first assessment of continental shelf area variation by depth and latitude. Similar to terrestrial mountain ranges (Elsen & Tingley, 2015), the majority of marine ecosystems do not exhibit a monotonic decrease in continental shelf area as species move deeper. Instead, there is tremendous variation in how shelf area availability varies by latitude. Whether range shifts across and down the continental shelf will lead to an opportunity for growth or decline at the level of the species and the community depends on regional bathymetry.

### 4.1 Shelf area availability across depths and latitudes

In contrast to the global trend, movement deeper onto the continental shelf does not always coincide with a loss in shelf area at the regional scale. For most LMEs, shelf area is either most abundant at moderate depths or there are multiple depths at which shelf area is most readily available. As a result, the change in shelf area availability as species shift deeper will be regionally specific due to differing geomorphology. Similar results were found to be true when assessing how continental shelf area will change with projected sea level rise (Holland, 2012). This pattern of regional variability also matches those for area at elevation across terrestrial mountain ranges (Elsen & Tingley, 2015). The lack of LMEs exhibiting Deep Dominant depth distributions reveals that while regions exhibiting monotonic decreases in area with depth are uncommon, regions exhibiting monotonic increases in area with depth are nonexistent. In some regions, species shifting deeper will experience increased shelf availability, but if species are forced to move past a depth threshold, shelf availability will decline precipitously.

Across latitudes, species are likely to encounter variability in shelf area availability, including expansions in the Northern West Indian Ocean and contractions along the Southern East Indian Ocean. Larger shelf areas have the potential to support larger population sizes of individual species, in addition to higher overall species richness (Chisholm et al., 2018; MacArthur & Wilson, 1967; Melbourne & Hastings, 2008; Shaffer, 1981). The most notable contrast between continental shelf distribution in the Northern versus the Southern Hemisphere is apparent in the transition from temperate to polar regions. For species in the Northern Hemisphere, nearly continuous shelf area between the equator and the poles serves as a corridor for species to move onto the continental shelves of the Arctic Ocean. In contrast, species in the Southern Hemisphere face hundreds of kilometers of deep ocean between the most southern points of Oceania, Africa, and South America and the deep and narrow shelves of Antarctica. Shelf area continuity for species in the southern hemisphere is truncated at 55°S, while the complementary pathway for species in the northern hemisphere reaches latitudes above 80°N. This break in shelf area continuity, in tandem with the Antarctic Polar Front and the Antarctic Circumpolar Current, has limited poleward range expansion of species through evolutionary time (Rogers, 2007; Wilson et al., 2016). However, evidence is accumulating for some dispersal of plant and invertebrate species across the Antarctic Polar Front through rafting and rare long distance dispersal events, which may facilitate some range shifts of a diverse array of species despite the lack of contiguous continental shelf area (Bernardes Batista et al., 2018; Fraser et al., 2017).

Shifts in climate regime through the geologic record and subsequent changes in species distributions and richness provide context for the role of continental shelf area in shaping modern range shifts in the ocean. The Late Ordovician greenhouse–icehouse transition led to a mass extinction of an estimated 85% of marine species (Sheehan, 2001). Similar greenhouse‐icehouse transitions occurred later in the Cenozoic, but did not lead to the same magnitude of loss in species globally. This inconsistency can be partially explained by differences in the continental configurations. The Late Ordovician planet was characterized by isolated island continents, which would have limited the capacity for species to shift their ranges into more suitable habitat. In contrast, the latitudinally oriented coastlines present today were also largely formed by the Cenozoic, allowing for poleward shifts in distributions and therefore reducing overall extinction risk (Finnegan et al., 2016).

### 4.2 Impacts of shelf area availability on populations and species richness

Population sizes vary with habitat availability (Alzate et al., 2019; Halpern et al., 2005). Larger habitats provide more resources for individuals, supporting a larger population. Small populations run the risk of stochastic extinction and restricted growth due to Allee effects (Aalto et al., 2019; Hanski et al., 1996; Kuparinen et al., 2014; Opdam & Wascher, 2004; White et al., 2021). Range shifts into depths or latitudes of reduced shelf area availability may lead to local extinction or the inability to establish. In contrast, shifts into depths or latitudes of higher relative shelf area may lead to increased population growth rates as individuals take advantage of increased space and foraging opportunities.

Given that SAR suggests that the number of species scales with habitat size, we expect latitudes and depths of greater continental shelf area to support a larger number of species as niche space, resource availability, and likelihood of species arrival increase (Chisholm et al., 2018; MacArthur & Wilson, 1967; Rosenzweig, 1995). Shifts in latitude and depths have the potential to impact regional species richness as the number of species able to successfully shift is limited by continental shelf area. The anticipated changes in species richness due to variations in shelf area are tightly linked to geographic features at the local and regional scale. For one, because of the non‐linearity in SAR, we predict much more dramatic shifts in richness across latitudes and depths in regions of overall limited shelf area in comparison to regions defined by wide continental shelves. For example, changes in species richness as a result of equatorward shifts along the tropical and temperate regions of the East Pacific will reflect changes from a baseline narrow shelf area. In contrast, changes in richness along the Northern Atlantic coasts will likely be muted due to the wide continental shelves in these regions. Using SAR ignores the complexities of endemic versus cosmopolitan distributions and assumes species are randomly distributed (He & Hubbell, 2011) and our calculations must therefore be viewed cautiously. However, our analyses identify regions at a higher risk of species loss and regions that may serve as biodiversity refuges.

### 4.3 Other barriers to successful range shifts

Availability of continental shelf area acts as a first‐order constraint on movement poleward and deeper. However, other constraints will likely be important, including availability of biogenic habitat, prey, or mutualists (Brooker et al., 2007; Urban et al., 2019) and the presence of predators or competitors (Bates et al., 2014; Gilman et al., 2010; McIntosh et al., 2018; Spence & Tingley, 2020). Changes in area offers a proxy for expected trends in richness and population sizes, but these patterns can be complicated by species interactions. Movement into depths or latitudes with more shelf area may actually lead to a decline in population growth for some low trophic species due to an increased risk of predation (McIntosh et al., 2018). In the case of depth shifts, many species of plants and algae that form the foundation for many types of marine structures only grow in the photic zone. The intensity of light decreases exponentially with depth and can restrict the productivity of photosynthetic organisms like seagrasses and coral symbionts (Duarte, 1991; Kahng et al., 2019; Lesser et al., 2021). Light can also constrain latitudinal shifts because highly seasonal diel cycles lead to reduced nutrient availability and limited opportunities for visual foraging in the winter (Chen & Wang, 2016; Last et al., 2020; Ljungström et al., 2021).

Range shifts are also limited by abiotic habitats associated with continental shelves. The distribution of abiotic habitat is determined by geologic history, and therefore stationary in ecological time (Ford & HilleRisLambers, 2020; Spence & Tingley, 2020). Marine species highly dependent on a particular substrate, rugosity, or geologic feature will be limited in their ability to track temperature isotherms poleward or deeper (Champion & Coleman, 2021; Harman et al., 2003; McHenry et al., 2019), similar to how plant communities can be limited by soils (Carteron et al., 2020; Smithers & North, 2020). The potential for marine species to occupy thermally suitable continental shelves may also be limited by water characteristics as ideal temperature conditions do not guarantee tolerable pressure, oxygen, or pH conditions. A number of LMEs, including the California Current, Benguela Current, the Arabian Sea, and the Bay of Bengal have oxygen minimum zones (OMZ) that span from roughly 200 to 1000 m in depth (Al Azhar et al., 2017; Bograd et al., 2008; Zettler et al., 2009). Marine ectotherms have specific oxygen requirements to maintain effective metabolism, and therefore, most are limited to depths above the OMZ (Stramma et al., 2012). Latitudinal variation in pH can also limit successful poleward range shifts. Because of the tilt of the earth on its axis and circulation patterns in the ocean, the most acidic waters are found in polar regions. These same regions are also experiencing the fastest rates of ocean acidification, posing a challenge for growth and reproduction for a wide array of marine ectotherms, most notably calcifying species (Fabry et al., 2009; Qi et al., 2017; Yara et al., 2012). Even when continental shelf area is available, range shifts may be restricted due to additional biotic and abiotic factors.

Human activities have altered continental shelf characteristics across the globe. These transformations may limit the capacity for species to traverse continental shelves even when abundant area is available. For example, regular bottom trawling leads to a rise in suspended solids, and a reduction of seafloor microhabitats such as boulders, sand waves, and biogenic structures such as reefs and anemones (de Marignac et al., 2009; Oberle et al., 2016). As another example, high densities of impervious surfaces in coastal areas coupled with fertilizer application has led to algal blooms and a subsequent decline in pH within nearshore habitats (Conway, 2007; Gledhill et al., 2015). The potential for human action to restrict species movement to and establishment within otherwise suitable habitats emphasizes the essential role of marine protected areas in climate‐ready ocean management (Harvey et al., 2018; Holsman et al., 2019).

### 4.4 Regions of opportunity, and regions of concern

We have highlighted regions expected to experience reductions in continental shelf area where shifting species may face challenges. These areas include Shallow‐Dominant LMEs and areas of coastline where substantial contractions in continental shelf area occur. Species that are experiencing warming and are living in regions with limited shelf area in surrounding depths or latitudes are most at risk. For example, the Arabian Sea exhibits a Shallow‐Dominant shelf bathymetry and its semi‐enclosed basin limits latitudinal movement (Ben‐Hasan & Christensen, 2019). Warming, coupled with declining oxygen concentrations has already led to a rise in Harmful Algal Blooms and resultant fish kills in this relatively understudied region (Al Azhar et al., 2017; Ben‐Hasan & Christensen, 2019; Harrison et al., 2017). Marine species in the Arabian Sea are already at higher risk of extinction due to above average climate velocity and human impacts (Finnegan et al., 2015). Other regions pose risks to resident species due to less conspicuous latitudinal restrictions. One example is the Sea of Japan, which has experienced consistent warming of up to 0.5°C since the 1960s and shoaling of the OMZ (Kim et al., 2001). On top of rising temperatures and declining access to oxygenated waters, continental shelf area in this region is limited. Shelf area contracts 75% as species shift from 38° to 40° N, and remains limited until 56° N when the wide shelf of the Bering Sea becomes accessible. However, the cumulative intensity of climate velocity and human impacts is lower in this region in comparison to the Arabian Sea and therefore species may be able to persist despite limited transitional shelf availability (Finnegan et al., 2015).

We have also highlighted regions where species may benefit from continental shelf expansions as they track temperature isotherms deeper and poleward. LMEs across the globe exhibit opportunities for range expansion into deeper continental shelf areas, if light and other factors are not limiting. The rapidly warming North Sea's (LME 22) Multimodal depth distribution may partially explain why many species have successfully shifted deeper in this region (Dulvy et al., 2008). Additionally, species moving poleward along each coastline will have opportunities to take advantage of expansions in shelf area. For example, species shifting from Brazil south towards the coasts of Uruguay and Argentina will gain access to wider continental shelves. This rise in available area, coupled with relatively low rates of climate velocity and human impact may help support the persistence of species in this region (Finnegan et al., 2015). Similarly, species shifting poleward along the eastern coast of Australia will gain access to more shelf area as they move with the Leeuwin Current into the Great Australian Bight along the continent's southern coast. Through geologic history, this region has experienced relatively high extinction rates, but access to ample shelf area may help mitigate extinction risk (Finnegan et al., 2015).

This information can be used to prioritize conservation efforts at a broad scale, focusing on regions where species will experience the greatest reductions in seafloor area following predicted range shifts. The immense loss of marine species in the Late Ordovician highlights the essential role of latitudinally oriented continental shelf area in allowing species to avoid extinction by moving towards cooler waters, which translates to a need for latitudinally oriented protected areas (Fredston‐Hermann et al., 2018; Saupe et al., 2020). In addition to protecting critical biotic and abiotic habitats, we recommend that practitioners also prioritize connectivity in regions experiencing contractions in shelf area to reduce the risk of extinction and ease movement poleward and deeper (Carr et al., 2017; Green et al., 2015). Limited access to biophysical data and analytical tools can restrict the capacity for communities and nations to conduct systematic site selection for protected areas (Hansen et al., 2011). Areas identified by this study as contraction zones and Shallow‐Dominant LMEs are strong candidates for protection that would help support ecologically and economically important species during this era of unprecedented environmental change. In contrast, areas identified as expansion zones and as either Mid‐Dominant or Multimodal LMEs may act as refugia and as locations for blue economy development if managed proactively (Queirós et al., 2021). Designation of protected areas that include latitudinal corridors and implementation of climate‐ready management in high risk regions to limit habitat degradation, pollution, and resource extraction may help facilitate successful range shifts despite limited continental shelf availability (Frazão Santos et al., 2020; Meyer‐Gutbrod et al., 2018; Mills et al., 2013).

## AUTHOR CONTRIBUTIONS

Malin L. Pinsky and Rebecca L. Selden conceptualized study; Zoë J. Kitchel and Hailey M. Conrad wrote the code and analyzed the data; Zoë J. Kitchel and Hailey M. Conrad wrote the first draft of the manuscript, and all authors contributed substantially to revisions.

## CONFLICT OF INTEREST

The authors declare there is no conflict of interest.

## Supporting information


Appendix S1

## Data Availability

All data used in the following analyses are available publicly. ETOPO1 bathymetric data are available from NOAA, https://www.ncei.noaa.gov/access/metadata/landing‐page/bin/iso?id=gov.noaa.ngdc.mgg.dem:316. Continental shelf delineation is available from Blue Habitats, https://www.bluehabitats.org/?page_id=58. Large Marine Ecosystem shapefiles are available from the USGS https://www.sciencebase.gov/catalog/item/55c77722e4b08400b1fd8244. FAO Major Fishing Areas are available from the FAO, http://www.fao.org/geonetwork/srv/en/main.home?uuid=ac02a460‐da52‐11dc‐9d70‐0017f293bd28. Code and aggregated data are available on the public GitHub repository, https://github.com/zoekitchel/shelf_habitat_distribution. For convenience, raw data products are available on Zenodo at the following DOI: 10.5281/zenodo.6557756
