# Supplementary material for: The role of continental shelf bathymetry in shaping marine range shifts in the face of climate change
Source: Glob Chang Biol. 2022 Jun 13;28(17):5185–99. doi: 10.1111/gcb.16276 (PMC9540106; doi:10.1111/gcb.16276)

Kitchel et al. Bathymetric limits on range shifts

Supplemental Materials

Table S1 Pearson's correlation coefficient between alternate calculations of percent change in species richness using the minimum (z = 0.175), mean (z = 0.38), and max (z = 0.62) reported exponent values for 2˚ latitudinal shifts (Levin et al. 2009).

	Percent Change Species Richness z = 0.175	Percent Change Species Richness z = 0.38	Percent Change Species Richness z = 0.62
Percent Change Species Richness z = 0.175	1	0.97	0.91
Percent Change Species Richness z = 0.38		1	0.99
Percent Change Species Richness z = 0.62			1

Table S2. Pearson's correlation coefficient between alternate calculations of percent change in species richness using the minimum (z = 0.175), mean (z = 0.38), and max (z = 0.62) reported exponent values for 15 m depth shifts (Levin et al. 2009).

	Percent Change Species Richness z = 0.175	Percent Change Species Richness z = 0.38	Percent Change Species Richness z = 0.62
Percent Change Species Richness z = 0.175	1	0.99	0.97
Percent Change Species Richness z = 0.38		1	0.99
Percent Change Species Richness z = 0.62			1

Raster versus Projected Shapefile Comparison

We conducted a comparison to confirm that calculating continental shelf area from unprojected rasters instead of projected shapefiles would not change conclusions of the latitudinal and depth analyses. We tested the difference in area calculations for regions that were likely to present the largest differences due to high latitudes—East Atlantic Ocean for latitudinal shifts and High Arctic Canada/Greenland (LME 66) for depth shifts.

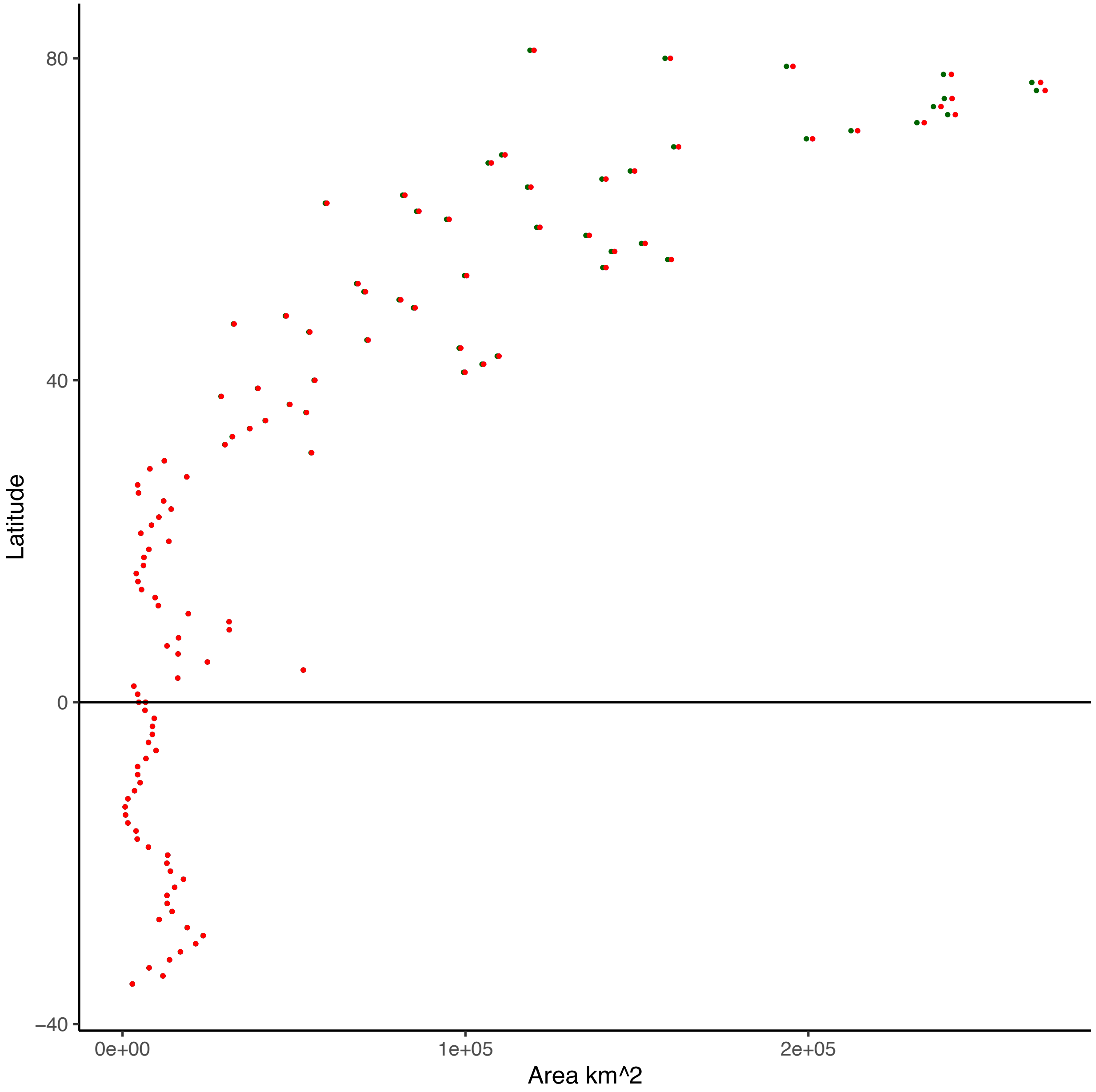

Figure S1. Latitude versus continental shelf area calculated by applying raster::area() function to a projected shapefile for the 2˚ latitudinal bin using the equal area projection (red) and to an unprojected raster for the 2˚ latitudinal bin (green). The two alternative methods lead to a Pearson correlation coefficient of 0.99 between the two sets of values.

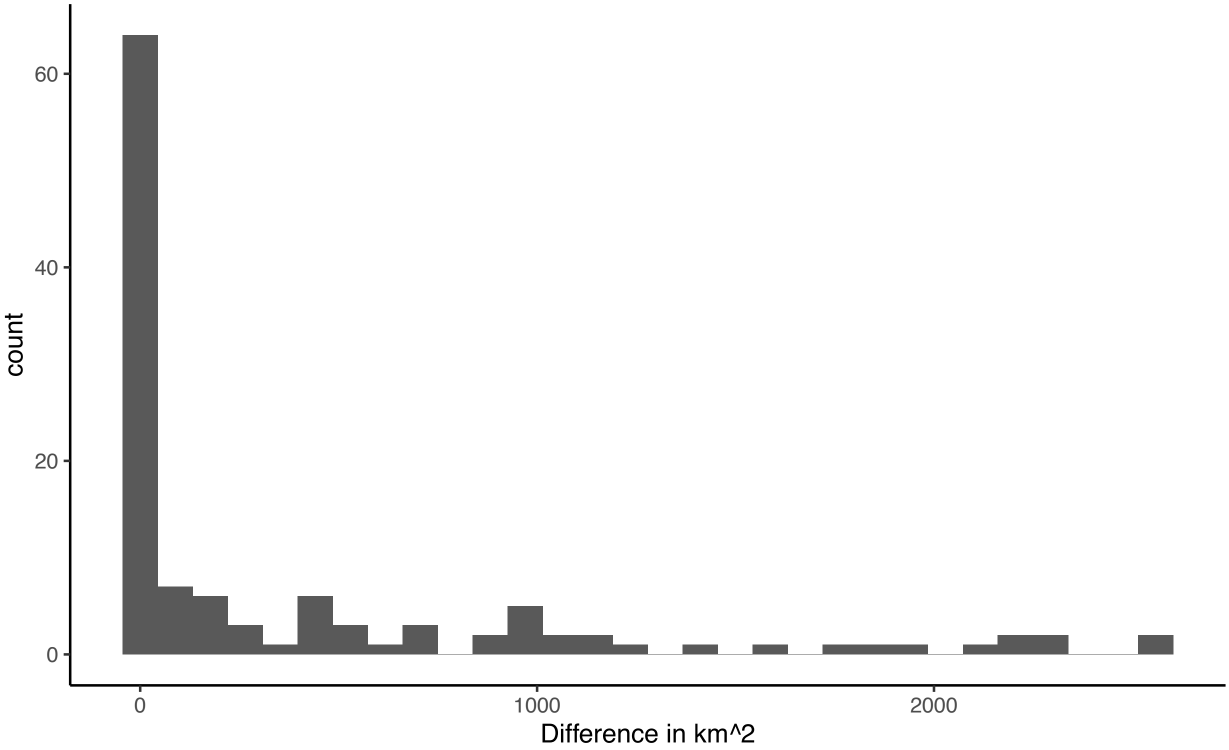

Figure S2. The distribution of differences between calculated area of 2˚ latitudinal bins using the unprojected raster and the projected shapefile. The differences range from 0 to 2558 km^2, with a mean of 405 km^2 and a median of 30 km^2. The measured differences scale with the size of the 2˚ latitudinal bin. At its maximum, the measured difference between the two methodologies is 1% of the total area of the 2˚ latitudinal bin calculated using the unprojected raster method.

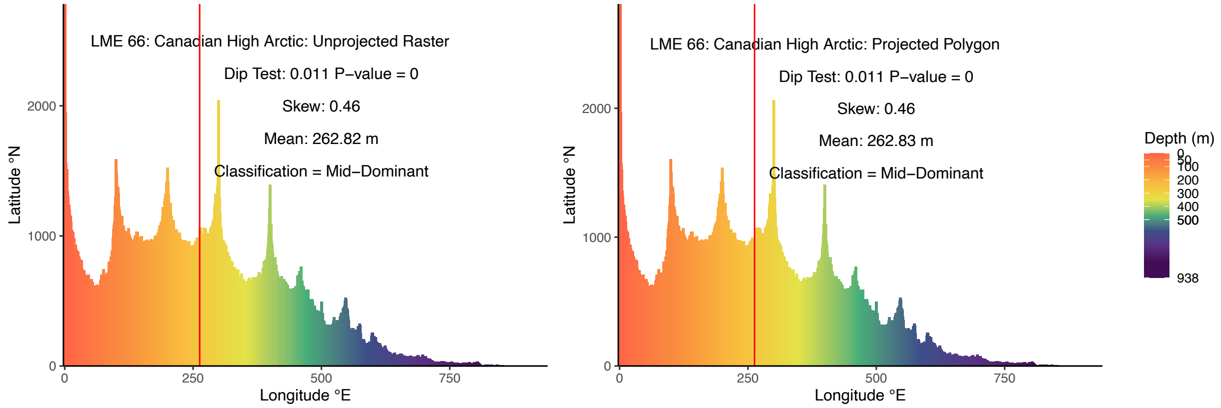

Figure S3. Hypsometric curves for continental shelf area by depth using area calculated from the unprojected raster (left) and from the projected polygon (right). For both methodologies, LME 66 is classified as Mid-Dominant.

Additional Supplemental Figures

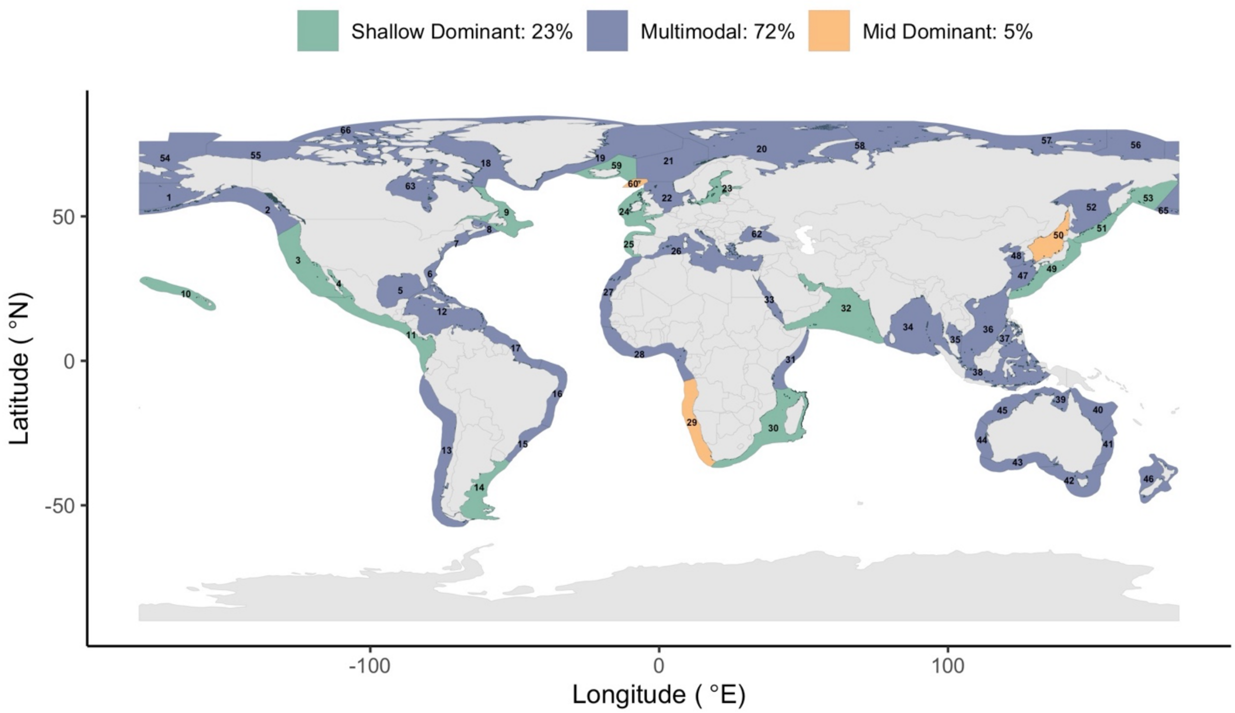

Figure S4. Figure 2 in main text but with less conservative skew classification (-0.5/0.5 cutoff). World map with 64 LMEs colored by depth distribution classification. No LMEs were classified as Deep Dominant or Uniform. Three LMEs (5%) reclassified from Mid-Dominant to Shallow-Dominant

Figure S5. Depth maps for all 64 LMEs included in the analysis. Color represents depth ranging from orange (shallow) to purple (deep).

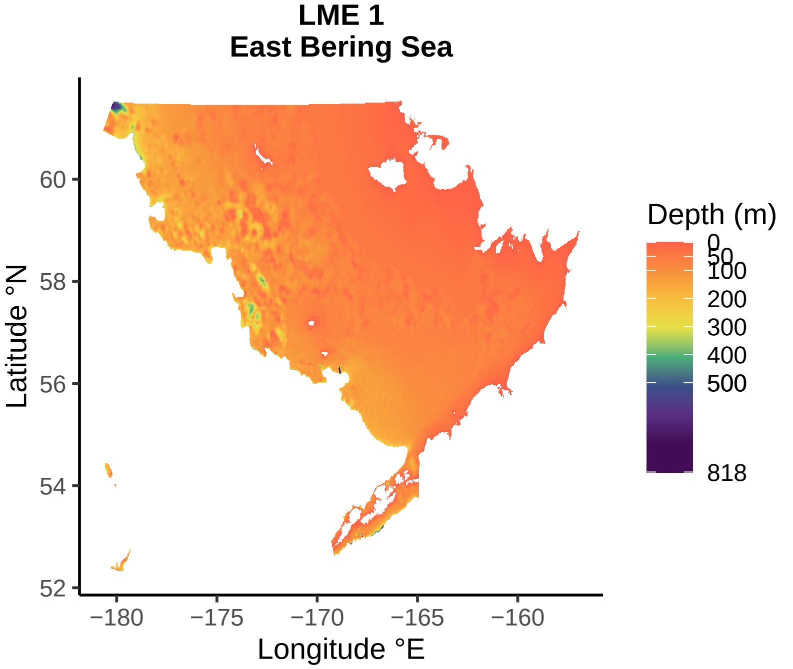
LME 1
East Bering Sea
Latitude °N
Longitude °E
60
58
56
54
52
−180
−175
−170
−165
−160
Depth (m)
0
50
100
200
300
400
500
818

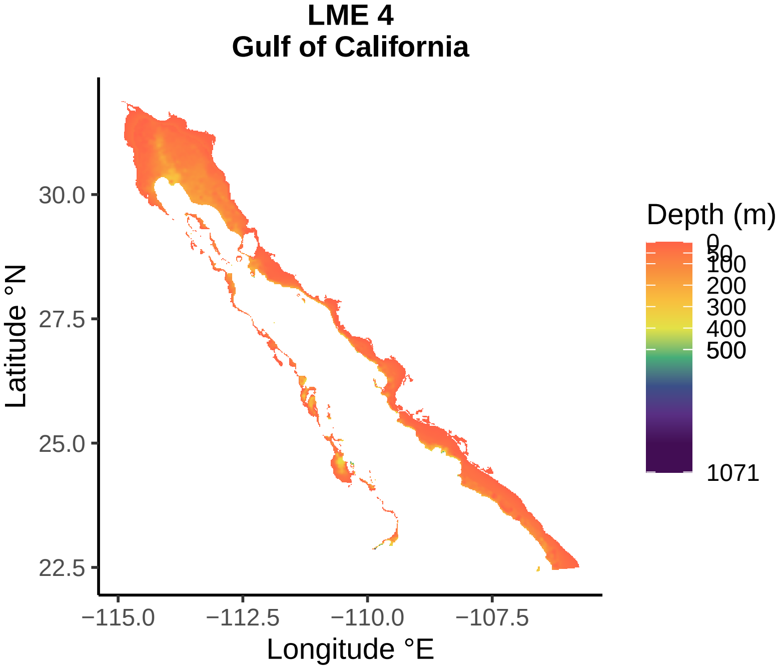
LME 4
Gulf of California
Latitude °N
Longitude °E
30.0
27.5
25.0
22.5
−115.0
−112.5
−110.0
−107.5
Depth (m)
0
50
100
200
300
400
500
1071

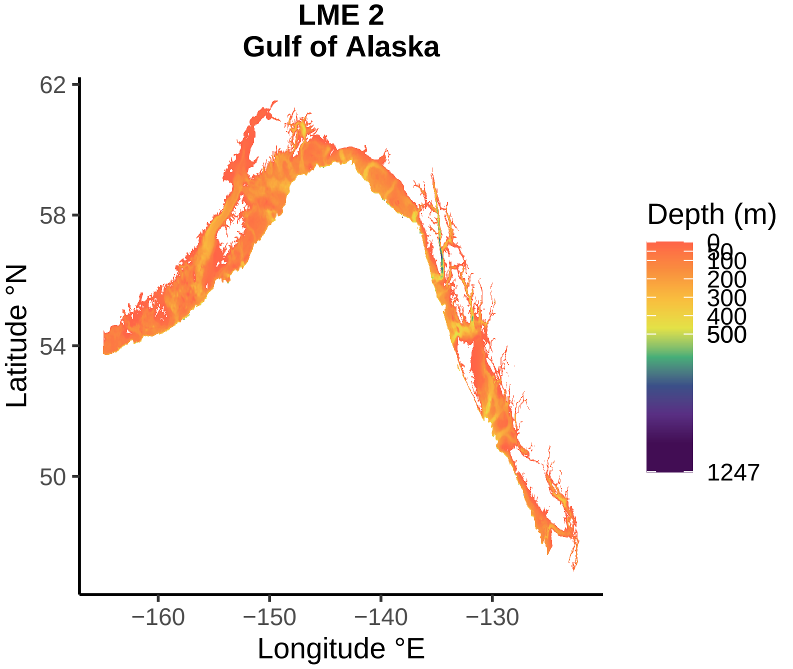
LME 2
Gulf of Alaska
Latitude °N
Longitude °E
62
58
54
50
−160
−150
−140
−130
Depth (m)
0
50
100
200
300
400
500
1247

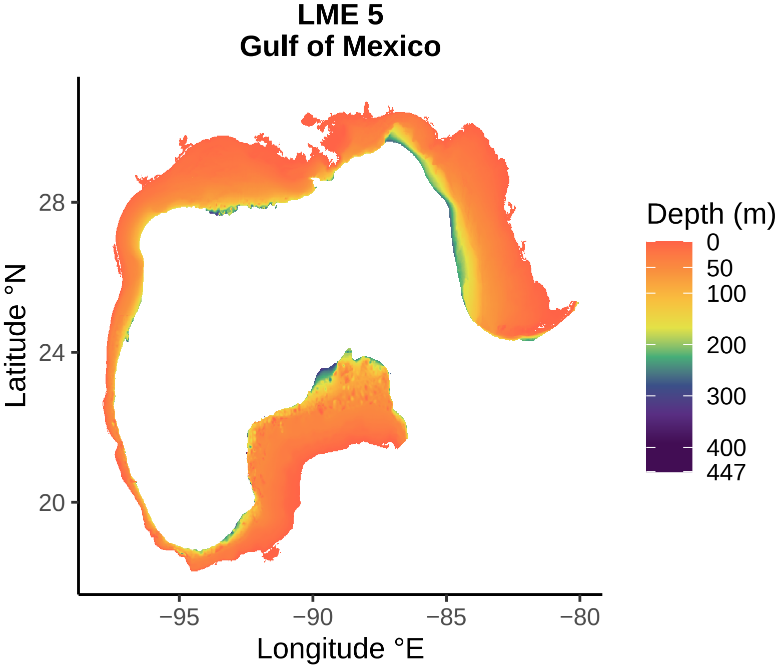
LME 5
Gulf of Mexico
Latitude °N
Longitude °E
28
24
20
−95
−90
−85
−80
Depth (m)
0
50
100
200
300
400
447

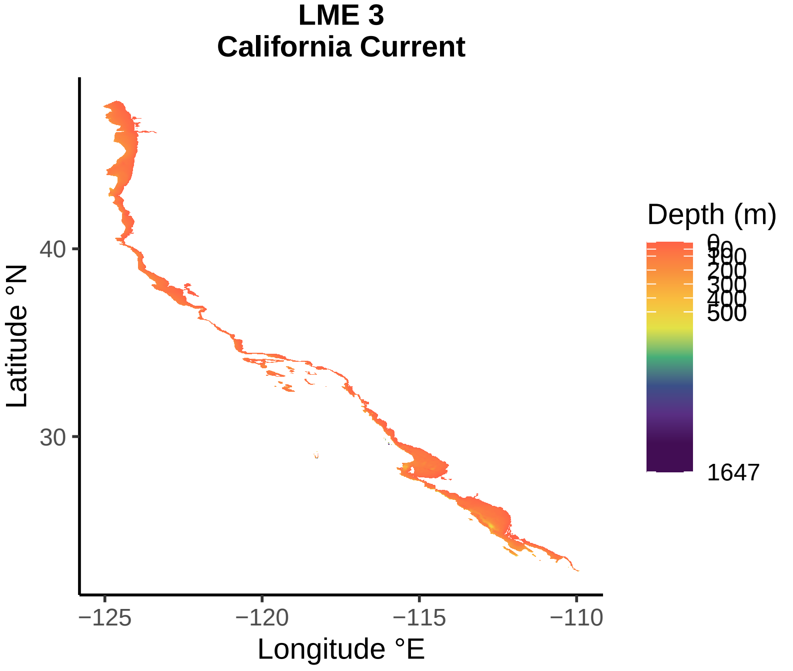
LME 3
California Current
Latitude °N
Longitude °E
40
30
−125
−120
−115
−110
Depth (m)
0
50
100
200
300
400
500
1647

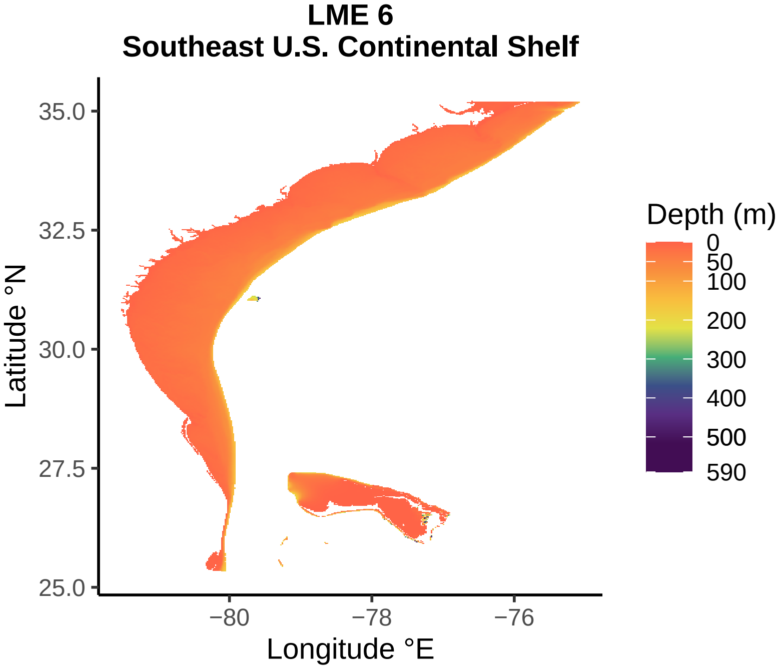
LME 6
Southeast U.S. Continental Shelf
Latitude °N
Longitude °E
35.0
32.5
30.0
27.5
25.0
−80
−78
−76
Depth (m)
0
50
100
200
300
400
500
590

LME 7
Northeast U.S. Continental Shelf

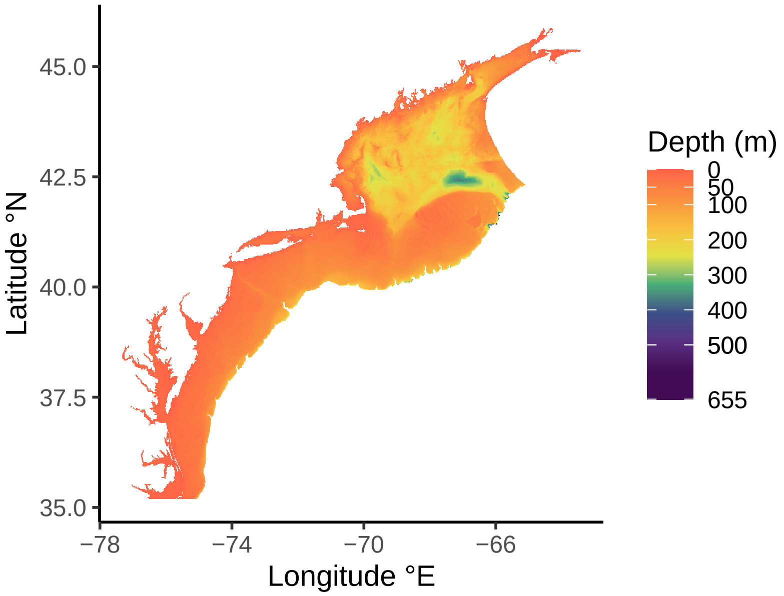

LME 10
Insular Pacific−Hawaiian

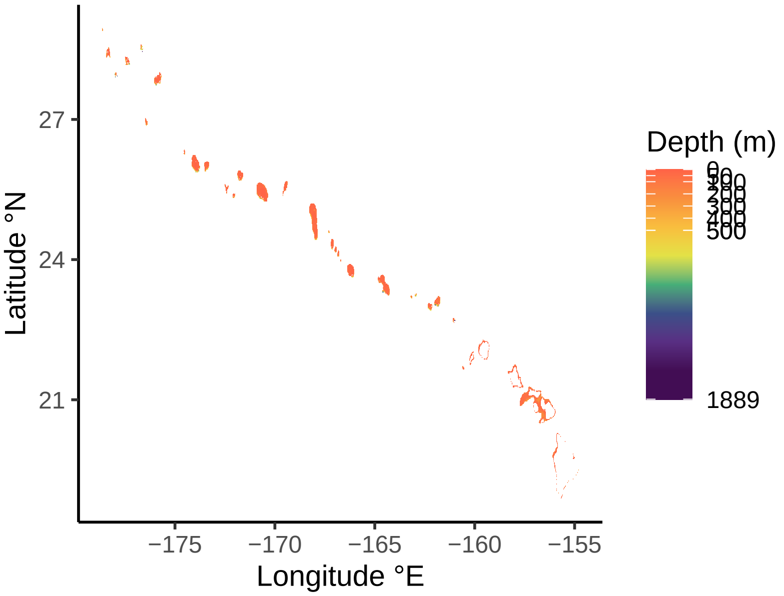

LME 8
Scotian Shelf

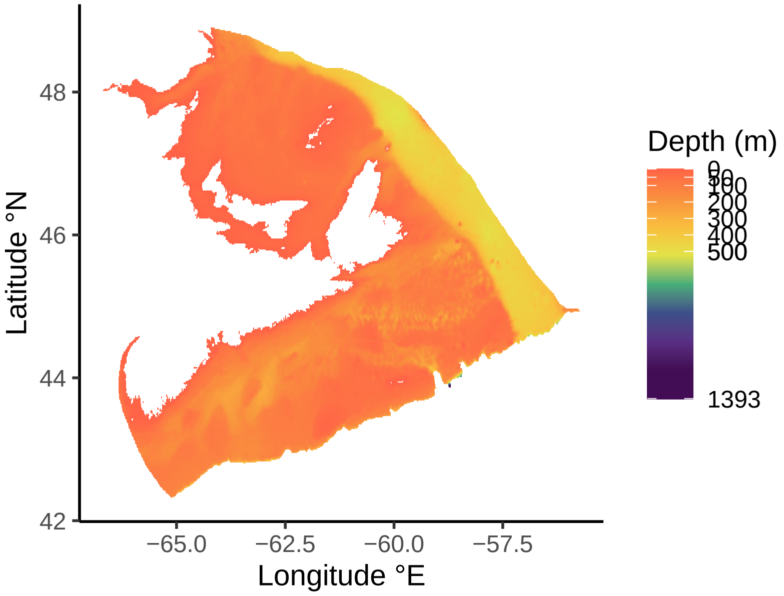

LME 11
Pacific Central−American Coastal

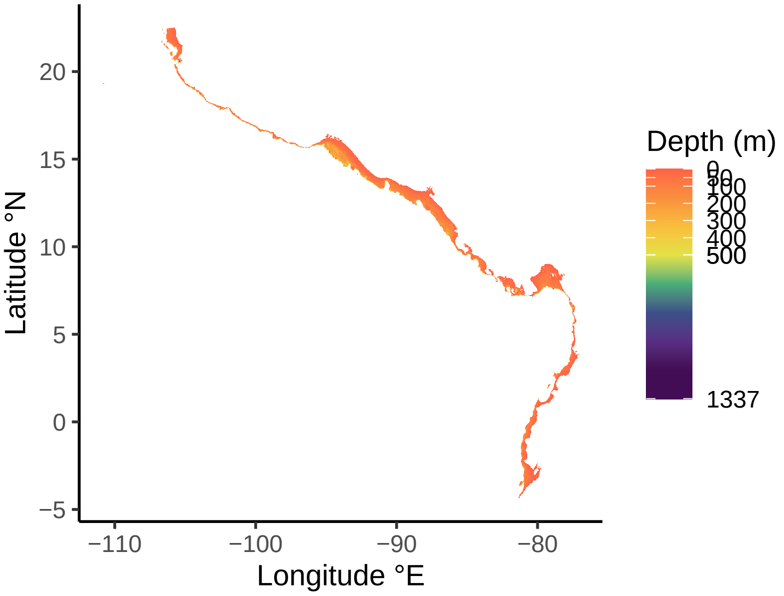

LME 9
Labrador − Newfoundland

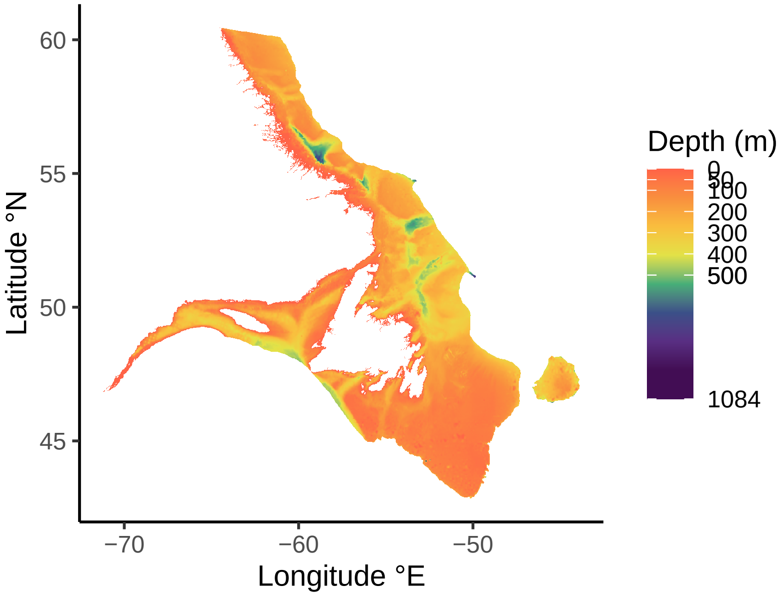

LME 12
Caribbean Sea

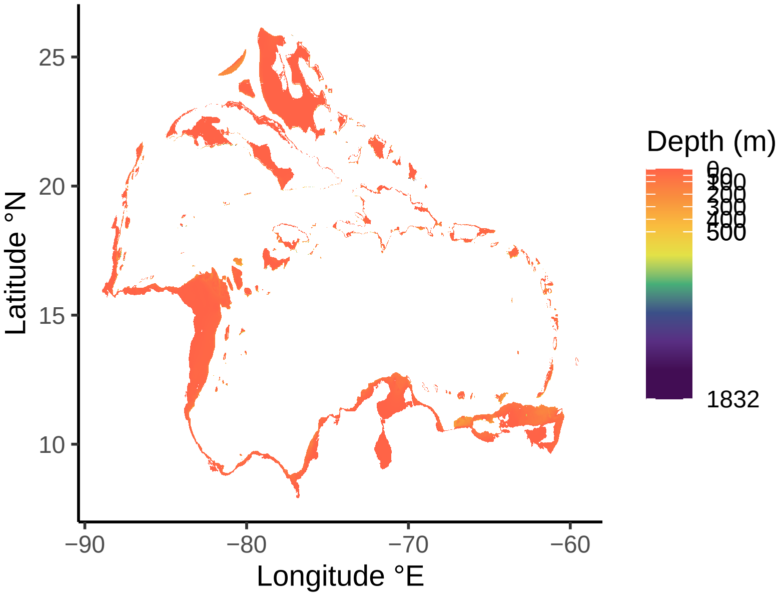

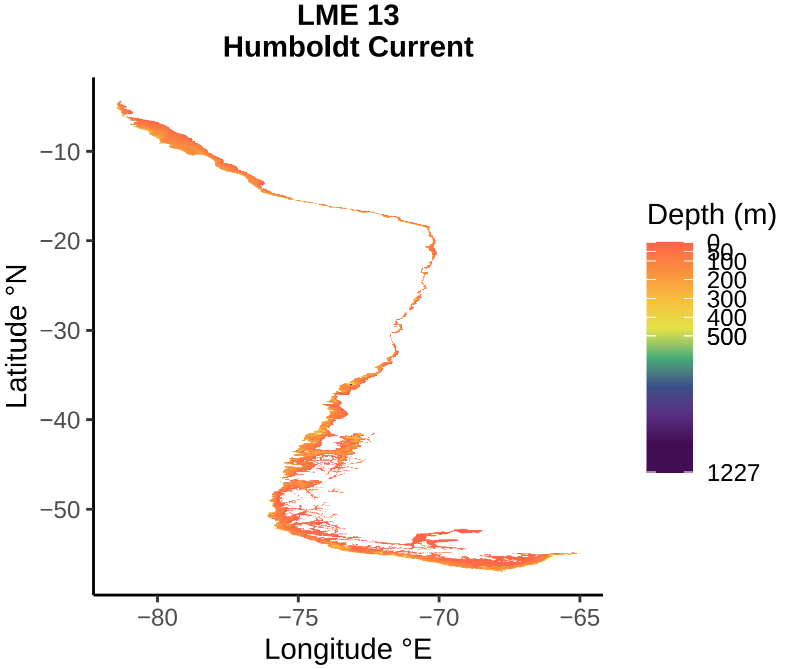
LME 13
Humboldt Current
Latitude °N
−10
−20
−30
−40
−50
−80
−75
−70
−65
Longitude °E
Depth (m)
0
50
100
200
300
400
500
1227

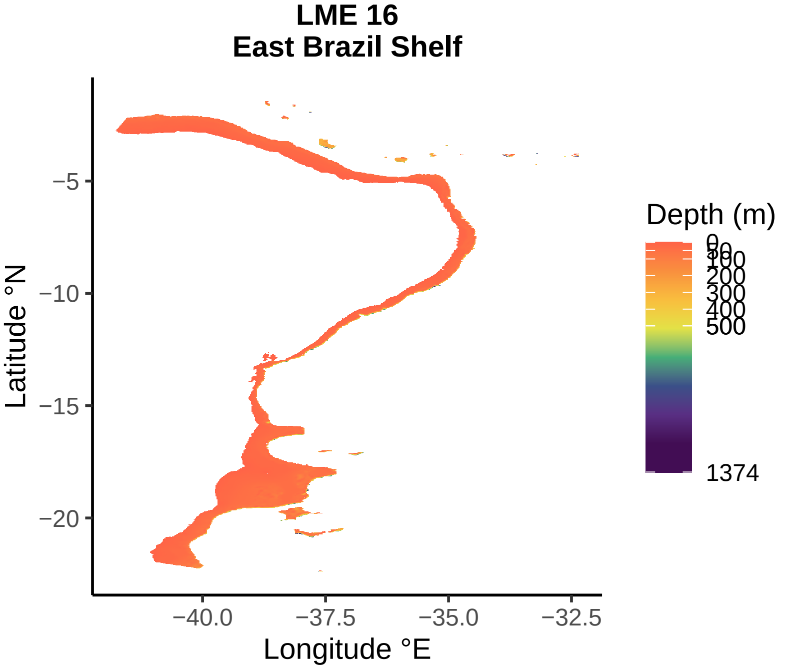
LME 16
East Brazil Shelf
Latitude °N
−5
−10
−15
−20
−40.0
−37.5
−35.0
−32.5
Longitude °E
Depth (m)
0
100
200
300
400
500
1374

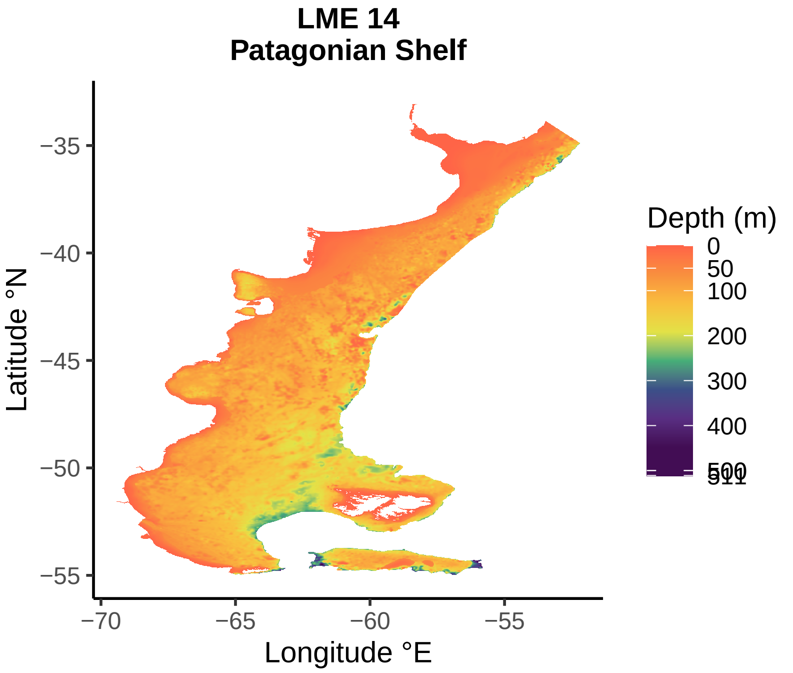
LME 14
Patagonian Shelf
Latitude °N
−35
−40
−45
−50
−55
−70
−65
−60
−55
Longitude °E
Depth (m)
0
50
100
200
300
400
500
511

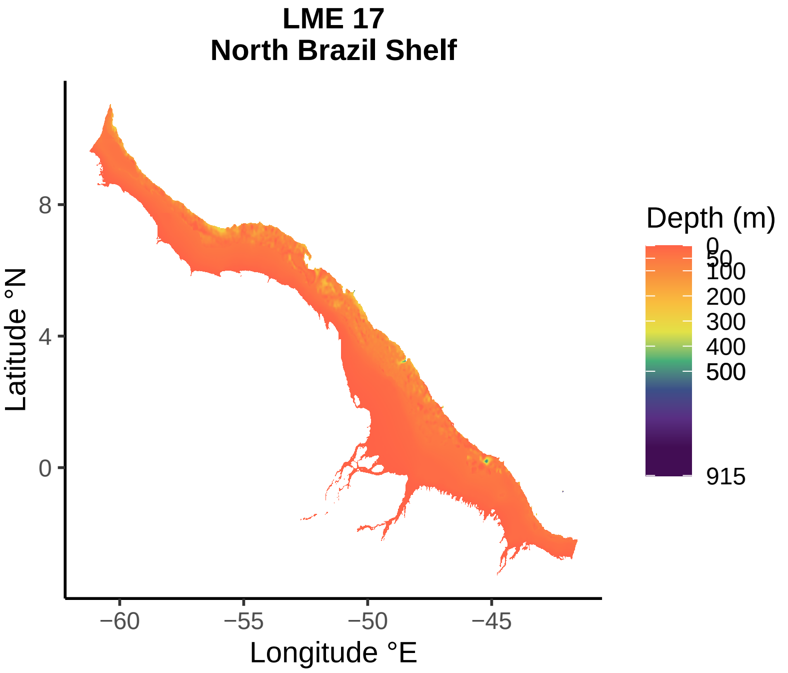
LME 17
North Brazil Shelf
Latitude °N
8
4
0
−60
−55
−50
−45
Longitude °E
Depth (m)
0
50
100
200
300
400
500
915

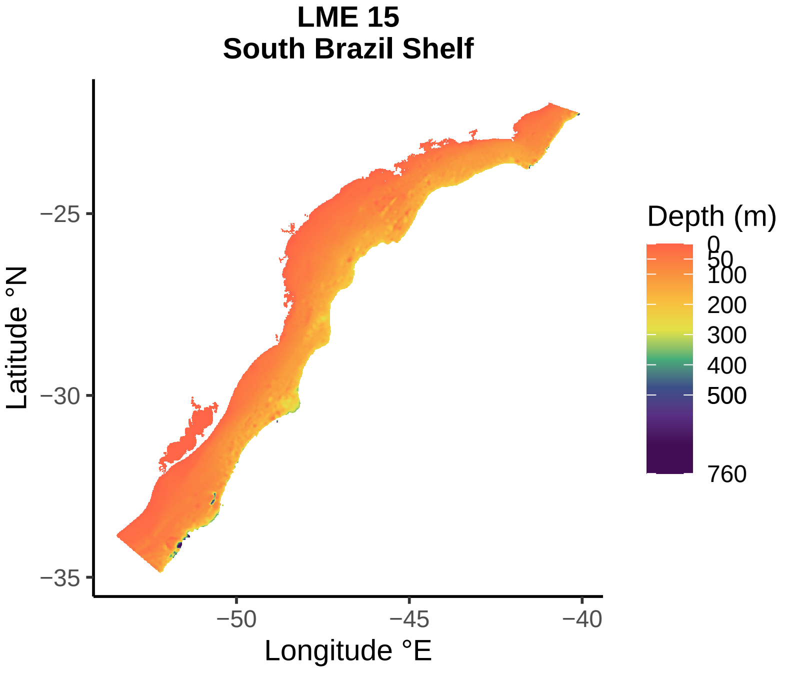
LME 15
South Brazil Shelf
Latitude °N
−25
−30
−35
−50
−45
−40
Longitude °E
Depth (m)
0
50
100
200
300
400
500
760

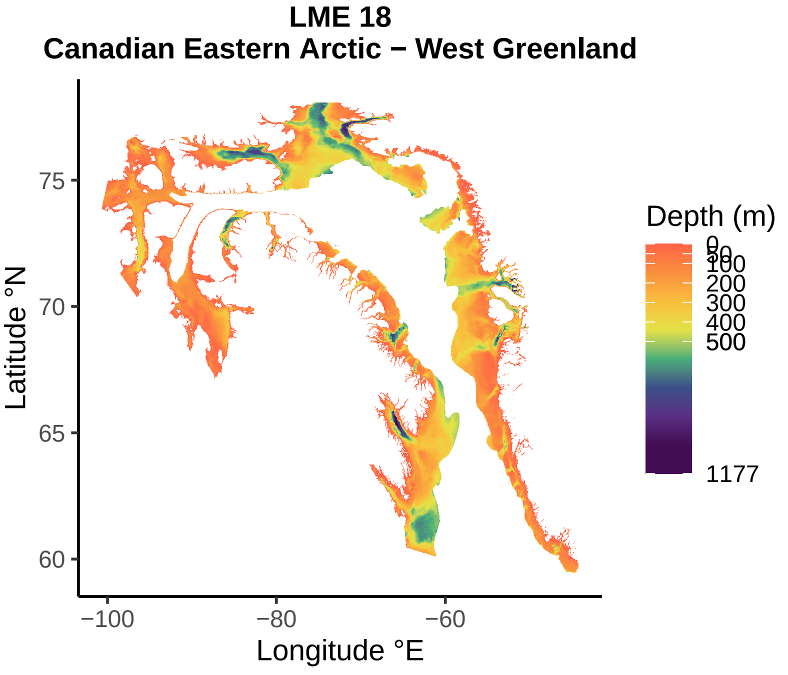
LME 18
Canadian Eastern Arctic − West Greenland
Latitude °N
75
70
65
60
−100
−80
−60
Longitude °E
Depth (m)
0
100
200
300
400
500
1177

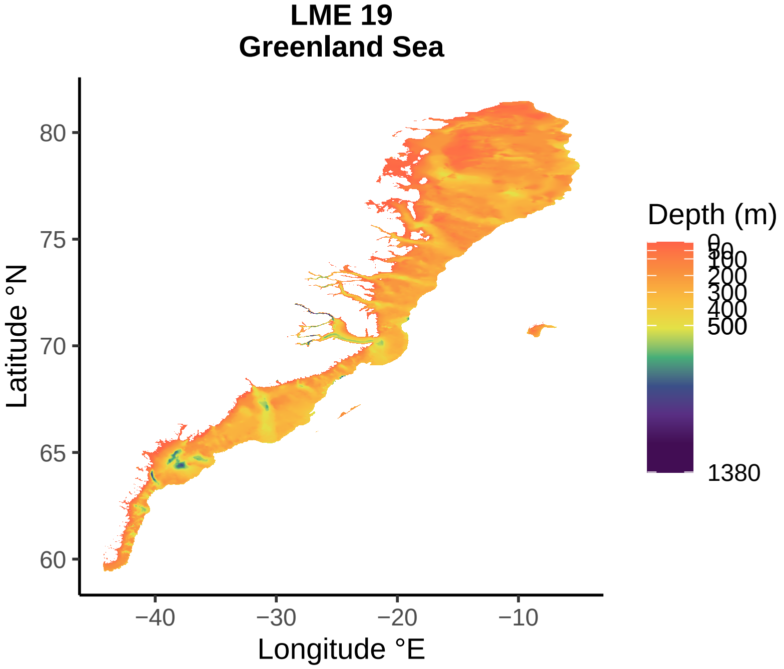
LME 19
Greenland Sea
Latitude °N
80
75
70
65
60
−40
−30
−20
−10
Longitude °E
Depth (m)
0
50
100
200
300
400
500
1380

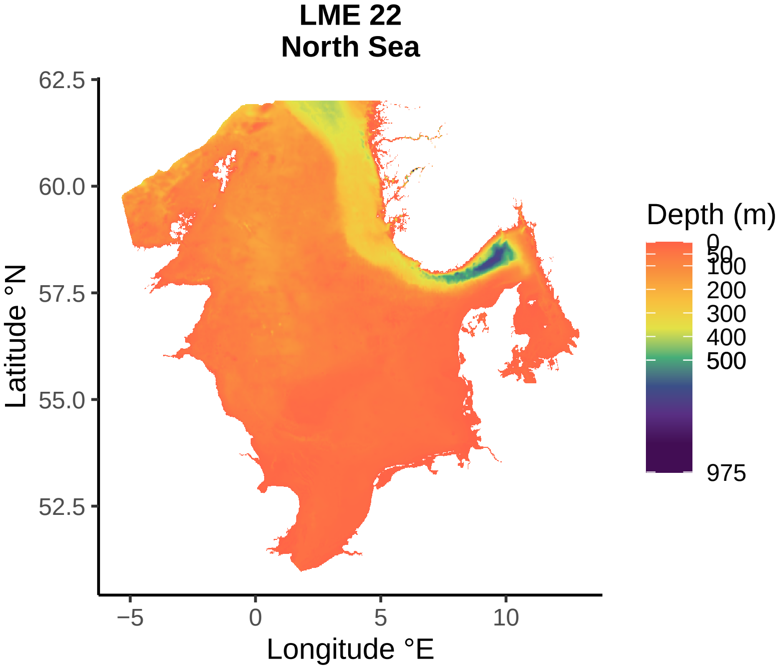
LME 22
North Sea
Latitude °N
62.5
60.0
57.5
55.0
52.5
−5
0
5
10
Longitude °E
Depth (m)
0
50
100
200
300
400
500
975

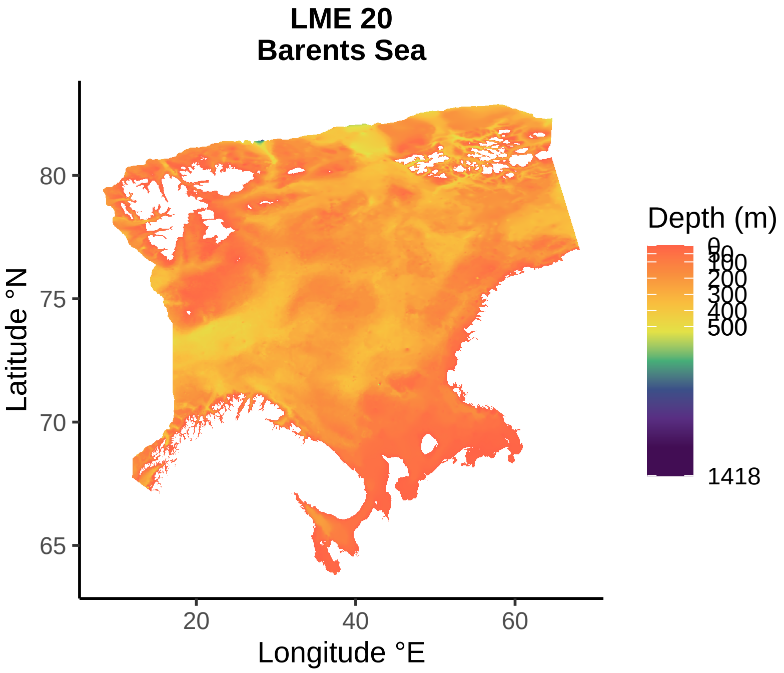
LME 20
Barents Sea
Latitude °N
80
75
70
65
20
40
60
Longitude °E
Depth (m)
0
50
100
200
300
400
500
1418

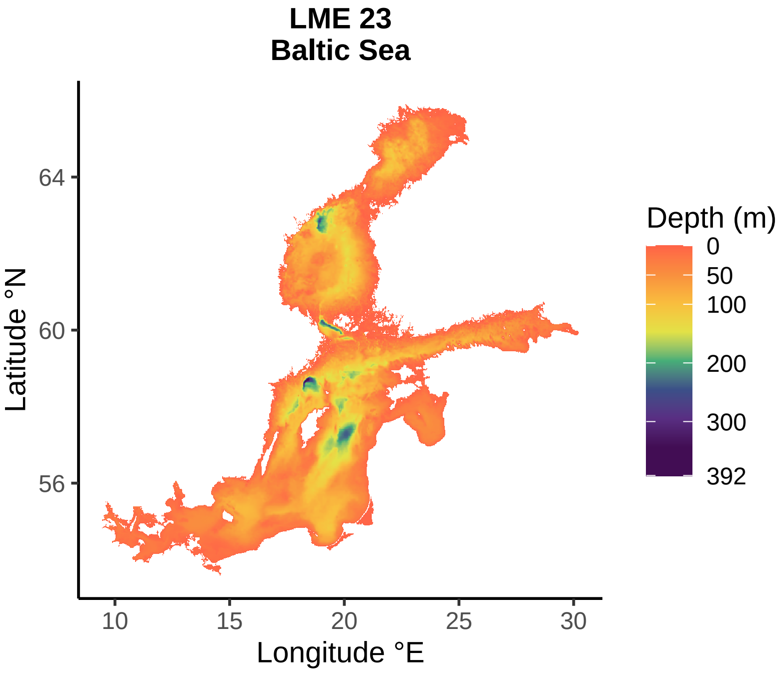
LME 23
Baltic Sea
Latitude °N
64
60
56
10
15
20
25
30
Longitude °E
Depth (m)
0
50
100
200
300
392

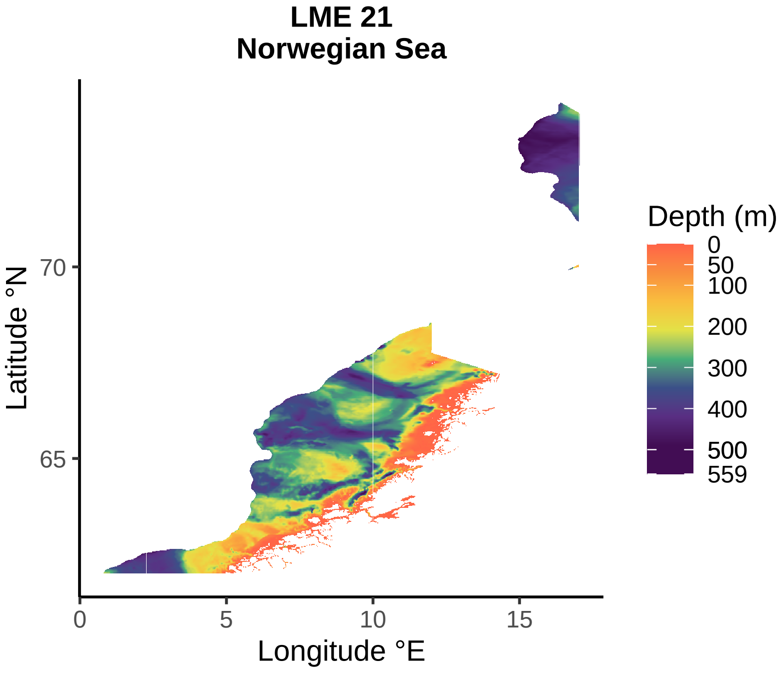
LME 21
Norwegian Sea
Latitude °N
70
65
0
5
10
15
Longitude °E
Depth (m)
0
50
100
200
300
400
500
559

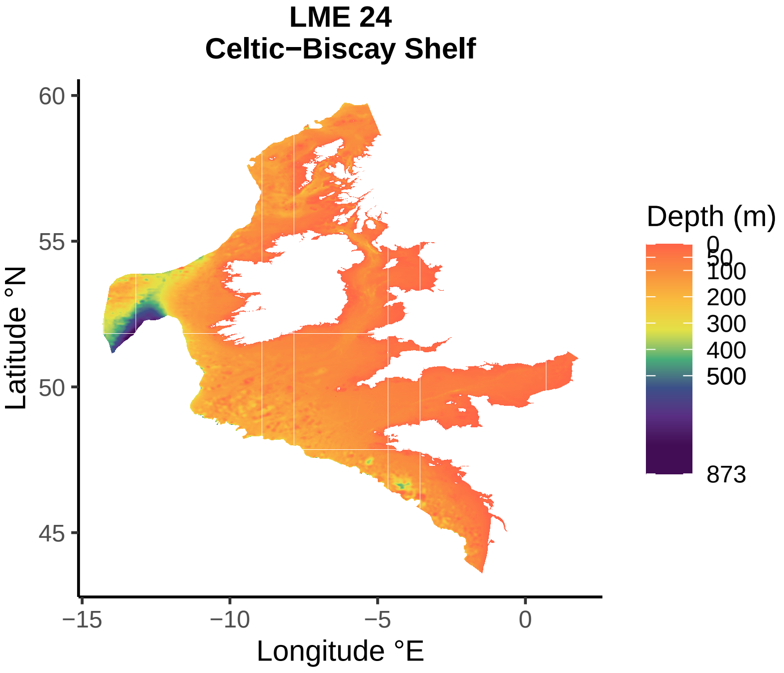
LME 24
Celtic−Biscay Shelf
Latitude °N
60
55
50
45
−15
−10
−5
0
Longitude °E
Depth (m)
0
50
100
200
300
400
500
873

LME 25
Iberian Coastal

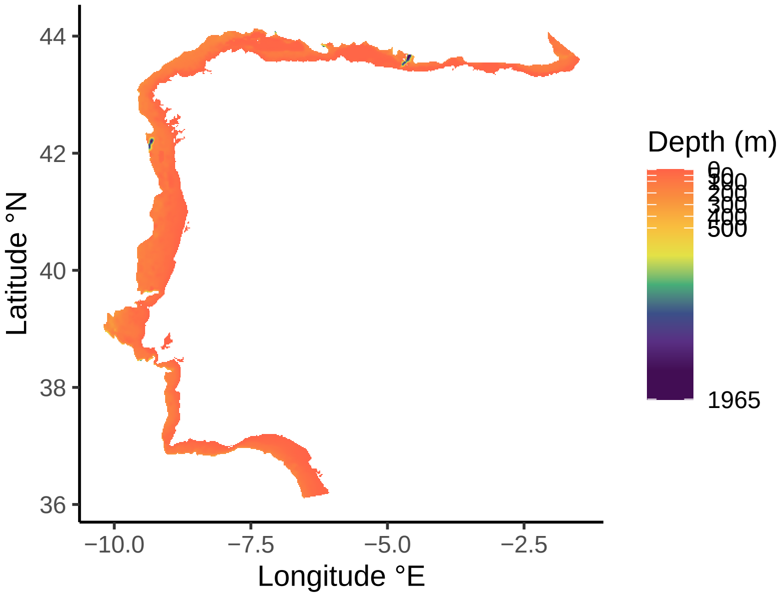

LME 28
Guinea Current

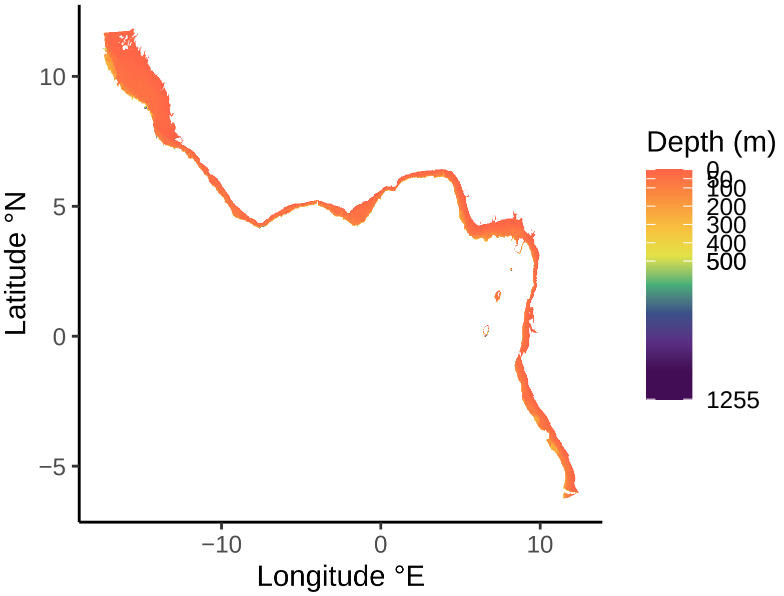

LME 26
Mediterranean Sea

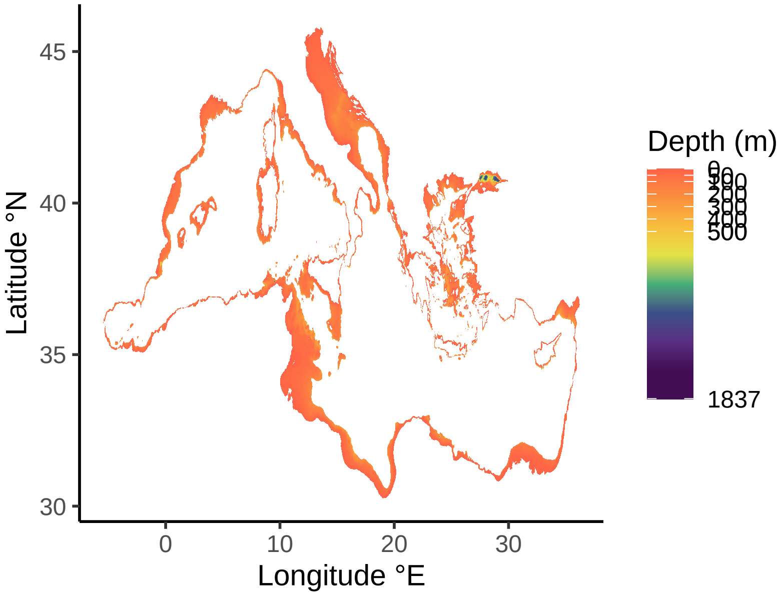

LME 29
Benguela Current

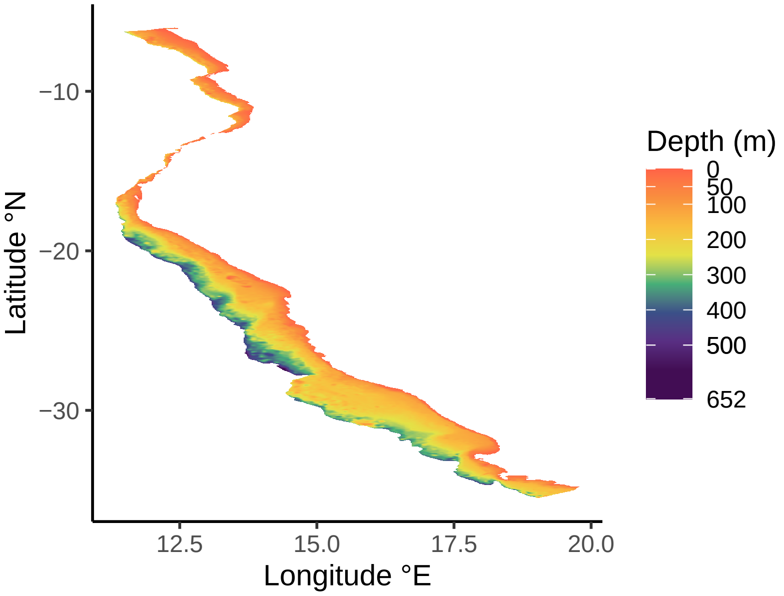

LME 27
Canary Current

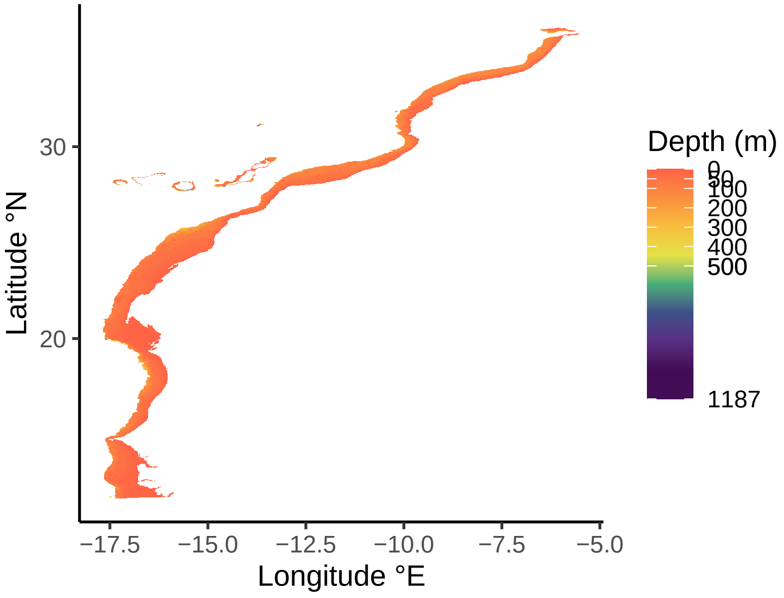

LME 30
Agulhas Current

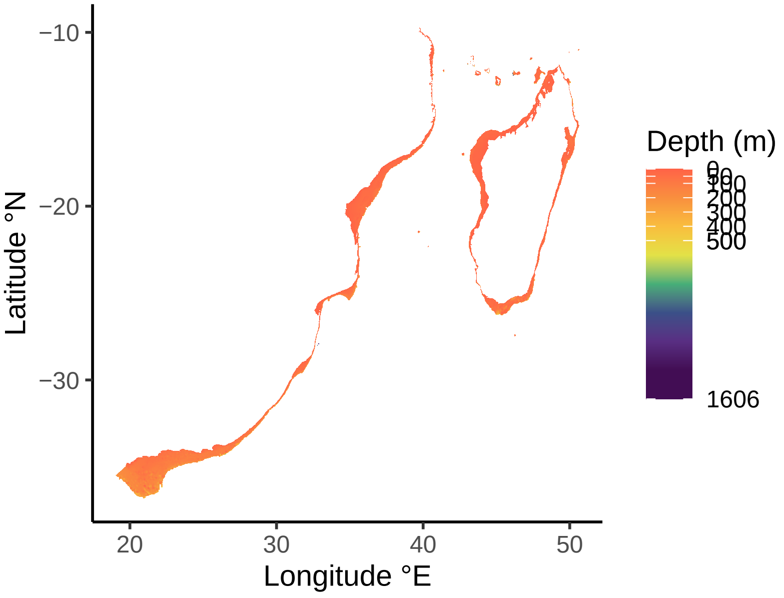

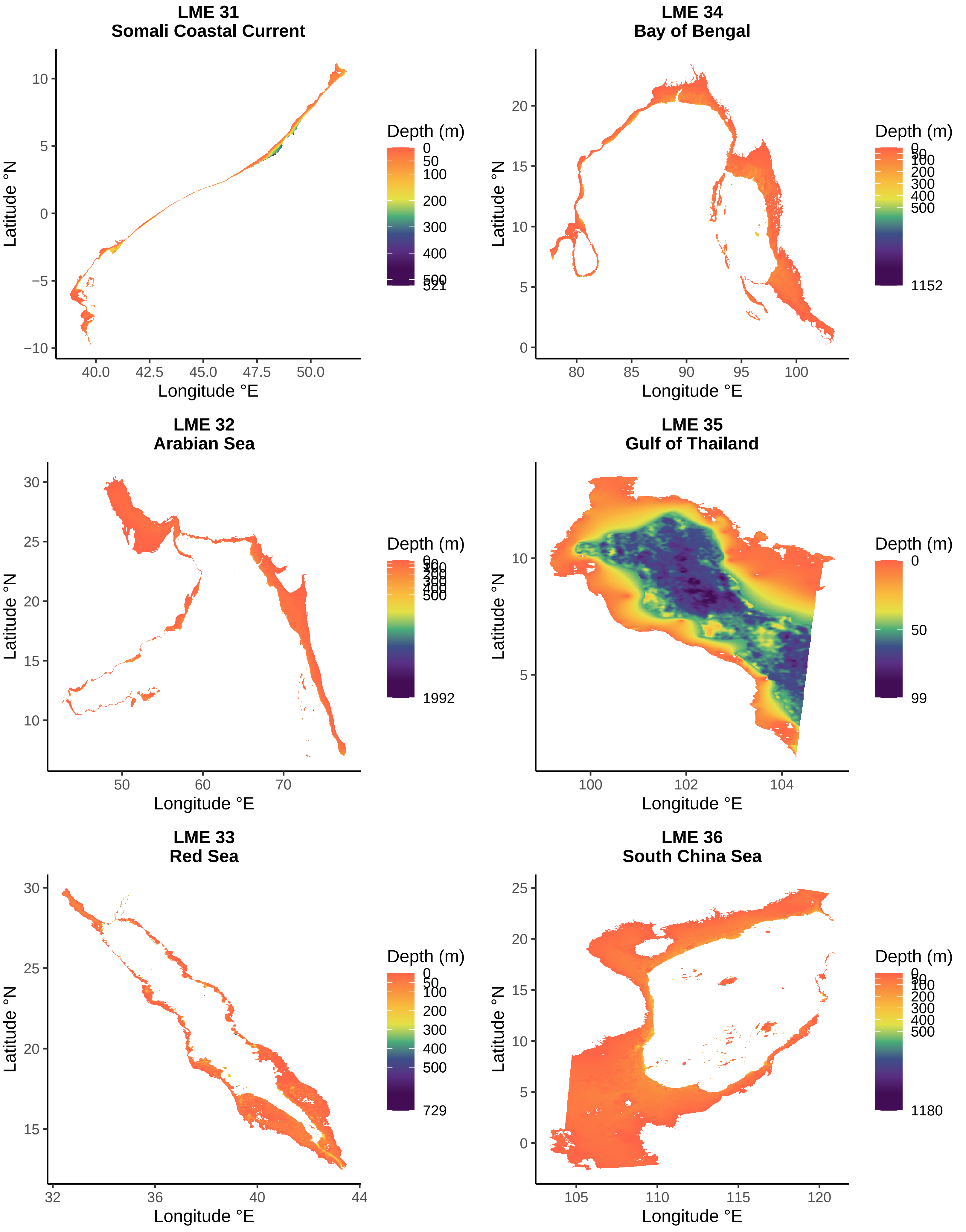
LME 31
Somali Coastal Current
Latitude °N
Longitude °E
Depth (m)
0
50
100
200
300
400
521
LME 34
Bay of Bengal
Latitude °N
Longitude °E
Depth (m)
100
200
300
400
500
1152
LME 32
Arabian Sea
Latitude °N
Longitude °E
Depth (m)
500
1992
LME 35
Gulf of Thailand
Latitude °N
Longitude °E
Depth (m)
0
50
99
LME 33
Red Sea
Latitude °N
Longitude °E
Depth (m)
100
200
300
400
500
729
LME 36
South China Sea
Latitude °N
Longitude °E
Depth (m)
100
200
300
400
500
1180

LME 37
Sulu−Celebes Sea

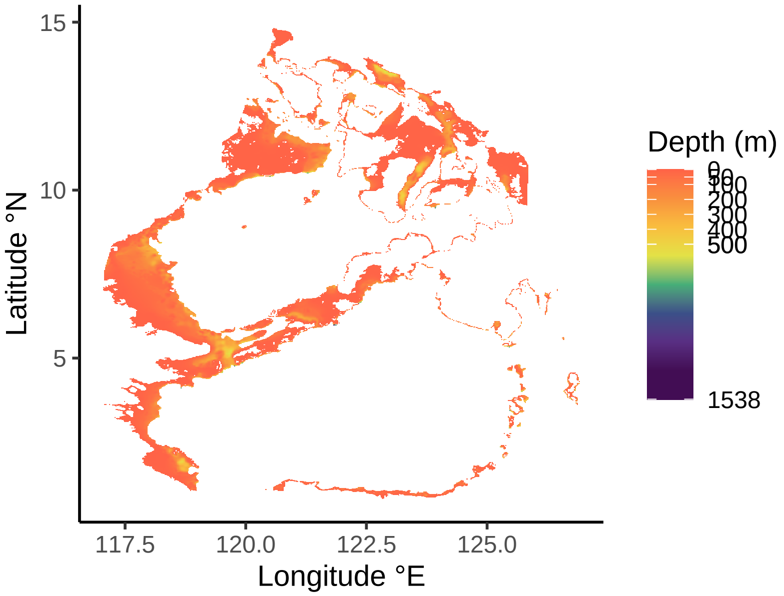

LME 40
Northeast Australian Shelf

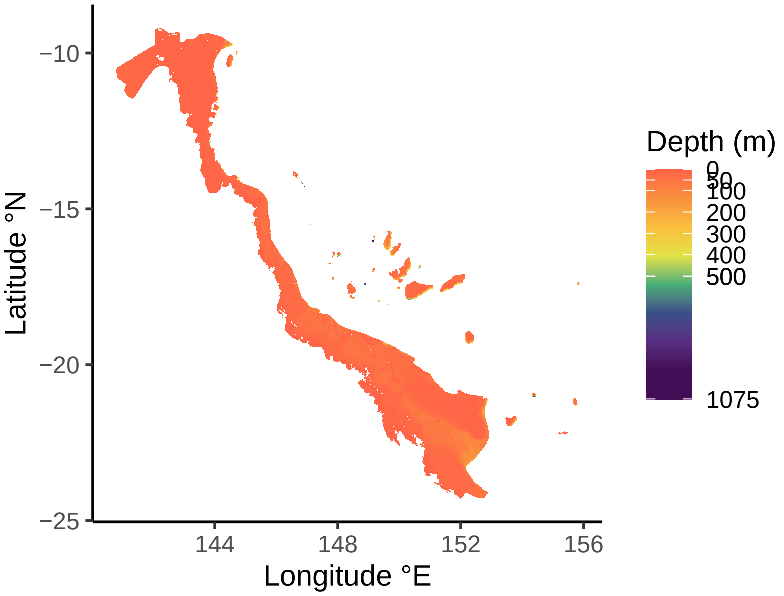

LME 38
Indonesian Sea

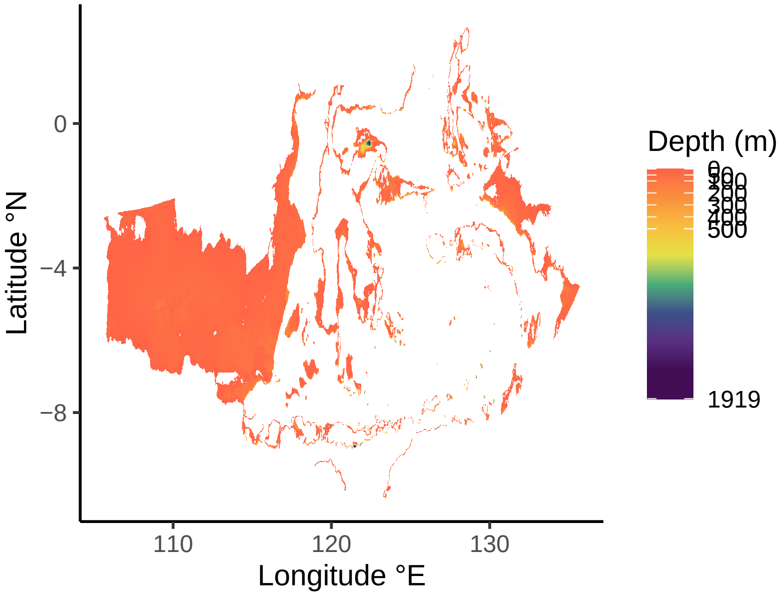

LME 41
East Central Australian Shelf

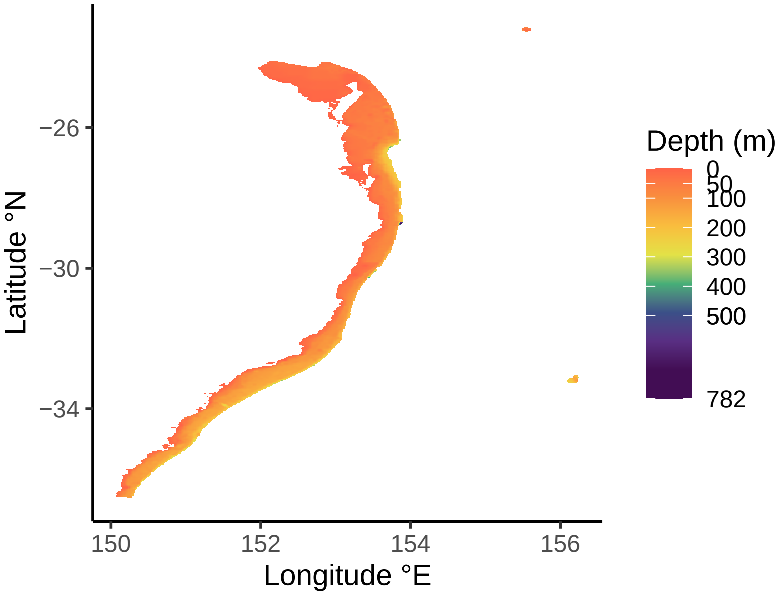

LME 39
North Australian Shelf

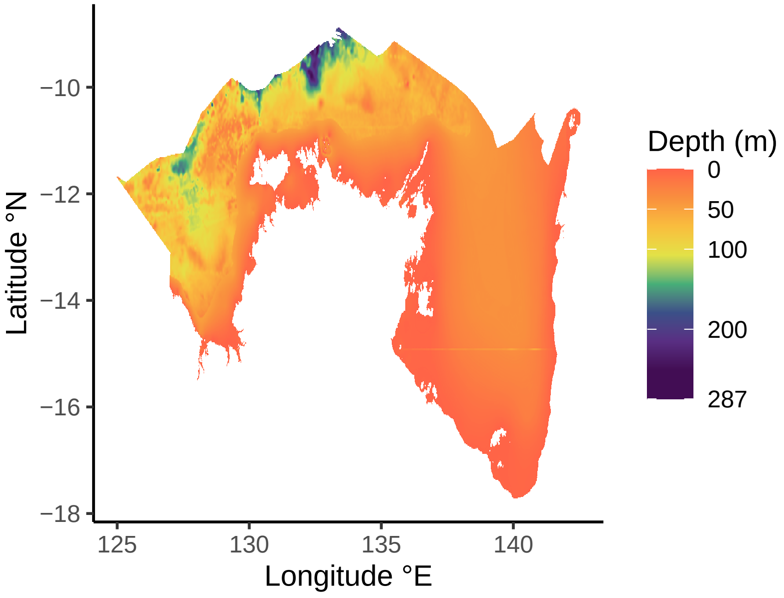

LME 42
Southeast Australian Shelf

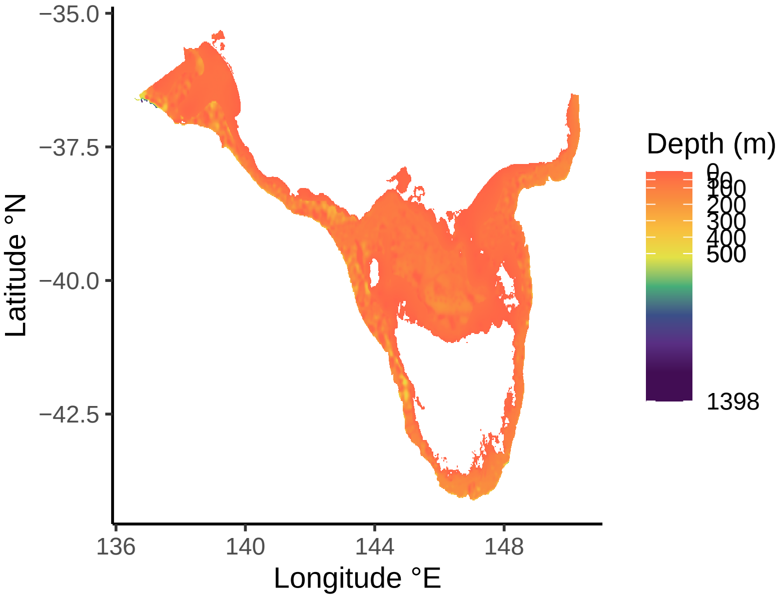

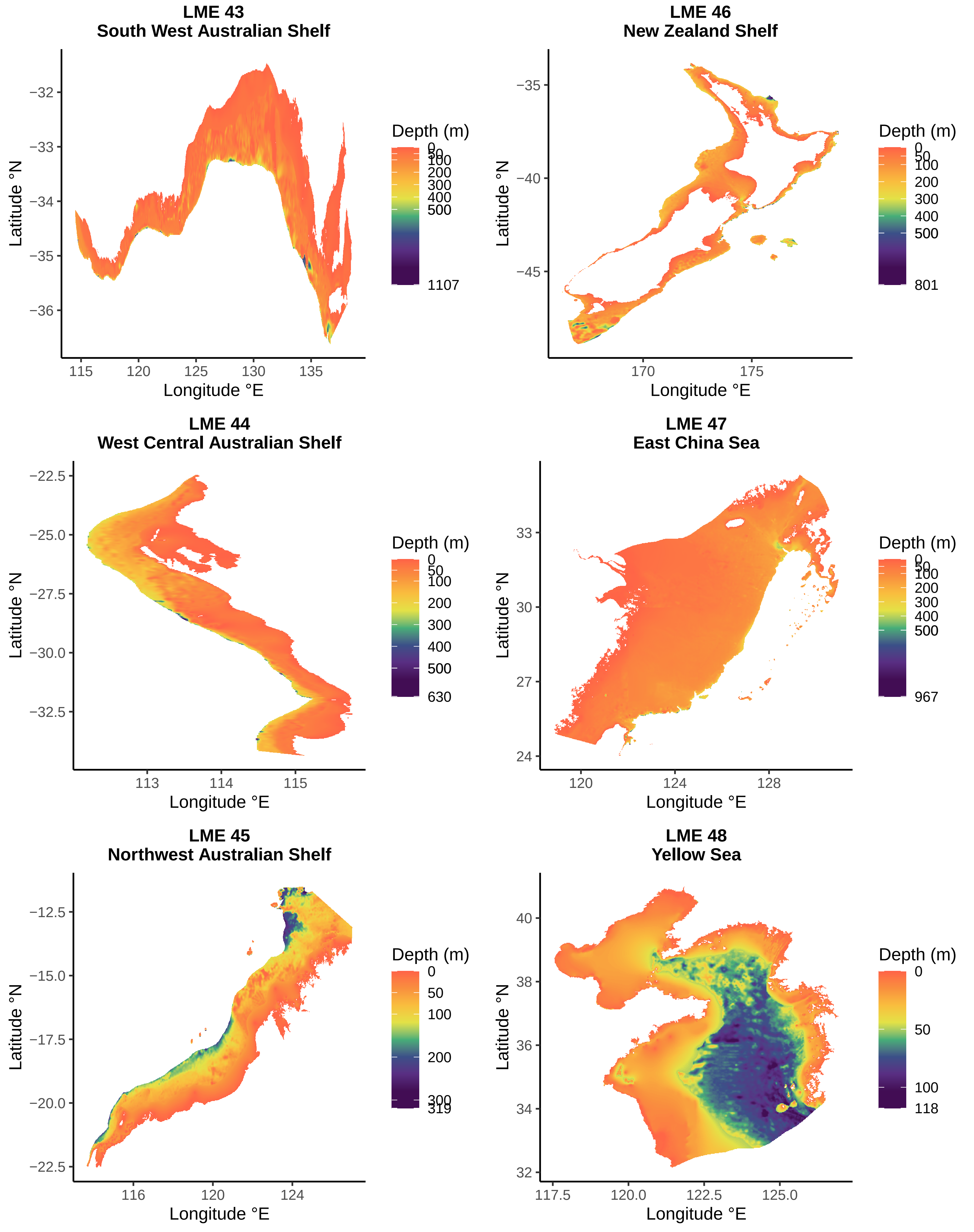

LME 43
South West Australian Shelf
Latitude °N
Longitude °E
Depth (m)
0
50
100
200
300
400
500
1107
LME 46
New Zealand Shelf
Latitude °N
Longitude °E
Depth (m)
0
50
100
200
300
400
500
801
LME 44
West Central Australian Shelf
Latitude °N
Longitude °E
Depth (m)
0
50
100
200
300
400
500
630
LME 47
East China Sea
Latitude °N
Longitude °E
Depth (m)
0
50
100
200
300
400
500
967
LME 45
Northwest Australian Shelf
Latitude °N
Longitude °E
Depth (m)
0
50
100
200
300
319
LME 48
Yellow Sea
Latitude °N
Longitude °E
Depth (m)
0
50
100
118

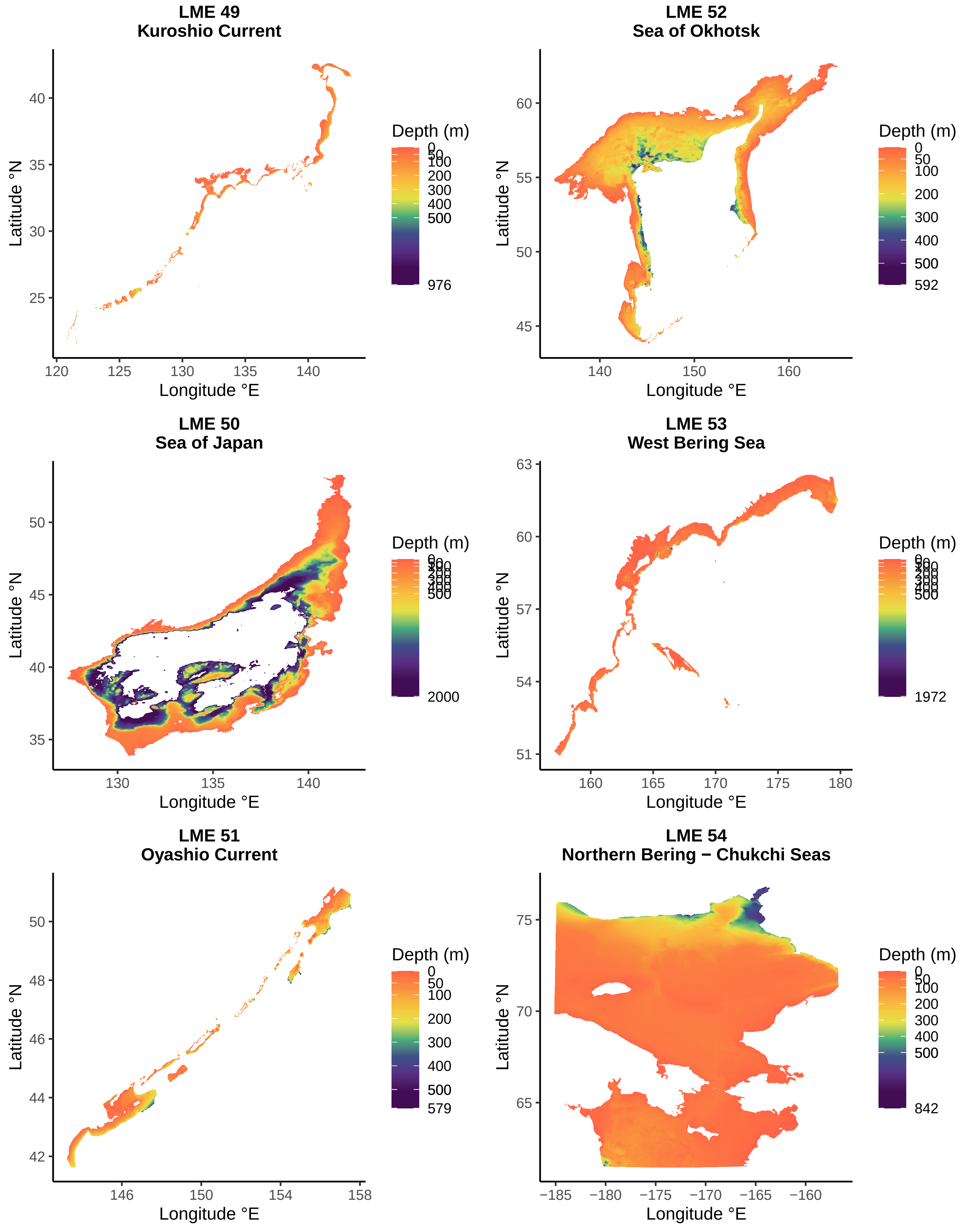
LME 49
Kuroshio Current
Latitude °N
Longitude °E
Depth (m)
0
50
100
200
300
400
500
976
LME 52
Sea of Okhotsk
Latitude °N
Longitude °E
Depth (m)
0
50
100
200
300
400
500
592
LME 50
Sea of Japan
Latitude °N
Longitude °E
Depth (m)
2000
LME 53
West Bering Sea
Latitude °N
Longitude °E
Depth (m)
1972
LME 51
Oyashio Current
Latitude °N
Longitude °E
Depth (m)
0
50
100
200
300
400
500
579
LME 54
Northern Bering − Chukchi Seas
Latitude °N
Longitude °E
Depth (m)
0
50
100
200
300
400
500
842

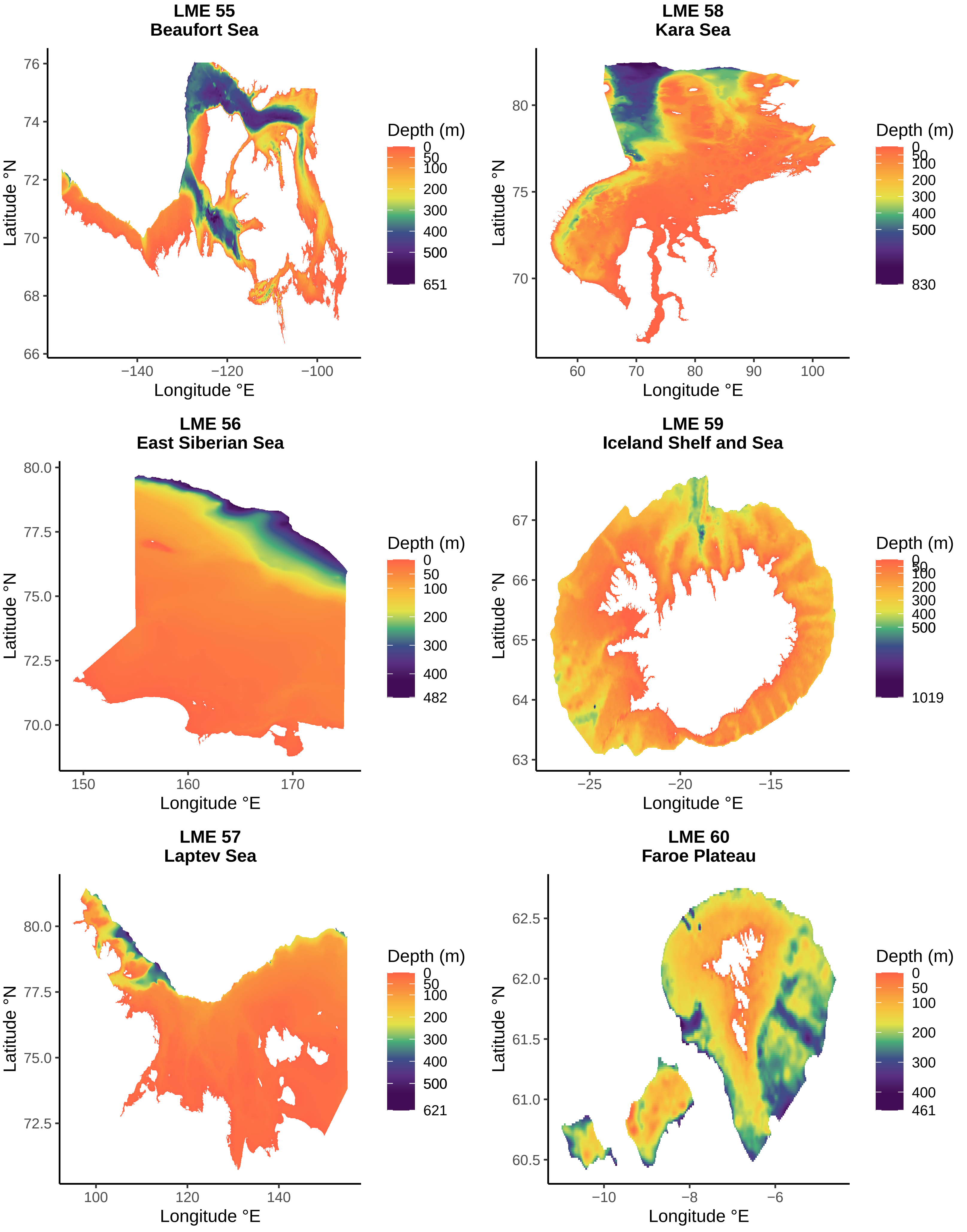

LME 55
Beaufort Sea
Depth (m)
0
50
100
200
300
400
500
651
Latitude °N
Longitude °E
76
74
72
70
68
66
−140
−120
−100
LME 58
Kara Sea
Depth (m)
0
50
100
200
300
400
500
830
Latitude °N
Longitude °E
80
75
70
60
70
80
90
100
LME 56
East Siberian Sea
Depth (m)
0
50
100
200
300
400
482
Latitude °N
Longitude °E
80.0
77.5
75.0
72.5
70.0
150
160
170
LME 59
Iceland Shelf and Sea
Depth (m)
0
50
100
200
300
400
500
1019
Latitude °N
Longitude °E
67
66
65
64
63
−25
−20
−15
LME 57
Laptev Sea
Depth (m)
0
50
100
200
300
400
500
621
Latitude °N
Longitude °E
80.0
77.5
75.0
72.5
100
120
140
LME 60
Faroe Plateau
Depth (m)
0
50
100
200
300
400
461
Latitude °N
Longitude °E
62.5
62.0
61.5
61.0
60.5
−10
−8
−6

LME 62
Black Sea

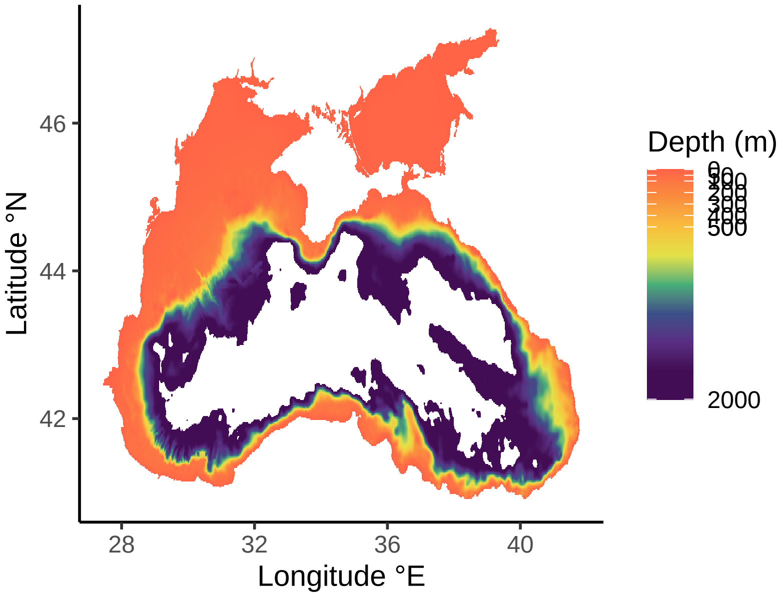

LME 66
Canadian High Arctic – North Greenland

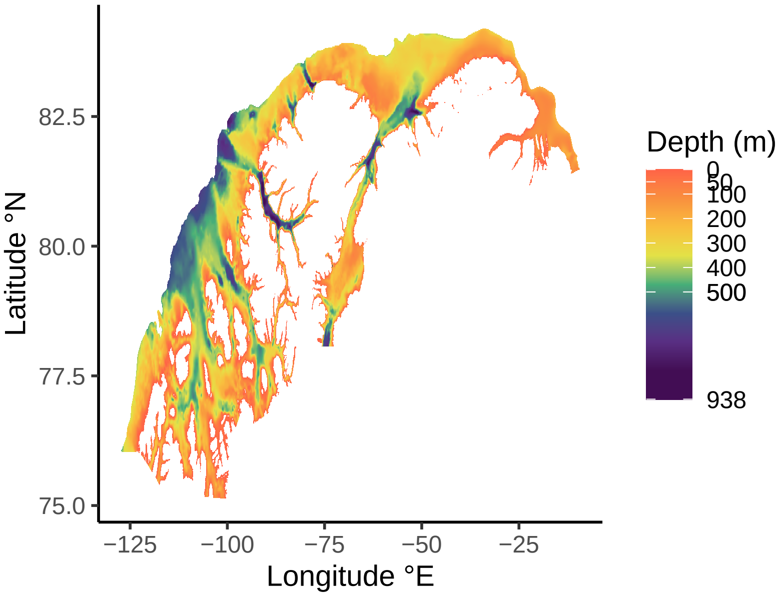

LME 63
Hudson Bay Complex

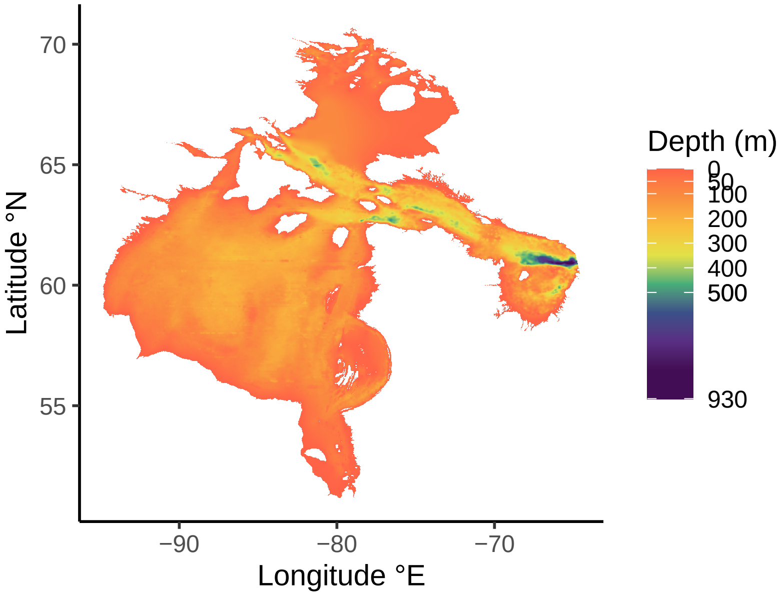

LME 65
Aleutian Islands

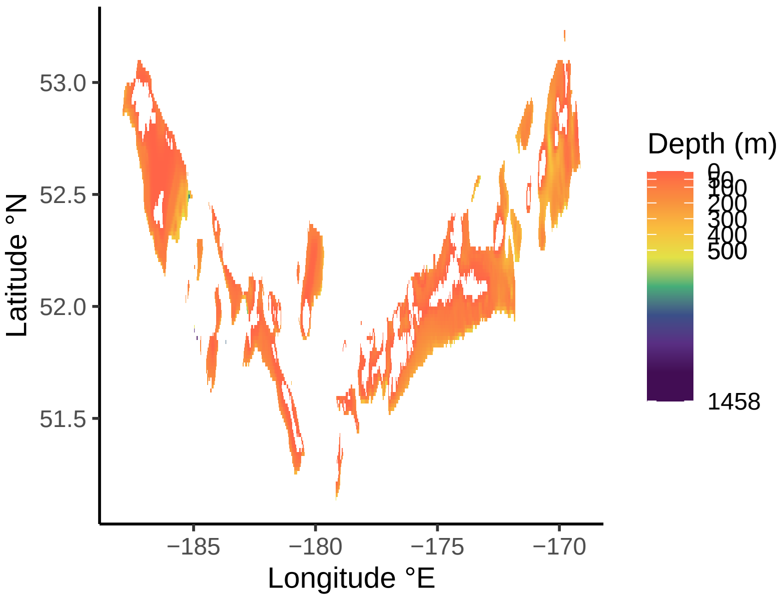

Figure S6. Hypsometric (area versus depth) curves for all 64 LMEs included in the analysis. Color represents depth ranging from orange (shallow) to purple (deep). The vertical red bar on the indicate mean depth for the LME.

Fig S6

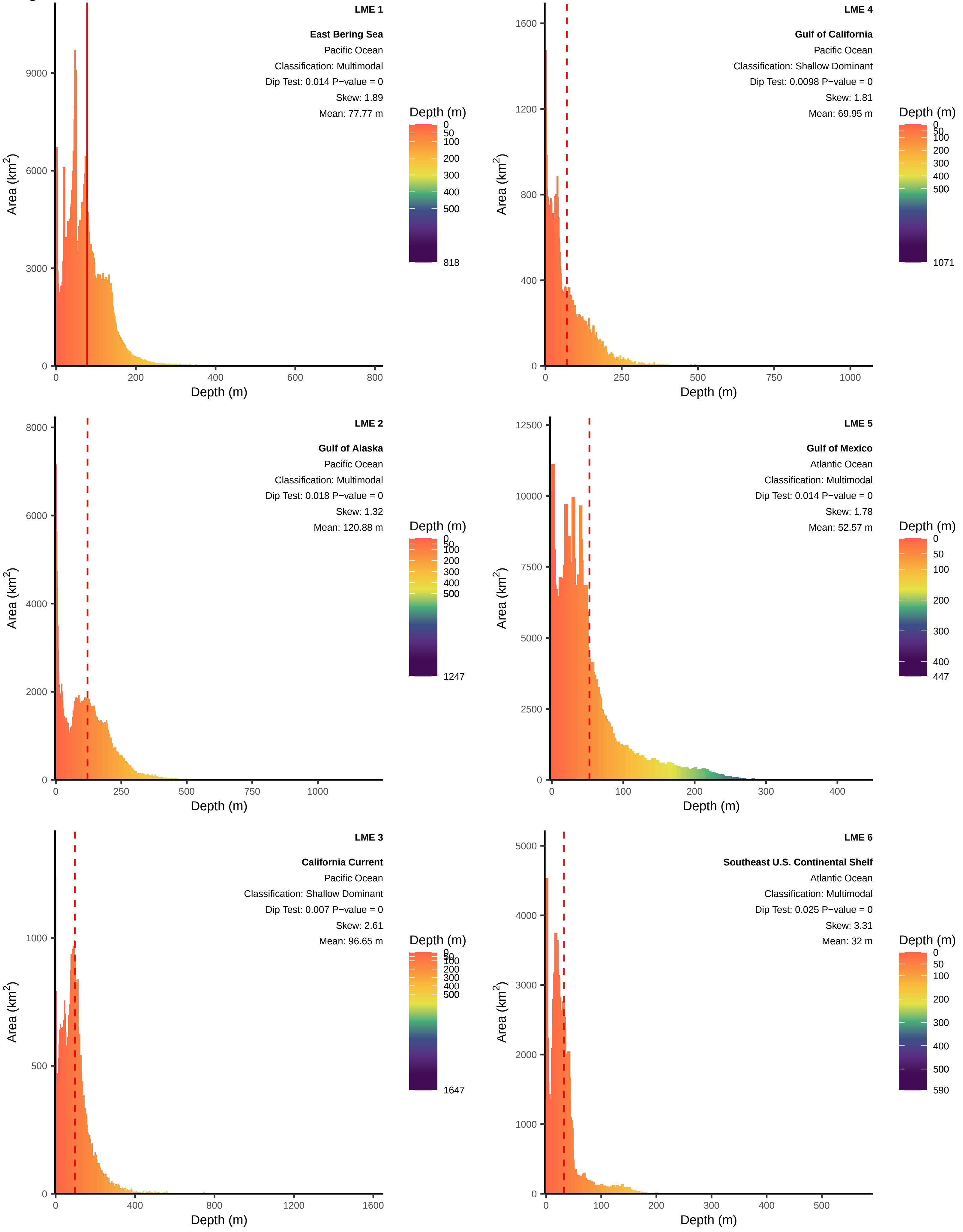
LME 1
East Bering Sea
Pacific Ocean
Classification: Multimodal
Dip Test: 0.014 P−value = 0
Skew: 1.89
Mean: 77.77 m
LME 4
Gulf of California
Pacific Ocean
Classification: Shallow Dominant
Dip Test: 0.0098 P−value = 0
Skew: 1.81
Mean: 69.95 m
LME 2
Gulf of Alaska
Pacific Ocean
Classification: Multimodal
Dip Test: 0.018 P−value = 0
Skew: 1.32
Mean: 120.88 m
LME 5
Gulf of Mexico
Atlantic Ocean
Classification: Multimodal
Dip Test: 0.014 P−value = 0
Skew: 1.78
Mean: 52.57 m
LME 3
California Current
Pacific Ocean
Classification: Shallow Dominant
Dip Test: 0.007 P−value = 0
Skew: 2.61
Mean: 96.65 m
LME 6
Southeast U.S. Continental Shelf
Atlantic Ocean
Classification: Multimodal
Dip Test: 0.025 P−value = 0
Skew: 3.31
Mean: 32 m
Depth (m)
Area (km2)

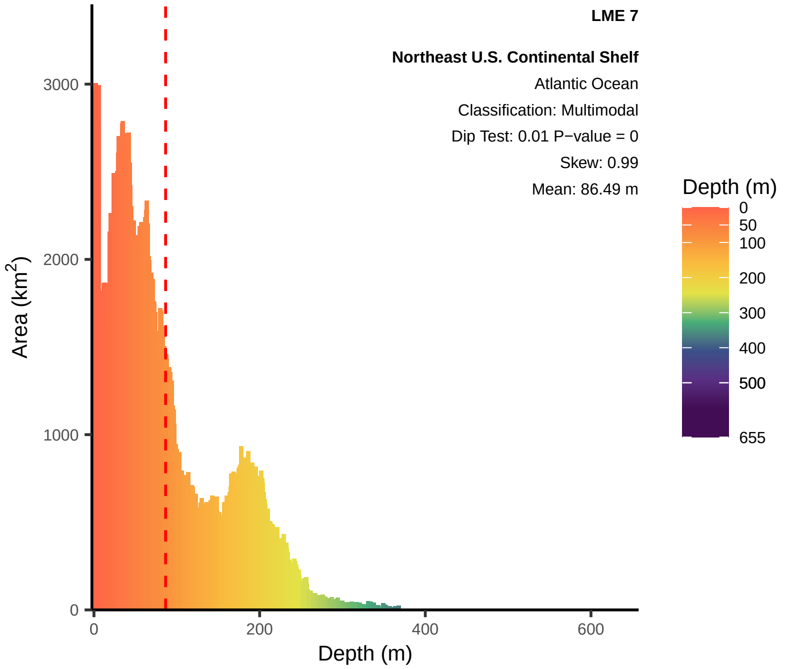
LME 7
Northeast U.S. Continental Shelf
Atlantic Ocean
Classification: Multimodal
Dip Test: 0.01 P−value = 0
Skew: 0.99
Mean: 86.49 m
Depth (m)
0
50
100
200
300
400
500
655
Area (km2)
Depth (m)

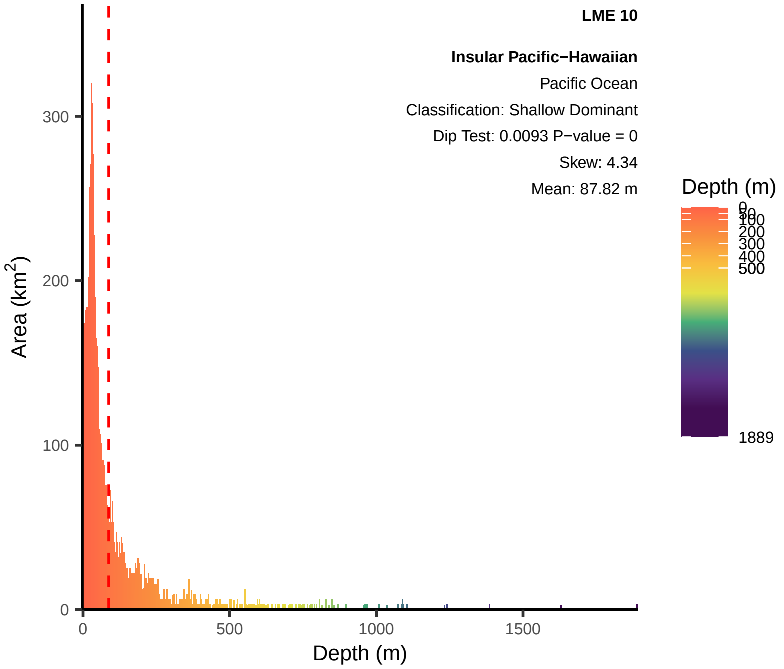
LME 10
Insular Pacific−Hawaiian
Pacific Ocean
Classification: Shallow Dominant
Dip Test: 0.0093 P−value = 0
Skew: 4.34
Mean: 87.82 m
Depth (m)
1889
Area (km2)
Depth (m)

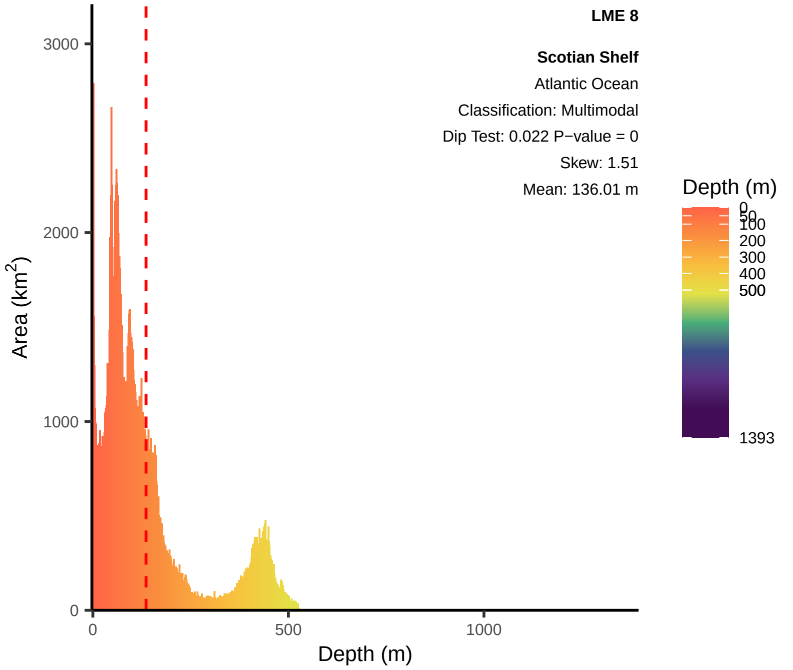
LME 8
Scotian Shelf
Atlantic Ocean
Classification: Multimodal
Dip Test: 0.022 P−value = 0
Skew: 1.51
Mean: 136.01 m
Depth (m)
1393
Area (km2)
Depth (m)

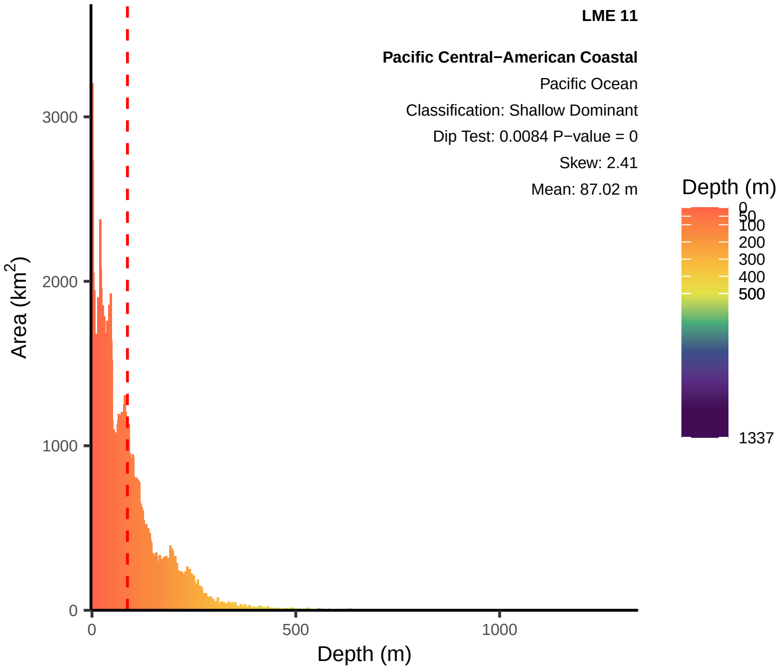
LME 11
Pacific Central−American Coastal
Pacific Ocean
Classification: Shallow Dominant
Dip Test: 0.0084 P−value = 0
Skew: 2.41
Mean: 87.02 m
Depth (m)
1337
Area (km2)
Depth (m)

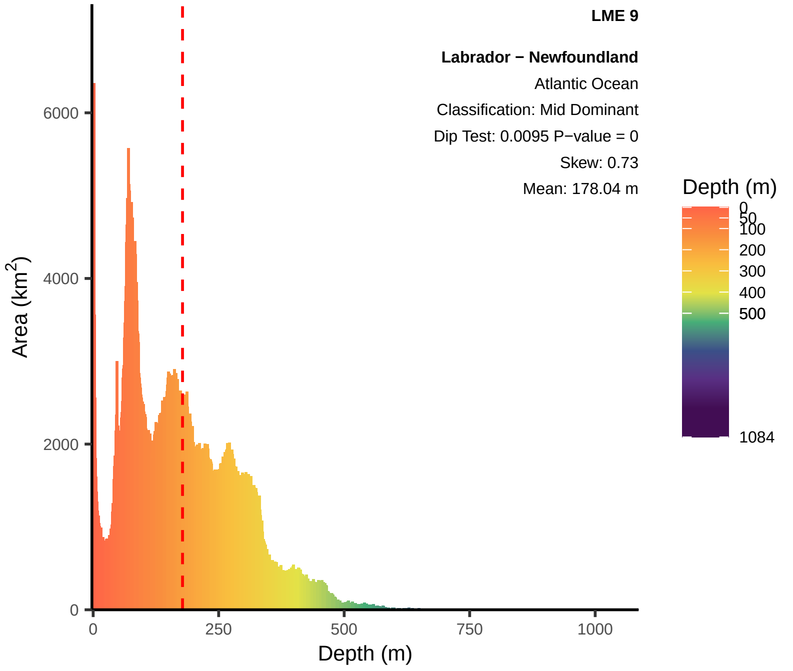
LME 9
Labrador − Newfoundland
Atlantic Ocean
Classification: Mid Dominant
Dip Test: 0.0095 P−value = 0
Skew: 0.73
Mean: 178.04 m
Depth (m)
1084
Area (km2)
Depth (m)

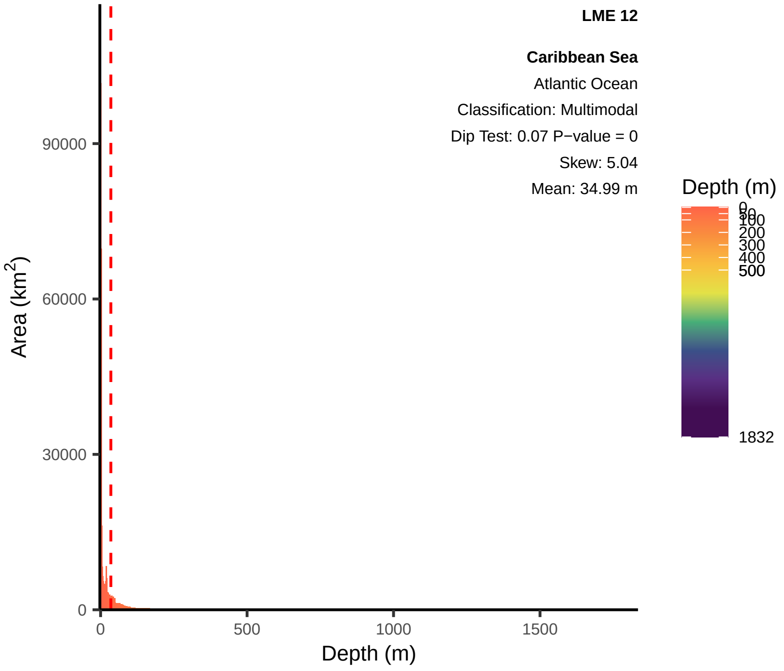
LME 12
Caribbean Sea
Atlantic Ocean
Classification: Multimodal
Dip Test: 0.07 P−value = 0
Skew: 5.04
Mean: 34.99 m
Depth (m)
1832
Area (km2)
Depth (m)

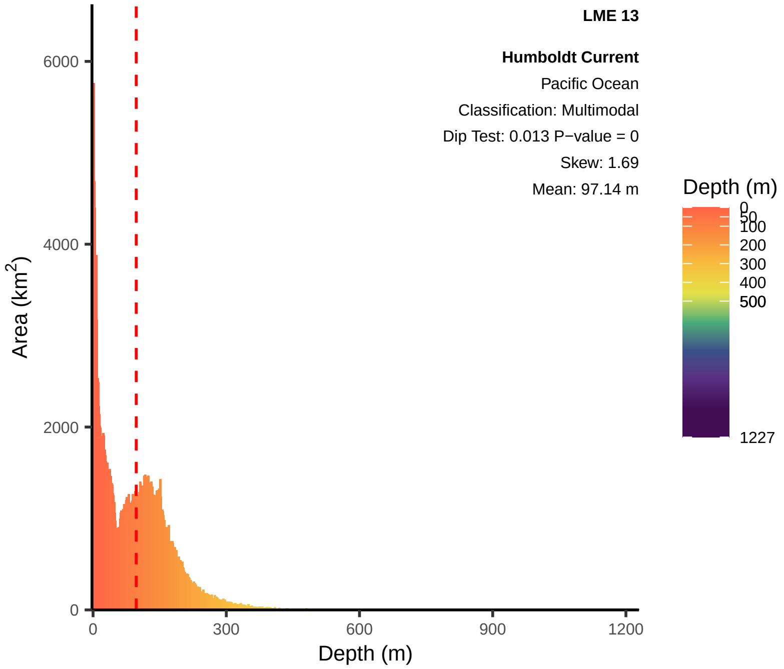
LME 13
Humboldt Current
Pacific Ocean
Classification: Multimodal
Dip Test: 0.013 P−value = 0
Skew: 1.69
Mean: 97.14 m
Area (km²)
Depth (m)
Depth (m)

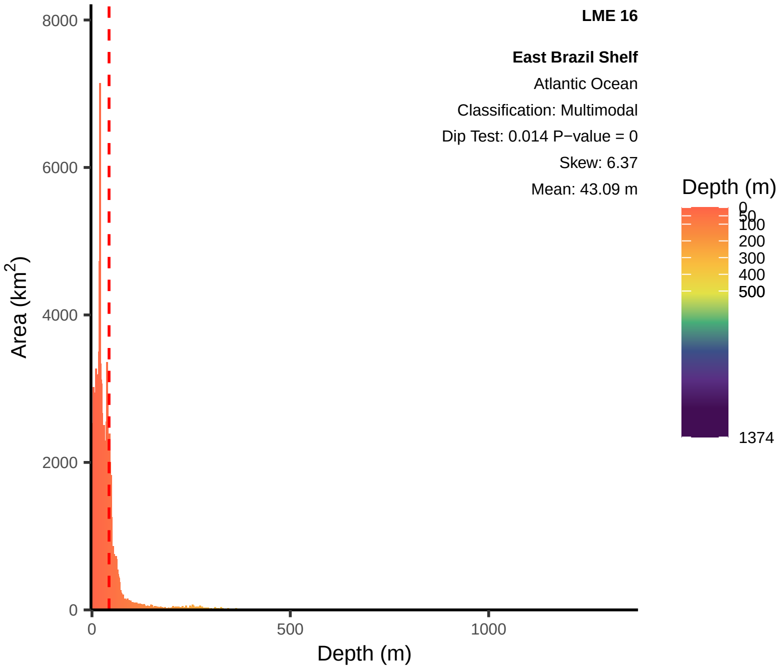
LME 16
East Brazil Shelf
Atlantic Ocean
Classification: Multimodal
Dip Test: 0.014 P−value = 0
Skew: 6.37
Mean: 43.09 m
Area (km²)
Depth (m)
Depth (m)

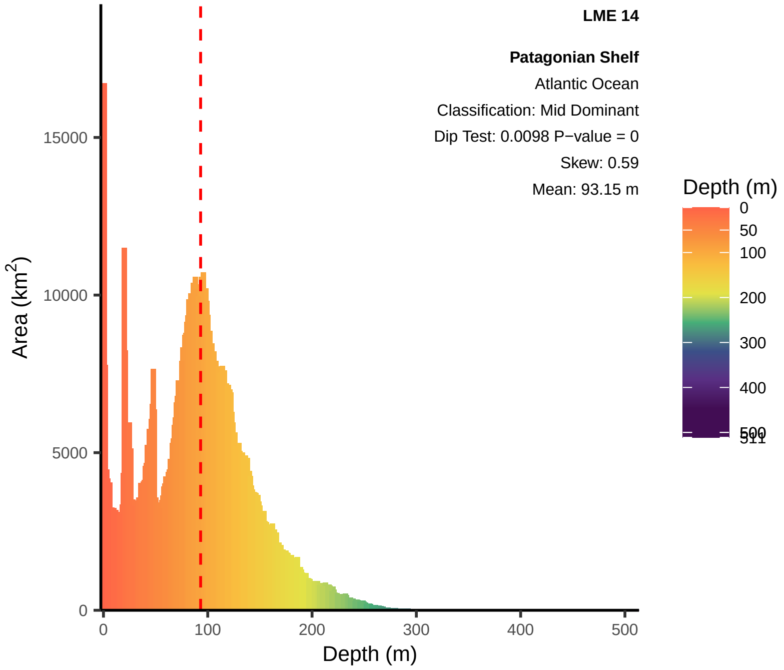
LME 14
Patagonian Shelf
Atlantic Ocean
Classification: Mid Dominant
Dip Test: 0.0098 P−value = 0
Skew: 0.59
Mean: 93.15 m
Area (km²)
Depth (m)
Depth (m)

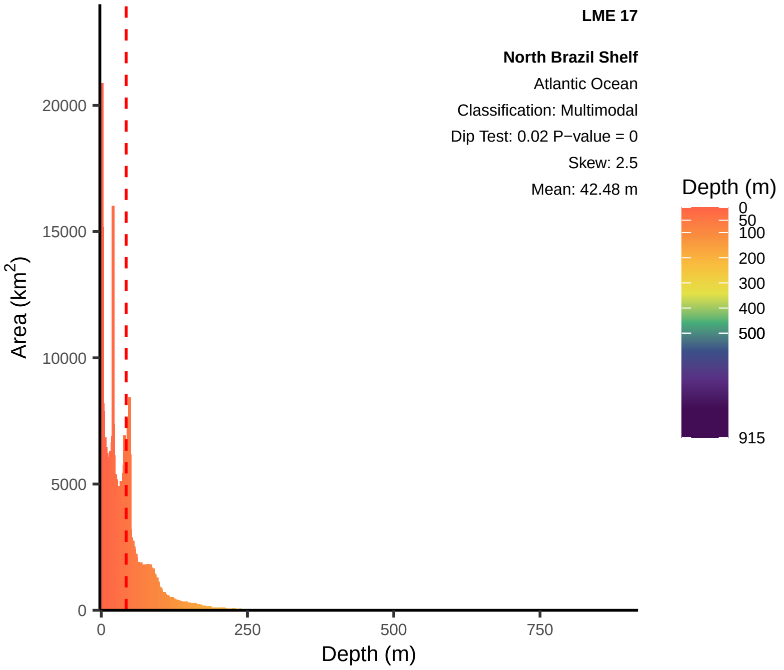
LME 17
North Brazil Shelf
Atlantic Ocean
Classification: Multimodal
Dip Test: 0.02 P−value = 0
Skew: 2.5
Mean: 42.48 m
Area (km²)
Depth (m)
Depth (m)

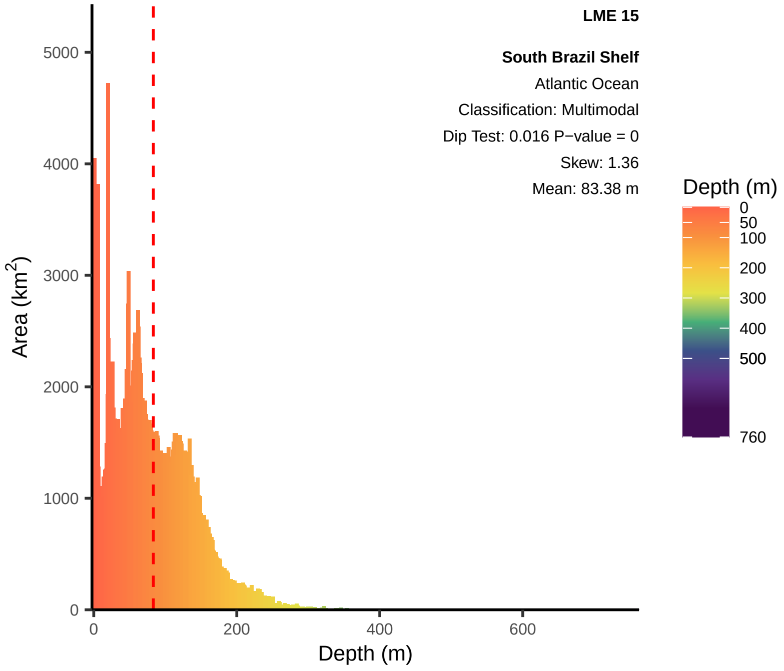
LME 15
South Brazil Shelf
Atlantic Ocean
Classification: Multimodal
Dip Test: 0.016 P−value = 0
Skew: 1.36
Mean: 83.38 m
Area (km²)
Depth (m)
Depth (m)

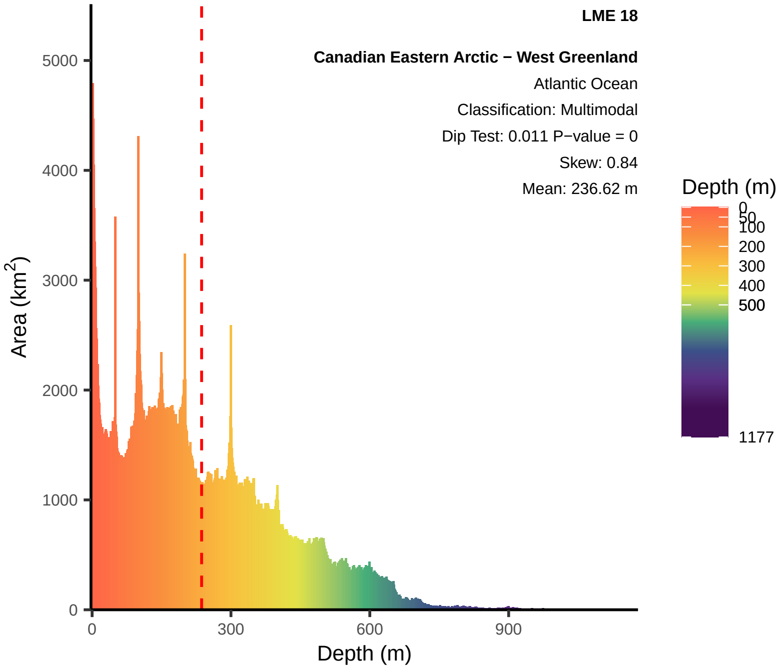
LME 18
Canadian Eastern Arctic − West Greenland
Atlantic Ocean
Classification: Multimodal
Dip Test: 0.011 P−value = 0
Skew: 0.84
Mean: 236.62 m
Area (km²)
Depth (m)
Depth (m)

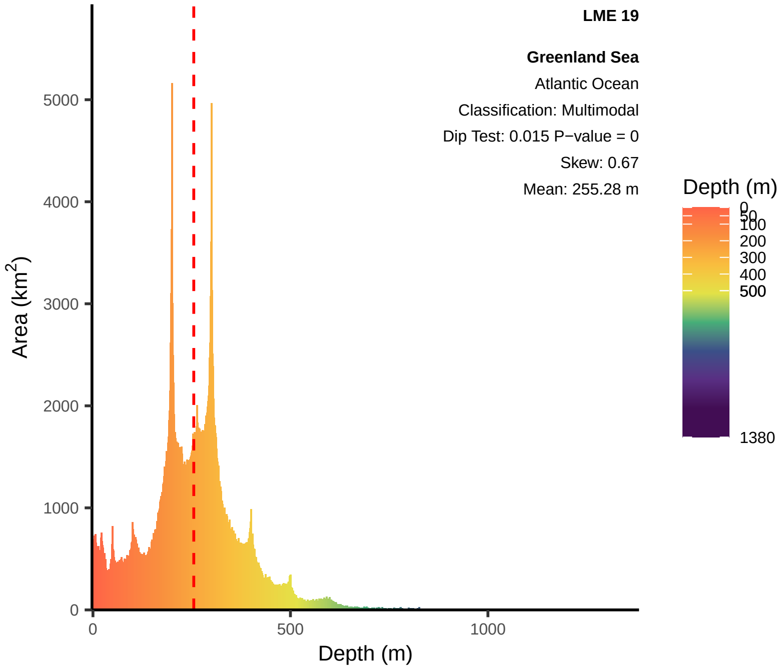
LME 19
Greenland Sea
Atlantic Ocean
Classification: Multimodal
Dip Test: 0.015 P−value = 0
Skew: 0.67
Mean: 255.28 m
Depth (m)
Area (km²)
Depth (m)
0
50
100
200
300
400
500
1380

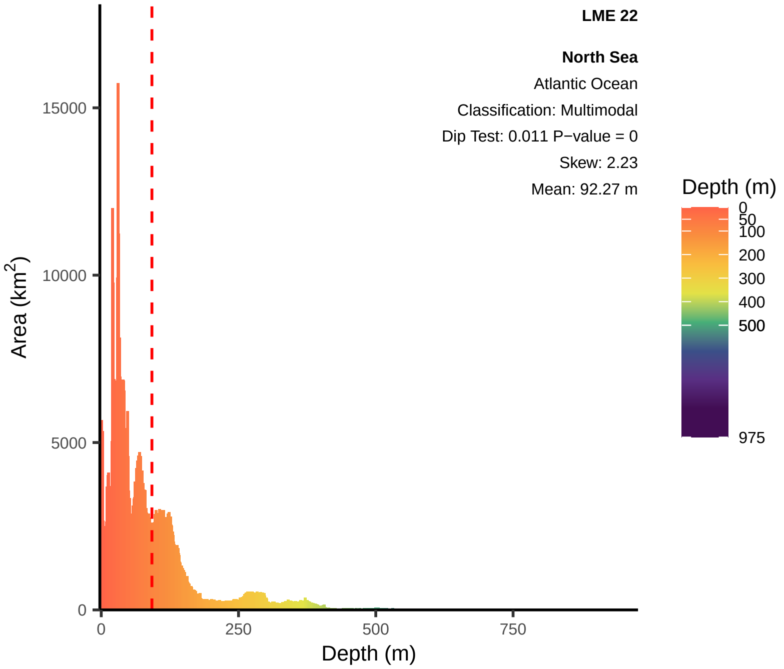
LME 22
North Sea
Atlantic Ocean
Classification: Multimodal
Dip Test: 0.011 P−value = 0
Skew: 2.23
Mean: 92.27 m
Depth (m)
Area (km²)
Depth (m)
0
50
100
200
300
400
500
975

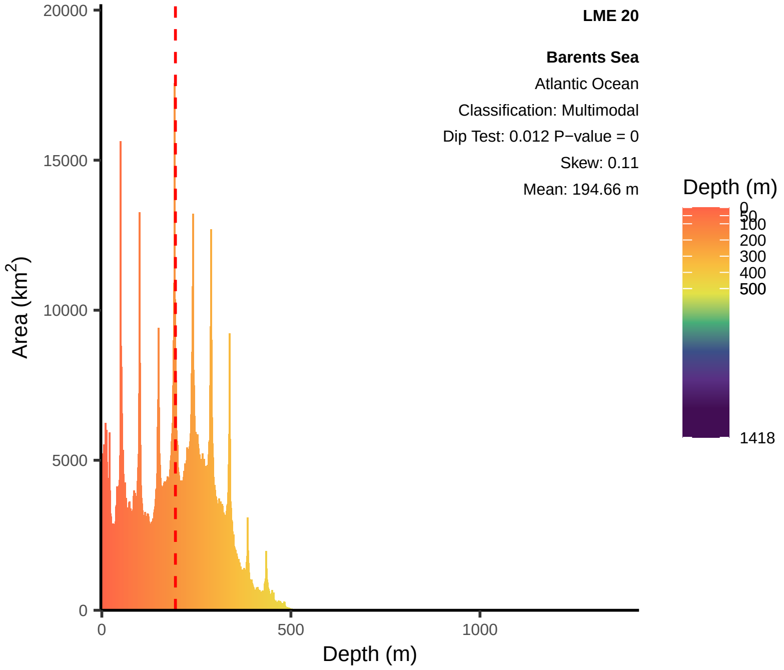
LME 20
Barents Sea
Atlantic Ocean
Classification: Multimodal
Dip Test: 0.012 P−value = 0
Skew: 0.11
Mean: 194.66 m
Depth (m)
Area (km²)
Depth (m)
0
50
100
200
300
400
500
1418

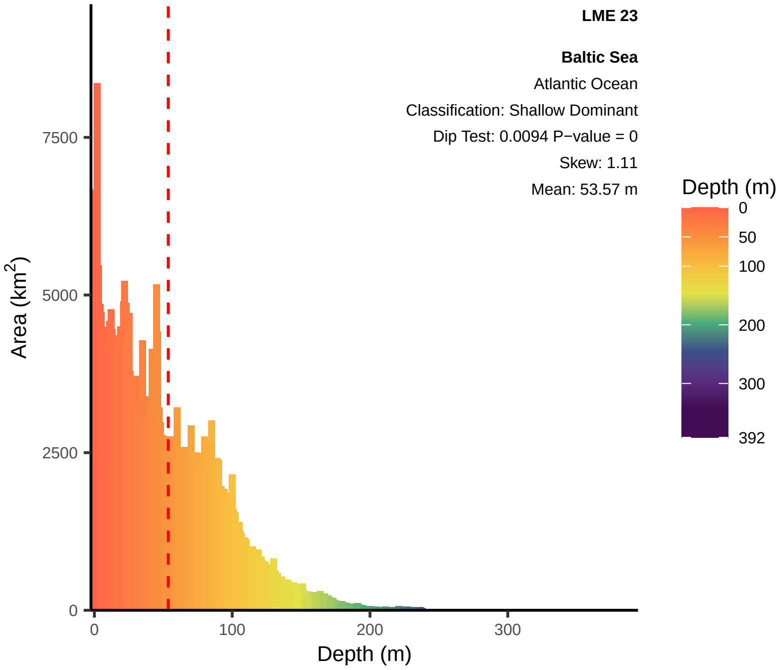
LME 23
Baltic Sea
Atlantic Ocean
Classification: Shallow Dominant
Dip Test: 0.0094 P−value = 0
Skew: 1.11
Mean: 53.57 m
Depth (m)
Area (km²)
Depth (m)
0
50
100
200
300
392

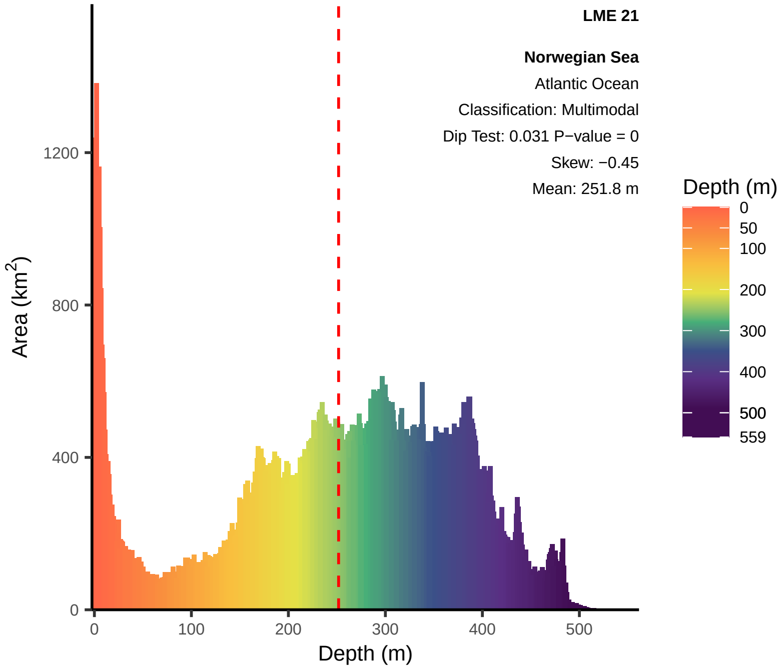
LME 21
Norwegian Sea
Atlantic Ocean
Classification: Multimodal
Dip Test: 0.031 P−value = 0
Skew: −0.45
Mean: 251.8 m
Depth (m)
Area (km²)
Depth (m)
0
50
100
200
300
400
500
559

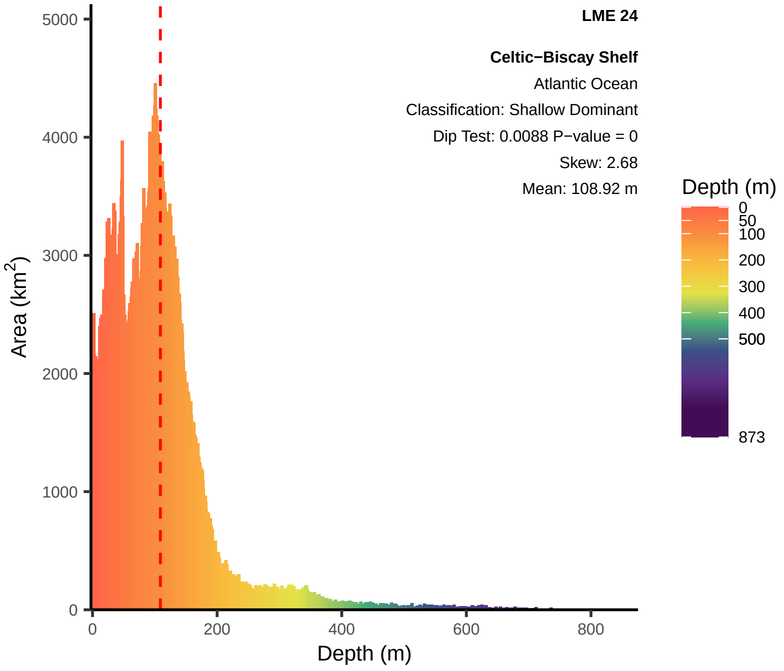
LME 24
Celtic−Biscay Shelf
Atlantic Ocean
Classification: Shallow Dominant
Dip Test: 0.0088 P−value = 0
Skew: 2.68
Mean: 108.92 m
Depth (m)
Area (km²)
Depth (m)
0
50
100
200
300
400
500
873

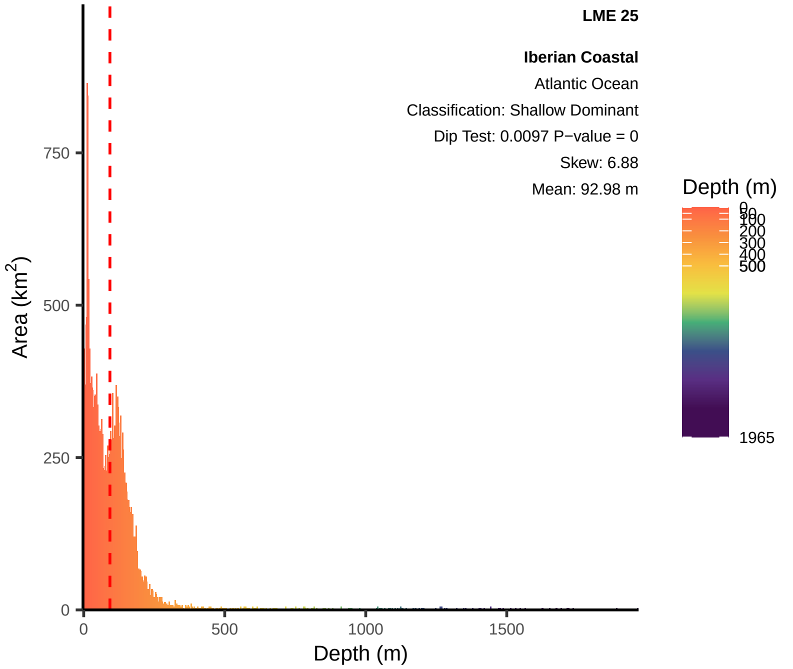
LME 25
Iberian Coastal
Atlantic Ocean
Classification: Shallow Dominant
Dip Test: 0.0097 P−value = 0
Skew: 6.88
Mean: 92.98 m
Depth (m)
1965
Area (km²)
Depth (m)
0
500
1000
1500
250
750

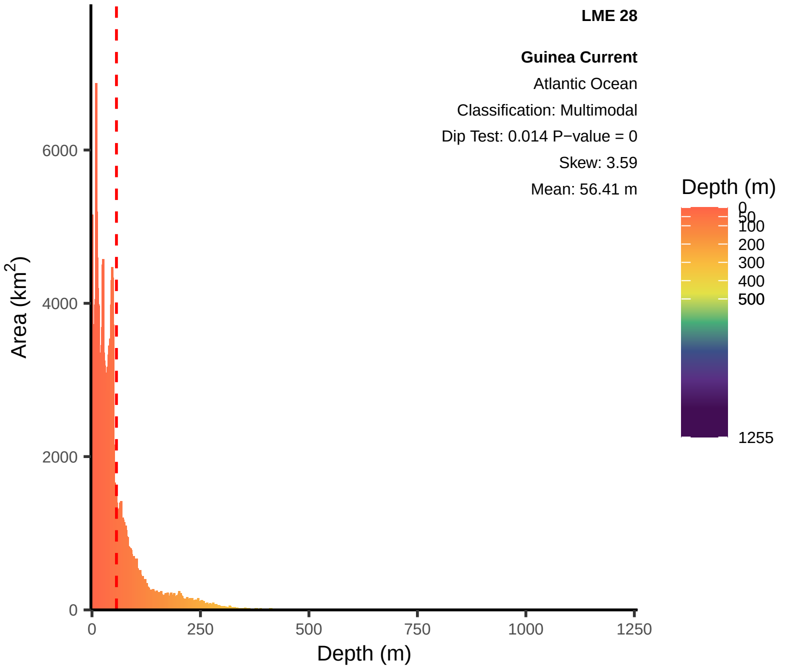
LME 28
Guinea Current
Atlantic Ocean
Classification: Multimodal
Dip Test: 0.014 P−value = 0
Skew: 3.59
Mean: 56.41 m
Depth (m)
100
200
300
400
500
1255
Area (km²)
Depth (m)
0
250
500
750
1000
1250
2000
4000
6000

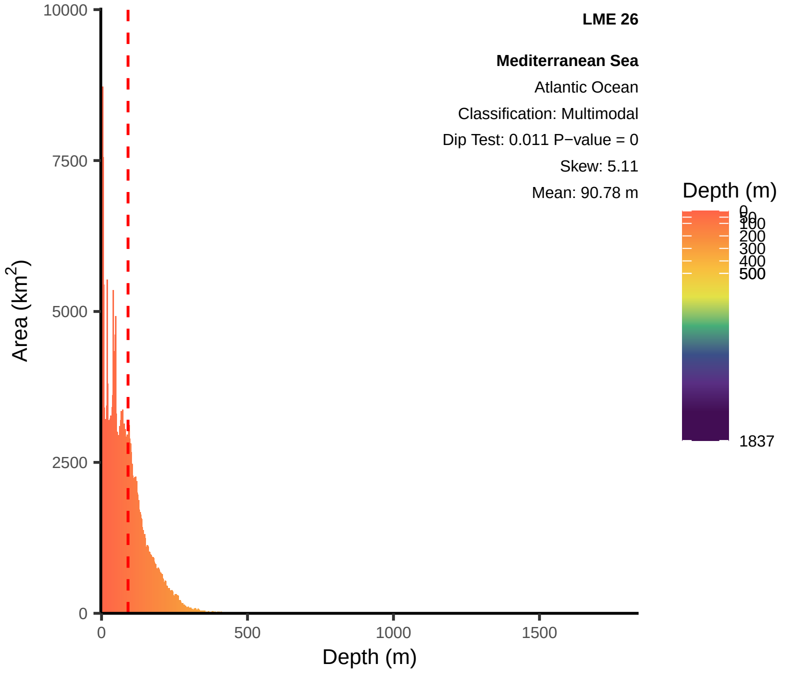
LME 26
Mediterranean Sea
Atlantic Ocean
Classification: Multimodal
Dip Test: 0.011 P−value = 0
Skew: 5.11
Mean: 90.78 m
Depth (m)
1837
Area (km²)
Depth (m)
0
500
1000
1500
2500
5000
7500
10000

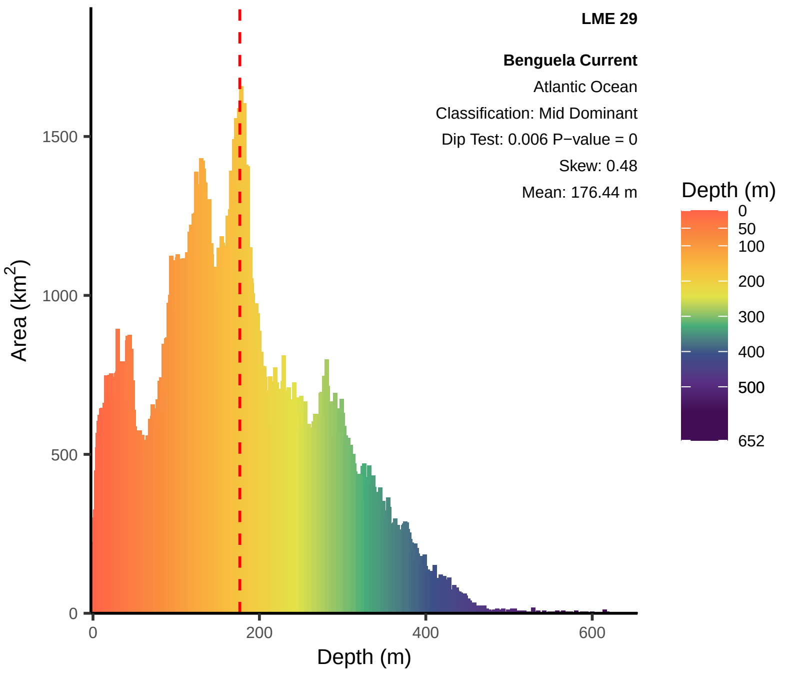
LME 29
Benguela Current
Atlantic Ocean
Classification: Mid Dominant
Dip Test: 0.006 P−value = 0
Skew: 0.48
Mean: 176.44 m
Depth (m)
0
50
100
200
300
400
500
652
Area (km²)
Depth (m)
0
200
400
600
500
1000
1500

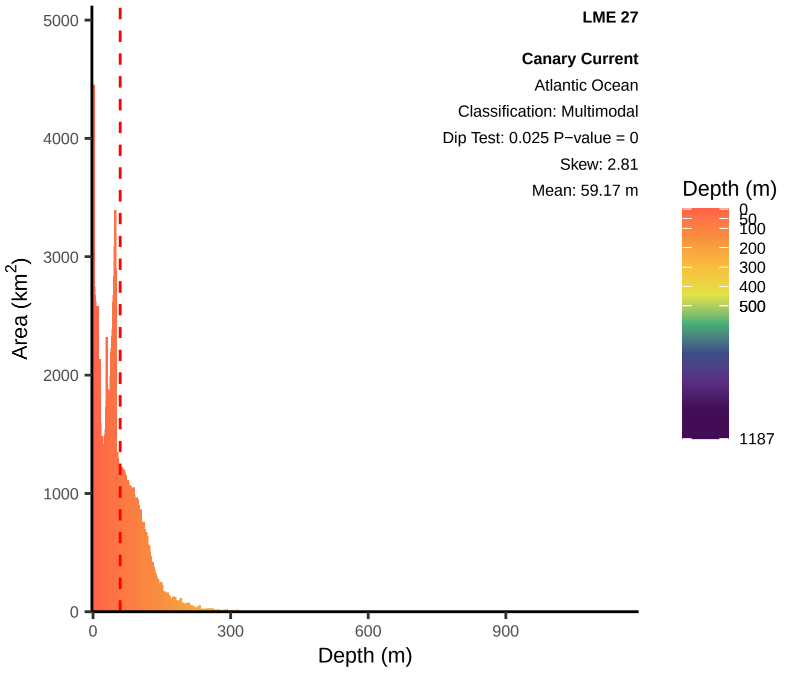
LME 27
Canary Current
Atlantic Ocean
Classification: Multimodal
Dip Test: 0.025 P−value = 0
Skew: 2.81
Mean: 59.17 m
Depth (m)
100
200
300
400
500
1187
Area (km²)
Depth (m)
0
300
600
900
1000
2000
3000
4000
5000

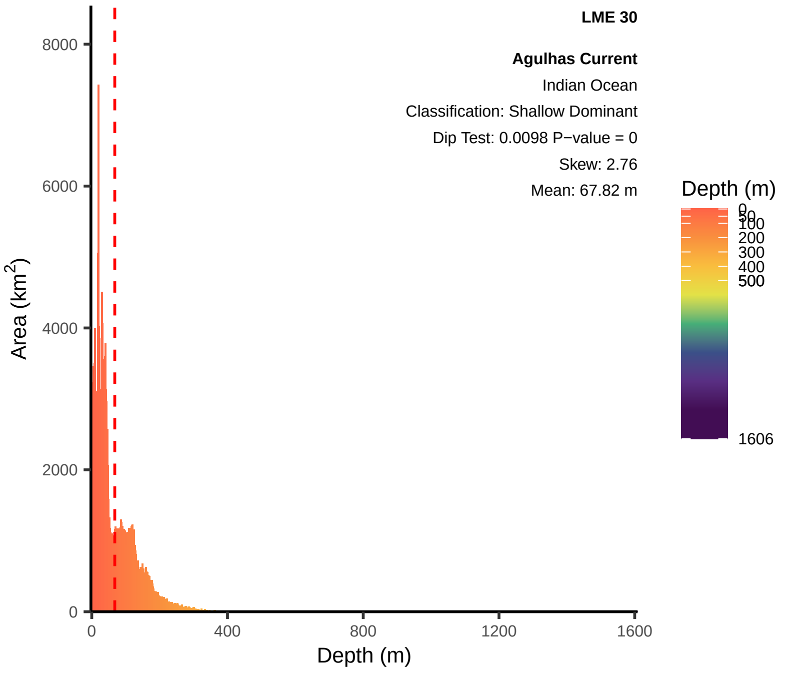
LME 30
Agulhas Current
Indian Ocean
Classification: Shallow Dominant
Dip Test: 0.0098 P−value = 0
Skew: 2.76
Mean: 67.82 m
Depth (m)
1606
Area (km²)
Depth (m)
0
400
800
1200
1600
2000
4000
6000
8000

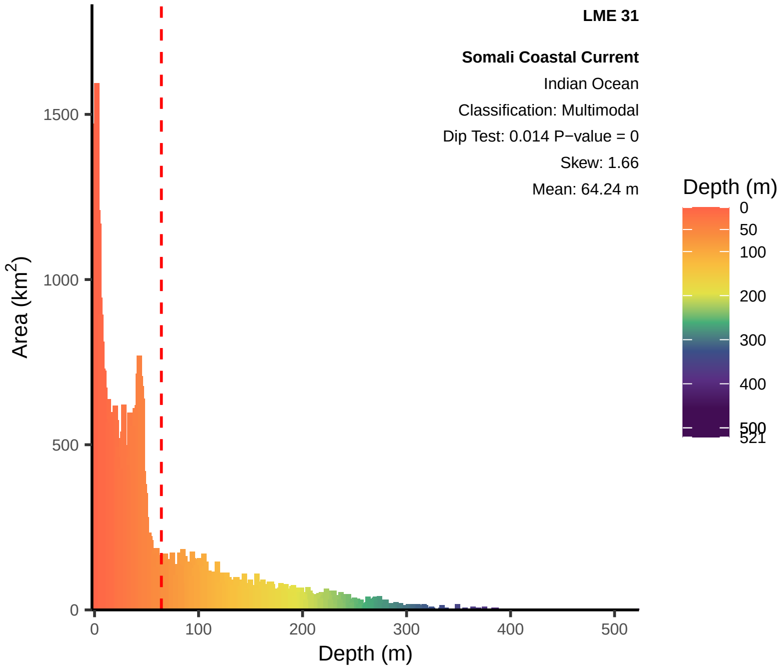
LME 31
Somali Coastal Current
Indian Ocean
Classification: Multimodal
Dip Test: 0.014 P−value = 0
Skew: 1.66
Mean: 64.24 m
Area (km^2)
Depth (m)
Depth (m)
0
50
100
200
300
400
500
521

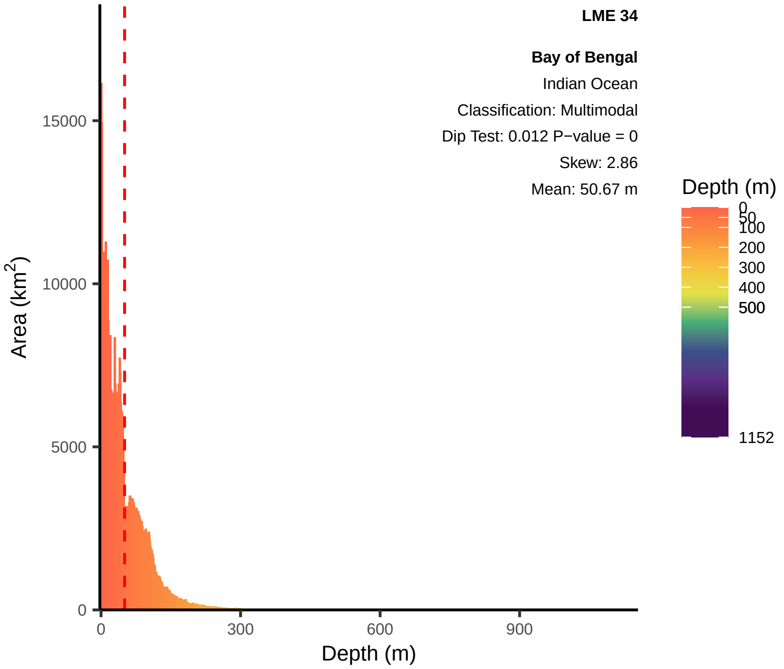
LME 34
Bay of Bengal
Indian Ocean
Classification: Multimodal
Dip Test: 0.012 P−value = 0
Skew: 2.86
Mean: 50.67 m
Area (km^2)
Depth (m)
Depth (m)
1152

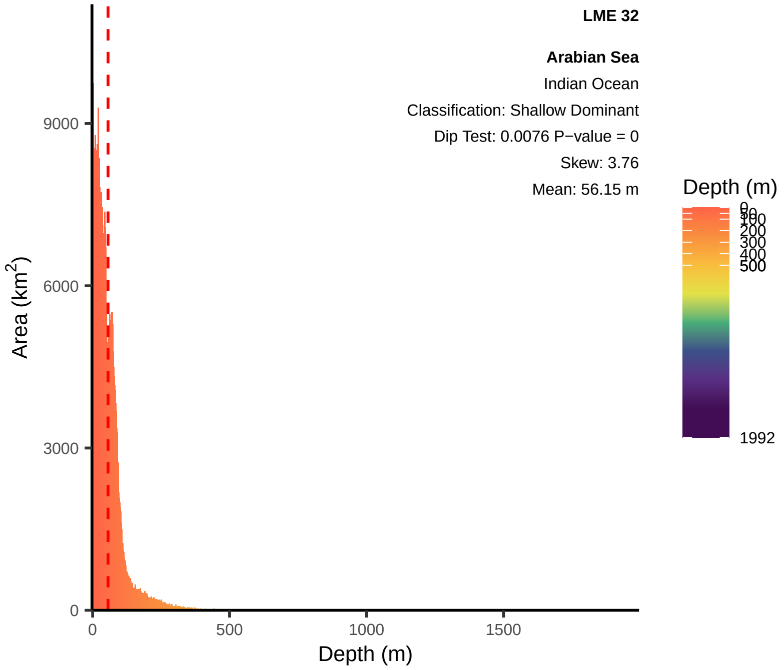
LME 32
Arabian Sea
Indian Ocean
Classification: Shallow Dominant
Dip Test: 0.0076 P−value = 0
Skew: 3.76
Mean: 56.15 m
Area (km^2)
Depth (m)
Depth (m)
1992

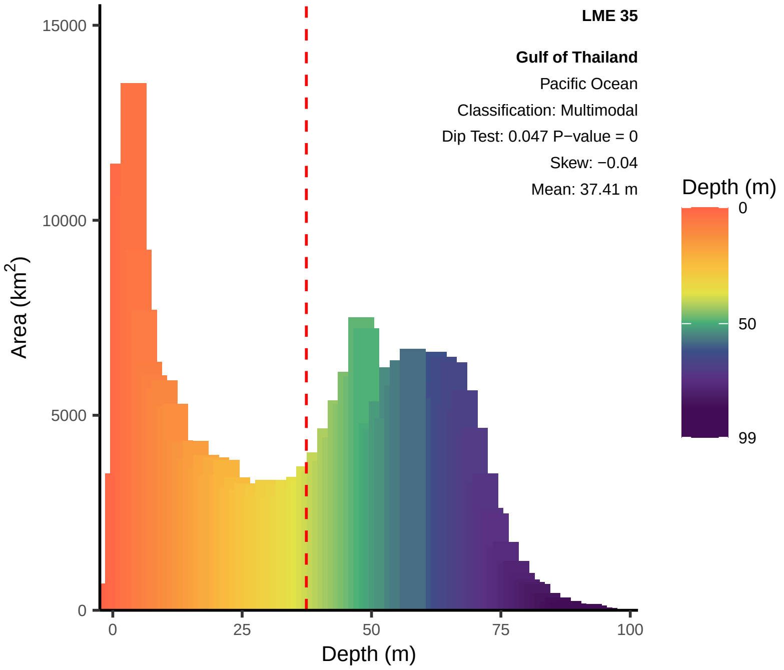
LME 35
Gulf of Thailand
Pacific Ocean
Classification: Multimodal
Dip Test: 0.047 P−value = 0
Skew: −0.04
Mean: 37.41 m
Area (km^2)
Depth (m)
Depth (m)
0
50
99

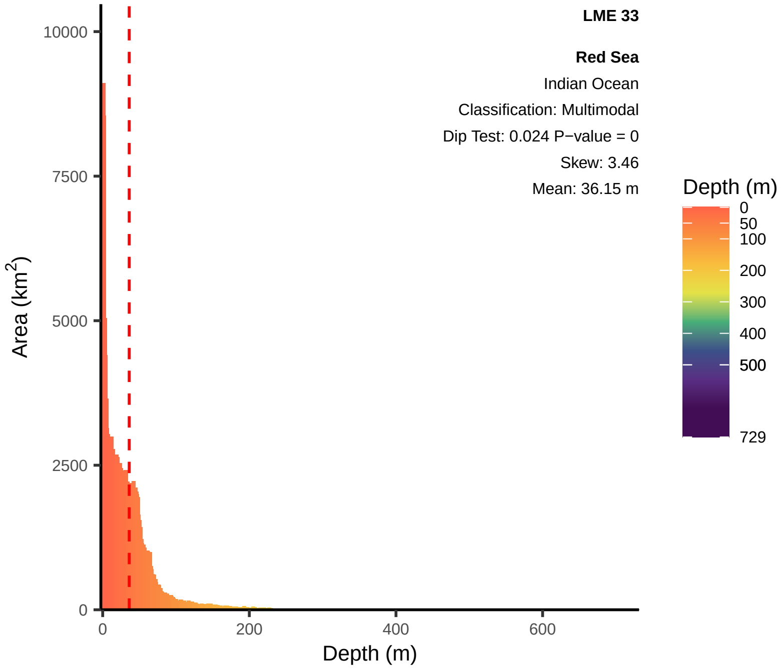
LME 33
Red Sea
Indian Ocean
Classification: Multimodal
Dip Test: 0.024 P−value = 0
Skew: 3.46
Mean: 36.15 m
Area (km^2)
Depth (m)
Depth (m)
0
50
100
200
300
400
500
729

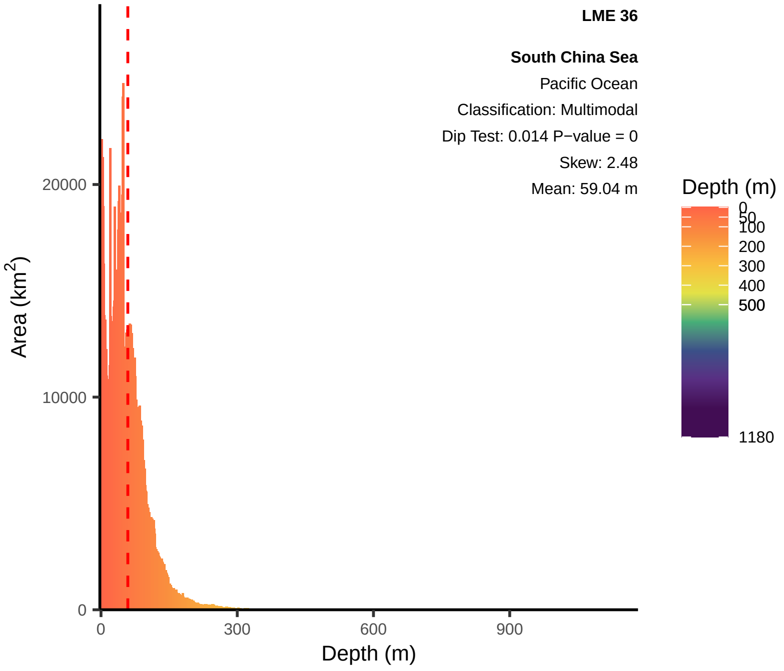
LME 36
South China Sea
Pacific Ocean
Classification: Multimodal
Dip Test: 0.014 P−value = 0
Skew: 2.48
Mean: 59.04 m
Area (km^2)
Depth (m)
Depth (m)
1180

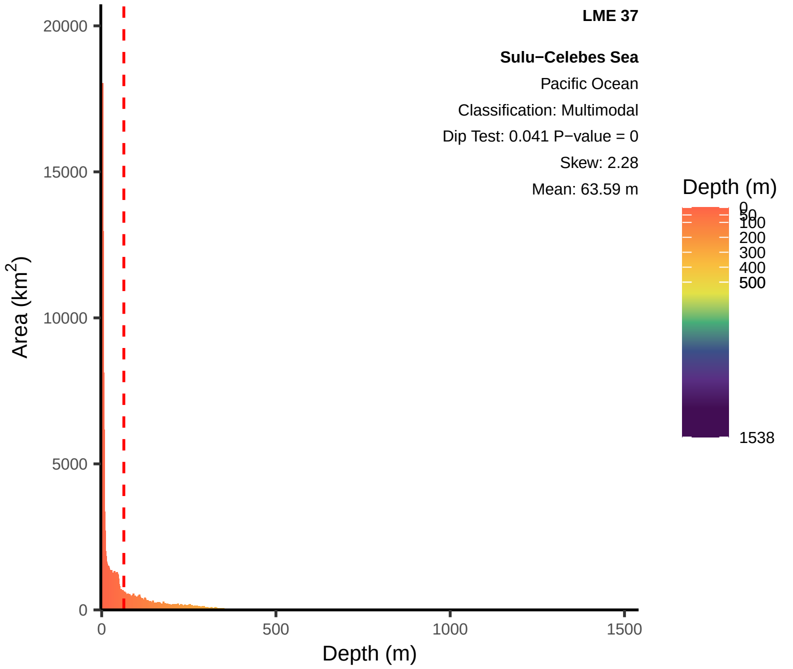
LME 37
Sulu−Celebes Sea
Pacific Ocean
Classification: Multimodal
Dip Test: 0.041 P−value = 0
Skew: 2.28
Mean: 63.59 m
Depth (m)
1538
Area (km2)
Depth (m)

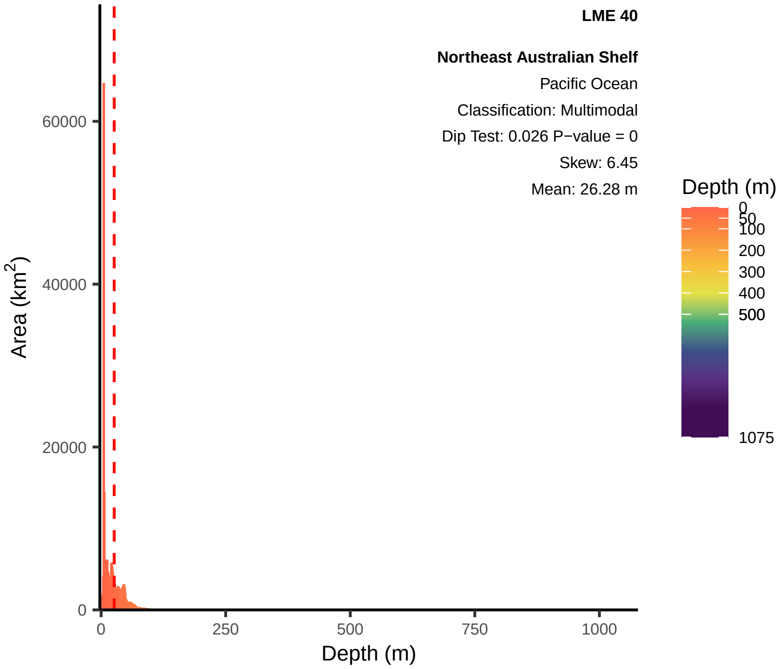
LME 40
Northeast Australian Shelf
Pacific Ocean
Classification: Multimodal
Dip Test: 0.026 P−value = 0
Skew: 6.45
Mean: 26.28 m
Depth (m)
1075
Area (km2)
Depth (m)

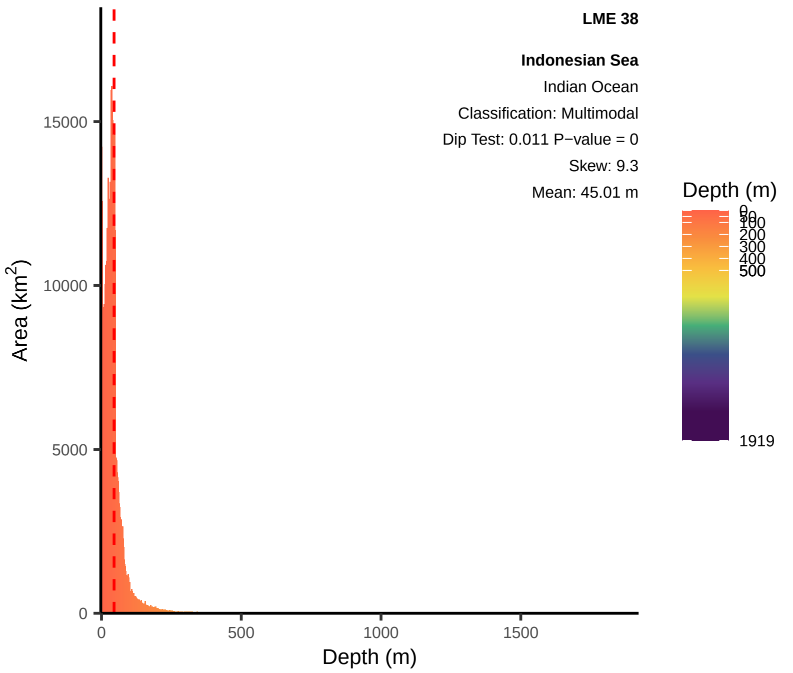
LME 38
Indonesian Sea
Indian Ocean
Classification: Multimodal
Dip Test: 0.011 P−value = 0
Skew: 9.3
Mean: 45.01 m
Depth (m)
1919
Area (km2)
Depth (m)

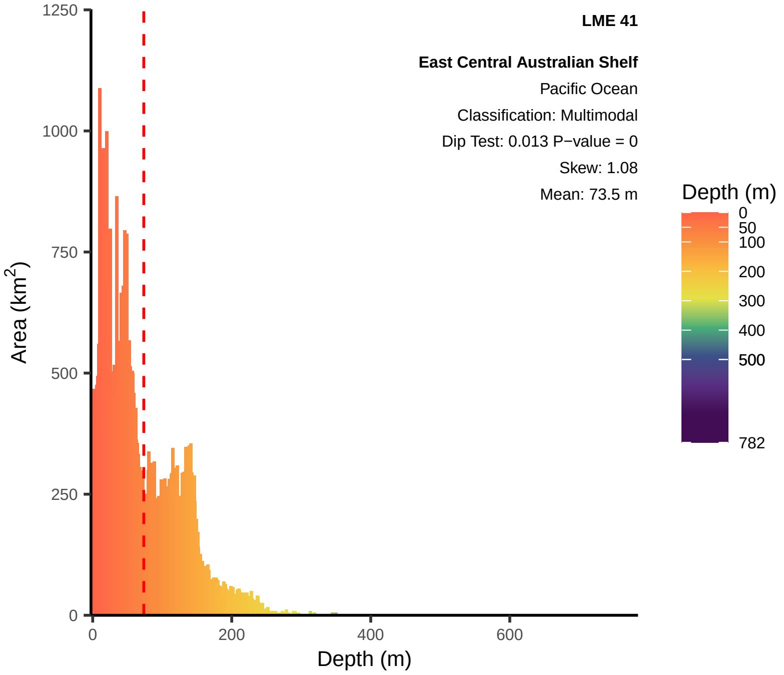
LME 41
East Central Australian Shelf
Pacific Ocean
Classification: Multimodal
Dip Test: 0.013 P−value = 0
Skew: 1.08
Mean: 73.5 m
Depth (m)
782
Area (km2)
Depth (m)

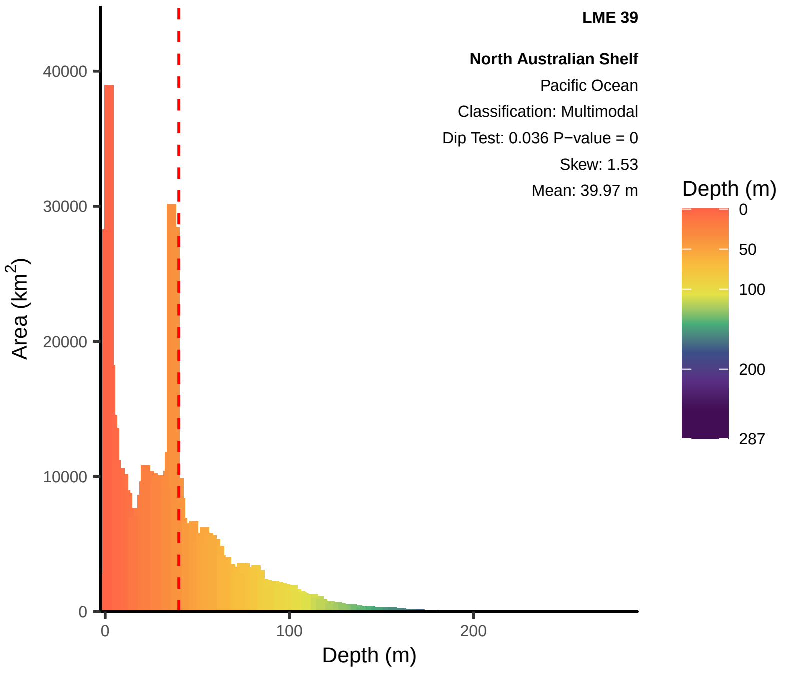
LME 39
North Australian Shelf
Pacific Ocean
Classification: Multimodal
Dip Test: 0.036 P−value = 0
Skew: 1.53
Mean: 39.97 m
Depth (m)
287
Area (km2)
Depth (m)

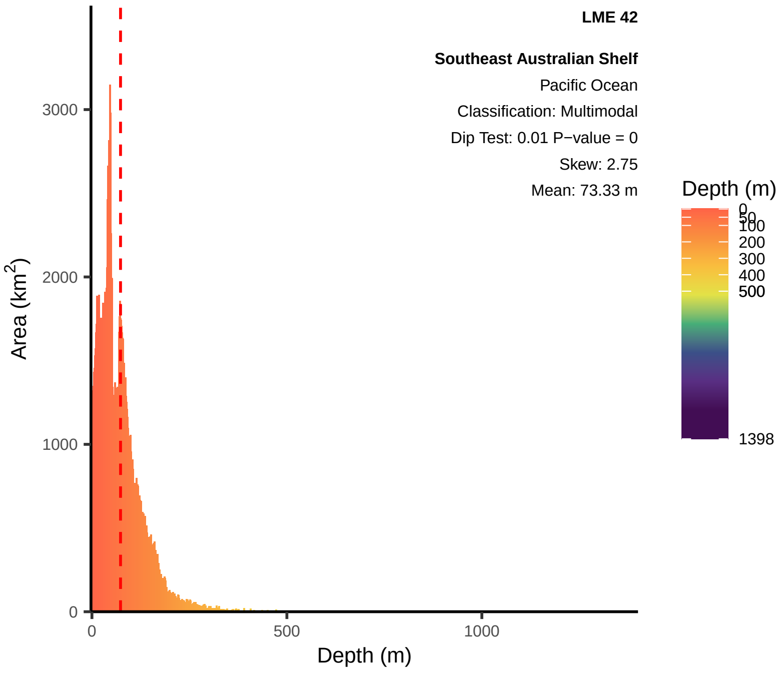
LME 42
Southeast Australian Shelf
Pacific Ocean
Classification: Multimodal
Dip Test: 0.01 P−value = 0
Skew: 2.75
Mean: 73.33 m
Depth (m)
1398
Area (km2)
Depth (m)

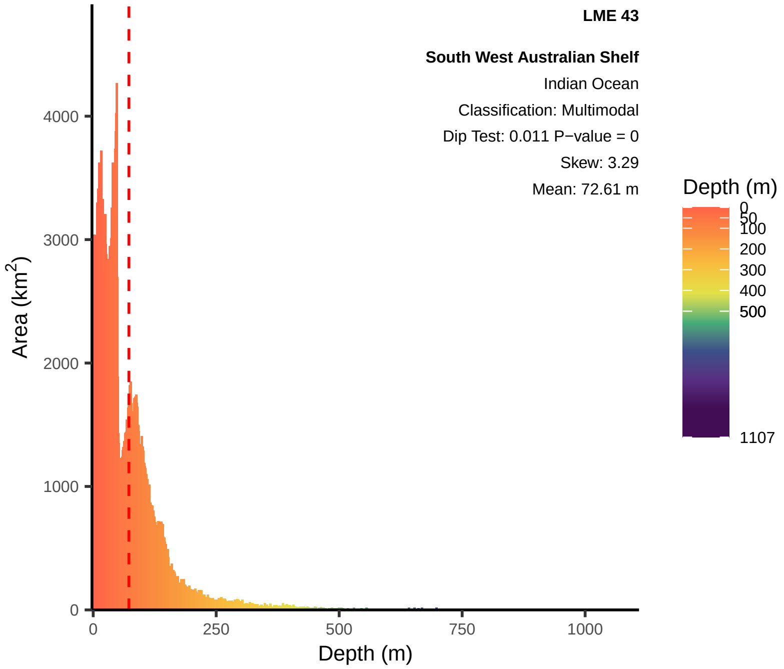
LME 43
South West Australian Shelf
Indian Ocean
Classification: Multimodal
Dip Test: 0.011 P−value = 0
Skew: 3.29
Mean: 72.61 m
Depth (m)
0
50
100
200
300
400
500
1107
Area (km2)
4000
3000
2000
1000
0
250
500
750
1000
Depth (m)

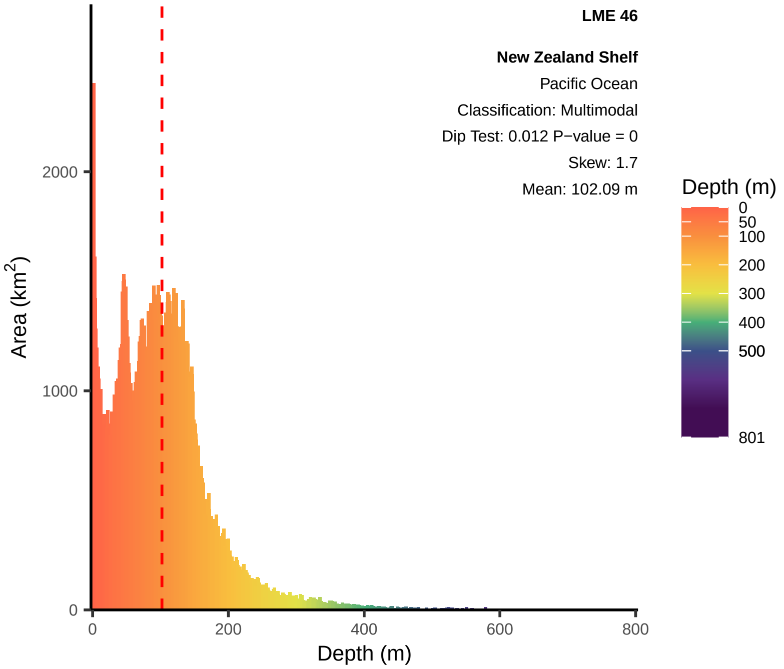
LME 46
New Zealand Shelf
Pacific Ocean
Classification: Multimodal
Dip Test: 0.012 P−value = 0
Skew: 1.7
Mean: 102.09 m
Depth (m)
0
50
100
200
300
400
500
801
Area (km2)
2000
1000
0
200
400
600
800
Depth (m)

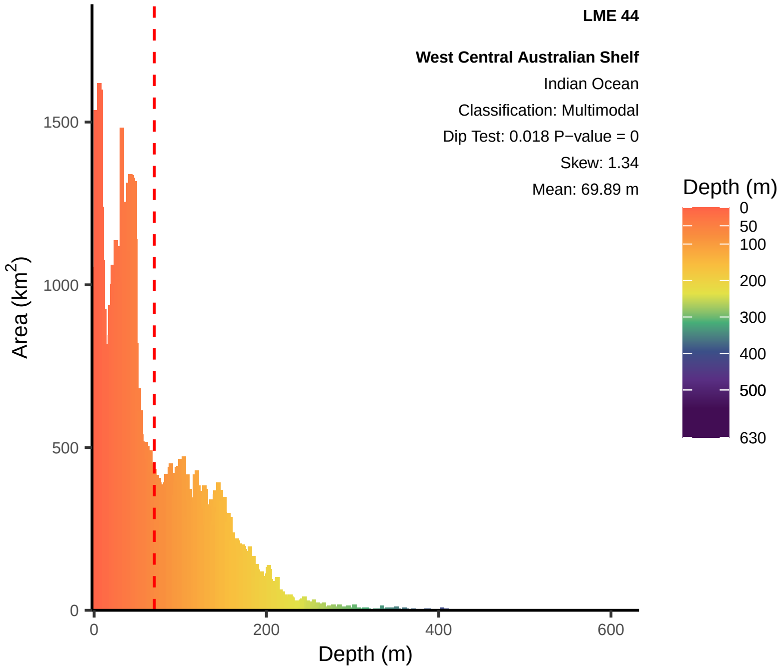
LME 44
West Central Australian Shelf
Indian Ocean
Classification: Multimodal
Dip Test: 0.018 P−value = 0
Skew: 1.34
Mean: 69.89 m
Depth (m)
0
50
100
200
300
400
500
630
Area (km2)
1500
1000
500
0
200
400
600
Depth (m)

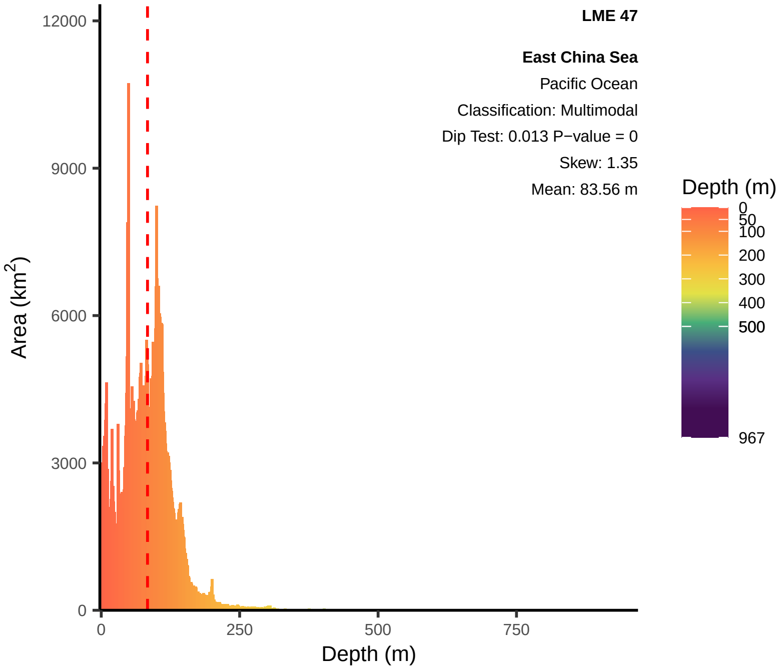
LME 47
East China Sea
Pacific Ocean
Classification: Multimodal
Dip Test: 0.013 P−value = 0
Skew: 1.35
Mean: 83.56 m
Depth (m)
0
50
100
200
300
400
500
967
Area (km2)
12000
9000
6000
3000
0
250
500
750
Depth (m)

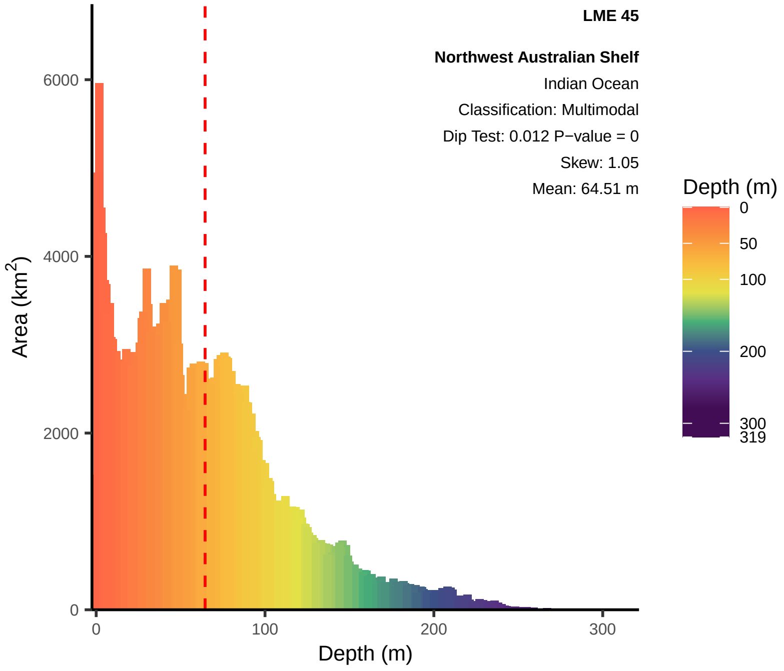
LME 45
Northwest Australian Shelf
Indian Ocean
Classification: Multimodal
Dip Test: 0.012 P−value = 0
Skew: 1.05
Mean: 64.51 m
Depth (m)
0
50
100
200
300
319
Area (km2)
6000
4000
2000
0
100
200
300
Depth (m)

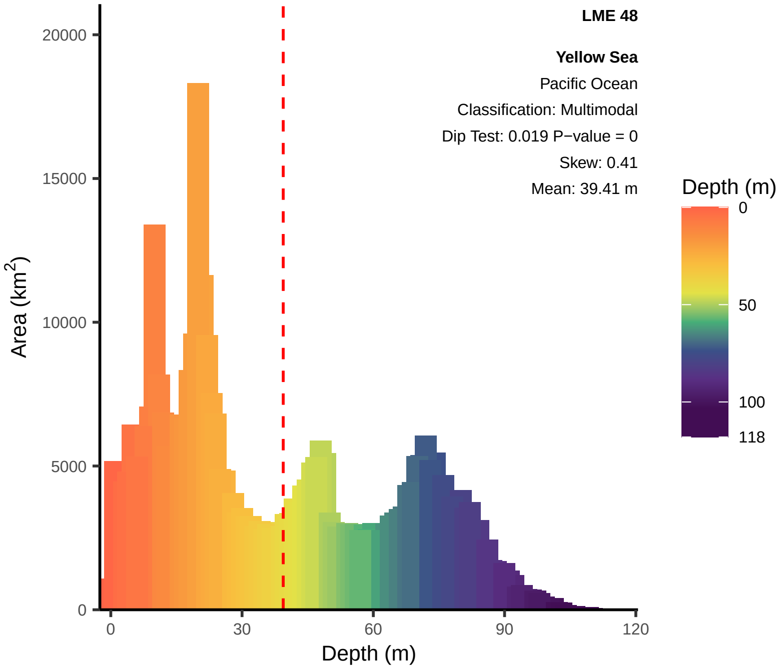
LME 48
Yellow Sea
Pacific Ocean
Classification: Multimodal
Dip Test: 0.019 P−value = 0
Skew: 0.41
Mean: 39.41 m
Depth (m)
0
50
100
118
Area (km2)
20000
15000
10000
5000
0
30
60
90
120
Depth (m)

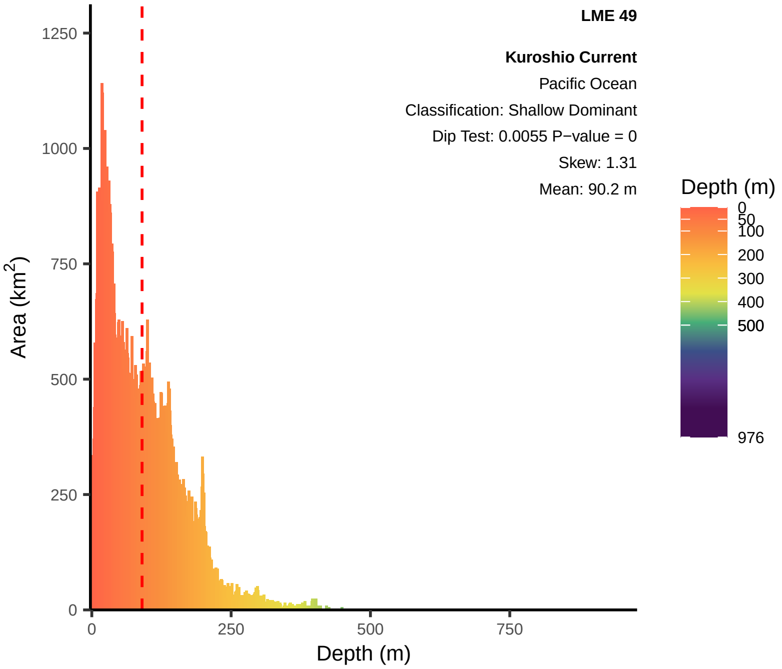
LME 49
Kuroshio Current
Pacific Ocean
Classification: Shallow Dominant
Dip Test: 0.0055 P−value = 0
Skew: 1.31
Mean: 90.2 m
Area (km^2)
Depth (m)
Depth (m)
0
50
100
200
300
400
500
976

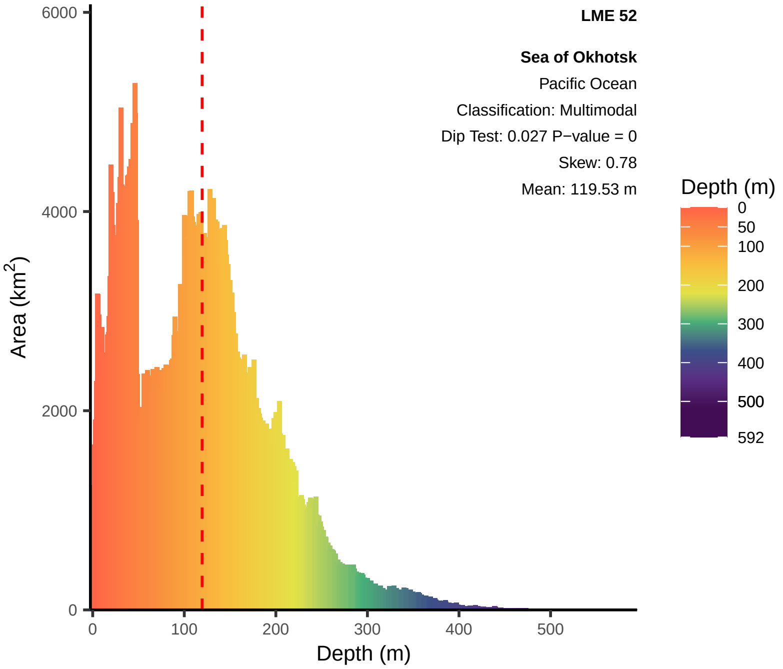
LME 52
Sea of Okhotsk
Pacific Ocean
Classification: Multimodal
Dip Test: 0.027 P−value = 0
Skew: 0.78
Mean: 119.53 m
Area (km^2)
Depth (m)
Depth (m)
0
50
100
200
300
400
500
592

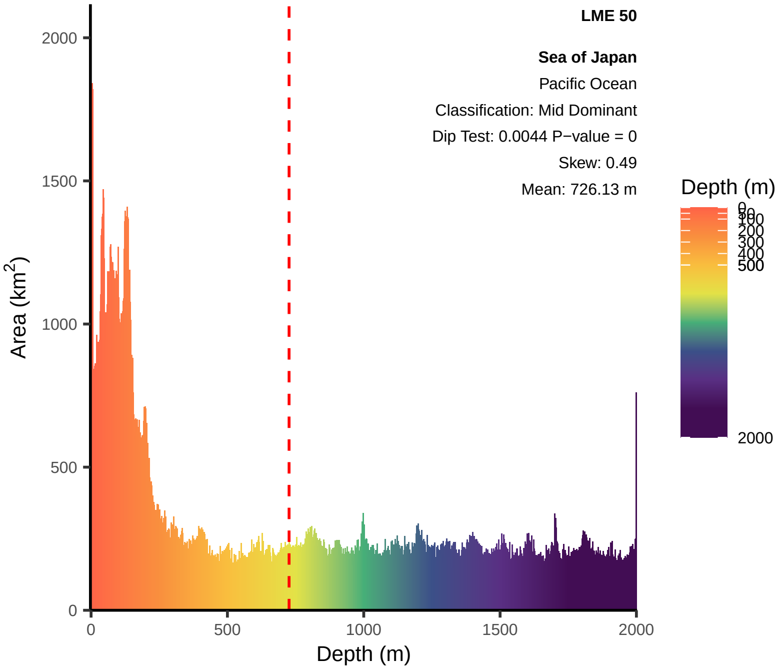
LME 50
Sea of Japan
Pacific Ocean
Classification: Mid Dominant
Dip Test: 0.0044 P−value = 0
Skew: 0.49
Mean: 726.13 m
Area (km^2)
Depth (m)
Depth (m)
2000

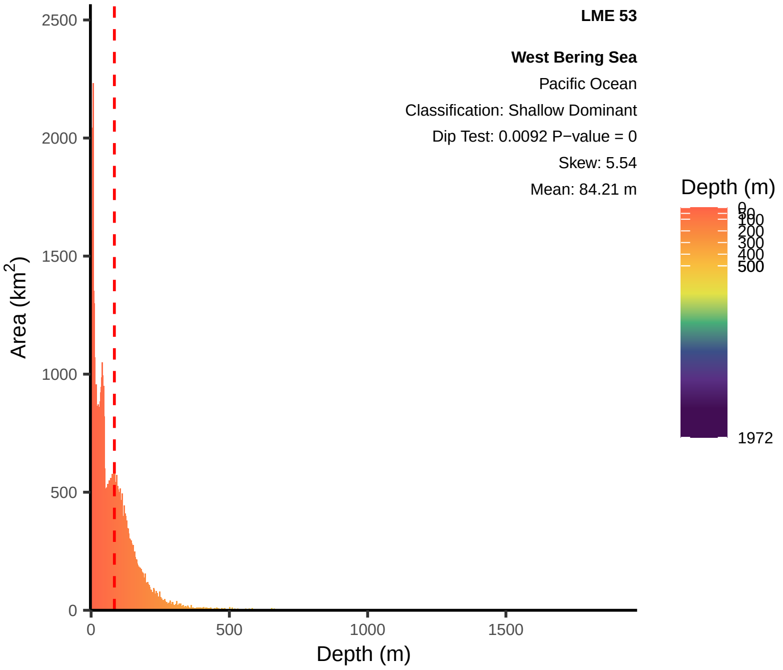
LME 53
West Bering Sea
Pacific Ocean
Classification: Shallow Dominant
Dip Test: 0.0092 P−value = 0
Skew: 5.54
Mean: 84.21 m
Area (km^2)
Depth (m)
Depth (m)
1972

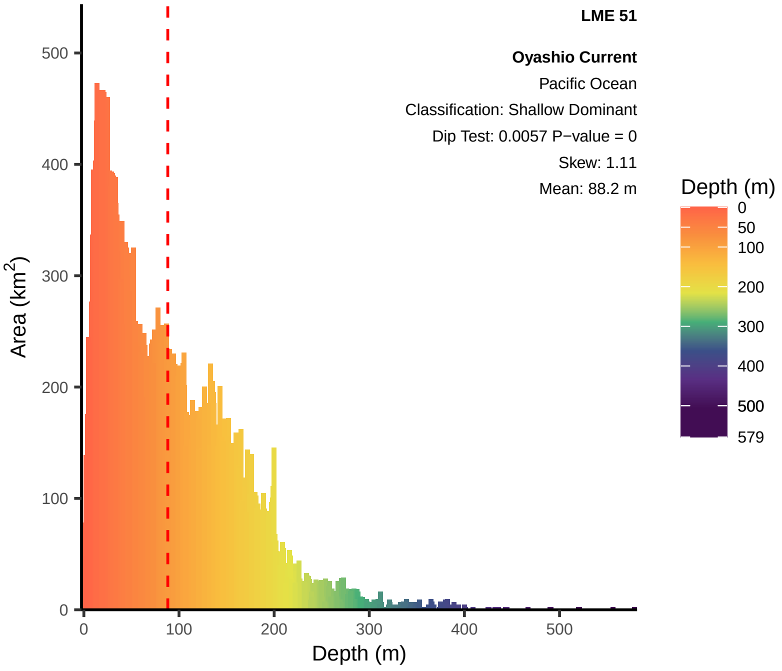
LME 51
Oyashio Current
Pacific Ocean
Classification: Shallow Dominant
Dip Test: 0.0057 P−value = 0
Skew: 1.11
Mean: 88.2 m
Area (km^2)
Depth (m)
Depth (m)
0
50
100
200
300
400
500
579

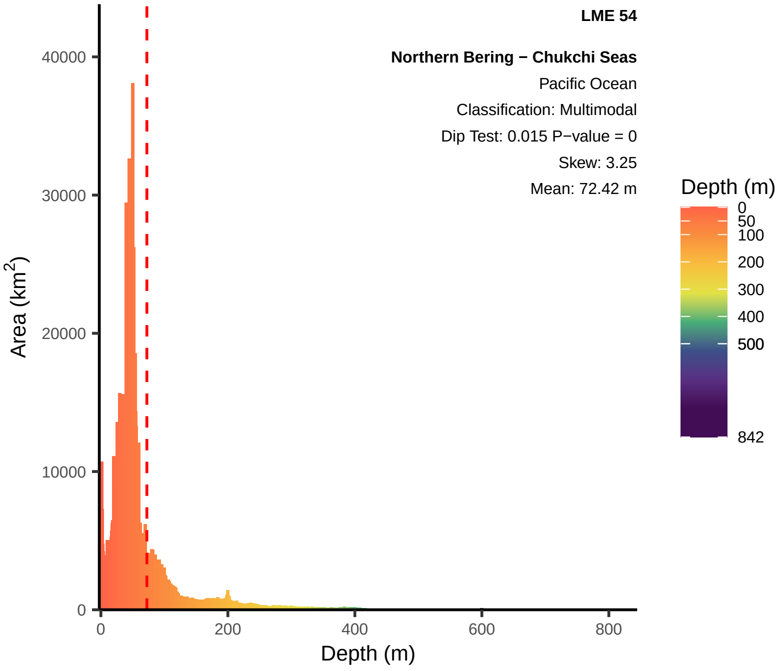
LME 54
Northern Bering − Chukchi Seas
Pacific Ocean
Classification: Multimodal
Dip Test: 0.015 P−value = 0
Skew: 3.25
Mean: 72.42 m
Area (km^2)
Depth (m)
Depth (m)
0
50
100
200
300
400
500
842

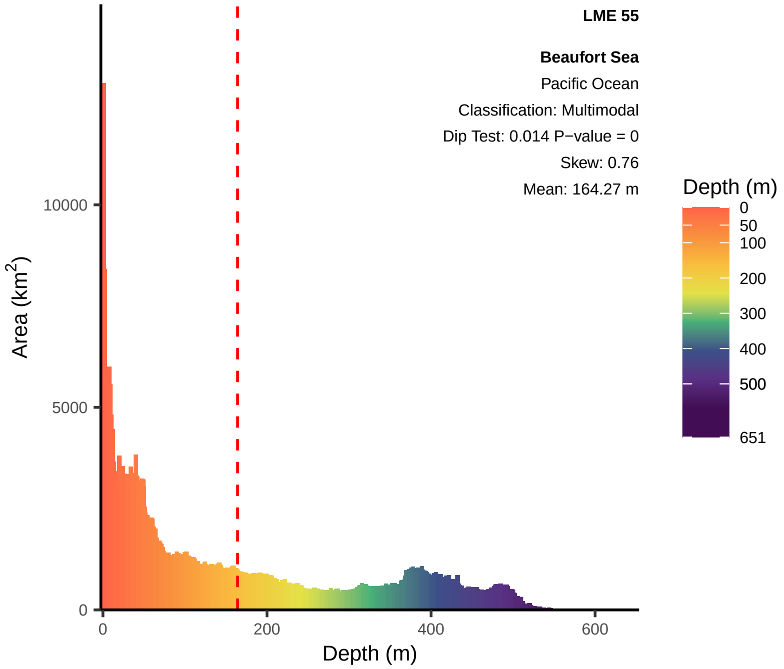
LME 55
Beaufort Sea
Pacific Ocean
Classification: Multimodal
Dip Test: 0.014 P−value = 0
Skew: 0.76
Mean: 164.27 m
Depth (m)
Area (km2)
Depth (m)
0
50
100
200
300
400
500
651
0
5000
10000
0
200
400
600

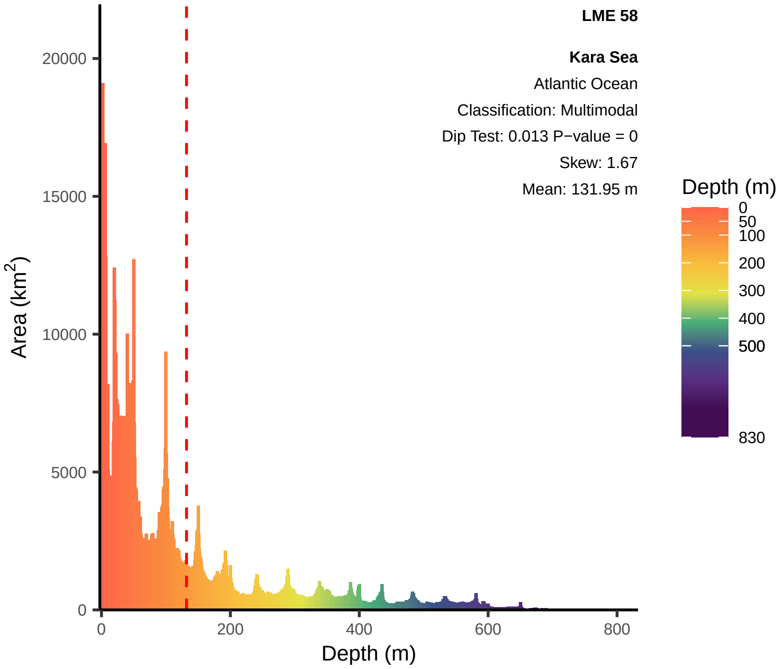
LME 58
Kara Sea
Atlantic Ocean
Classification: Multimodal
Dip Test: 0.013 P−value = 0
Skew: 1.67
Mean: 131.95 m
Depth (m)
Area (km2)
Depth (m)
0
50
100
200
300
400
500
830
0
5000
10000
15000
20000
0
200
400
600
800

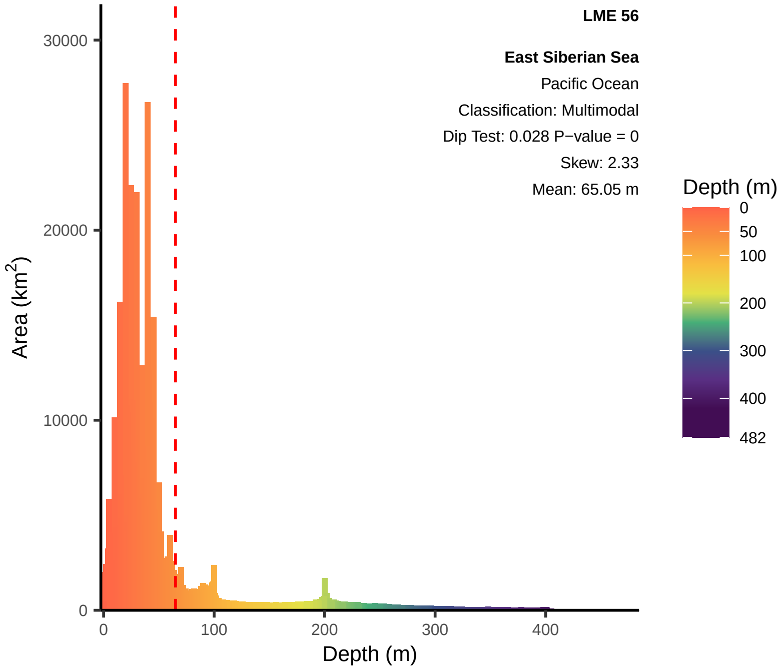
LME 56
East Siberian Sea
Pacific Ocean
Classification: Multimodal
Dip Test: 0.028 P−value = 0
Skew: 2.33
Mean: 65.05 m
Depth (m)
Area (km2)
Depth (m)
0
50
100
200
300
400
482
0
10000
20000
30000
0
100
200
300
400

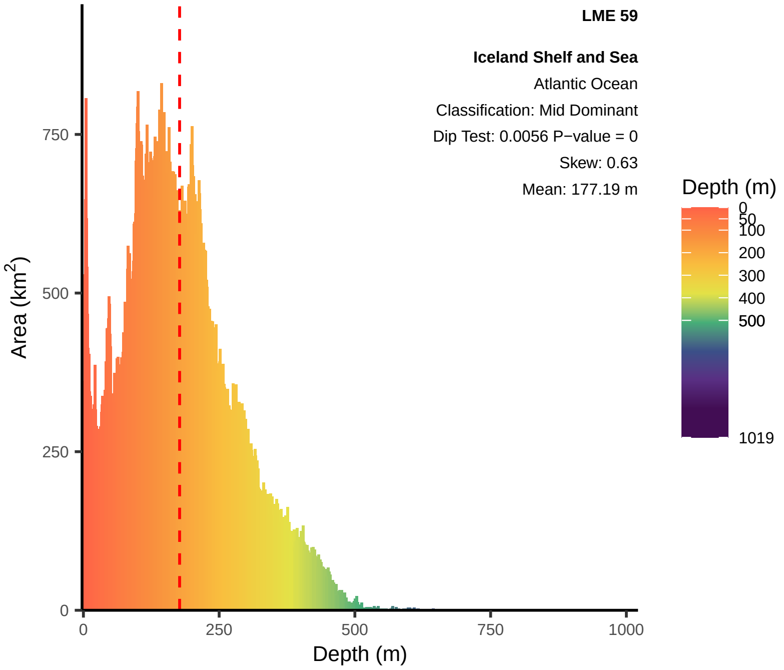
LME 59
Iceland Shelf and Sea
Atlantic Ocean
Classification: Mid Dominant
Dip Test: 0.0056 P−value = 0
Skew: 0.63
Mean: 177.19 m
Depth (m)
Area (km2)
Depth (m)
0
50
100
200
300
400
500
1019
0
250
500
750
0
250
500
750
1000

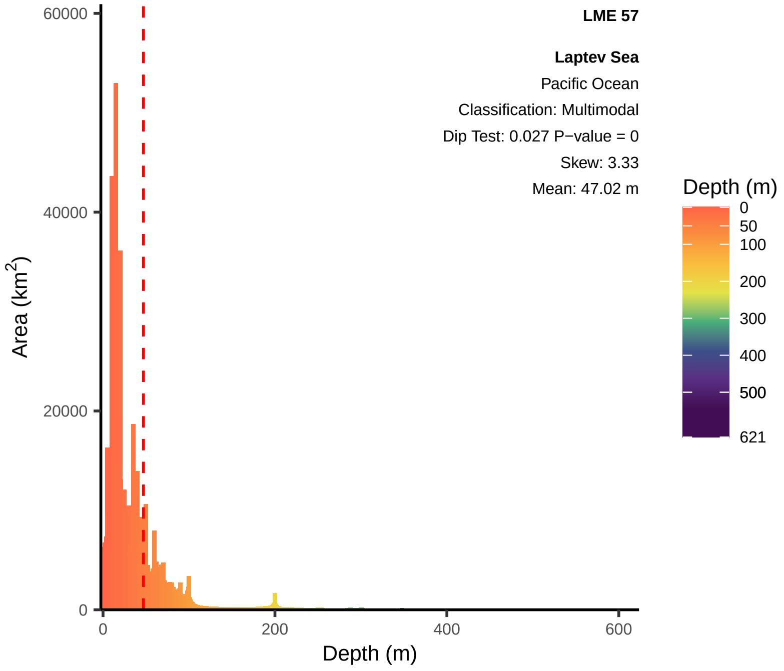
LME 57
Laptev Sea
Pacific Ocean
Classification: Multimodal
Dip Test: 0.027 P−value = 0
Skew: 3.33
Mean: 47.02 m
Depth (m)
Area (km2)
Depth (m)
0
50
100
200
300
400
500
621
0
20000
40000
60000
0
200
400
600

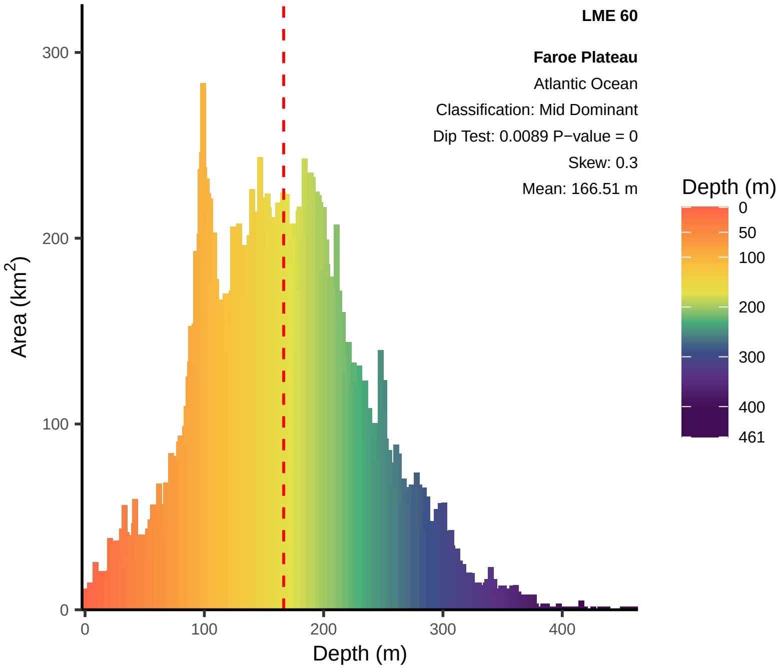
LME 60
Faroe Plateau
Atlantic Ocean
Classification: Mid Dominant
Dip Test: 0.0089 P−value = 0
Skew: 0.3
Mean: 166.51 m
Depth (m)
Area (km2)
Depth (m)
0
50
100
200
300
400
461
0
100
200
300
0
100
200
300
400

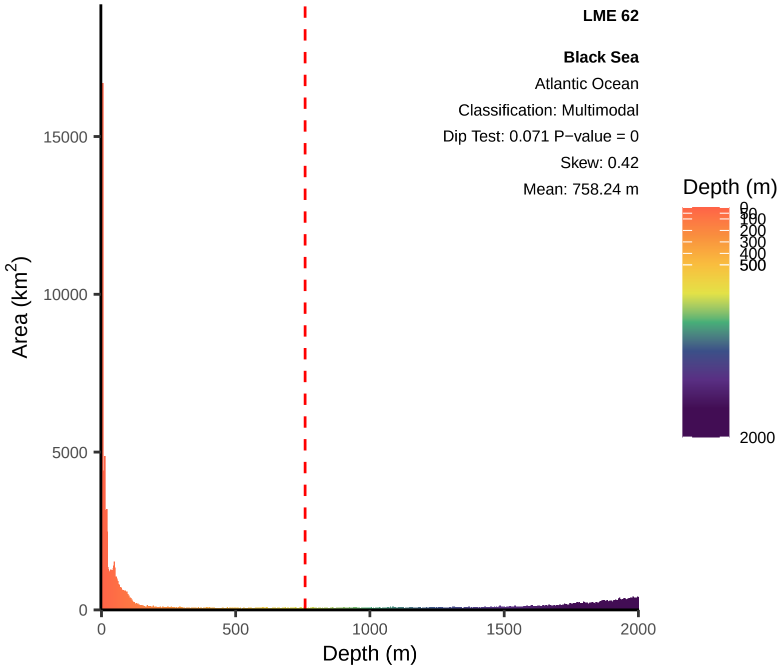
LME 62
Black Sea
Atlantic Ocean
Classification: Multimodal
Dip Test: 0.071 P−value = 0
Skew: 0.42
Mean: 758.24 m
Area (km2)
Depth (m)
Depth (m)
2000

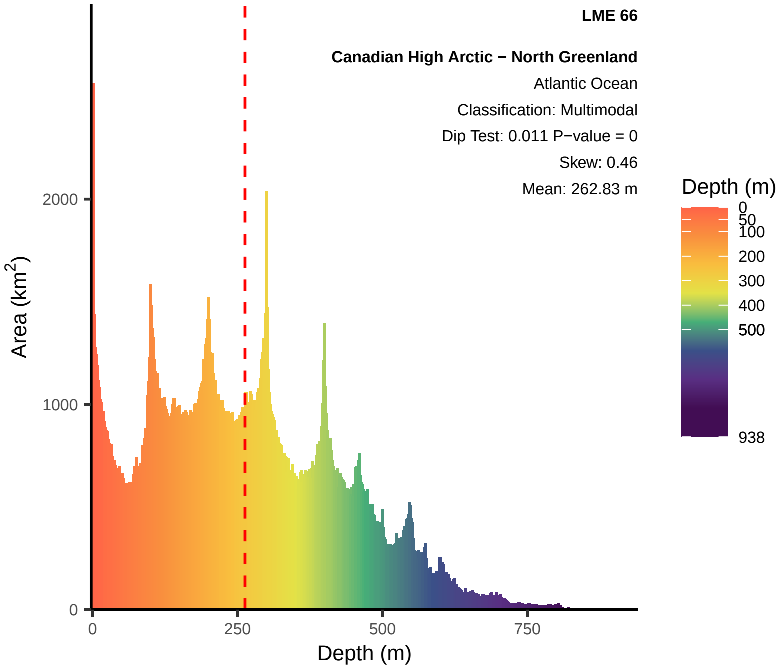
LME 66
Canadian High Arctic − North Greenland
Atlantic Ocean
Classification: Multimodal
Dip Test: 0.011 P−value = 0
Skew: 0.46
Mean: 262.83 m
Area (km2)
Depth (m)
Depth (m)
938

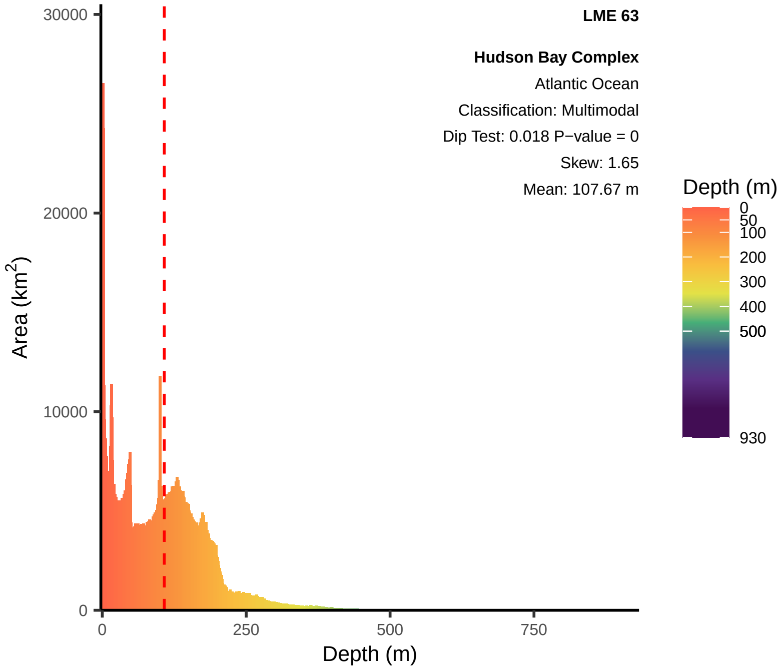
LME 63
Hudson Bay Complex
Atlantic Ocean
Classification: Multimodal
Dip Test: 0.018 P−value = 0
Skew: 1.65
Mean: 107.67 m
Area (km2)
Depth (m)
Depth (m)
930

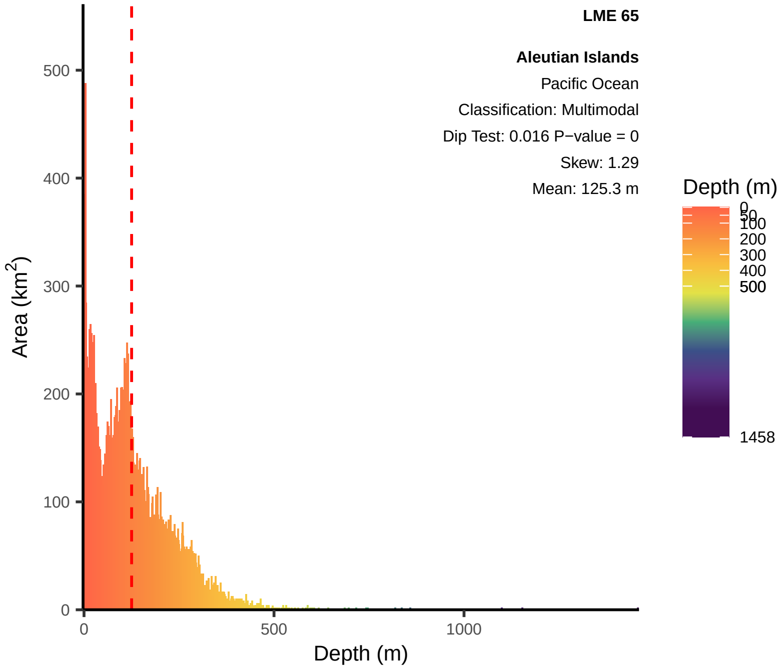
LME 65
Aleutian Islands
Pacific Ocean
Classification: Multimodal
Dip Test: 0.016 P−value = 0
Skew: 1.29
Mean: 125.3 m
Area (km2)
Depth (m)
Depth (m)
1458

Figure S7. Percent change in species richness by 2˚ latitudinal bin along coastlines of Pacific Ocean predicted using species area relationships with a z value of 0.175 (minimum, Frank and Shackell 2001; left panels), 0.38 (mean, Levin et al. 2009; middle panels also shown in Figure 3 in the main text), and 0.62 (max, Nanami & Nishihira 2003; right panels). Each 2˚ depth bin is defined as a community and has the potential to lose (purple) or gain (orange) species.

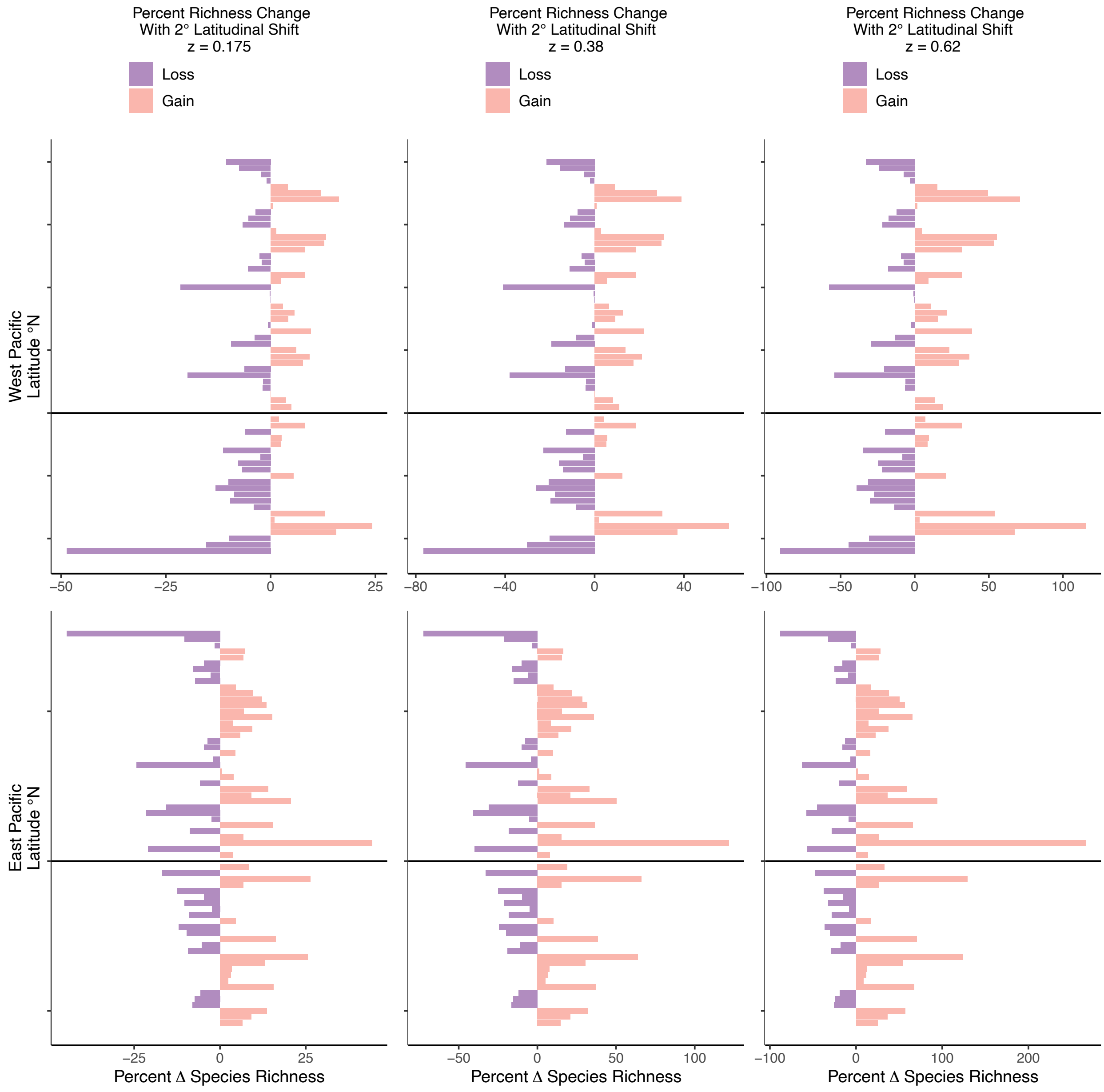

Figure S8. Percent change in species richness by 2° latitudinal bin along west (above) and east (below) coastlines of Atlantic Ocean. See Figure S7 for more details.

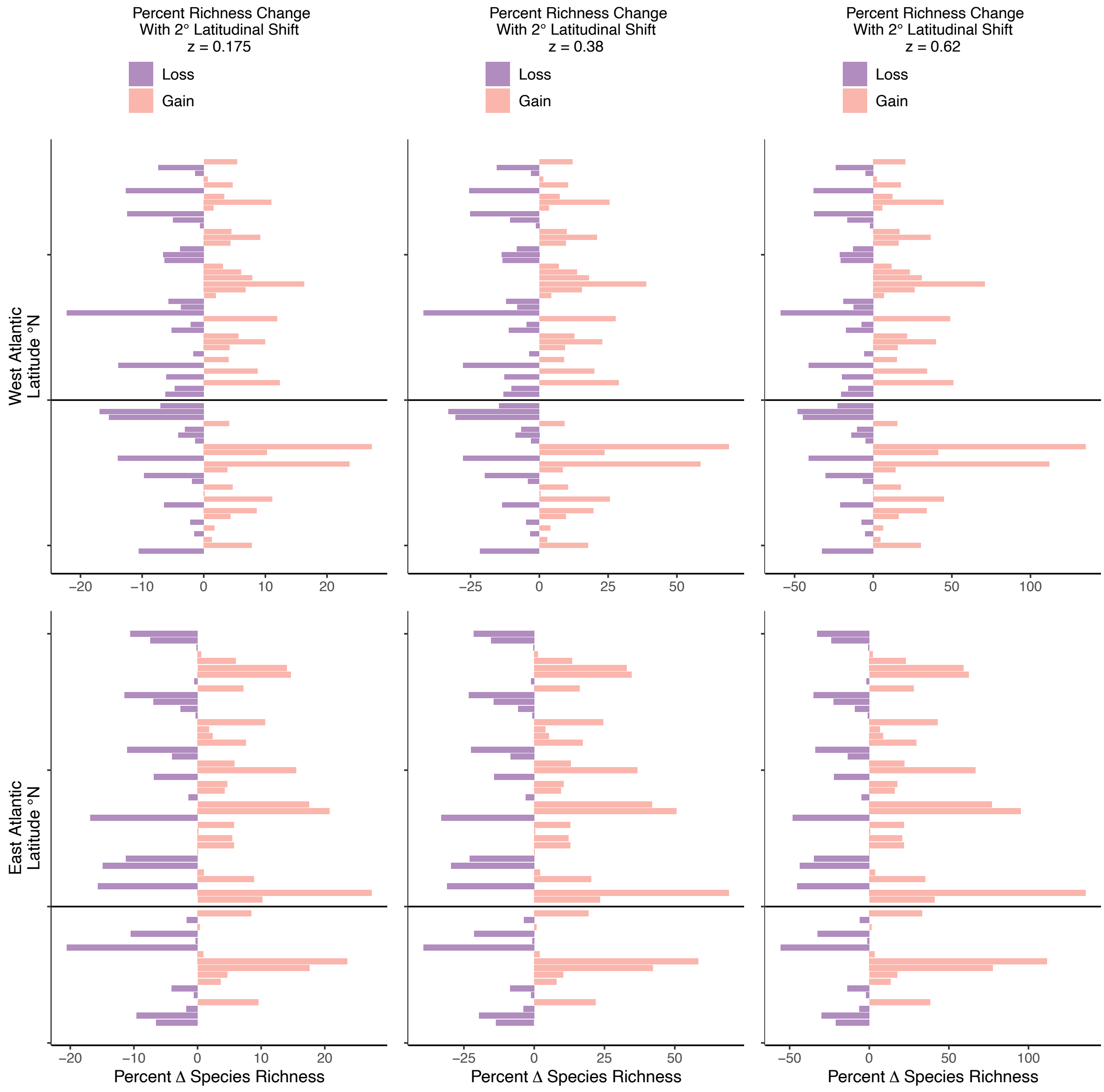

Figure S9. Percent change in species richness by 2˚ latitudinal bin along west (above) and east (below) coastlines of Indian Ocean. See Figure S7 for more details.

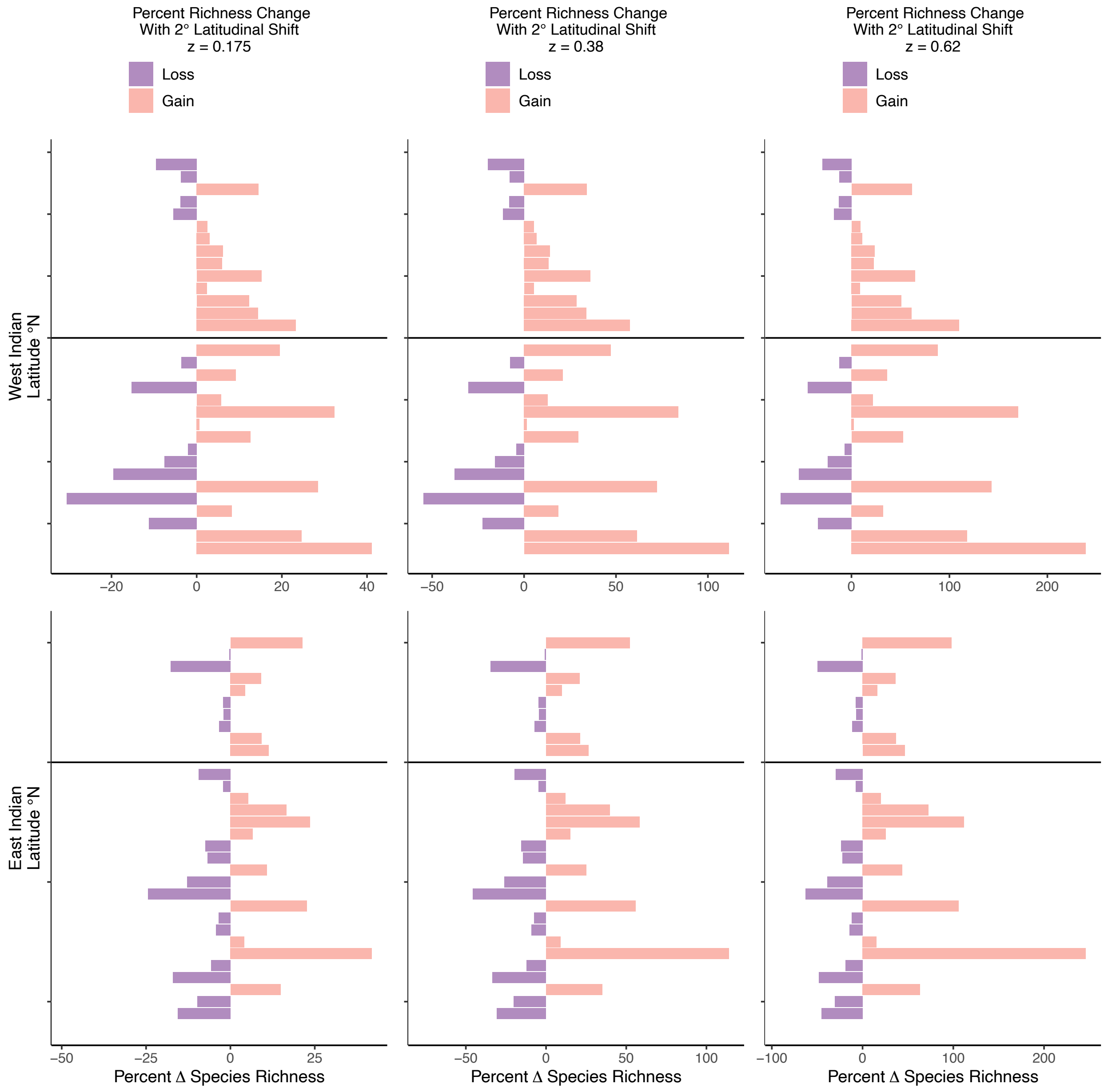

Figure S10a-c. Percent change in species richness by depth predicted using species area relationships with a z value of 0.175 (minimum, Frank and Shackell 2001; a.), 0.38 (mean, Levin et al. 2009; b.), and 0.62 (max, Nanami & Nishihira 2003; c.) with a 15m (40 year) depth shift for all 64 LMEs included in the analysis. Each 15m depth bin is defined as a community and has the potential to lose (purple) or gain (orange) species.

Fig S10 a.

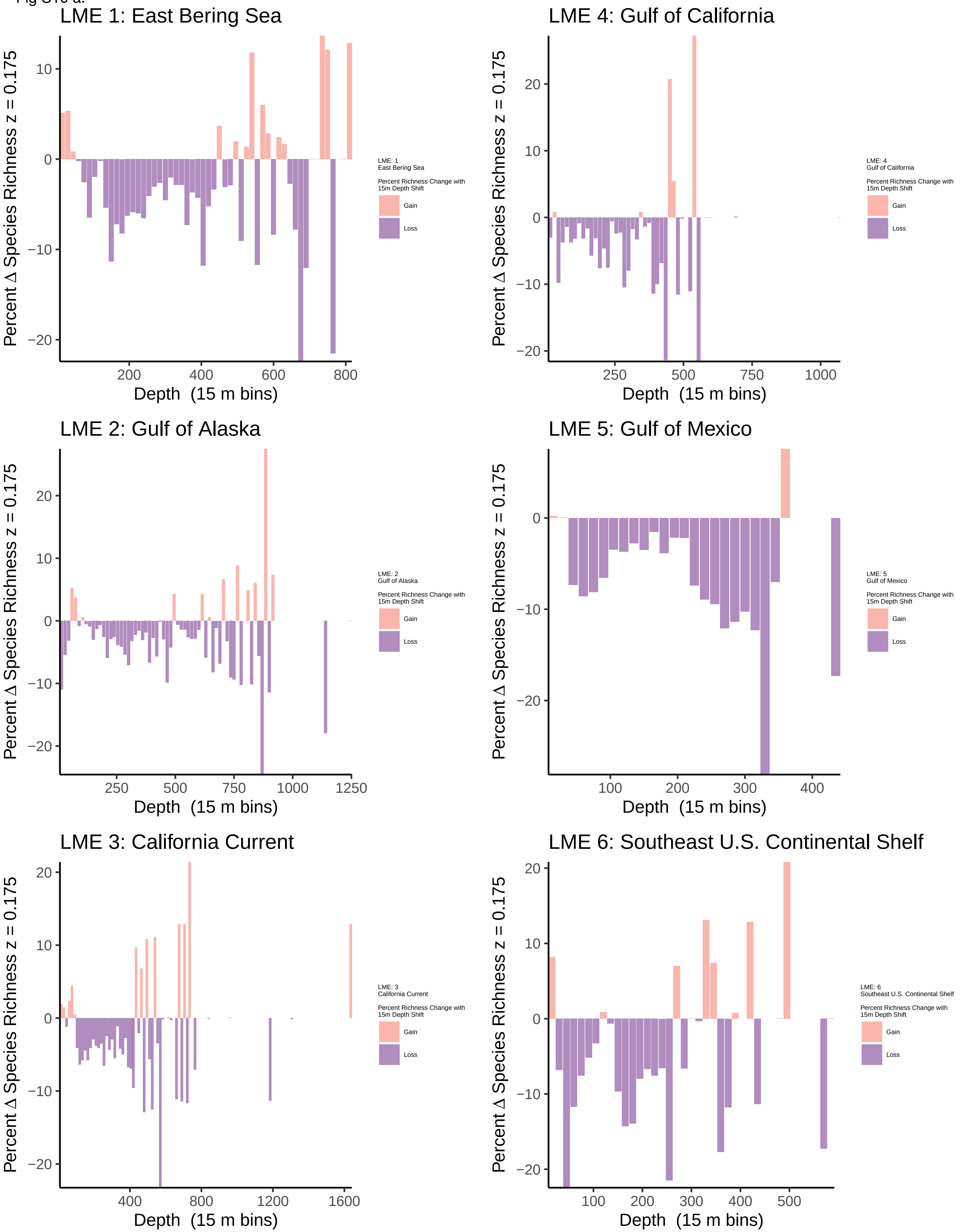
LME 1: East Bering Sea
Percent Δ Species Richness z = 0.175
Depth (15 m bins)
LME: 1
East Bering Sea
Percent Richness Change with
15m Depth Shift
Gain
Loss
LME 4: Gulf of California
Percent Δ Species Richness z = 0.175
Depth (15 m bins)
LME: 4
Gulf of California
Percent Richness Change with
15m Depth Shift
Gain
Loss
LME 2: Gulf of Alaska
Percent Δ Species Richness z = 0.175
Depth (15 m bins)
LME: 2
Gulf of Alaska
Percent Richness Change with
15m Depth Shift
Gain
Loss
LME 5: Gulf of Mexico
Percent Δ Species Richness z = 0.175
Depth (15 m bins)
LME: 5
Gulf of Mexico
Percent Richness Change with
15m Depth Shift
Gain
Loss
LME 3: California Current
Percent Δ Species Richness z = 0.175
Depth (15 m bins)
LME: 3
California Current
Percent Richness Change with
15m Depth Shift
Gain
Loss
LME 6: Southeast U.S. Continental Shelf
Percent Δ Species Richness z = 0.175
Depth (15 m bins)
LME: 6
Southeast U.S. Continental Shelf
Percent Richness Change with
15m Depth Shift
Gain
Loss

LME 7: Northeast U.S. Continental Shelf

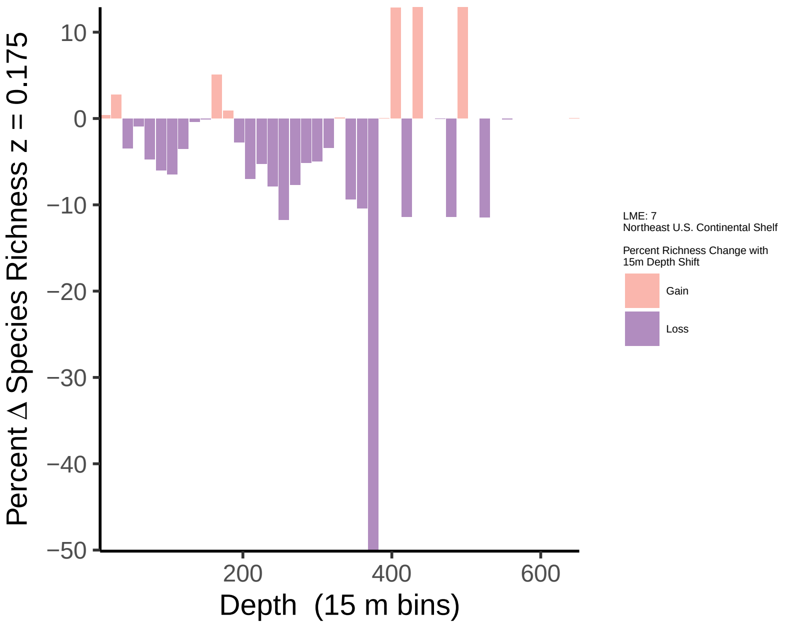

LME 10: Insular Pacific−Hawaiian

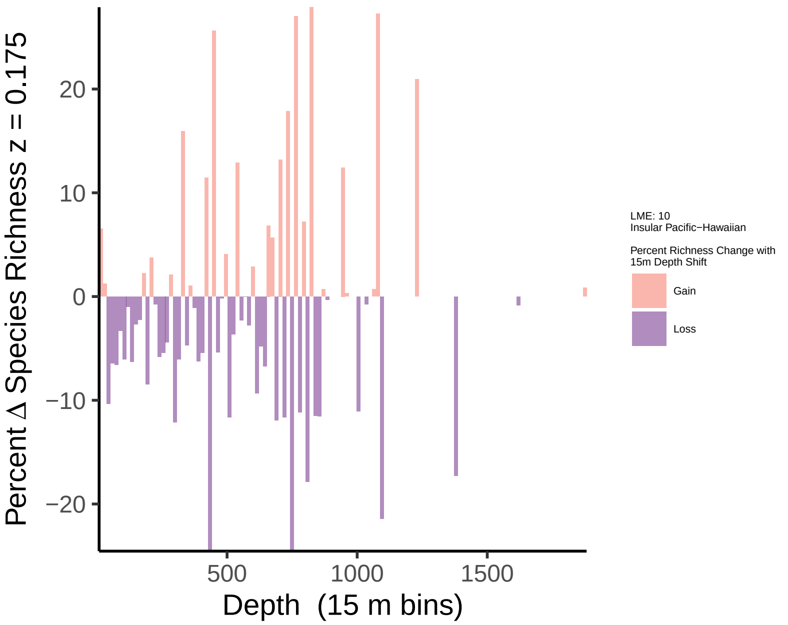

LME 8: Scotian Shelf

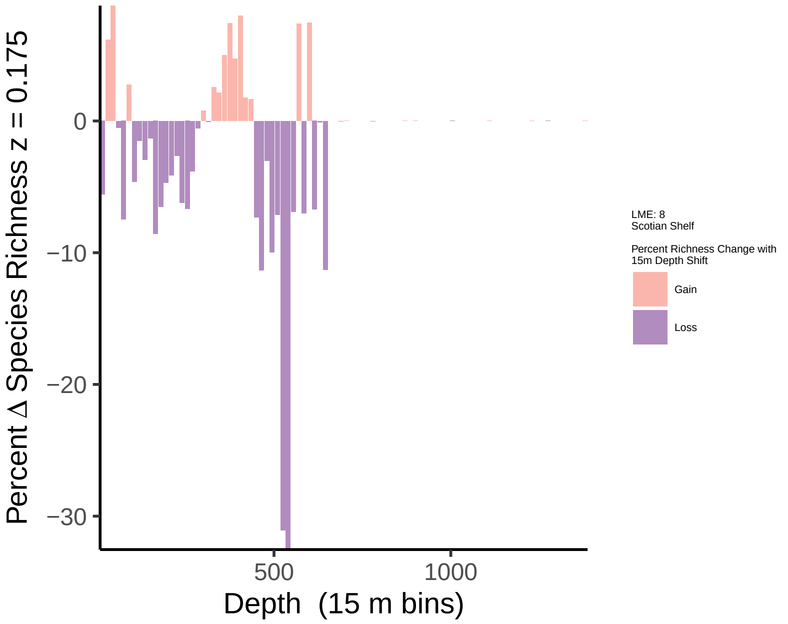

LME 11: Pacific Central−American Coastal

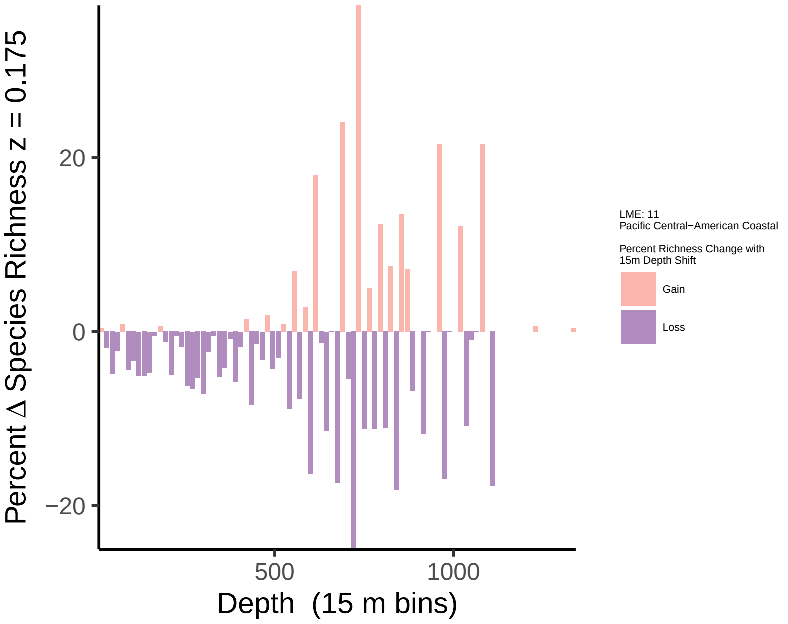

LME 9: Labrador − Newfoundland

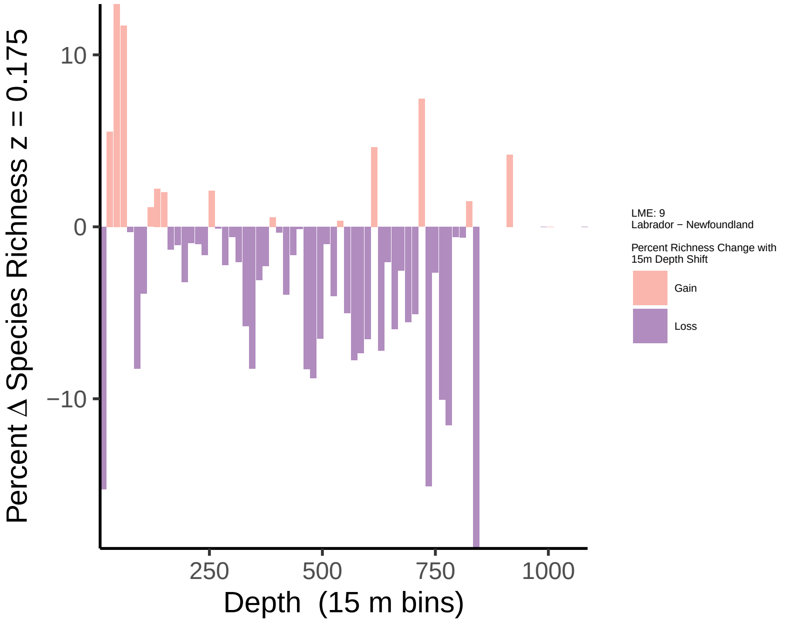

LME 12: Caribbean Sea

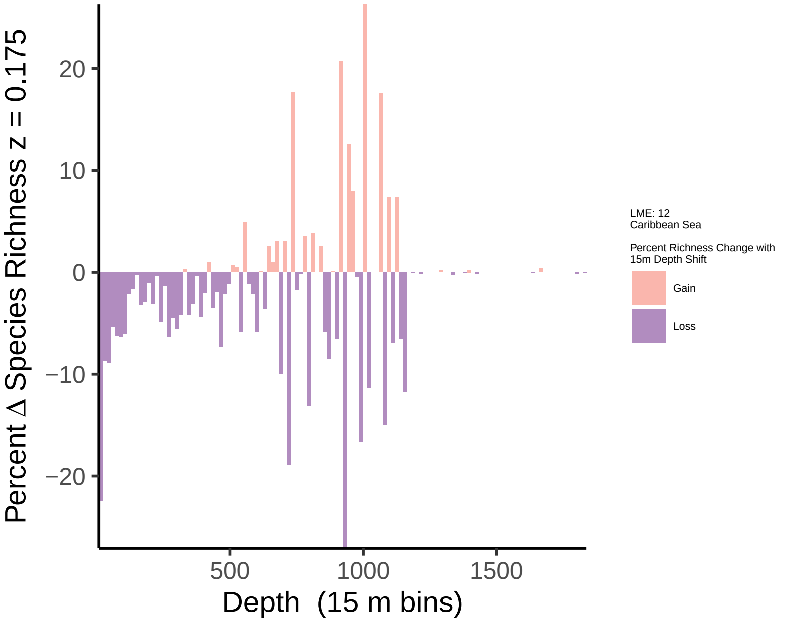

LME 13: Humboldt Current

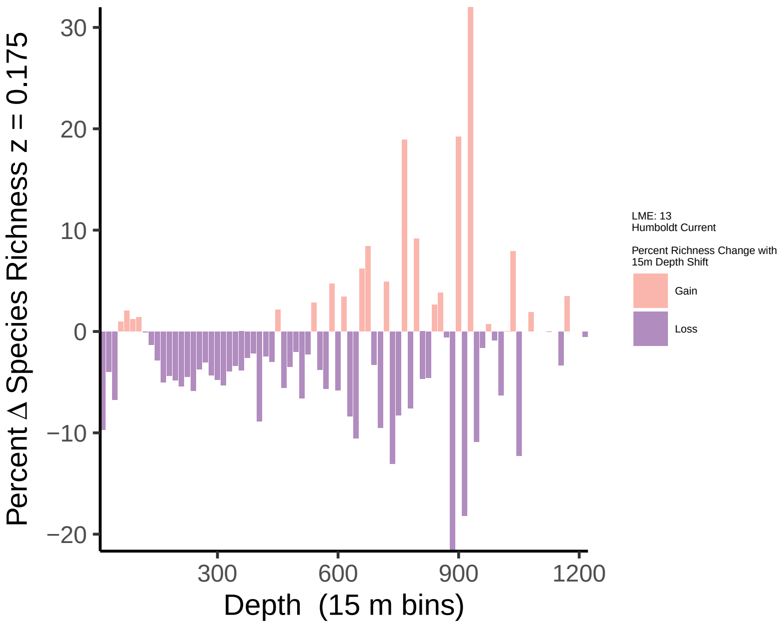

LME 16: East Brazil Shelf

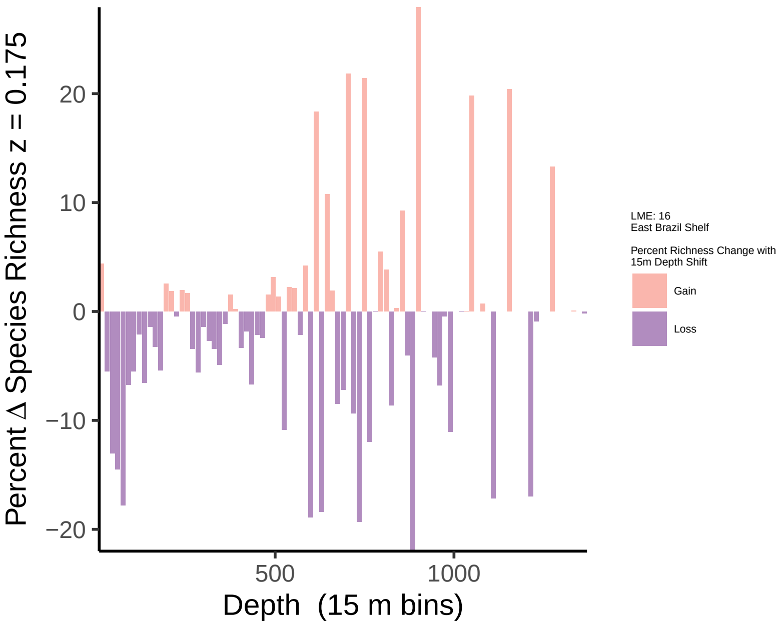

LME 14: Patagonian Shelf

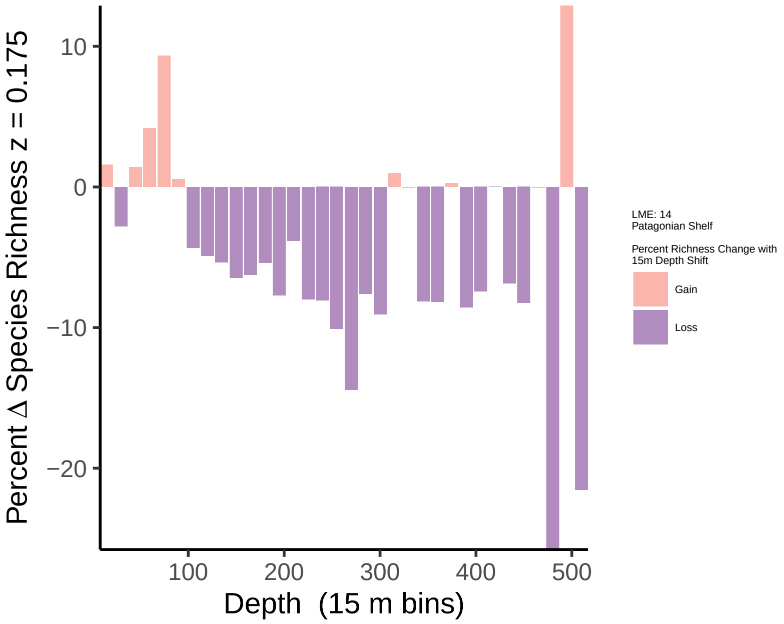

LME 17: North Brazil Shelf

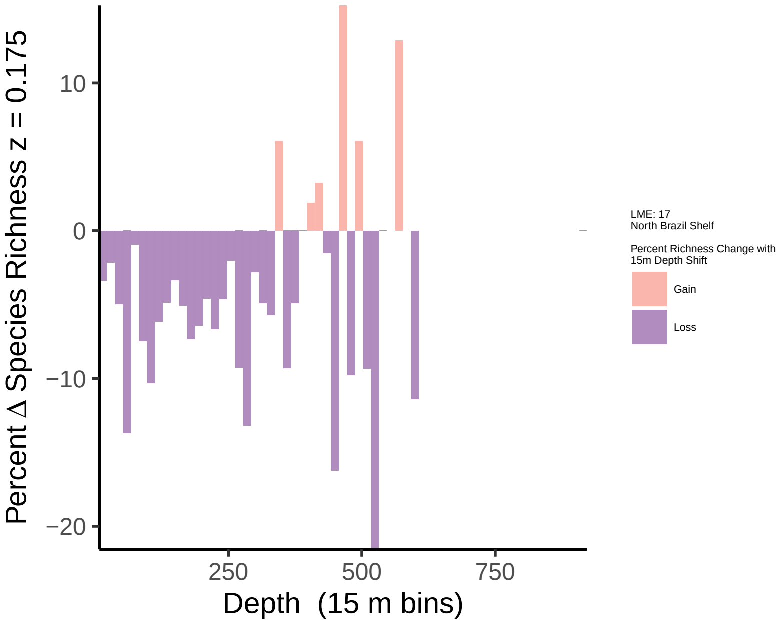

LME 15: South Brazil Shelf

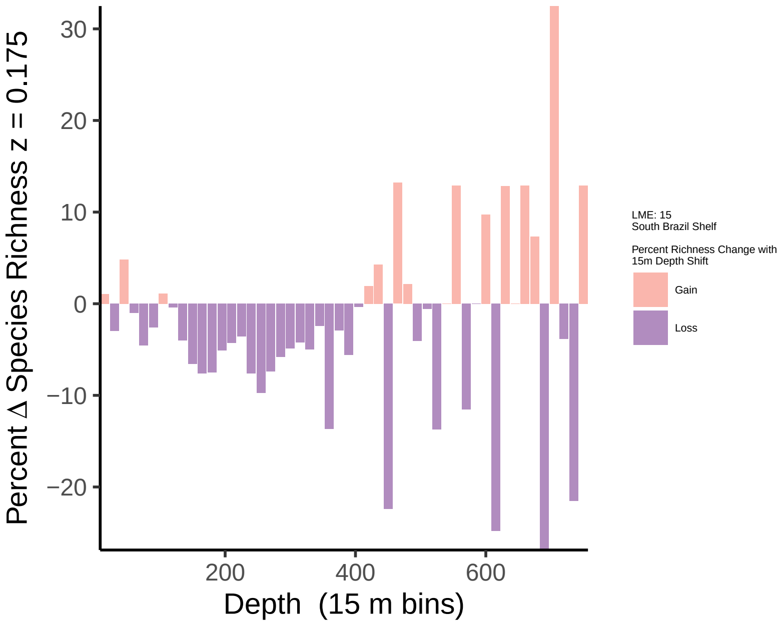

LME 18: Canadian Eastern Arctic − West Gree

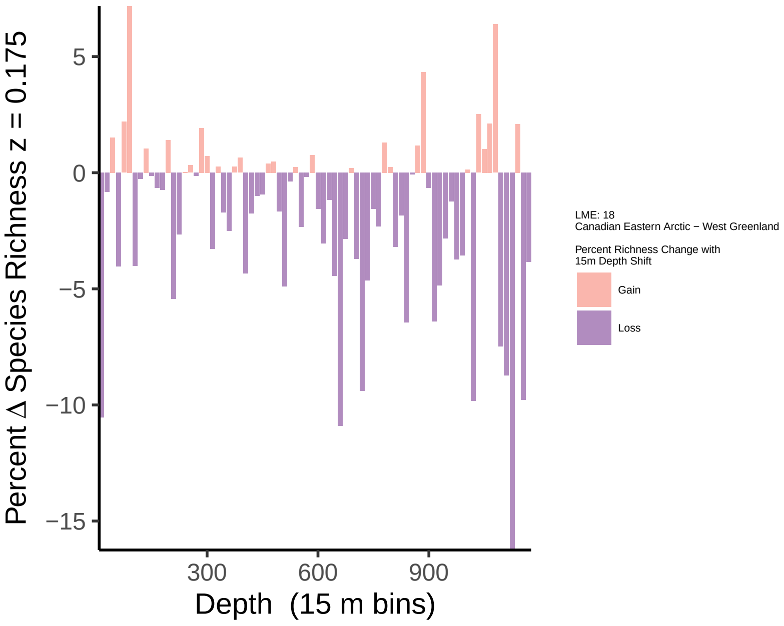

LME 19: Greenland Sea

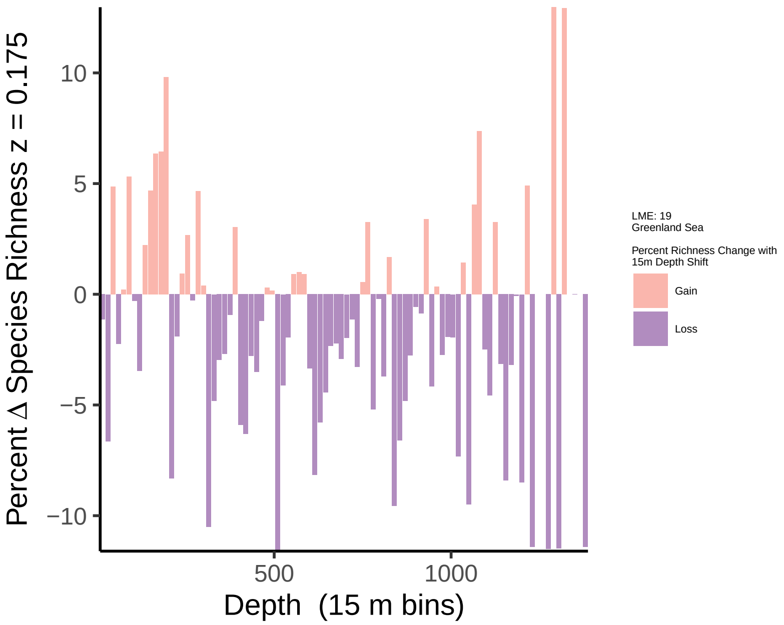

LME 22: North Sea

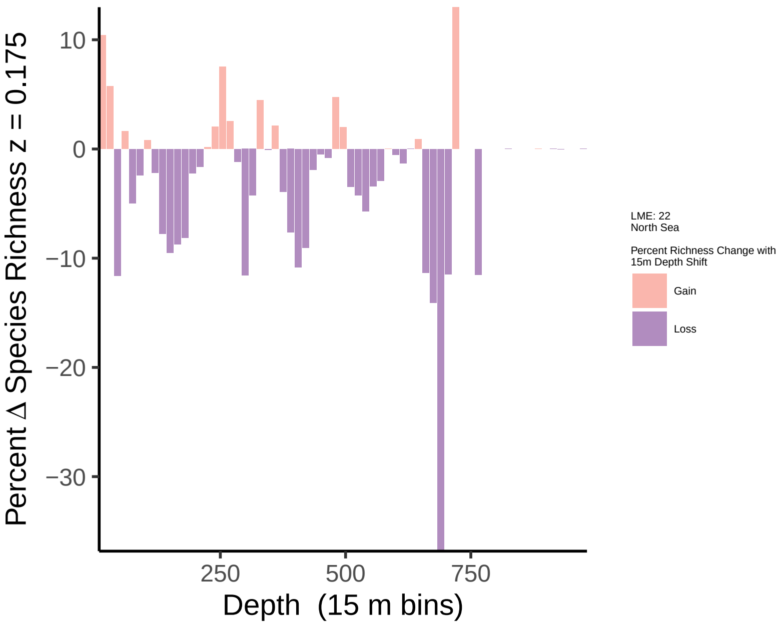

LME 20: Barents Sea

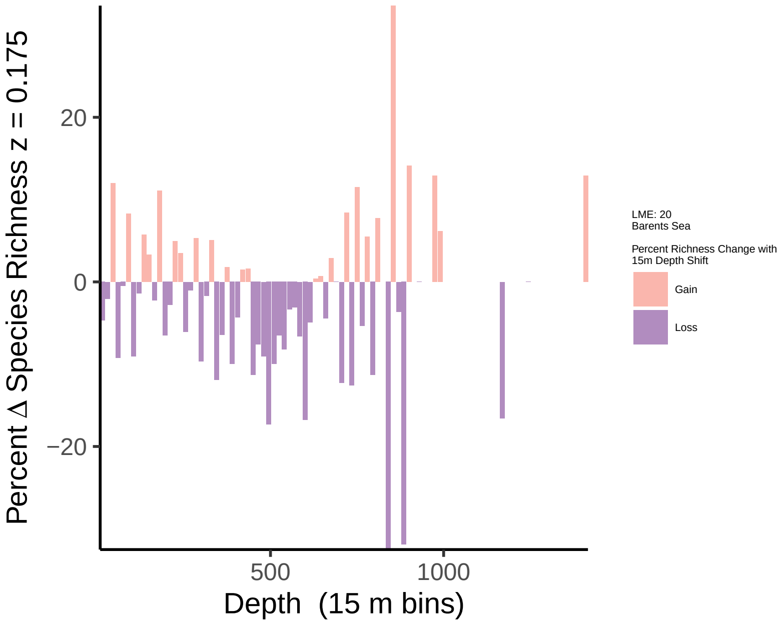

LME 23: Baltic Sea

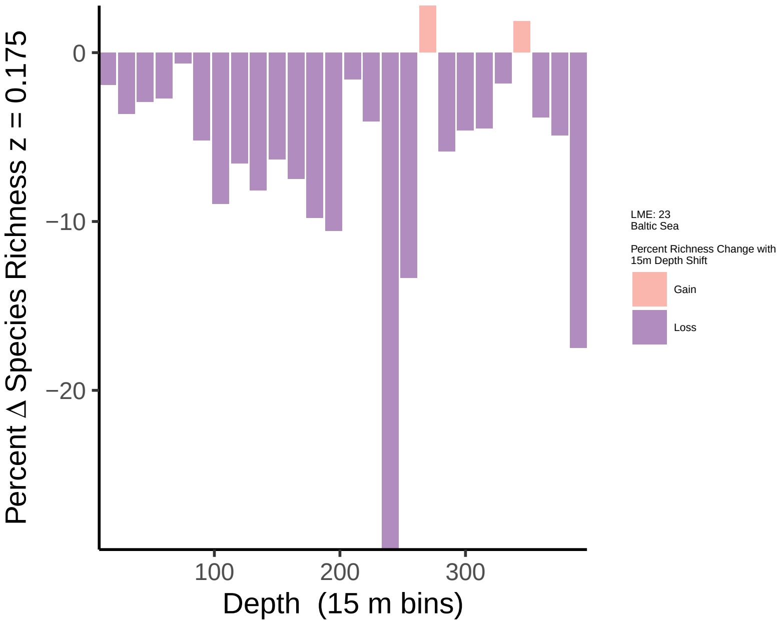

LME 21: Norwegian Sea

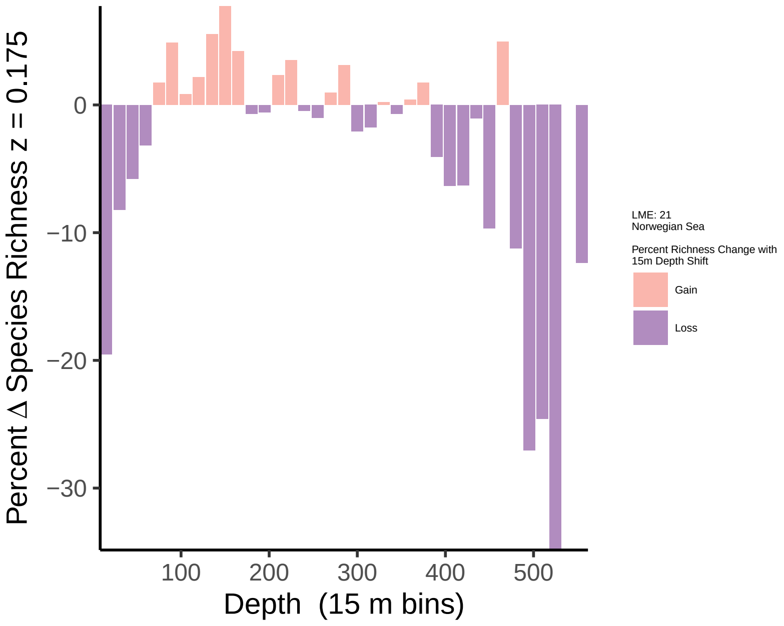

LME 24: Celtic−Biscay Shelf

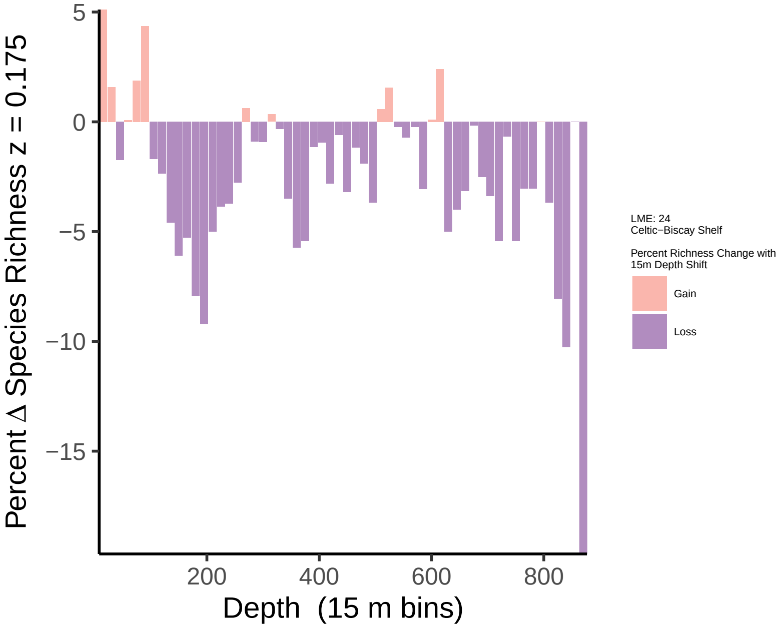

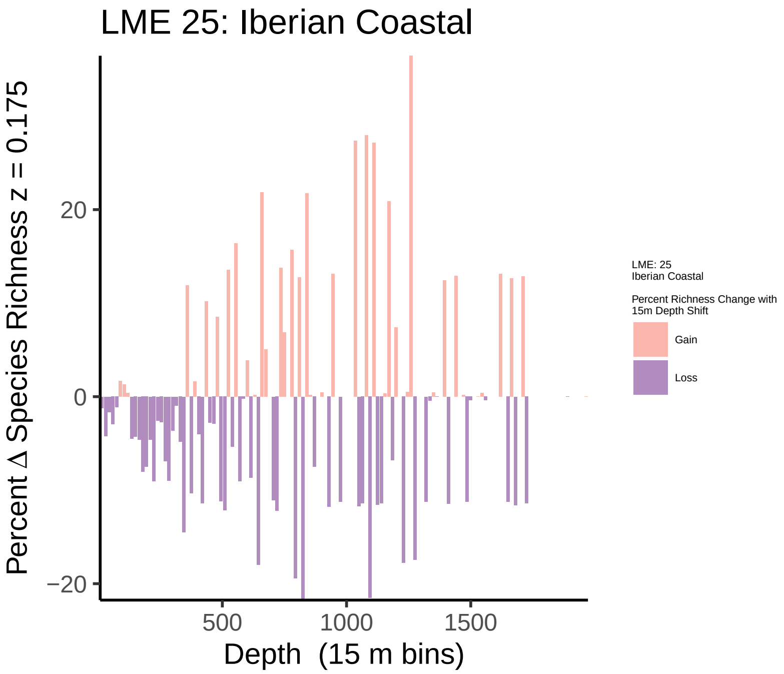
LME 25: Iberian Coastal
Percent Δ Species Richness z = 0.175
20
0
−20
500
1000
1500
Depth (15 m bins)
LME: 25
Iberian Coastal
Percent Richness Change with
15m Depth Shift
Gain
Loss

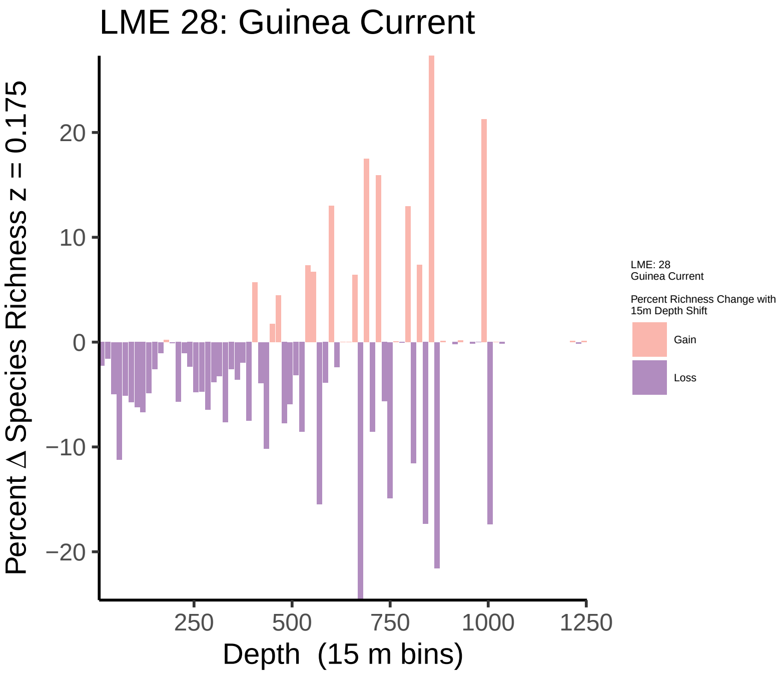
LME 28: Guinea Current
Percent Δ Species Richness z = 0.175
20
10
0
−10
−20
250
500
750
1000
1250
Depth (15 m bins)
LME: 28
Guinea Current
Percent Richness Change with
15m Depth Shift
Gain
Loss

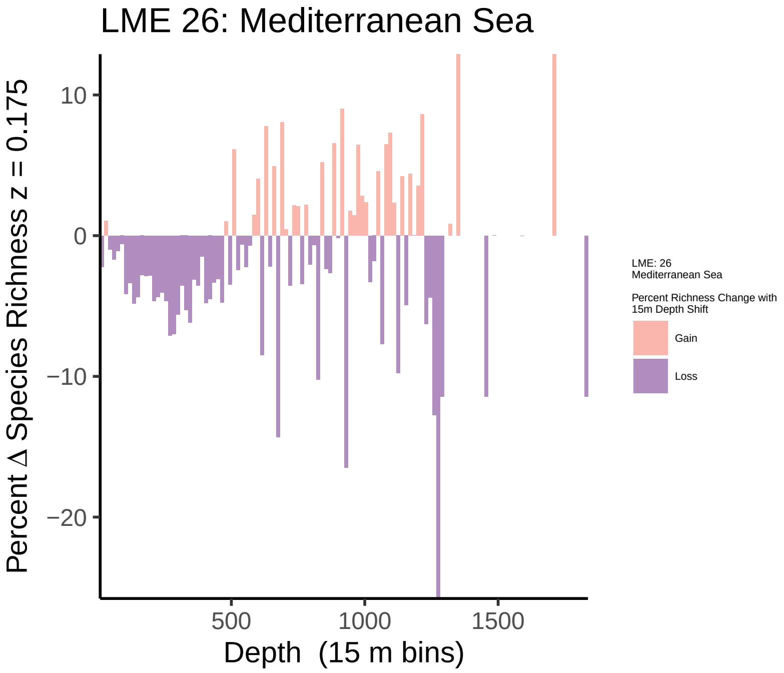
LME 26: Mediterranean Sea
Percent Δ Species Richness z = 0.175
10
0
−10
−20
500
1000
1500
Depth (15 m bins)
LME: 26
Mediterranean Sea
Percent Richness Change with
15m Depth Shift
Gain
Loss

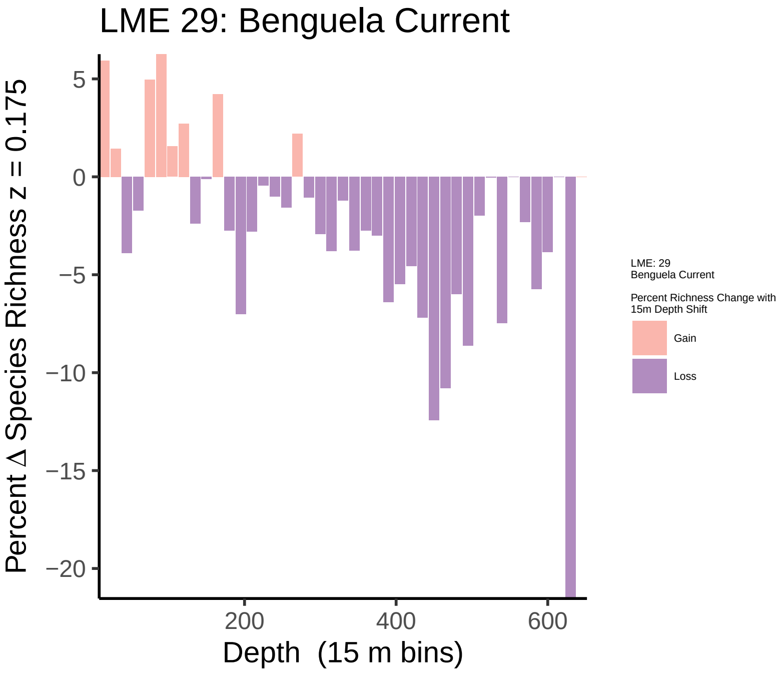
LME 29: Benguela Current
Percent Δ Species Richness z = 0.175
5
0
−5
−10
−15
−20
200
400
600
Depth (15 m bins)
LME: 29
Benguela Current
Percent Richness Change with
15m Depth Shift
Gain
Loss

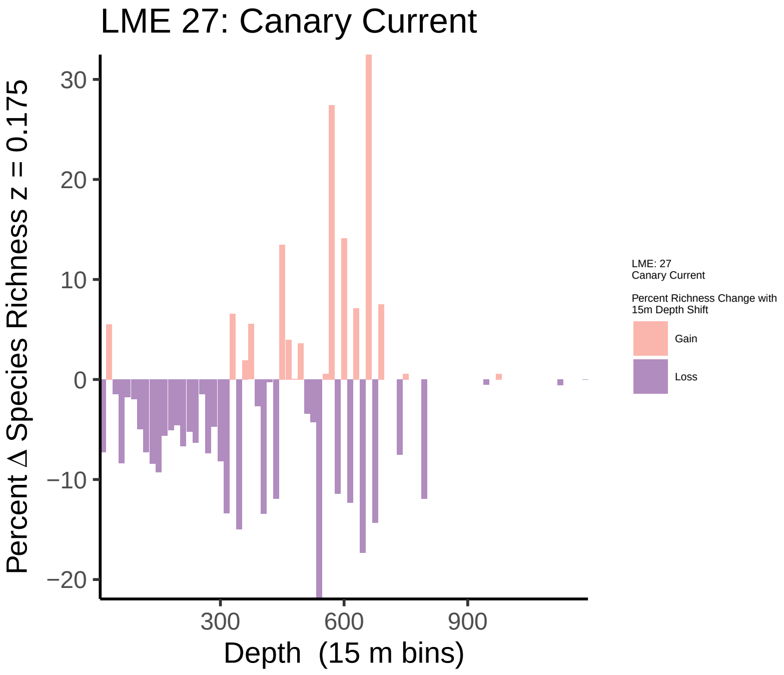
LME 27: Canary Current
Percent Δ Species Richness z = 0.175
30
20
10
0
−10
−20
300
600
900
Depth (15 m bins)
LME: 27
Canary Current
Percent Richness Change with
15m Depth Shift
Gain
Loss

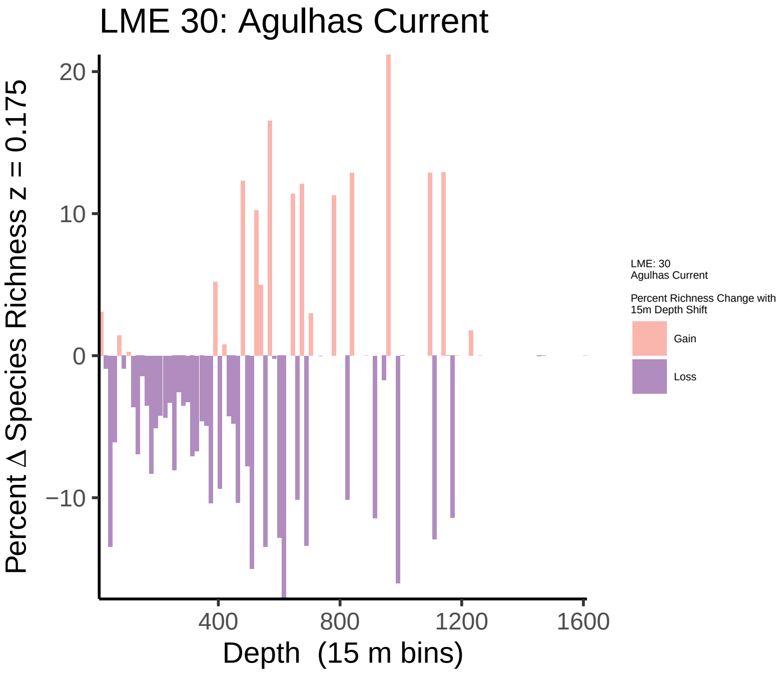
LME 30: Agulhas Current
Percent Δ Species Richness z = 0.175
20
10
0
−10
400
800
1200
1600
Depth (15 m bins)
LME: 30
Agulhas Current
Percent Richness Change with
15m Depth Shift
Gain
Loss

LME 31: Somali Coastal Current

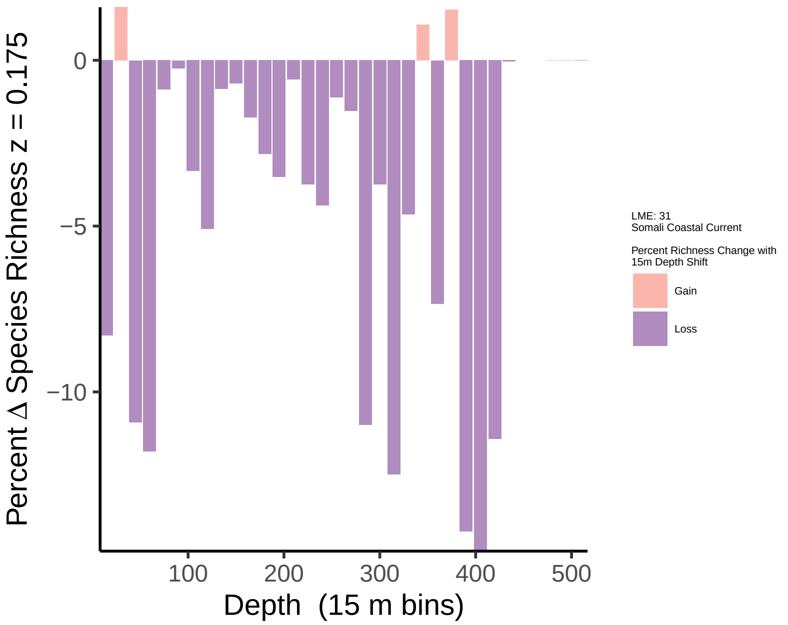

LME 34: Bay of Bengal

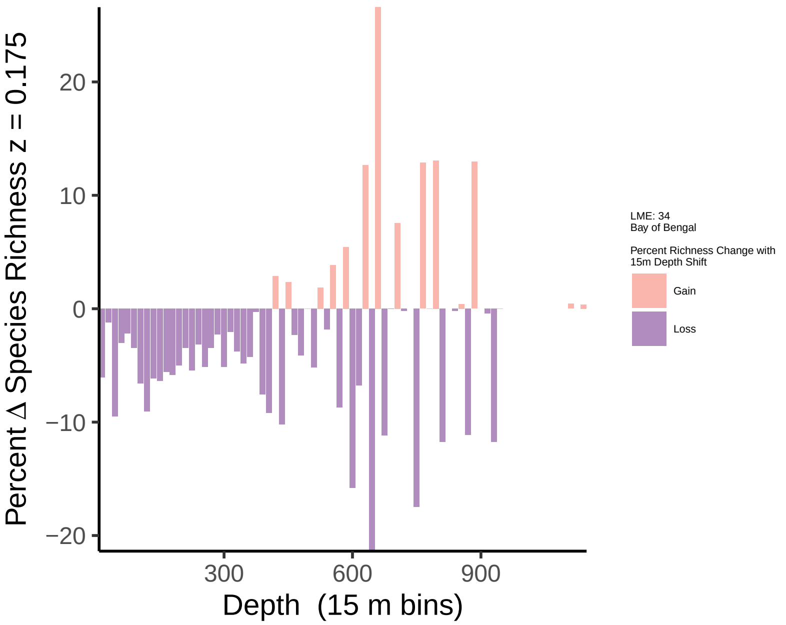

LME 32: Arabian Sea

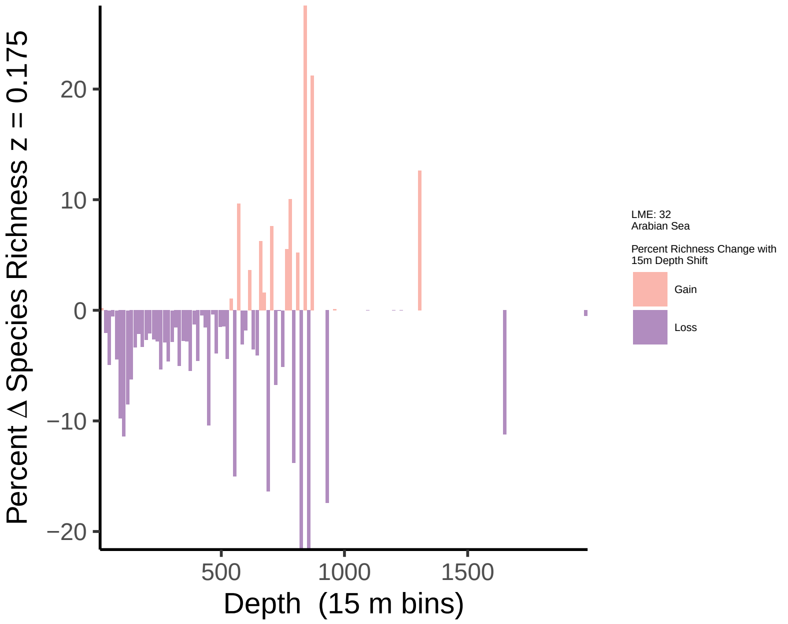

LME 35: Gulf of Thailand

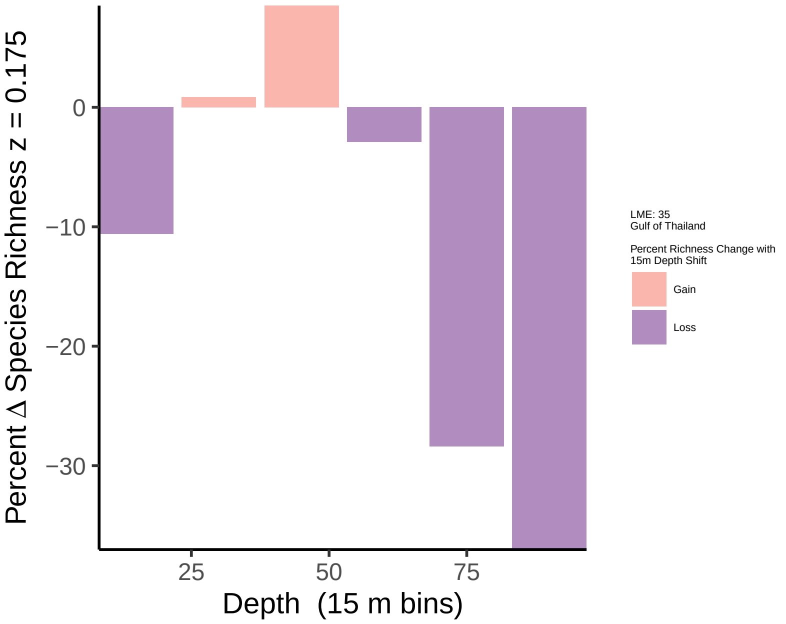

LME 33: Red Sea

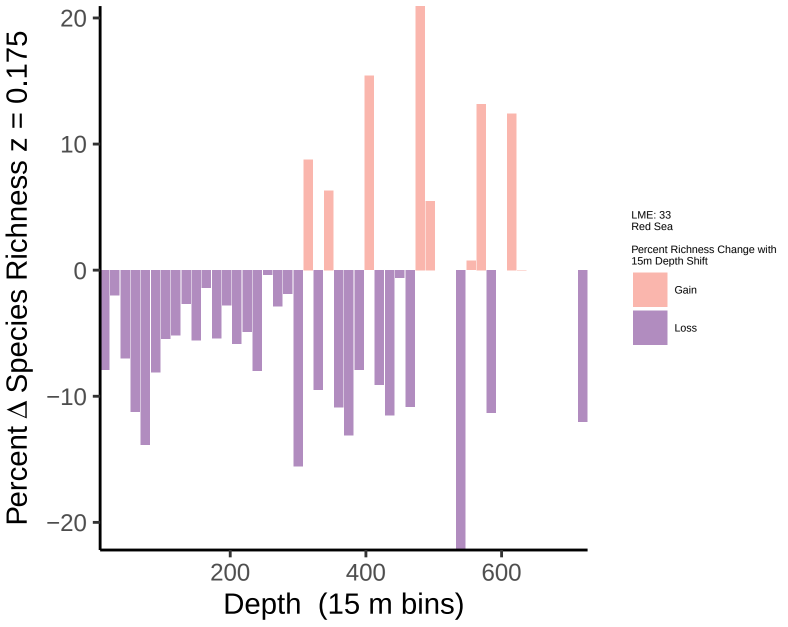

LME 36: South China Sea

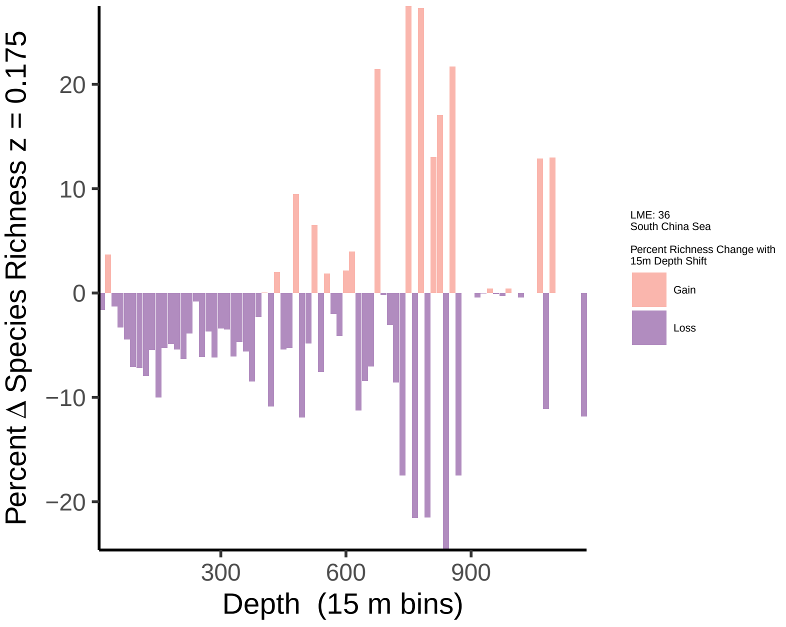

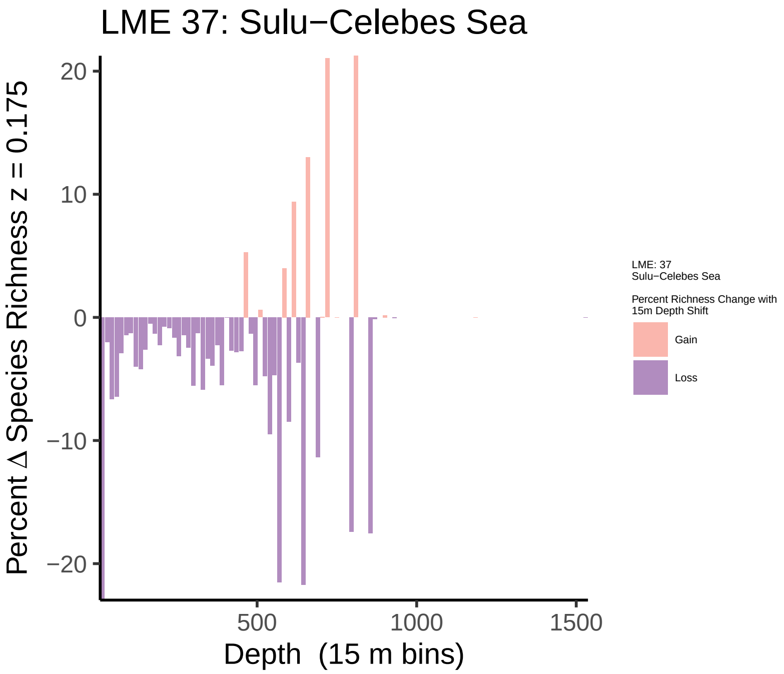
LME 37: Sulu−Celebes Sea
Percent Δ Species Richness z = 0.175
20
10
0
−10
−20
500
1000
1500
Depth (15 m bins)
LME: 37
Sulu−Celebes Sea
Percent Richness Change with
15m Depth Shift
Gain
Loss

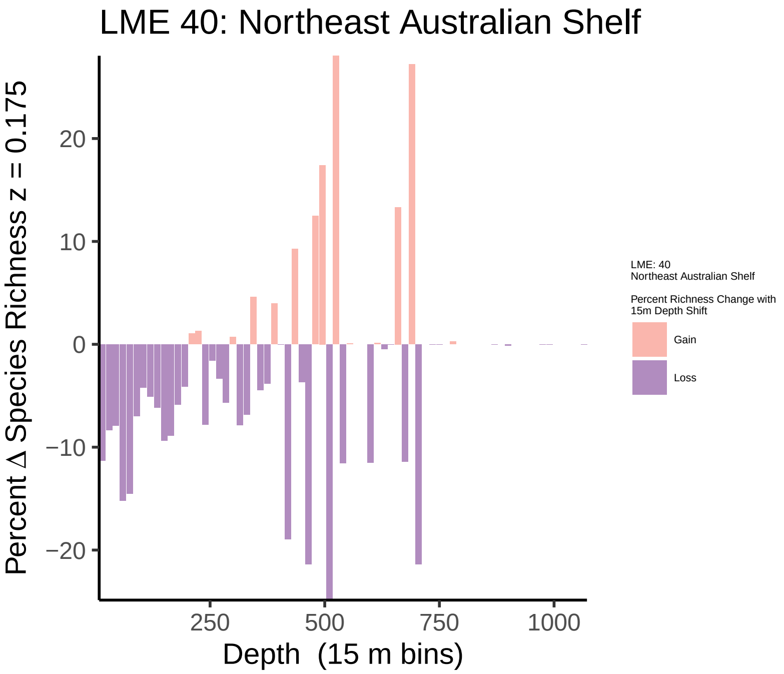
LME 40: Northeast Australian Shelf
Percent Δ Species Richness z = 0.175
20
10
0
−10
−20
250
500
750
1000
Depth (15 m bins)
LME: 40
Northeast Australian Shelf
Percent Richness Change with
15m Depth Shift
Gain
Loss

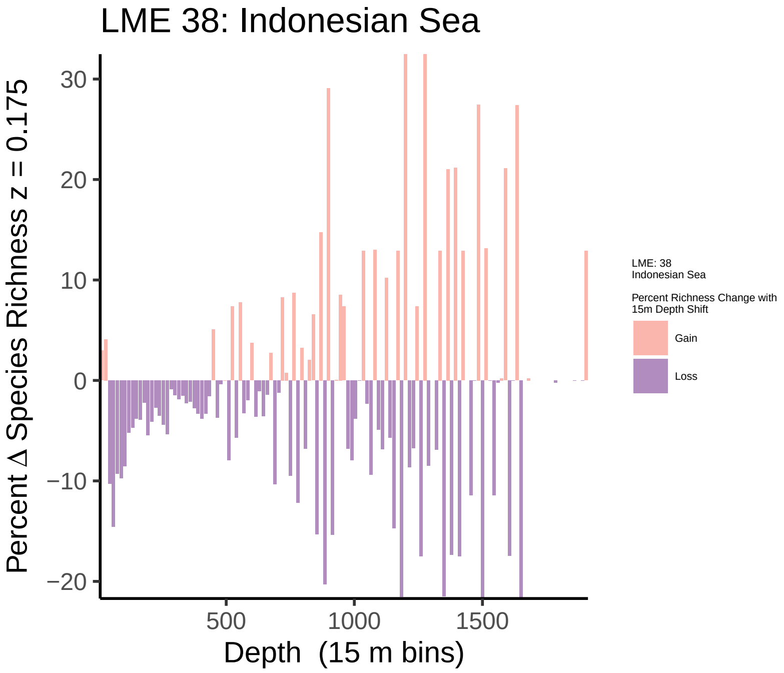
LME 38: Indonesian Sea
Percent Δ Species Richness z = 0.175
30
20
10
0
−10
−20
500
1000
1500
Depth (15 m bins)
LME: 38
Indonesian Sea
Percent Richness Change with
15m Depth Shift
Gain
Loss

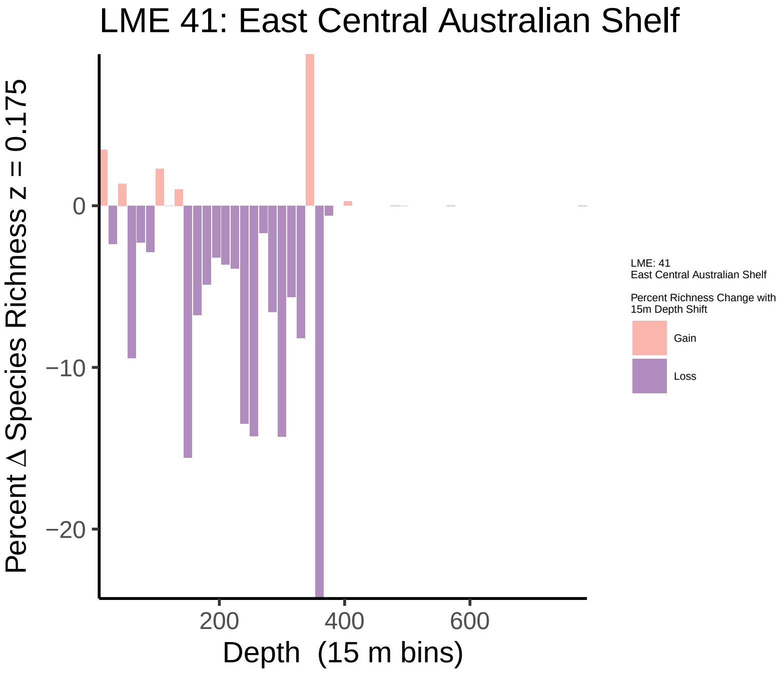
LME 41: East Central Australian Shelf
Percent Δ Species Richness z = 0.175
0
−10
−20
200
400
600
Depth (15 m bins)
LME: 41
East Central Australian Shelf
Percent Richness Change with
15m Depth Shift
Gain
Loss

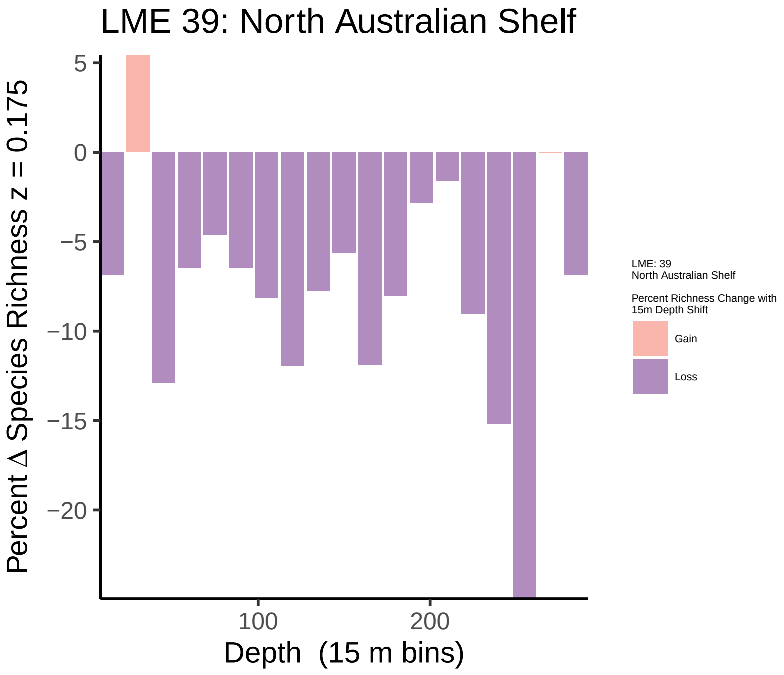
LME 39: North Australian Shelf
Percent Δ Species Richness z = 0.175
5
0
−5
−10
−15
−20
100
200
Depth (15 m bins)
LME: 39
North Australian Shelf
Percent Richness Change with
15m Depth Shift
Gain
Loss

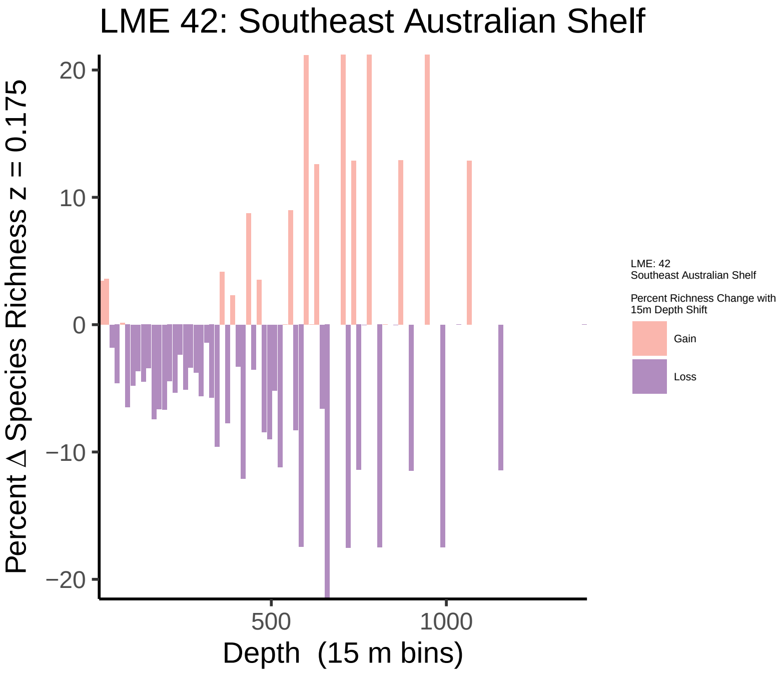
LME 42: Southeast Australian Shelf
Percent Δ Species Richness z = 0.175
20
10
0
−10
−20
500
1000
Depth (15 m bins)
LME: 42
Southeast Australian Shelf
Percent Richness Change with
15m Depth Shift
Gain
Loss

LME 43: South West Australian Shelf

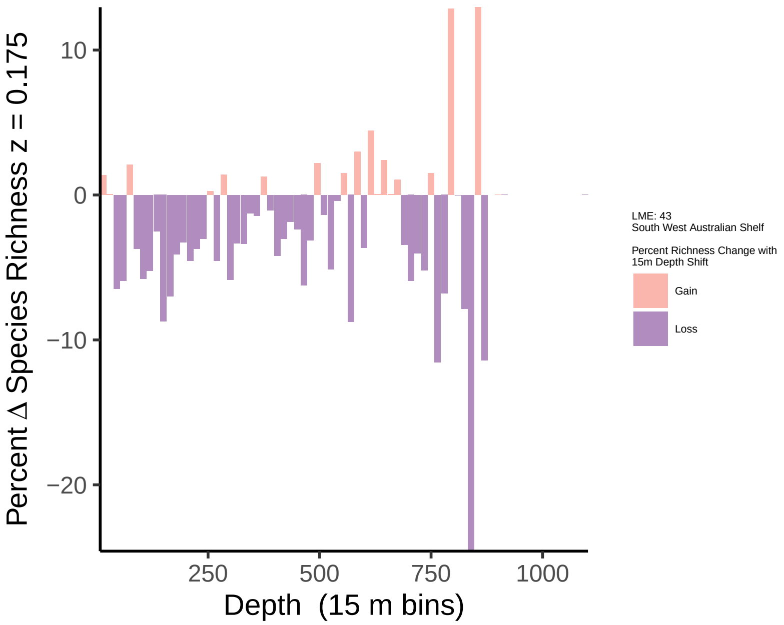

LME 46: New Zealand Shelf

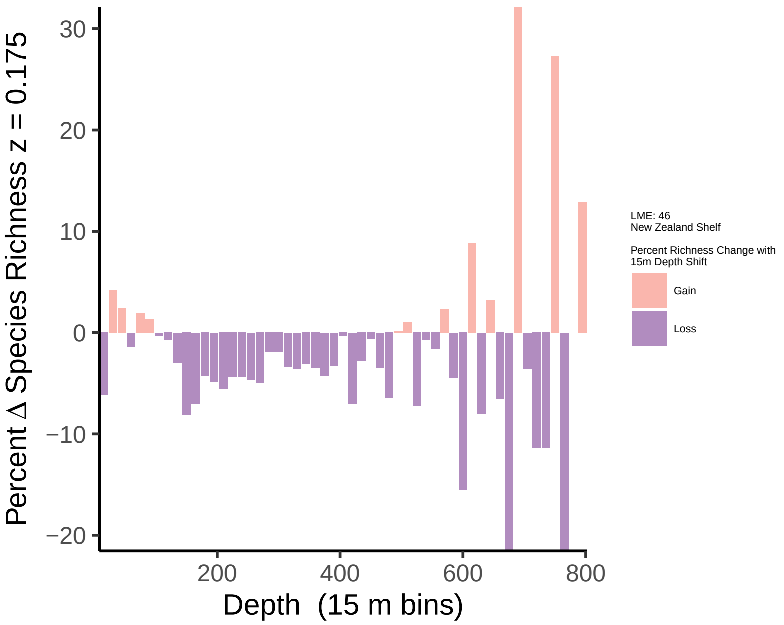

LME 44: West Central Australian Shelf

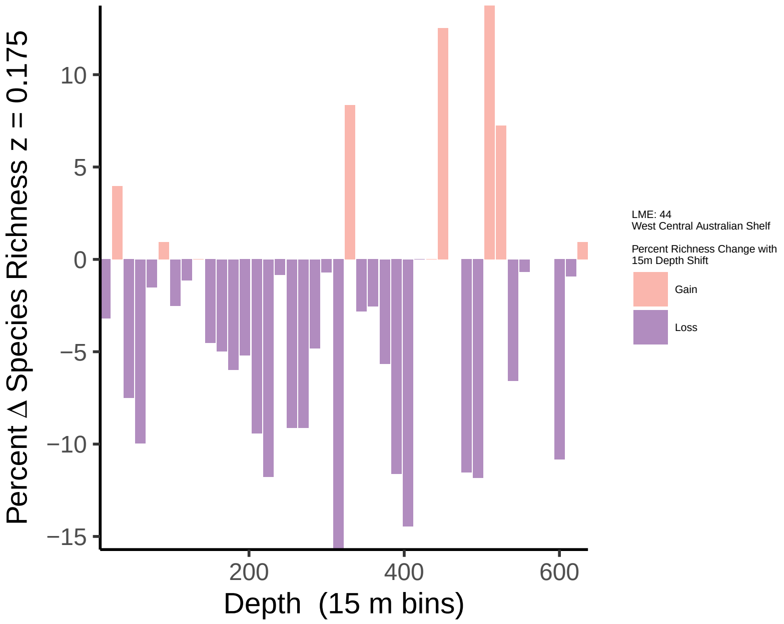

LME 47: East China Sea

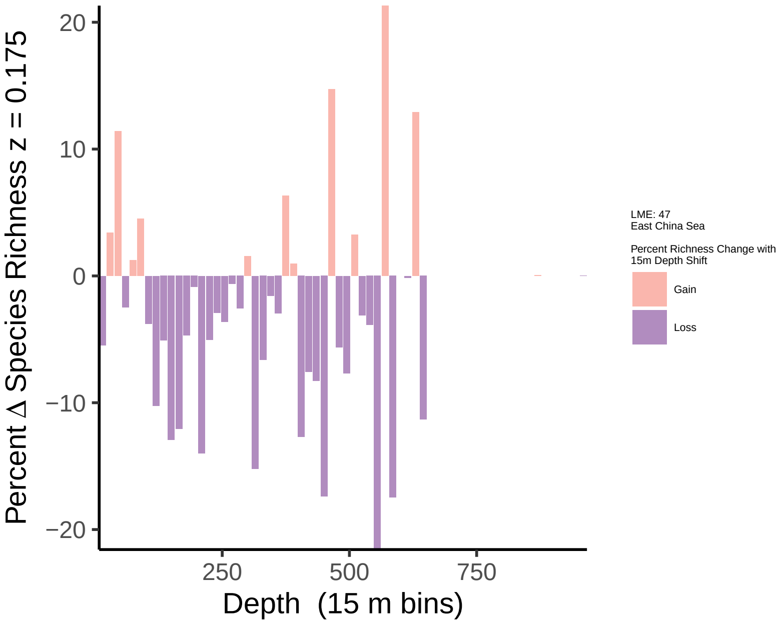

LME 45: Northwest Australian Shelf

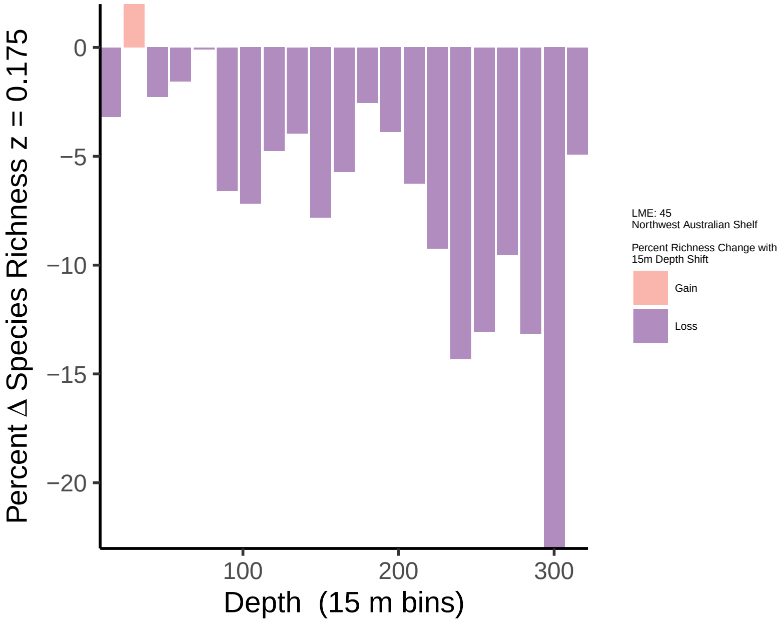

LME 48: Yellow Sea

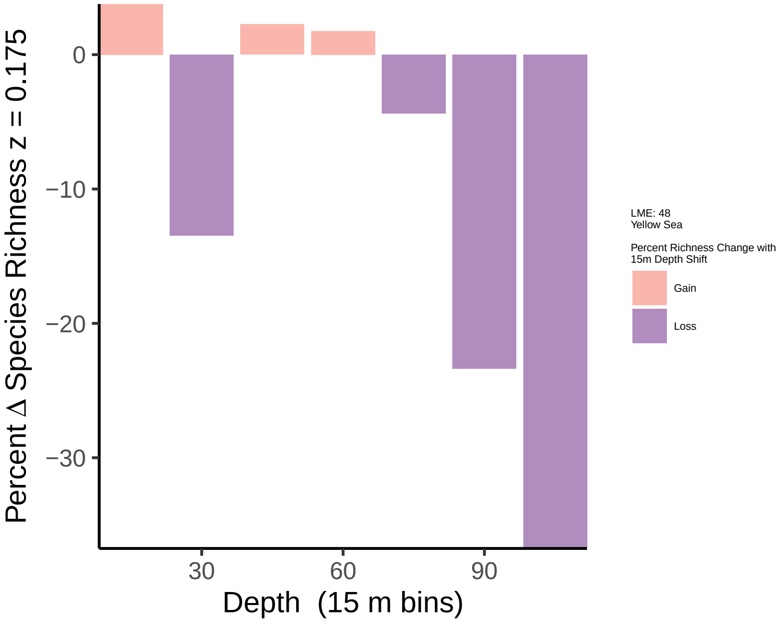

LME 49: Kuroshio Current

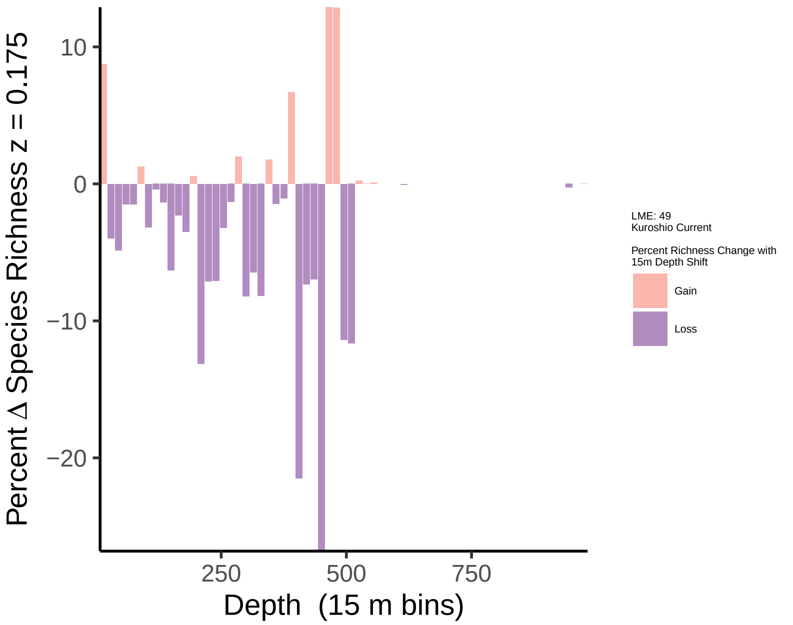

LME 52: Sea of Okhotsk

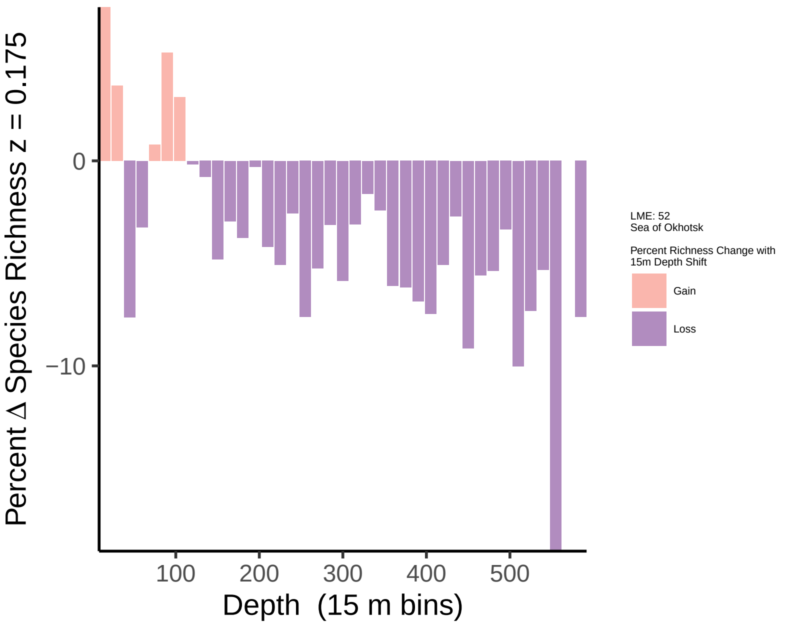

LME 50: Sea of Japan

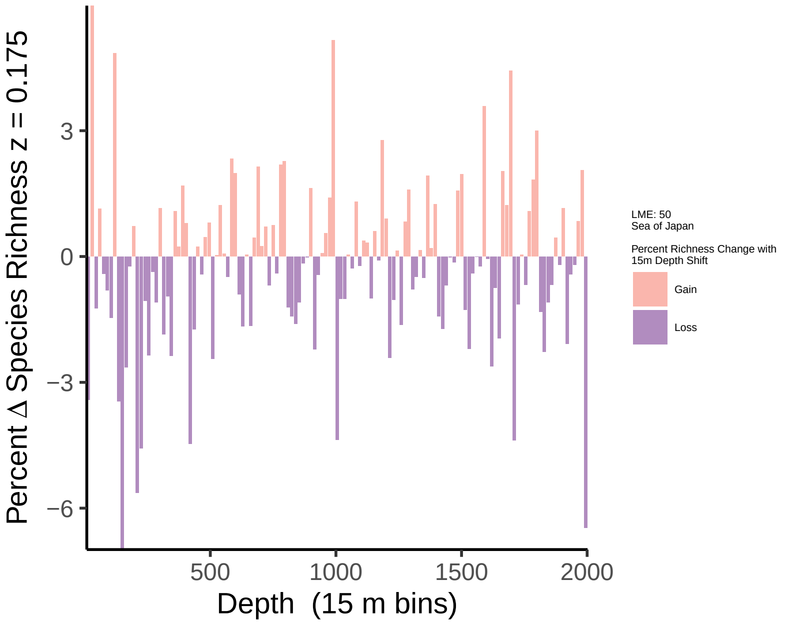

LME 53: West Bering Sea

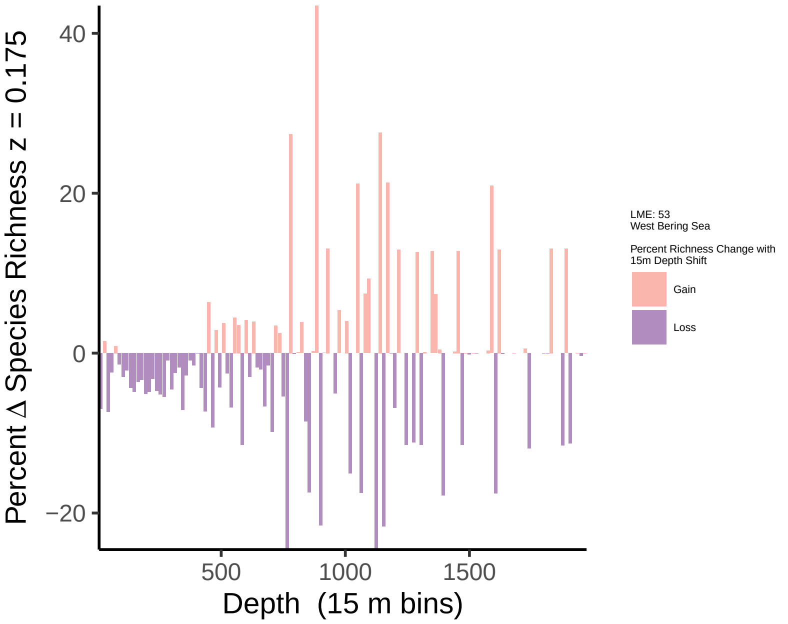

LME 51: Oyashio Current

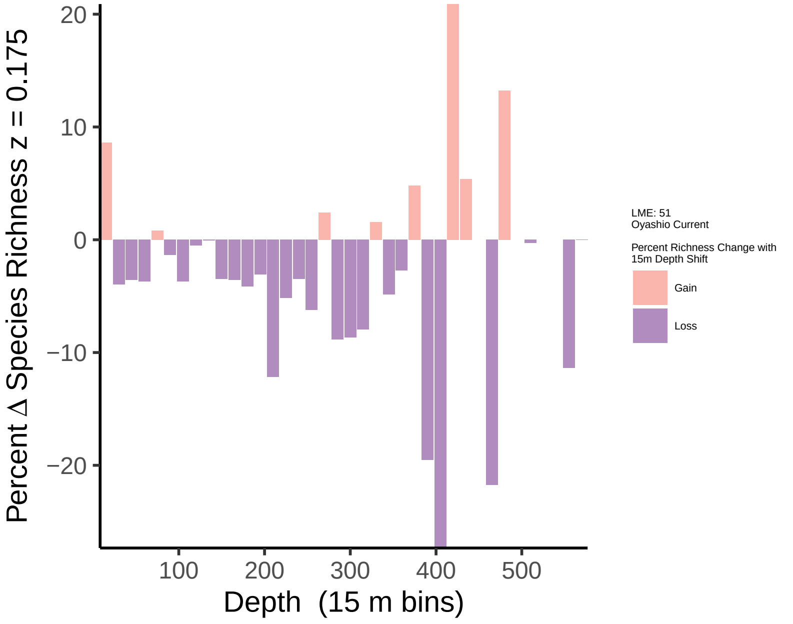

LME 54: Northern Bering − Chukchi Seas

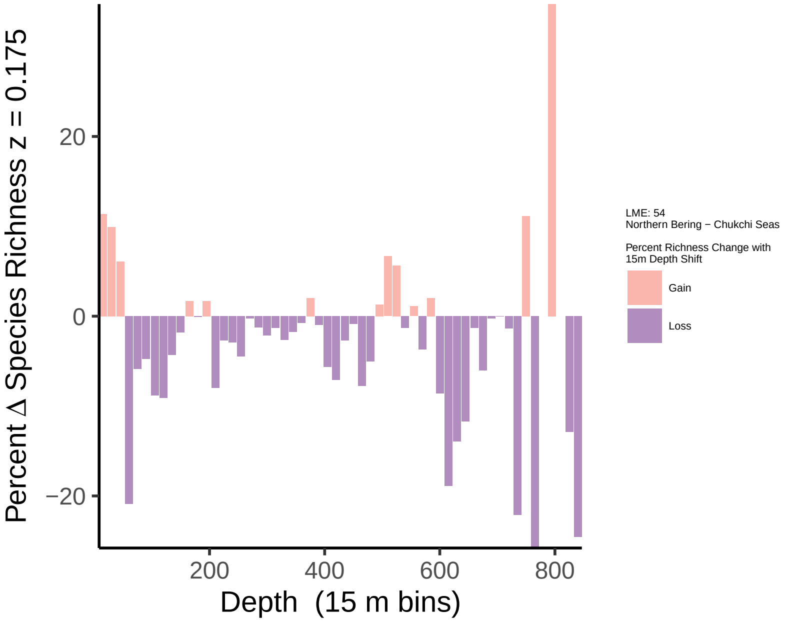

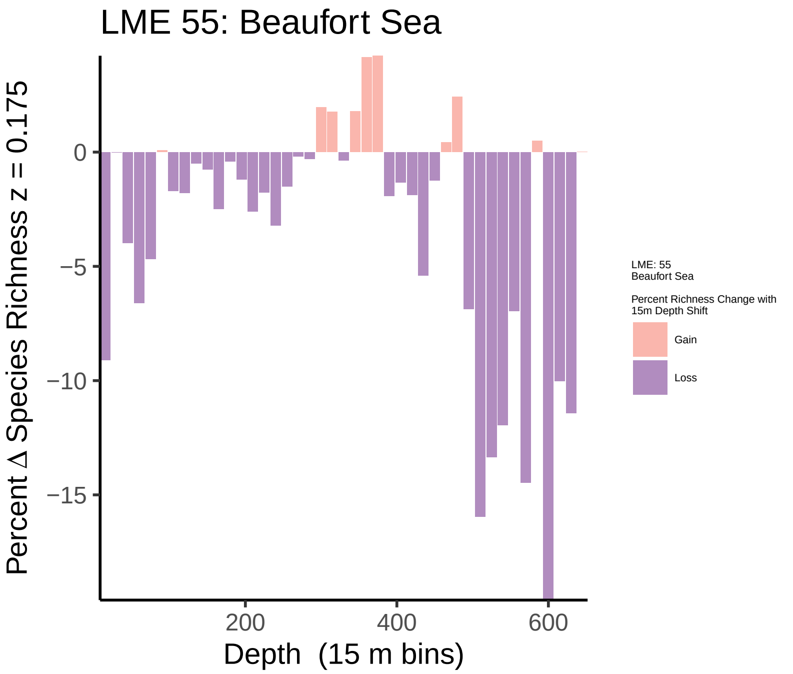
LME 55: Beaufort Sea
Percent Δ Species Richness z = 0.175
0
−5
−10
−15
200
400
600
Depth (15 m bins)
LME: 55
Beaufort Sea
Percent Richness Change with
15m Depth Shift
Gain
Loss

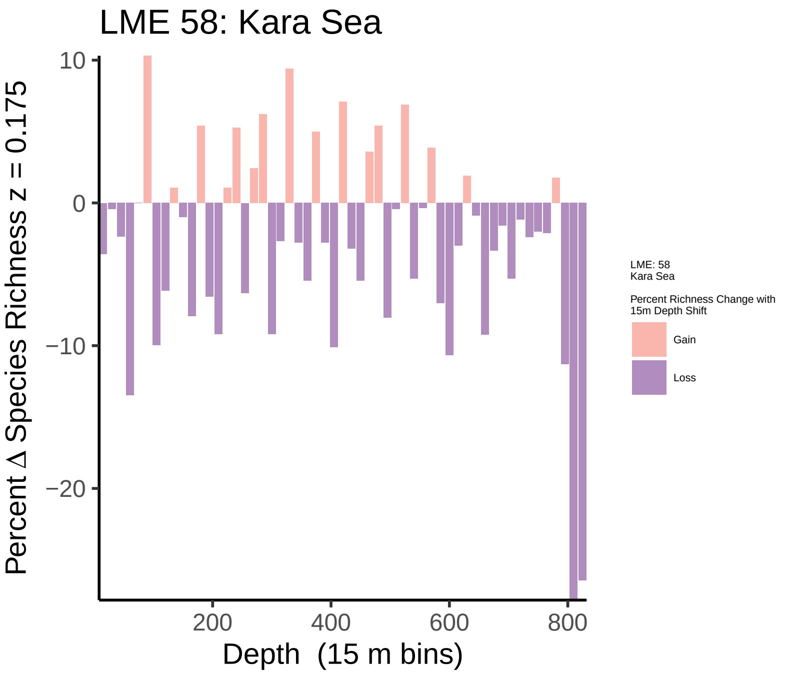
LME 58: Kara Sea
Percent Δ Species Richness z = 0.175
10
0
−10
−20
200
400
600
800
Depth (15 m bins)
LME: 58
Kara Sea
Percent Richness Change with
15m Depth Shift
Gain
Loss

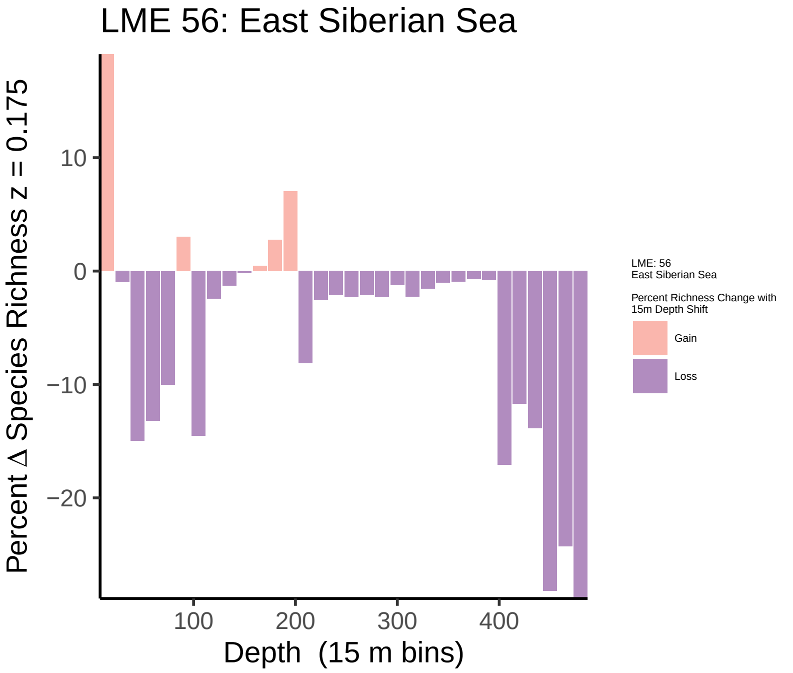
LME 56: East Siberian Sea
Percent Δ Species Richness z = 0.175
10
0
−10
−20
100
200
300
400
Depth (15 m bins)
LME: 56
East Siberian Sea
Percent Richness Change with
15m Depth Shift
Gain
Loss

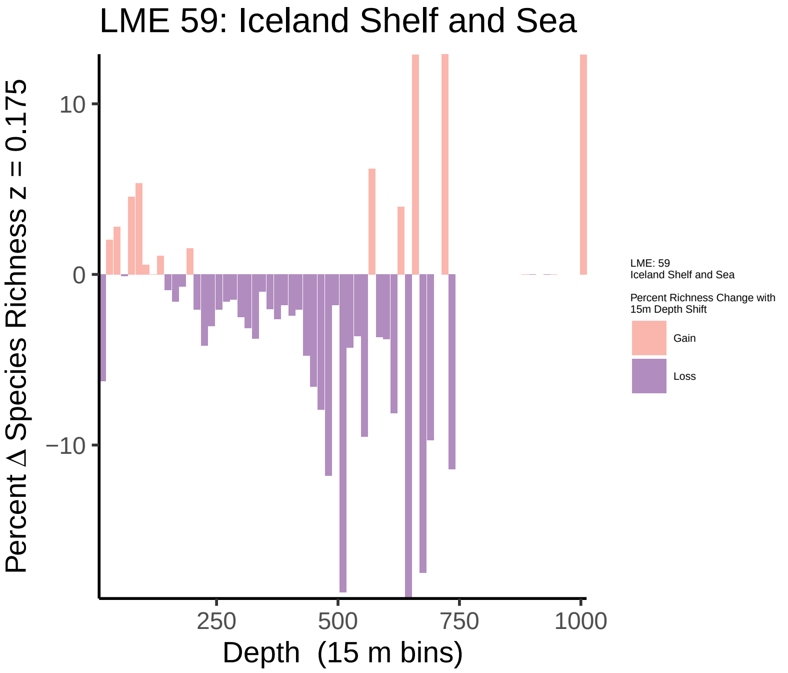
LME 59: Iceland Shelf and Sea
Percent Δ Species Richness z = 0.175
10
0
−10
250
500
750
1000
Depth (15 m bins)
LME: 59
Iceland Shelf and Sea
Percent Richness Change with
15m Depth Shift
Gain
Loss

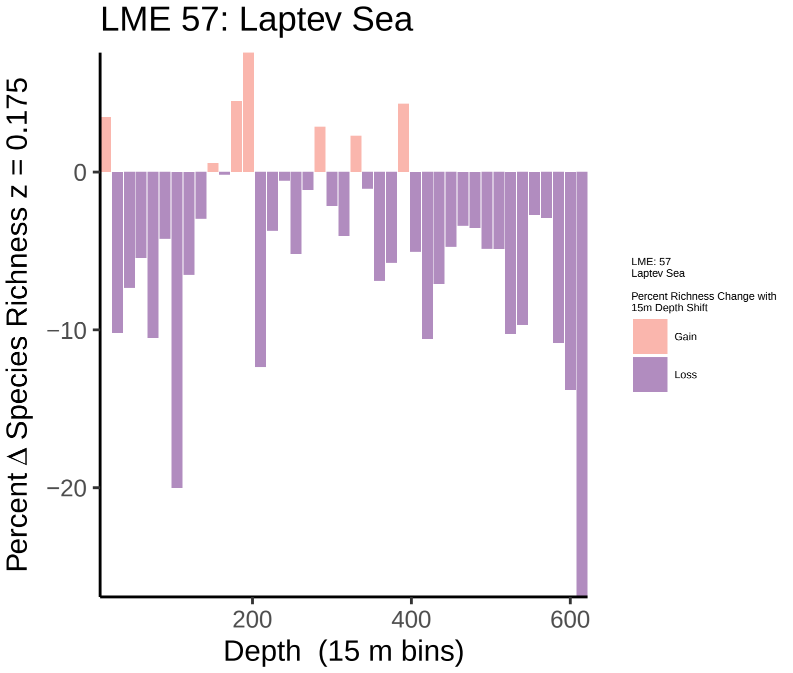
LME 57: Laptev Sea
Percent Δ Species Richness z = 0.175
0
−10
−20
200
400
600
Depth (15 m bins)
LME: 57
Laptev Sea
Percent Richness Change with
15m Depth Shift
Gain
Loss

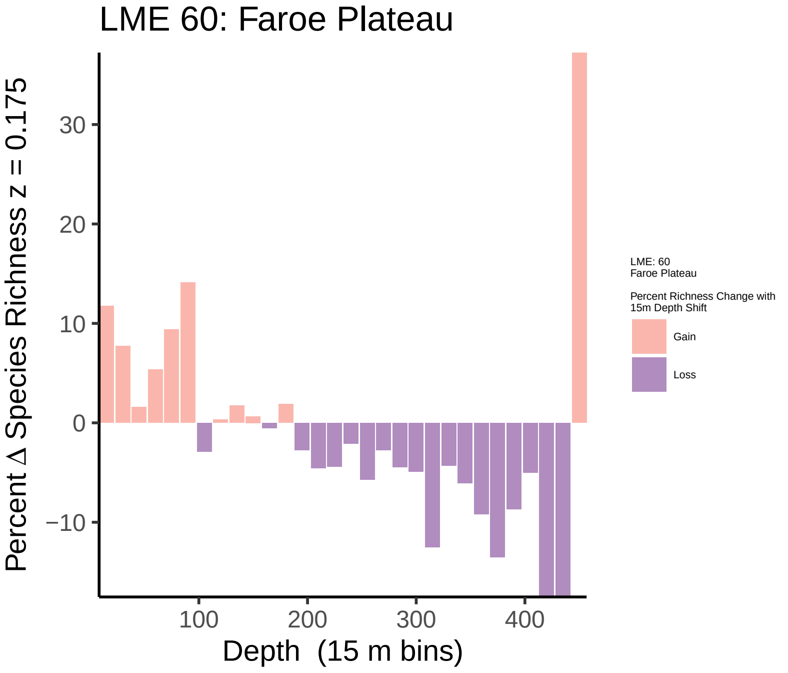
LME 60: Faroe Plateau
Percent Δ Species Richness z = 0.175
30
20
10
0
−10
100
200
300
400
Depth (15 m bins)
LME: 60
Faroe Plateau
Percent Richness Change with
15m Depth Shift
Gain
Loss

LME 62: Black Sea

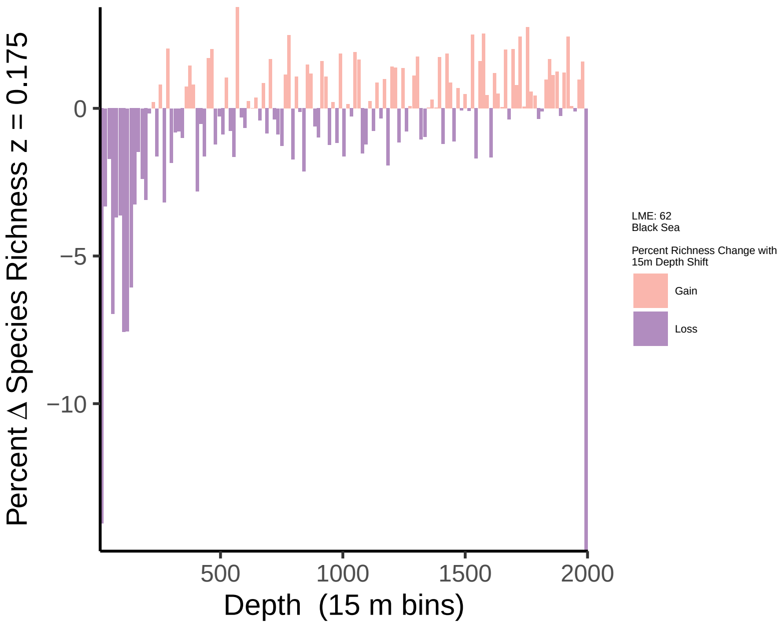

LME 66: Canadian High Arctic − North Greenl

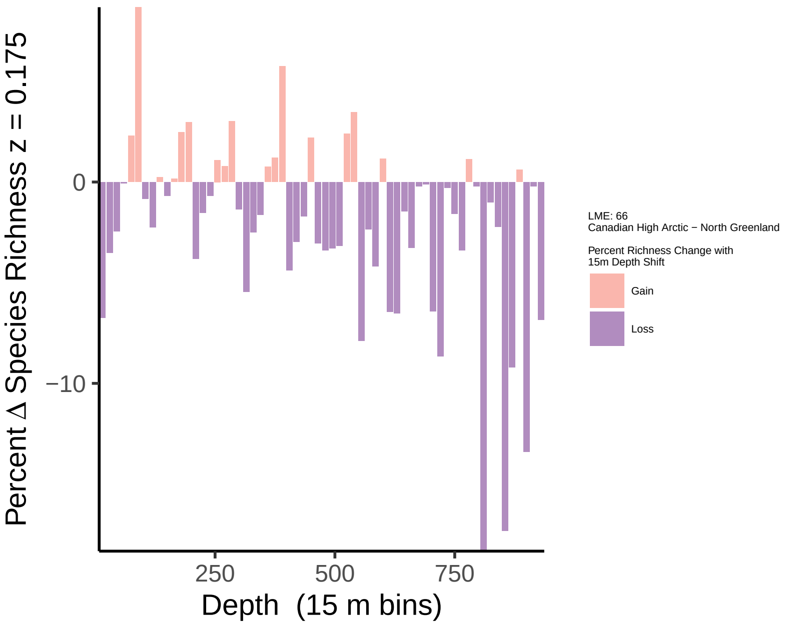

LME 63: Hudson Bay Complex

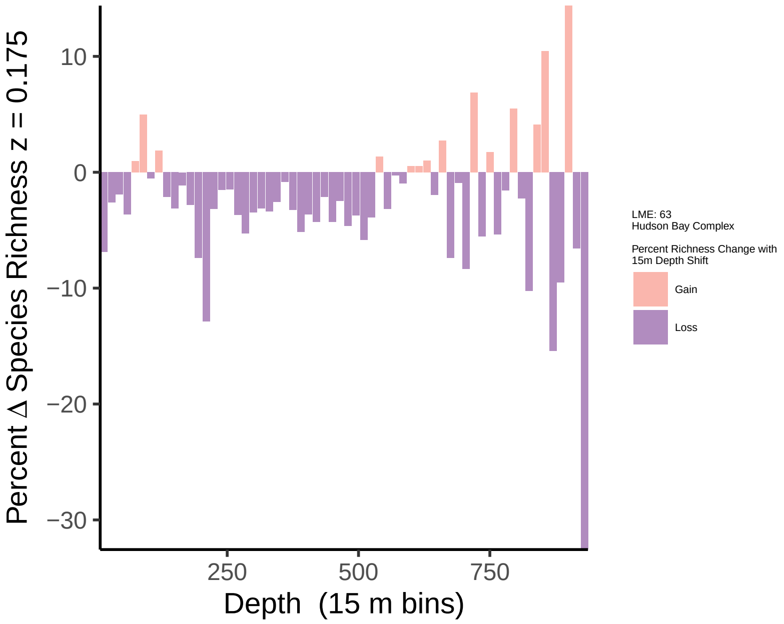

LME 65: Aleutian Islands

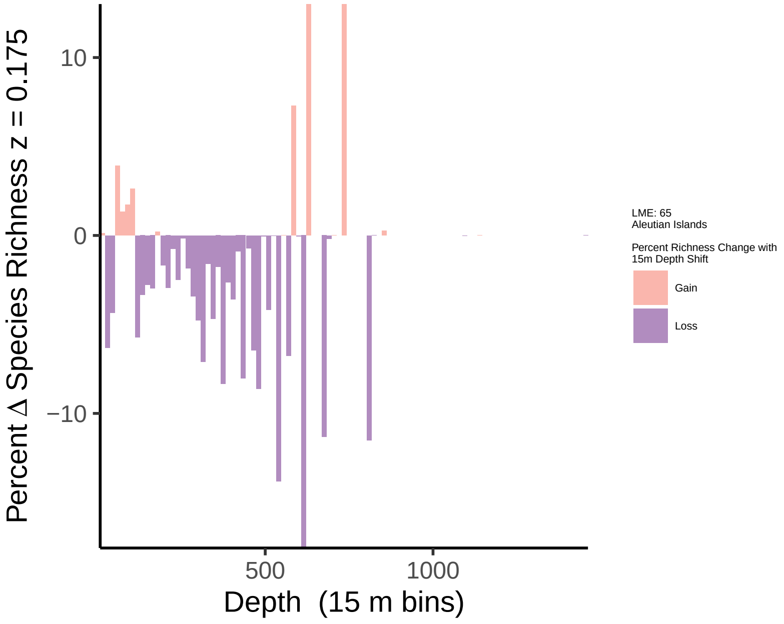

Fig S10 b.

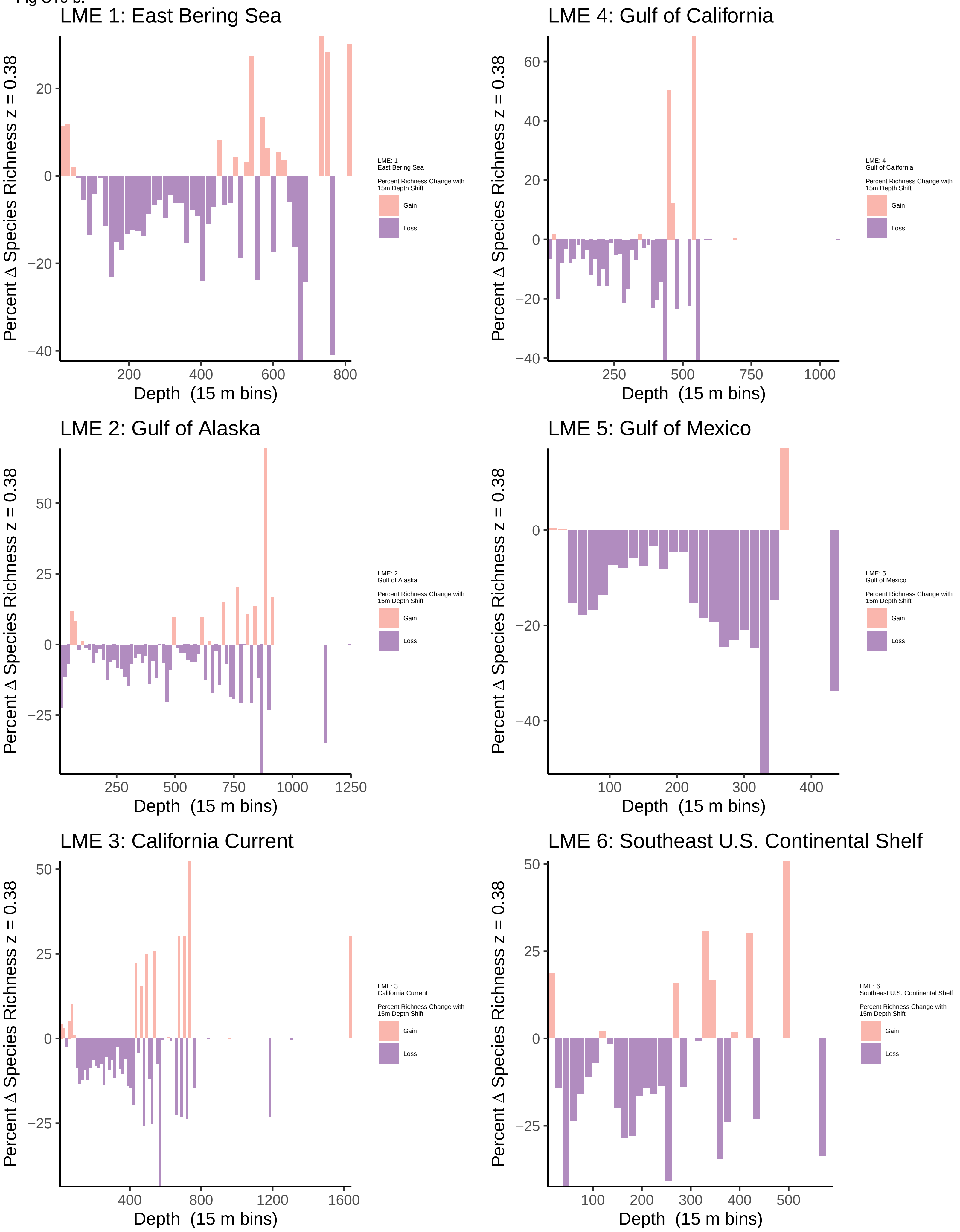
LME 1: East Bering Sea
Percent Δ Species Richness z = 0.38
Depth (15 m bins)
LME: 1
East Bering Sea
Percent Richness Change with
15m Depth Shift
Gain
Loss
LME 4: Gulf of California
Percent Δ Species Richness z = 0.38
Depth (15 m bins)
LME: 4
Gulf of California
Percent Richness Change with
15m Depth Shift
Gain
Loss
LME 2: Gulf of Alaska
Percent Δ Species Richness z = 0.38
Depth (15 m bins)
LME: 2
Gulf of Alaska
Percent Richness Change with
15m Depth Shift
Gain
Loss
LME 5: Gulf of Mexico
Percent Δ Species Richness z = 0.38
Depth (15 m bins)
LME: 5
Gulf of Mexico
Percent Richness Change with
15m Depth Shift
Gain
Loss
LME 3: California Current
Percent Δ Species Richness z = 0.38
Depth (15 m bins)
LME: 3
California Current
Percent Richness Change with
15m Depth Shift
Gain
Loss
LME 6: Southeast U.S. Continental Shelf
Percent Δ Species Richness z = 0.38
Depth (15 m bins)
LME: 6
Southeast U.S. Continental Shelf
Percent Richness Change with
15m Depth Shift
Gain
Loss

LME 7: Northeast U.S. Continental Shelf

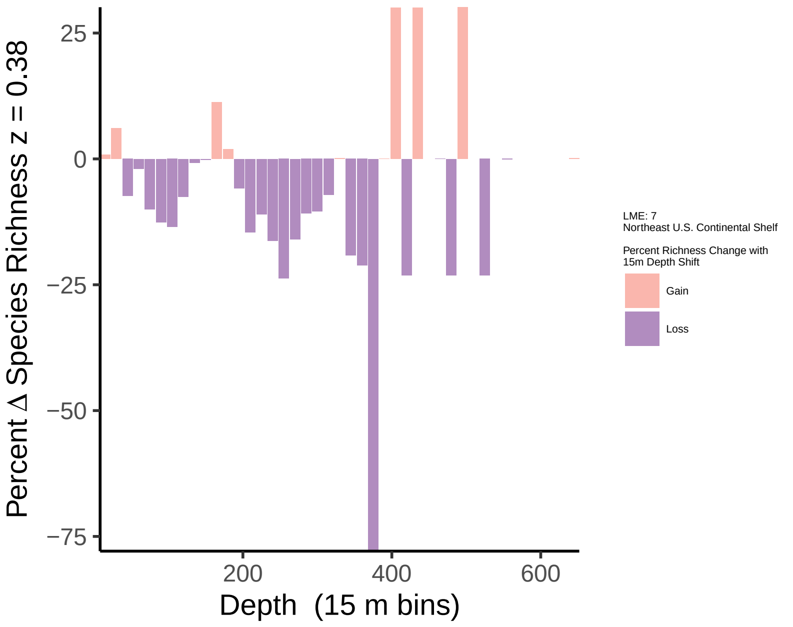

LME 10: Insular Pacific−Hawaiian

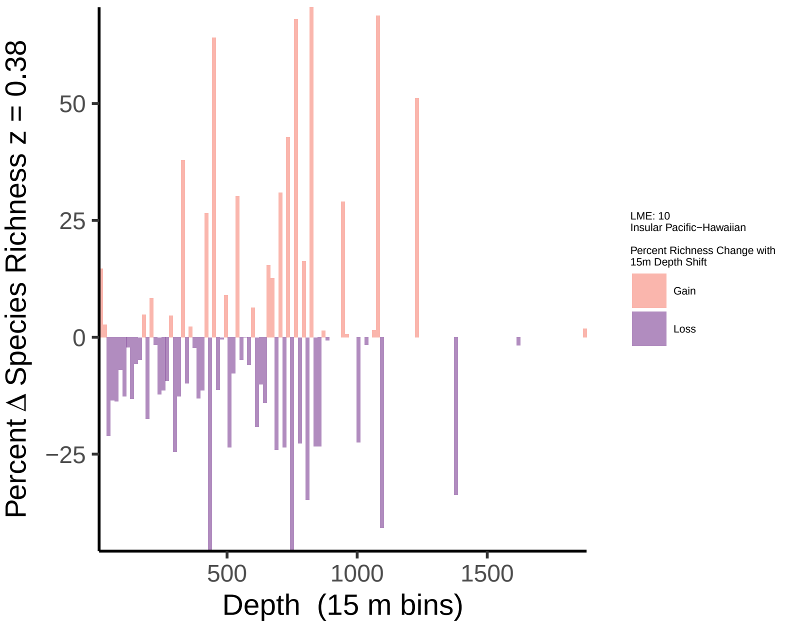

LME 8: Scotian Shelf

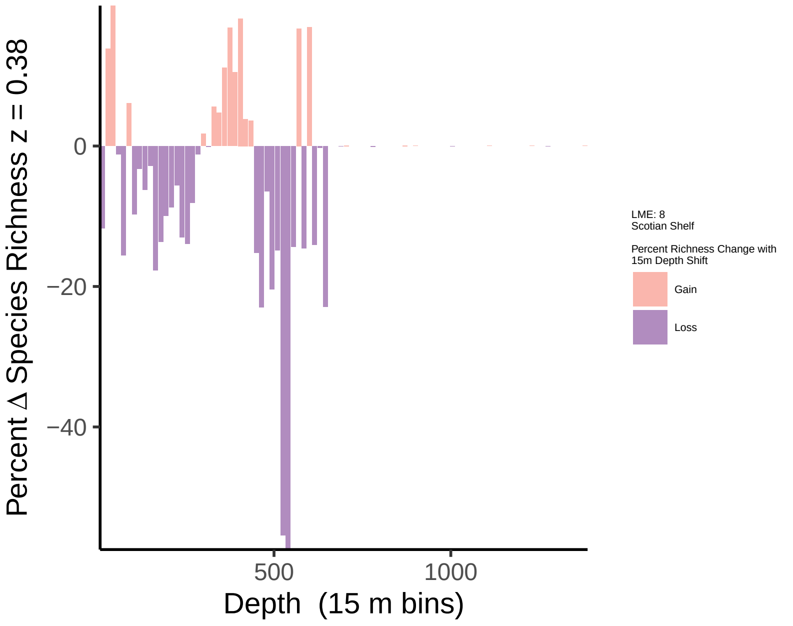

LME 11: Pacific Central−American Coastal

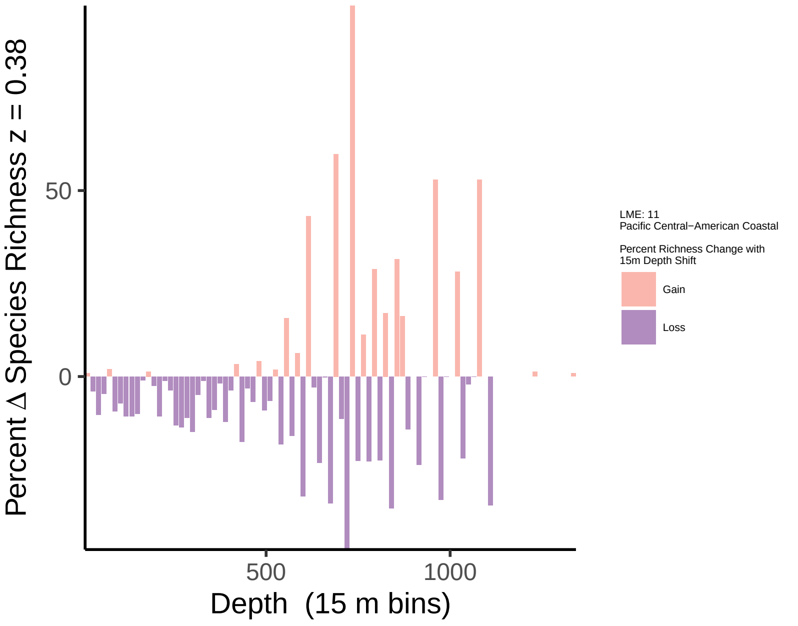

LME 9: Labrador − Newfoundland

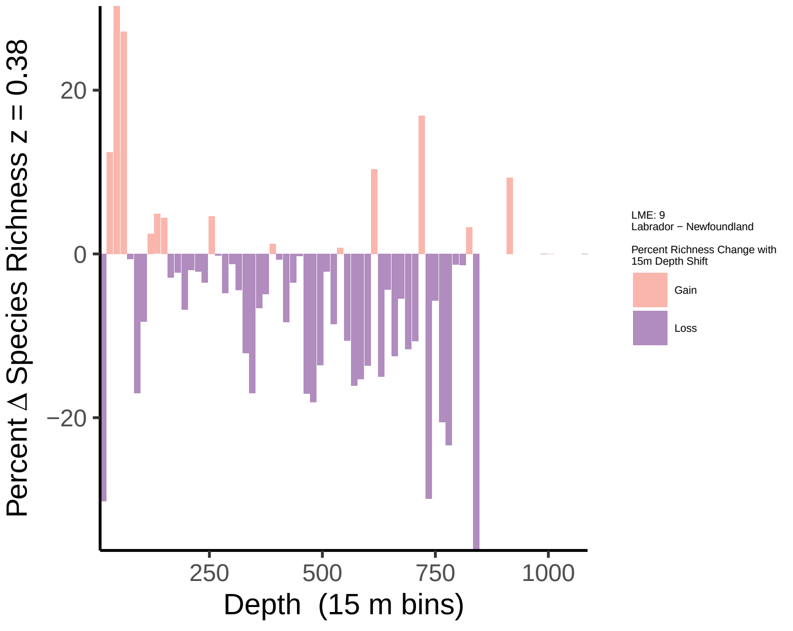

LME 12: Caribbean Sea

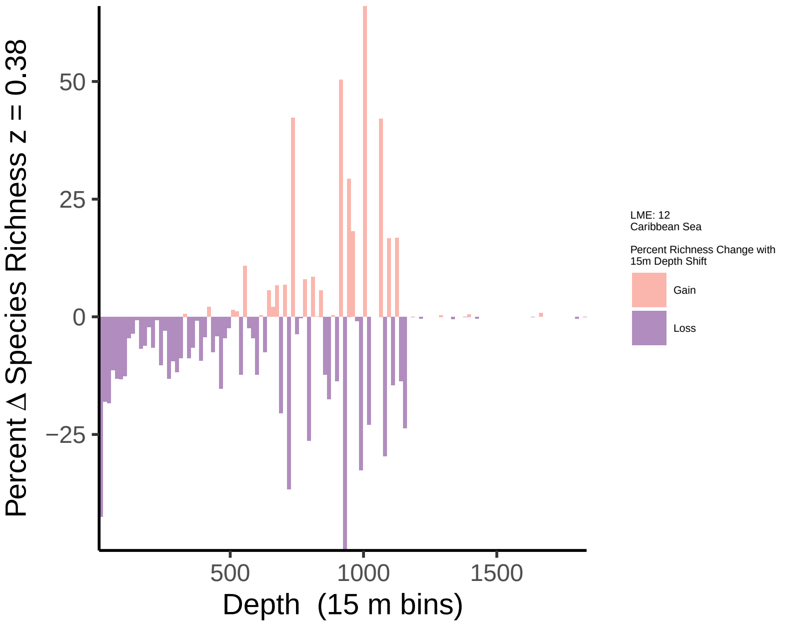

LME 13: Humboldt Current

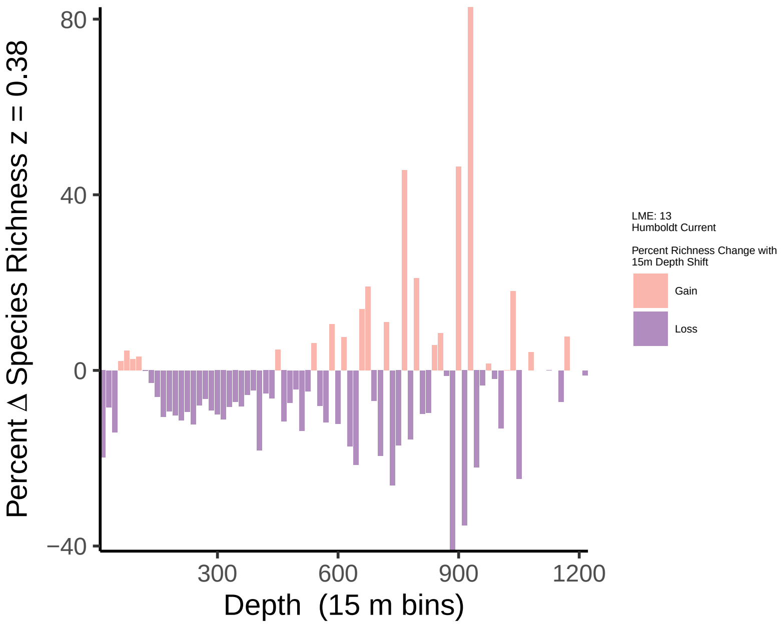

LME 16: East Brazil Shelf

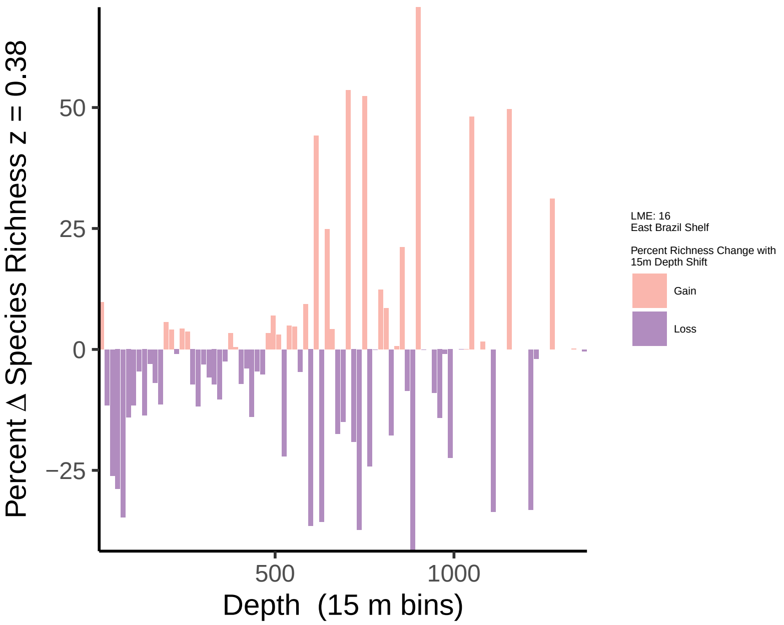

LME 14: Patagonian Shelf

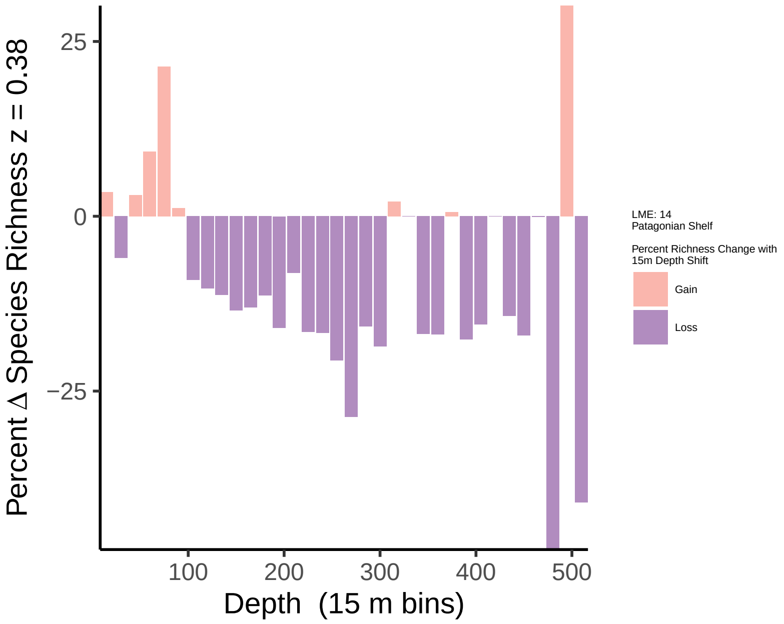

LME 17: North Brazil Shelf

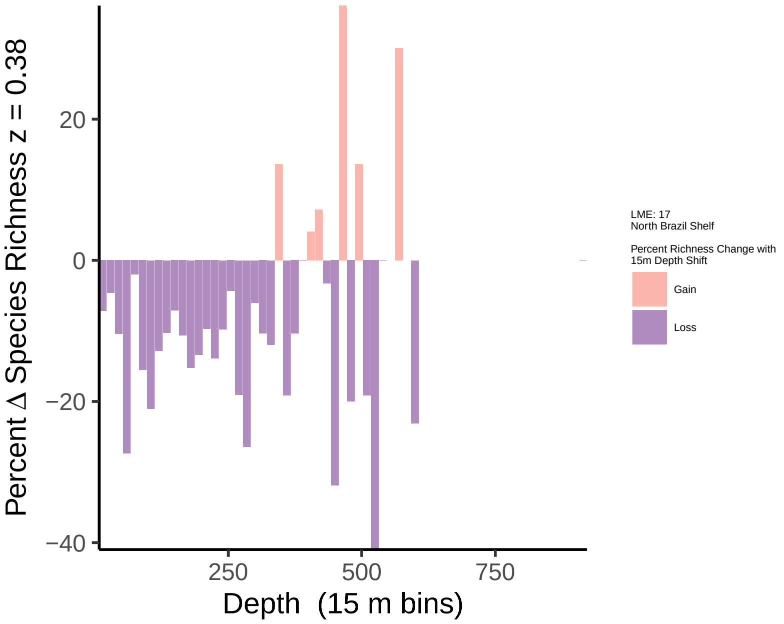

LME 15: South Brazil Shelf

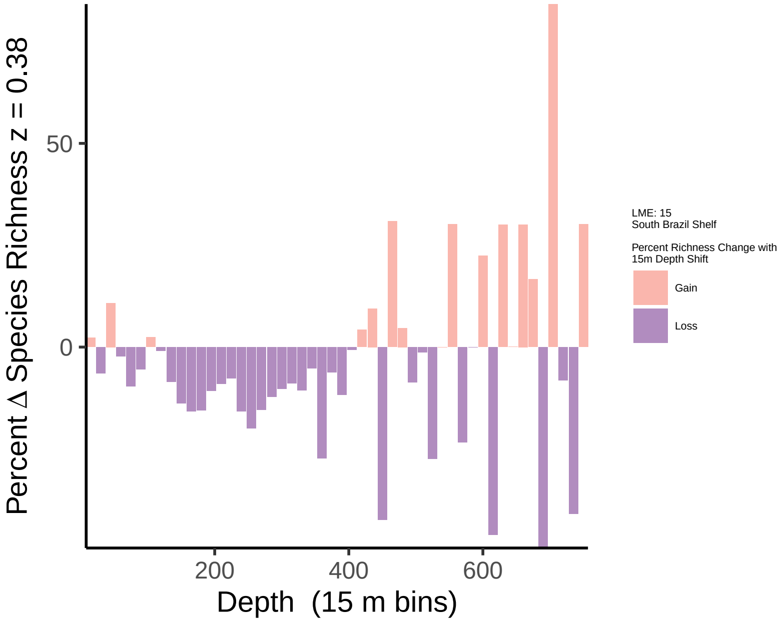

LME 18: Canadian Eastern Arctic − West Gree

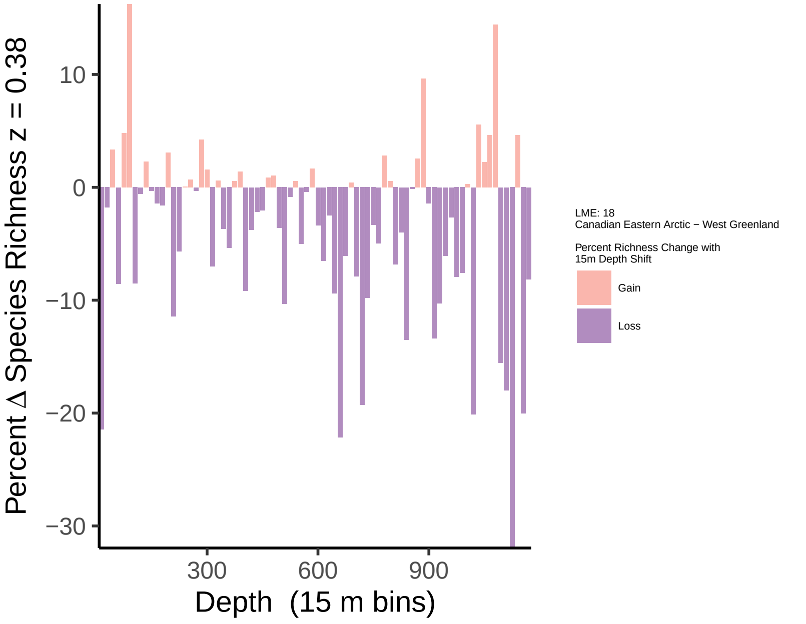

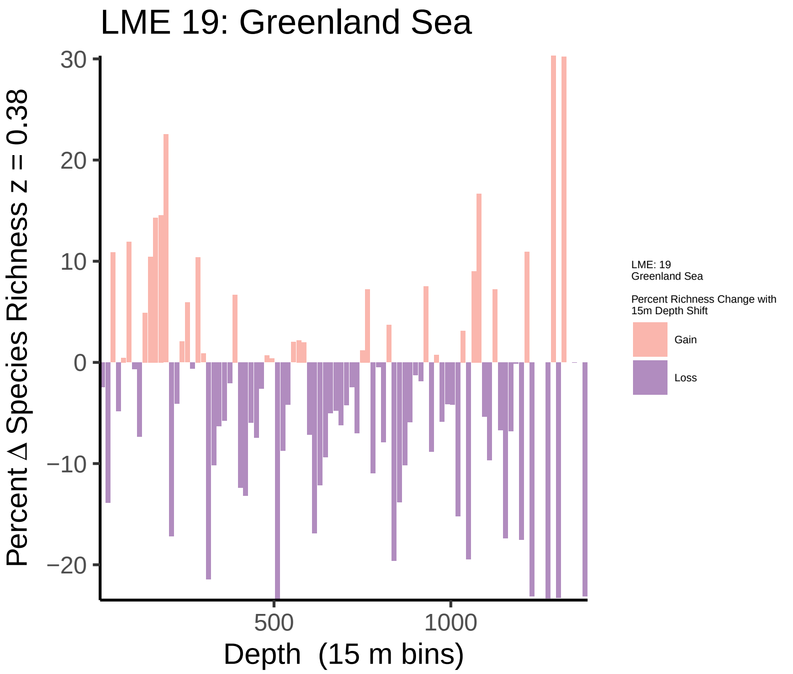
LME 19: Greenland Sea
Percent Δ Species Richness z = 0.38
30
20
10
0
−10
−20
500
1000
Depth (15 m bins)
LME: 19
Greenland Sea
Percent Richness Change with
15m Depth Shift
Gain
Loss

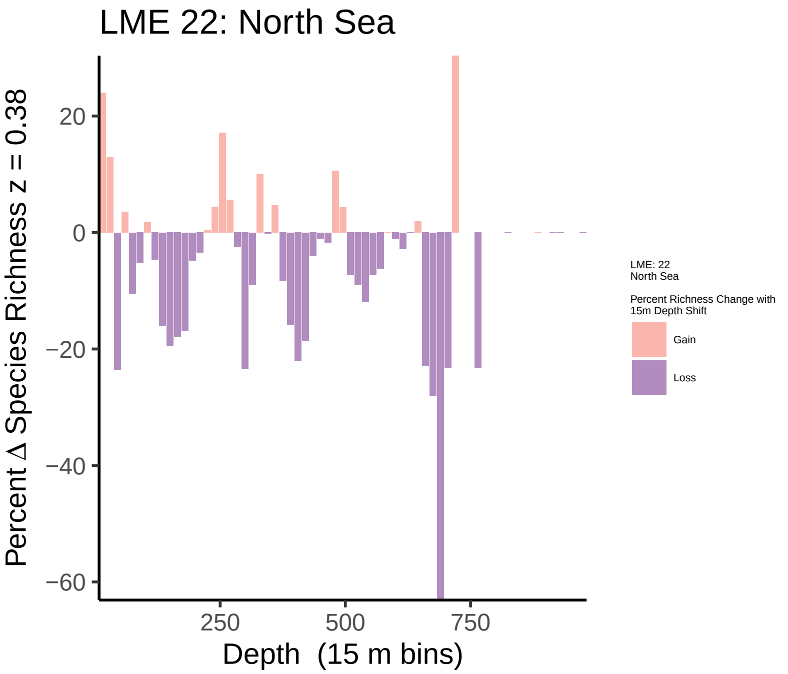
LME 22: North Sea
Percent Δ Species Richness z = 0.38
20
0
−20
−40
−60
250
500
750
Depth (15 m bins)
LME: 22
North Sea
Percent Richness Change with
15m Depth Shift
Gain
Loss

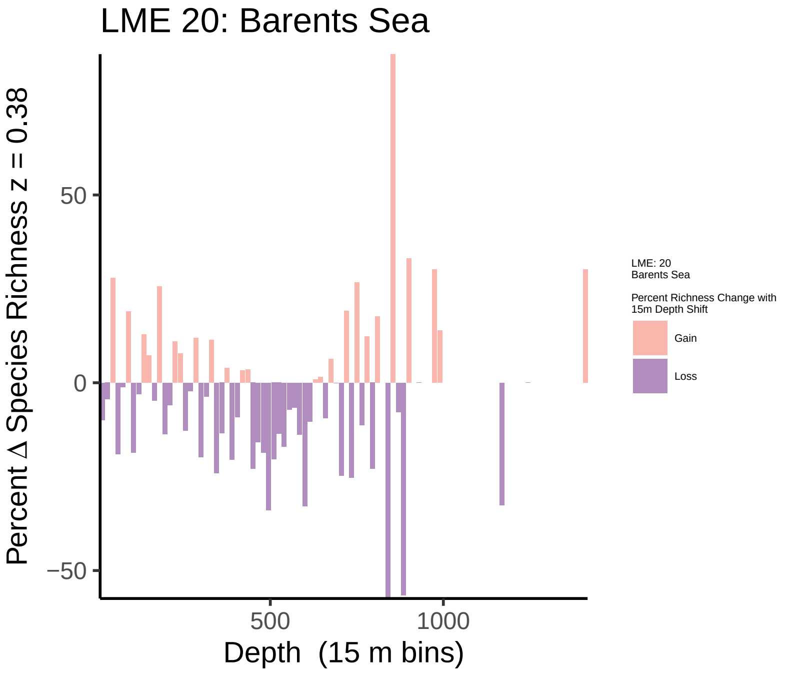
LME 20: Barents Sea
Percent Δ Species Richness z = 0.38
50
0
−50
500
1000
Depth (15 m bins)
LME: 20
Barents Sea
Percent Richness Change with
15m Depth Shift
Gain
Loss

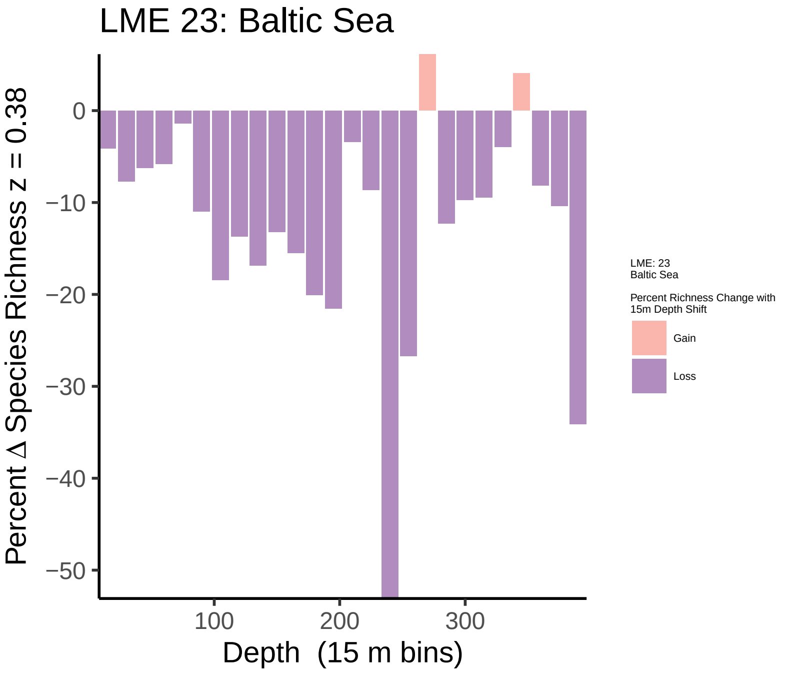
LME 23: Baltic Sea
Percent Δ Species Richness z = 0.38
0
−10
−20
−30
−40
−50
100
200
300
Depth (15 m bins)
LME: 23
Baltic Sea
Percent Richness Change with
15m Depth Shift
Gain
Loss

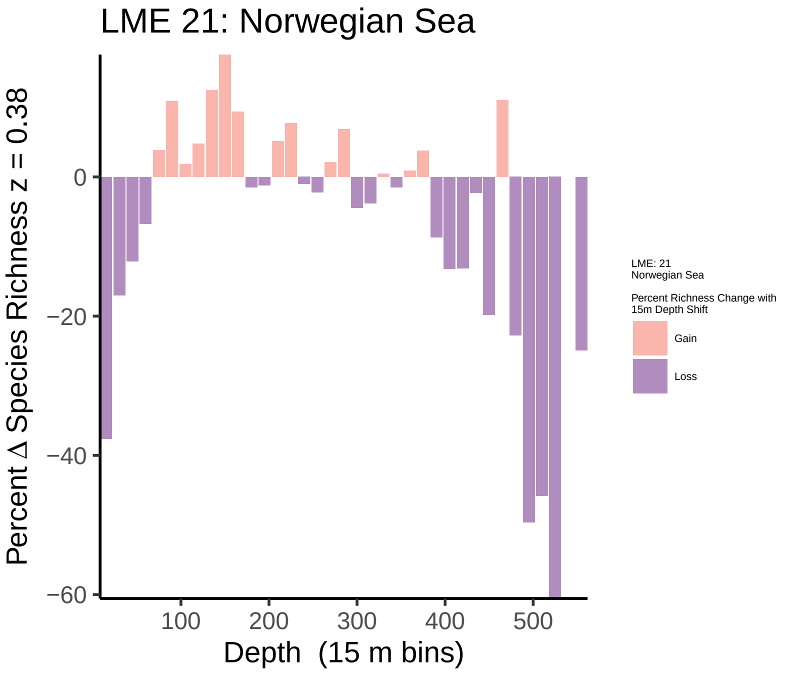
LME 21: Norwegian Sea
Percent Δ Species Richness z = 0.38
0
−20
−40
−60
100
200
300
400
500
Depth (15 m bins)
LME: 21
Norwegian Sea
Percent Richness Change with
15m Depth Shift
Gain
Loss

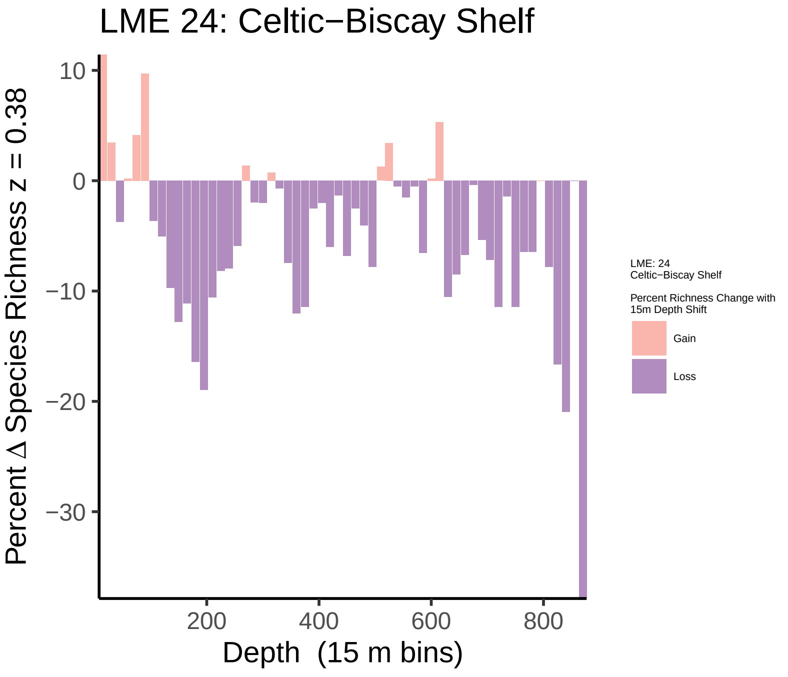
LME 24: Celtic−Biscay Shelf
Percent Δ Species Richness z = 0.38
10
0
−10
−20
−30
200
400
600
800
Depth (15 m bins)
LME: 24
Celtic−Biscay Shelf
Percent Richness Change with
15m Depth Shift
Gain
Loss

LME 25: Iberian Coastal

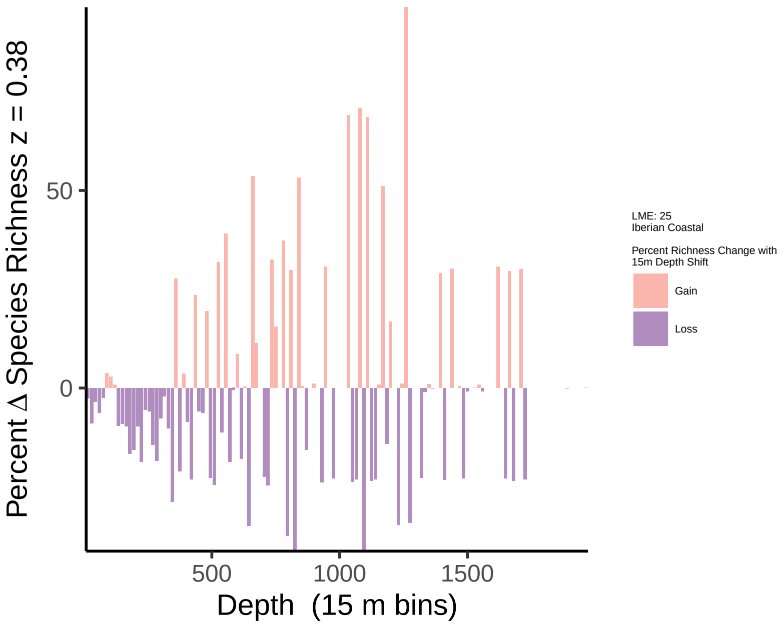

LME 28: Guinea Current

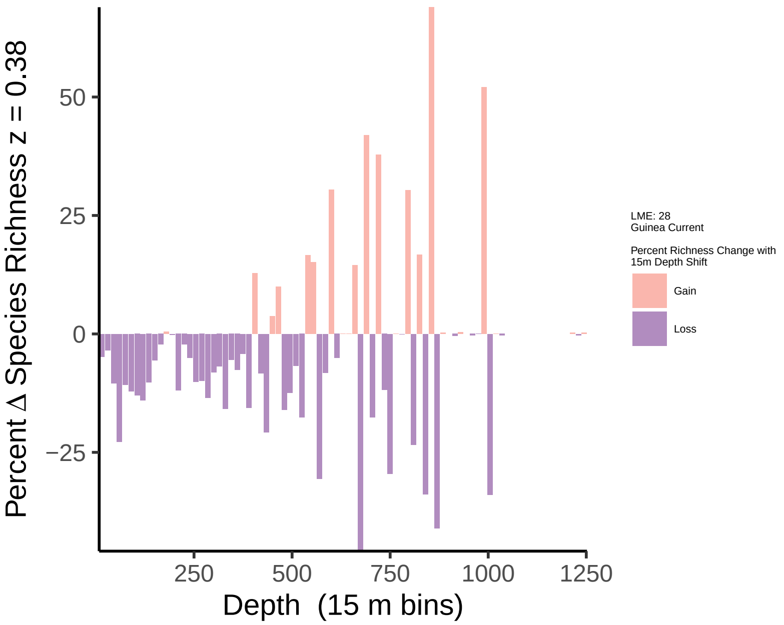

LME 26: Mediterranean Sea

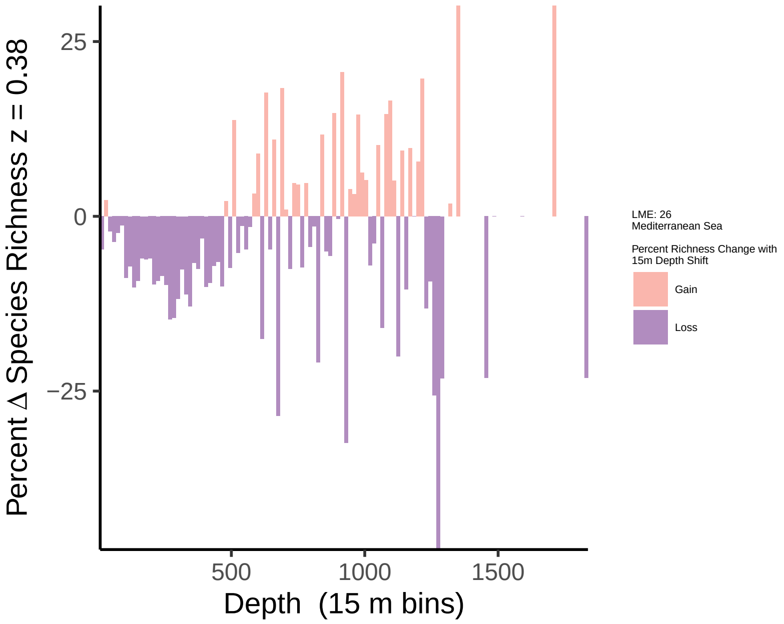

LME 29: Benguela Current

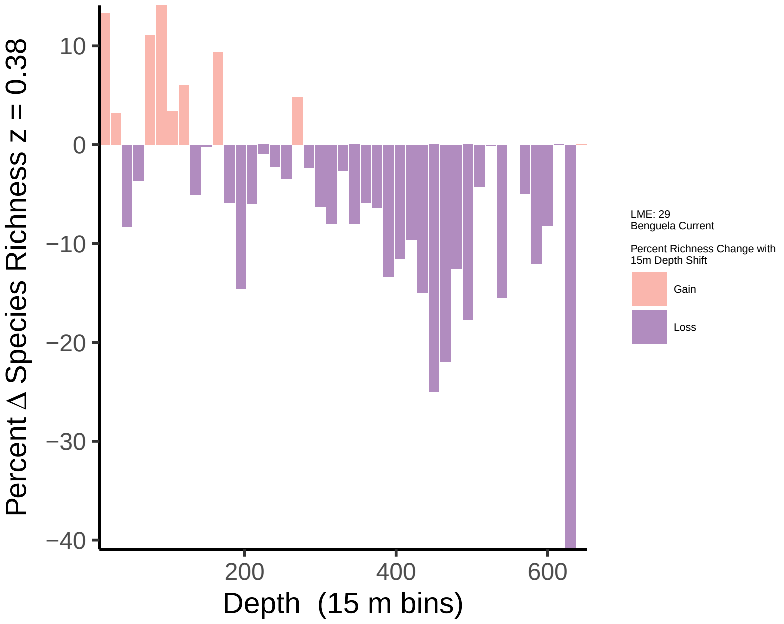

LME 27: Canary Current

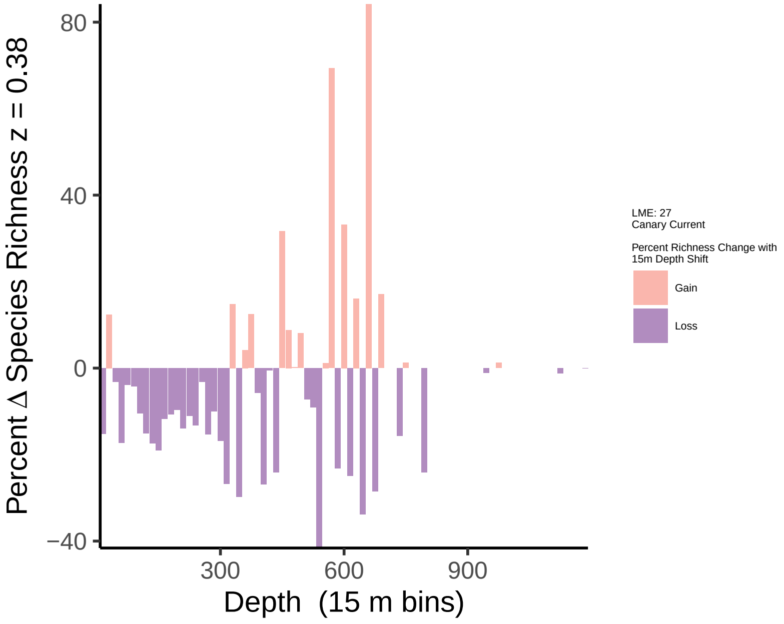

LME 30: Agulhas Current

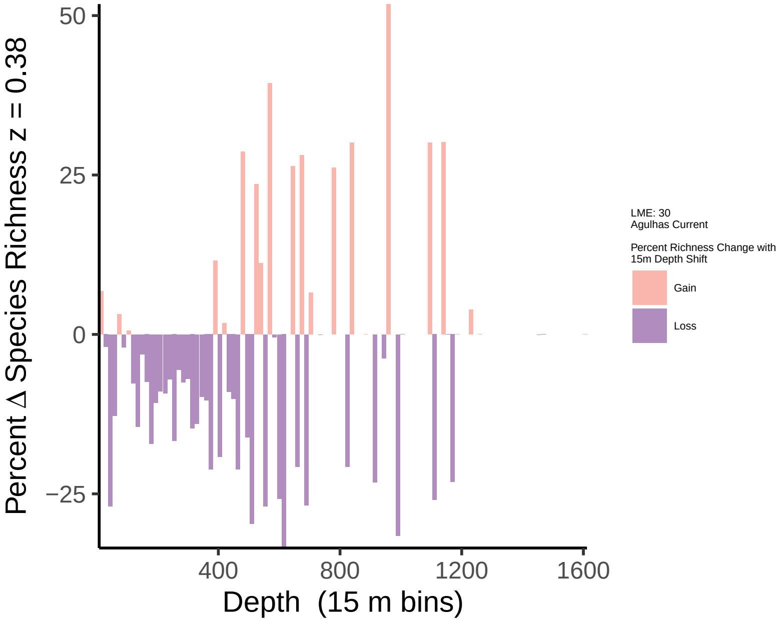

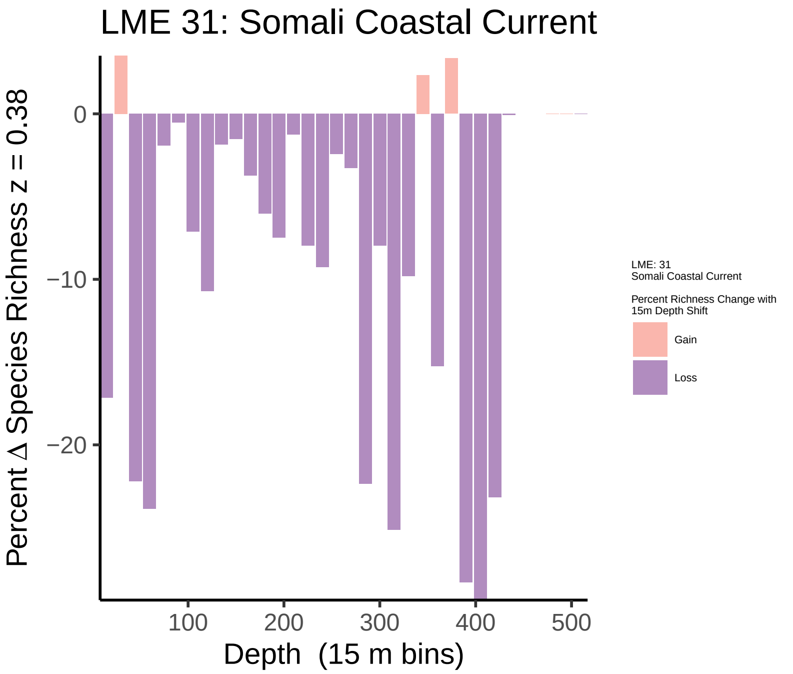
LME 31: Somali Coastal Current
Percent Δ Species Richness z = 0.38
0
−10
−20
100
200
300
400
500
Depth (15 m bins)
LME: 31
Somali Coastal Current
Percent Richness Change with
15m Depth Shift
Gain
Loss

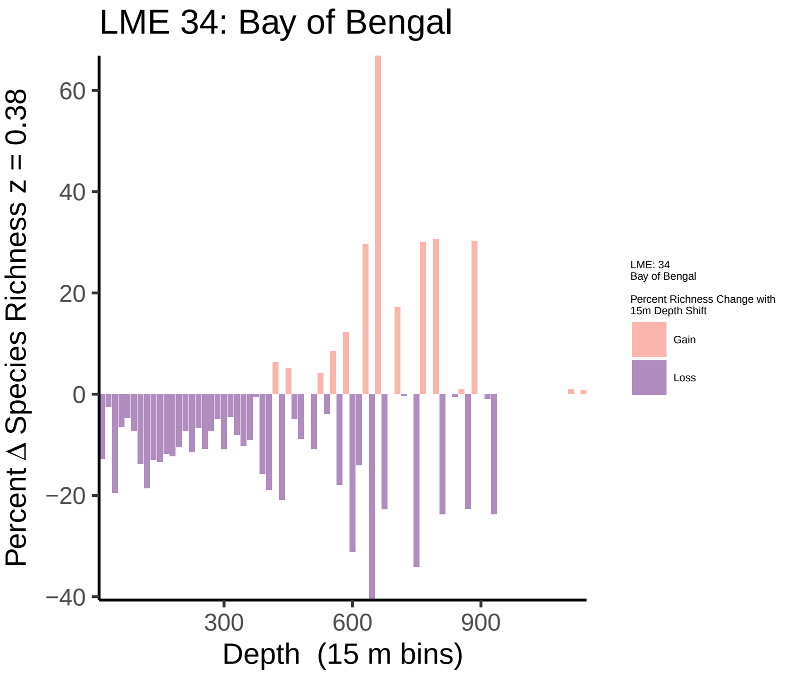
LME 34: Bay of Bengal
Percent Δ Species Richness z = 0.38
60
40
20
0
−20
−40
300
600
900
Depth (15 m bins)
LME: 34
Bay of Bengal
Percent Richness Change with
15m Depth Shift
Gain
Loss

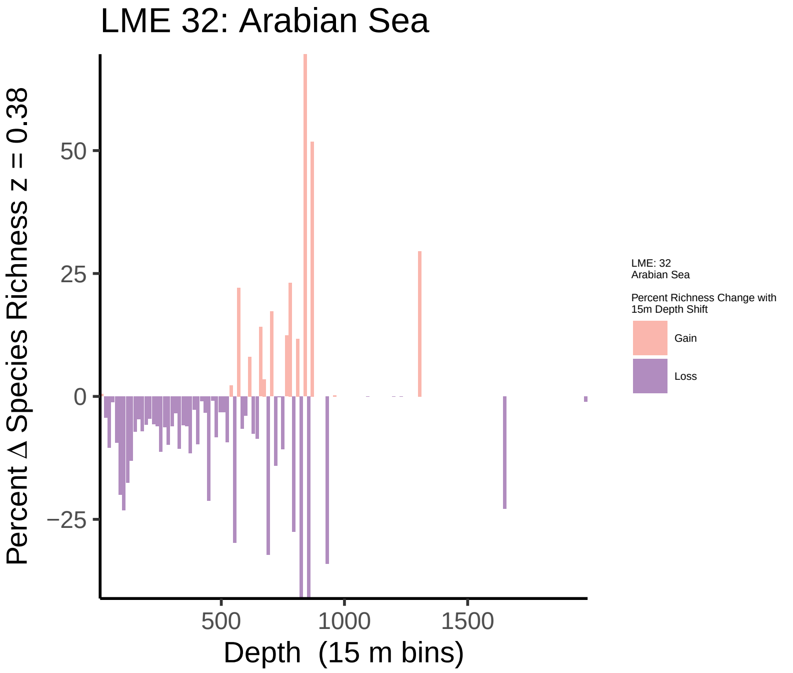
LME 32: Arabian Sea
Percent Δ Species Richness z = 0.38
50
25
0
−25
500
1000
1500
Depth (15 m bins)
LME: 32
Arabian Sea
Percent Richness Change with
15m Depth Shift
Gain
Loss

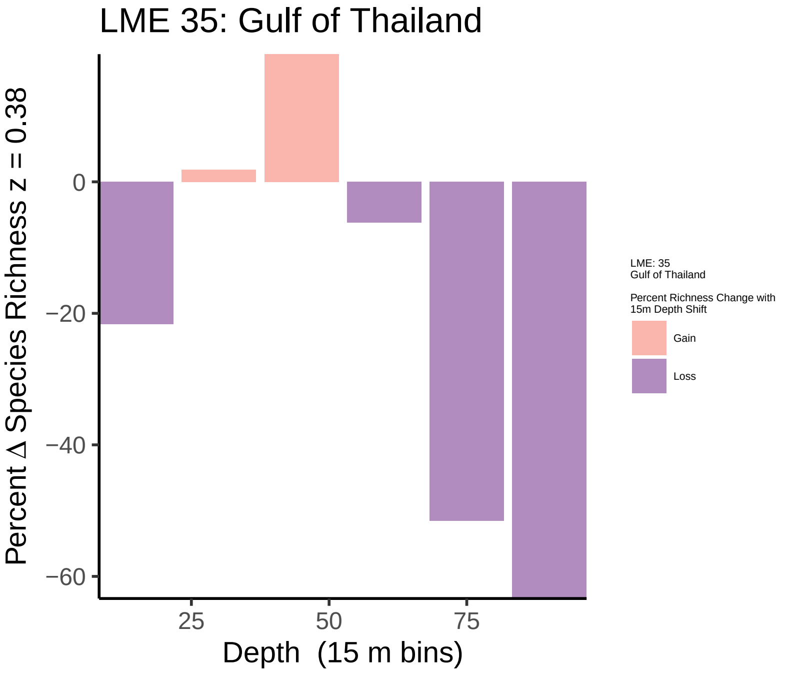
LME 35: Gulf of Thailand
Percent Δ Species Richness z = 0.38
0
−20
−40
−60
25
50
75
Depth (15 m bins)
LME: 35
Gulf of Thailand
Percent Richness Change with
15m Depth Shift
Gain
Loss

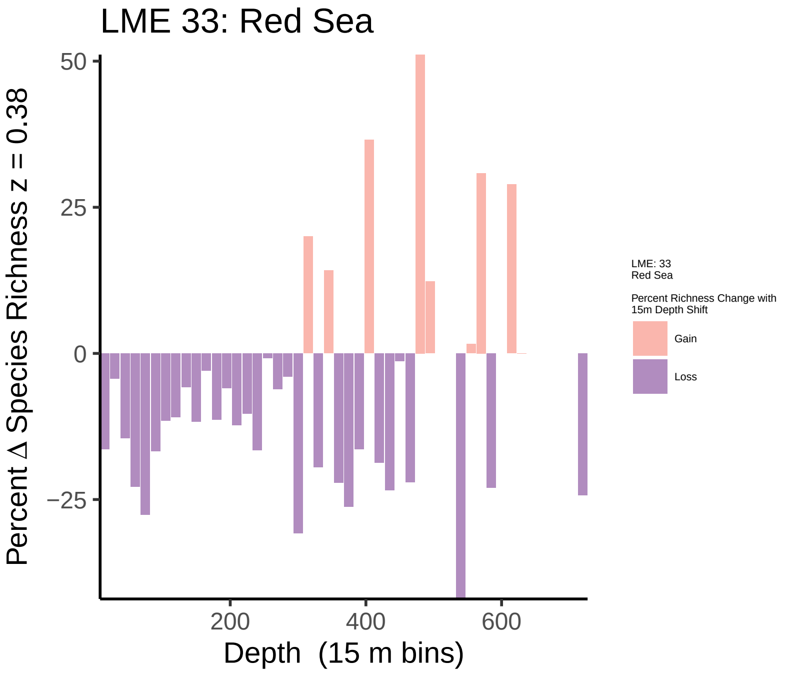
LME 33: Red Sea
Percent Δ Species Richness z = 0.38
50
25
0
−25
200
400
600
Depth (15 m bins)
LME: 33
Red Sea
Percent Richness Change with
15m Depth Shift
Gain
Loss

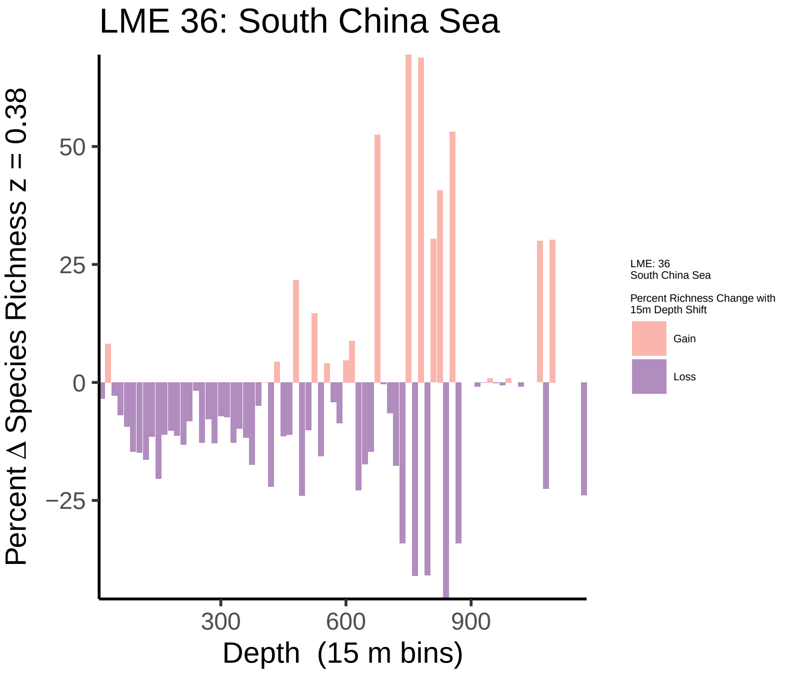
LME 36: South China Sea
Percent Δ Species Richness z = 0.38
50
25
0
−25
300
600
900
Depth (15 m bins)
LME: 36
South China Sea
Percent Richness Change with
15m Depth Shift
Gain
Loss

LME 37: Sulu−Celebes Sea

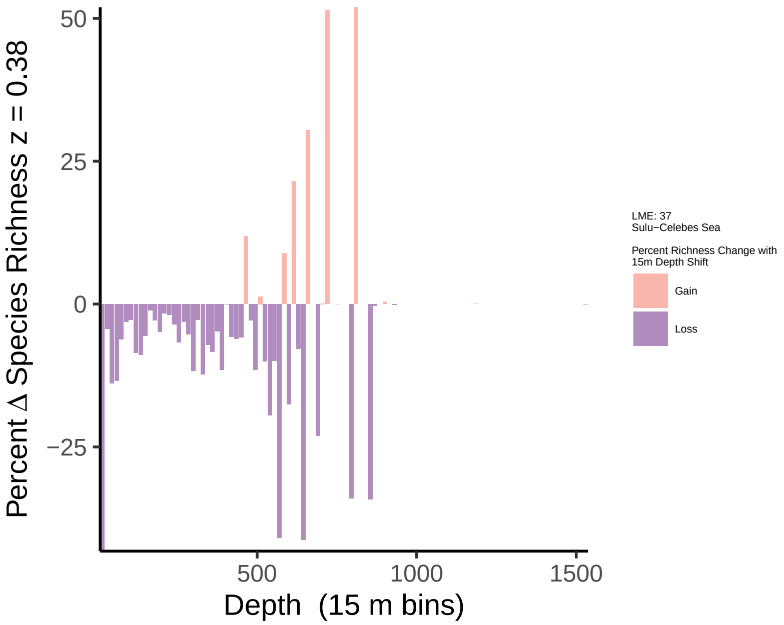

LME 38: Indonesian Sea

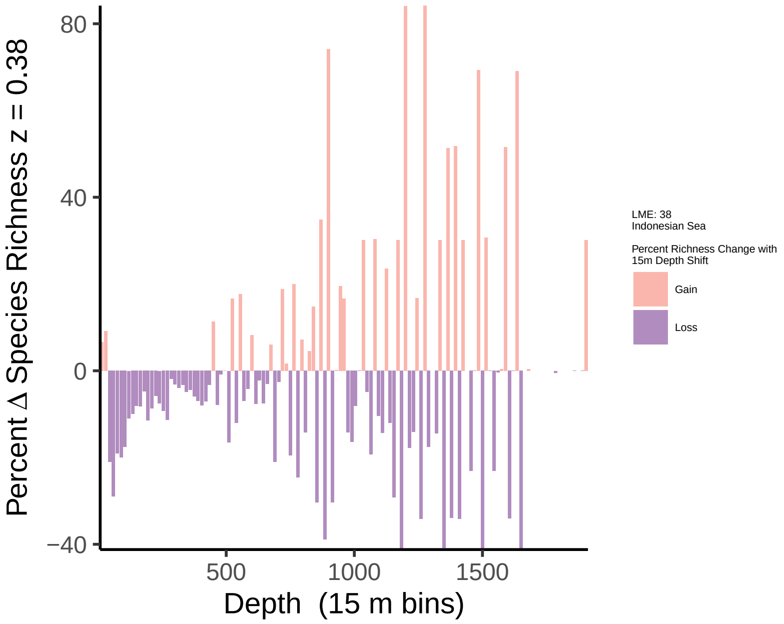

LME 39: North Australian Shelf

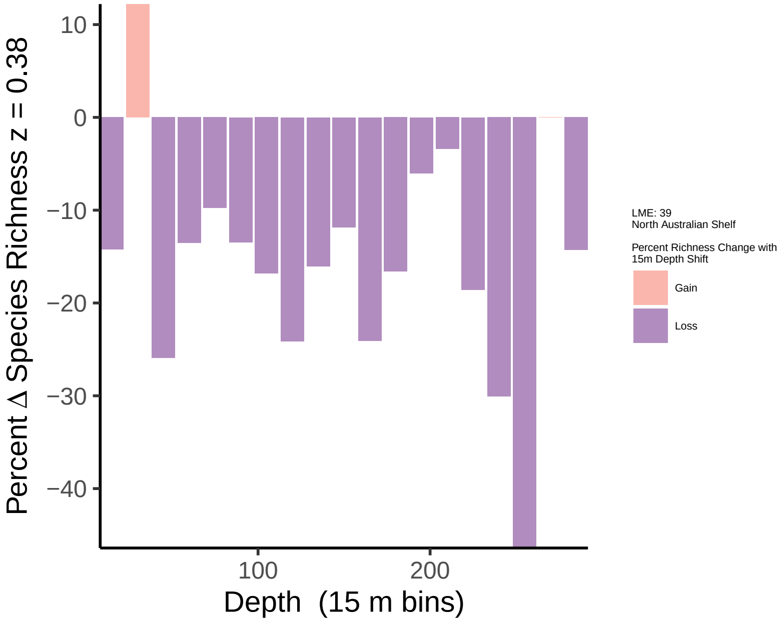

LME 40: Northeast Australian Shelf

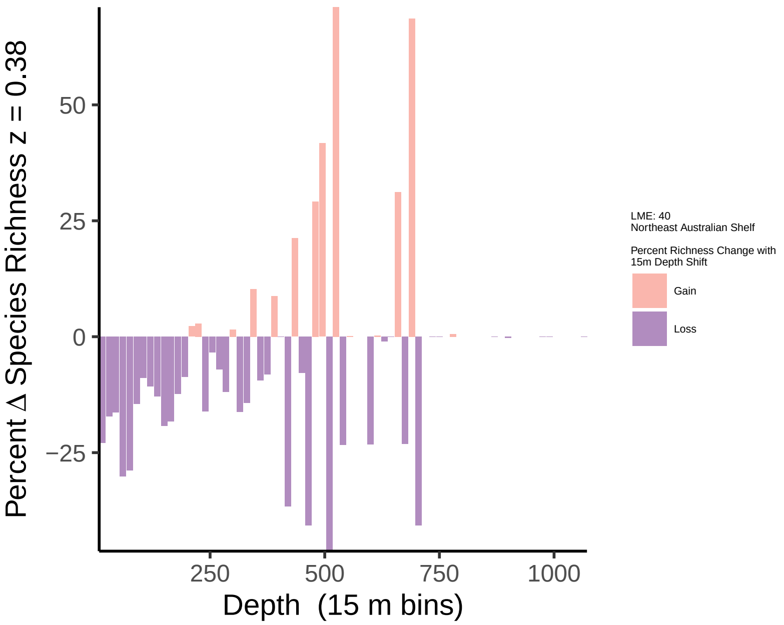

LME 41: East Central Australian Shelf

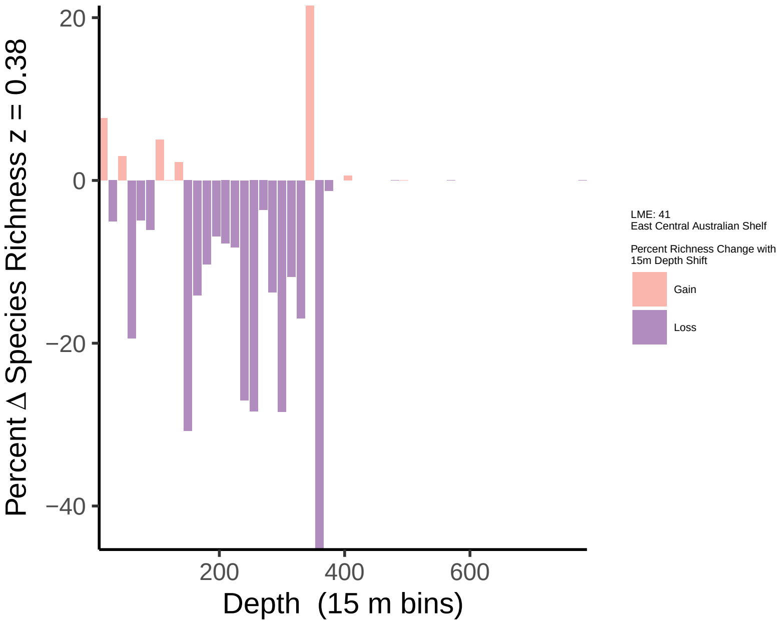

LME 42: Southeast Australian Shelf

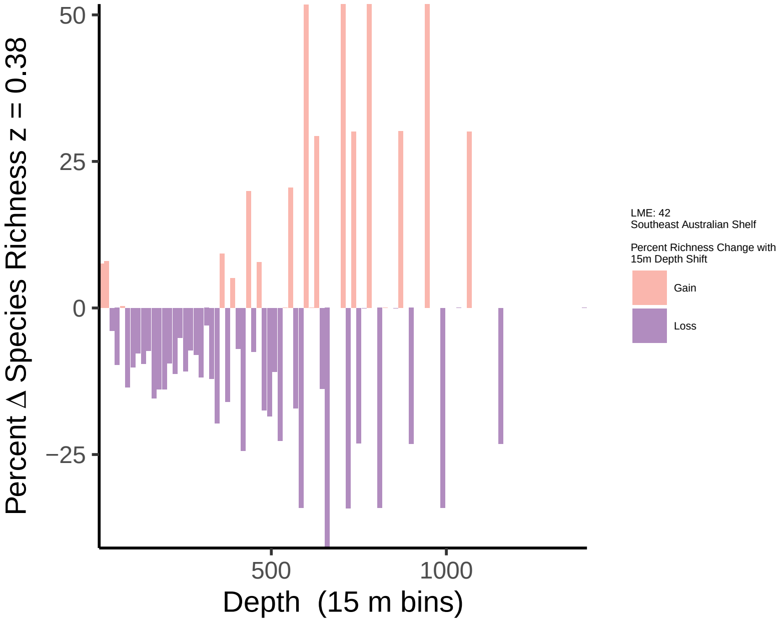

LME 43: South West Australian Shelf

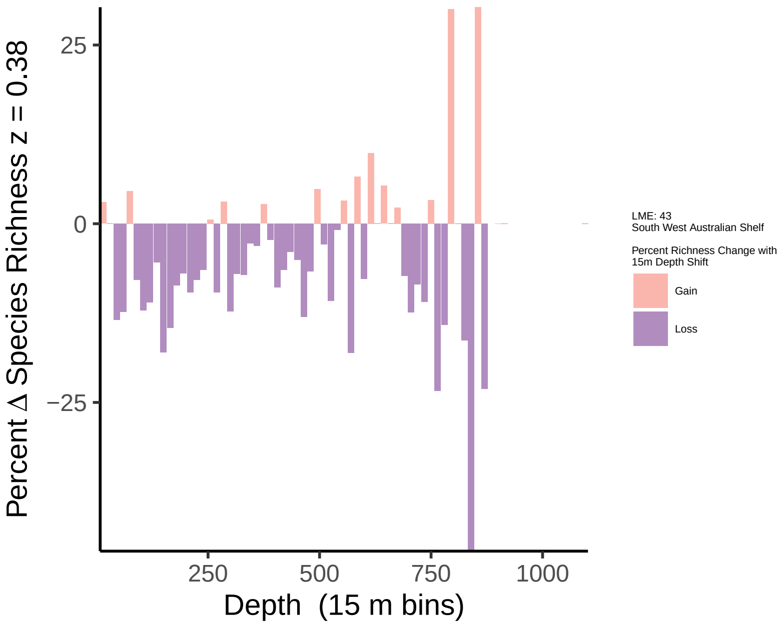

LME 46: New Zealand Shelf

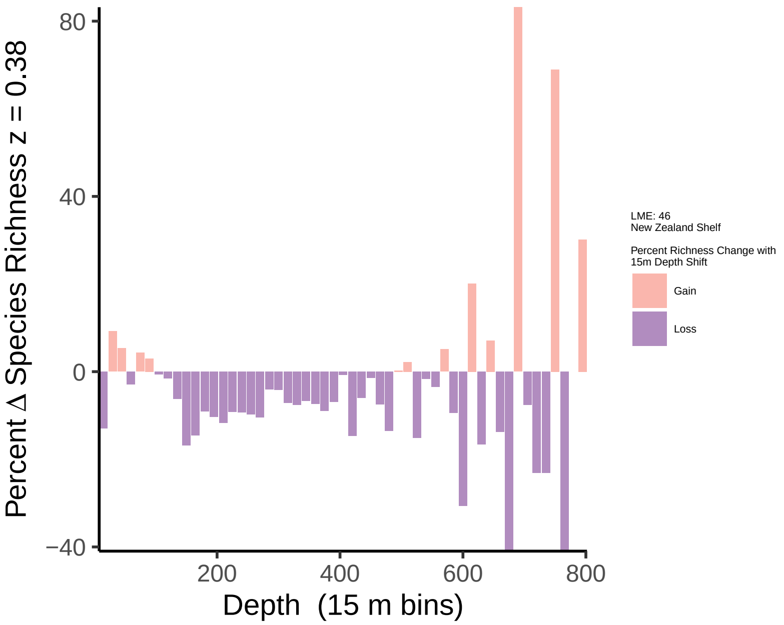

LME 44: West Central Australian Shelf

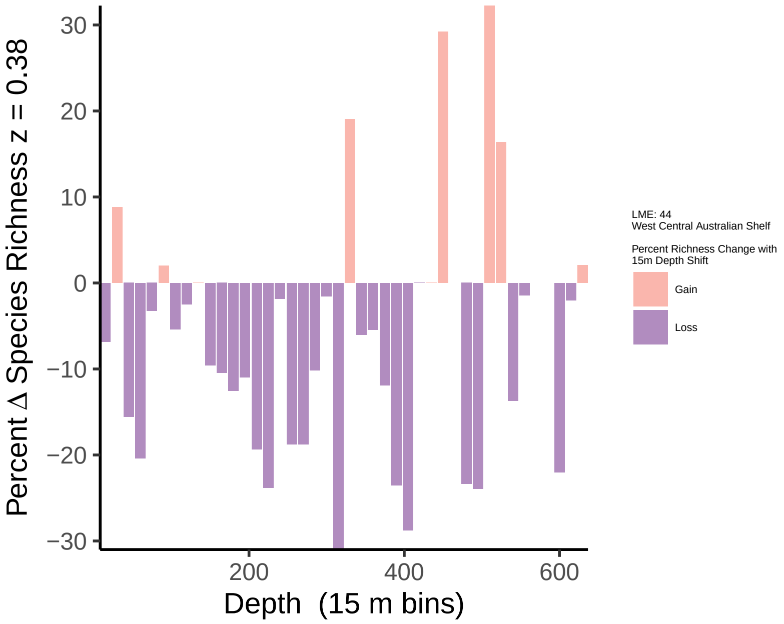

LME 47: East China Sea

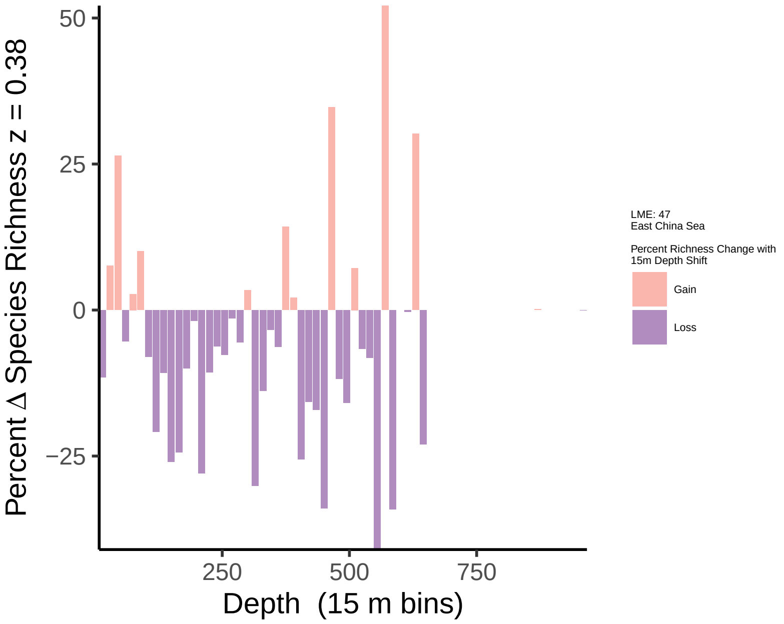

LME 45: Northwest Australian Shelf

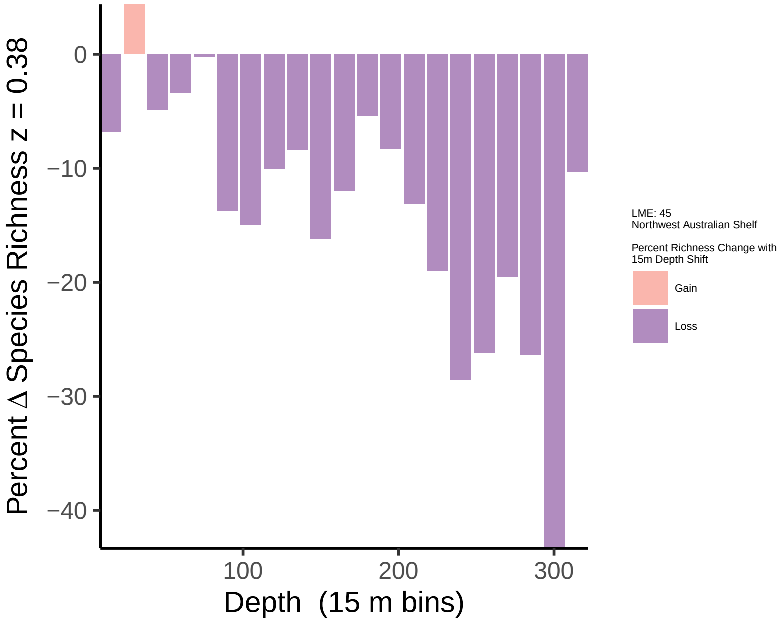

LME 48: Yellow Sea

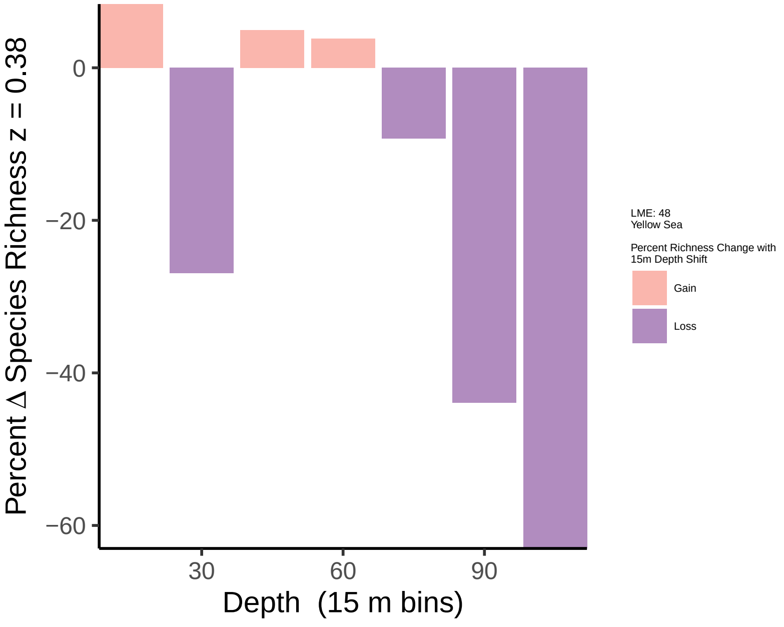

LME 49: Kuroshio Current

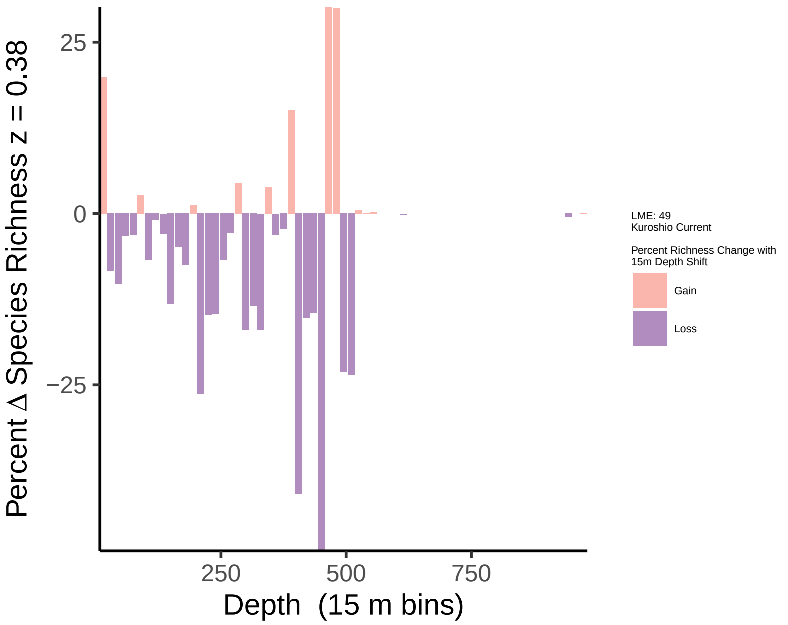

LME 52: Sea of Okhotsk

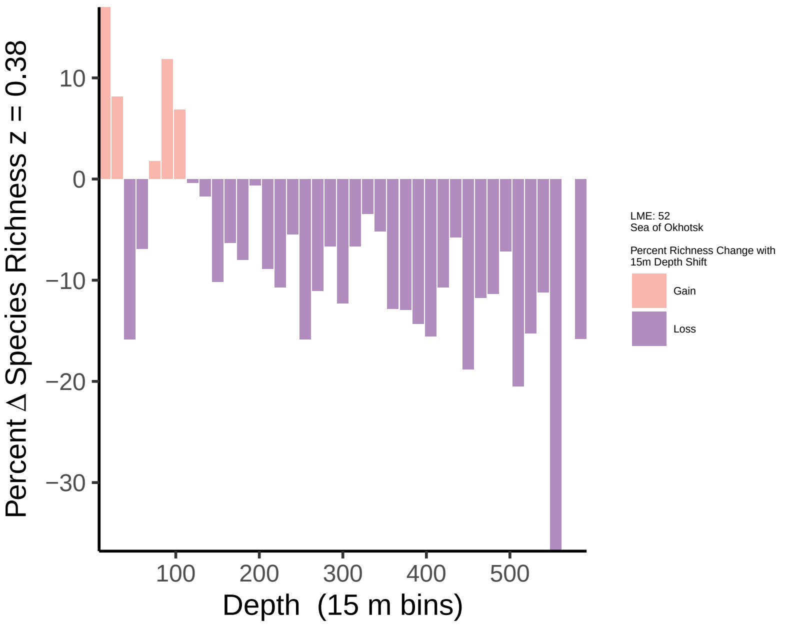

LME 50: Sea of Japan

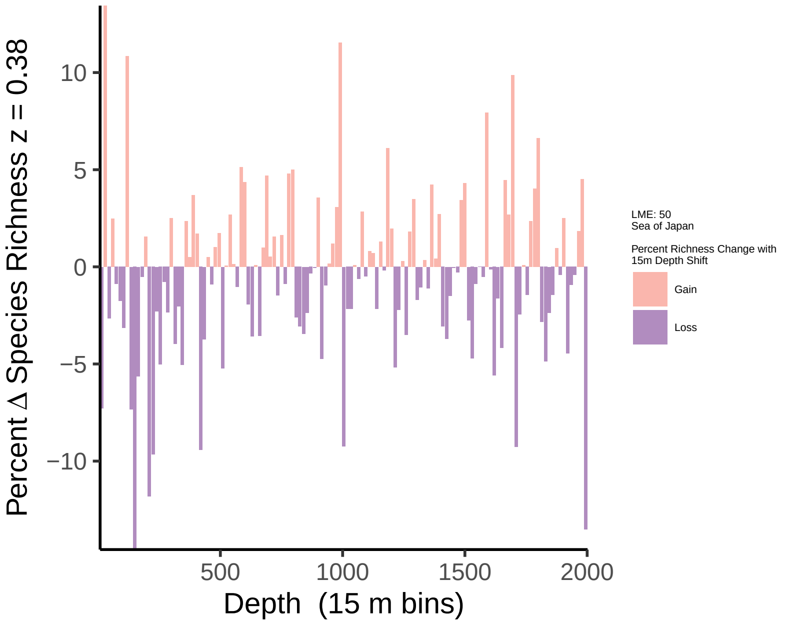

LME 53: West Bering Sea

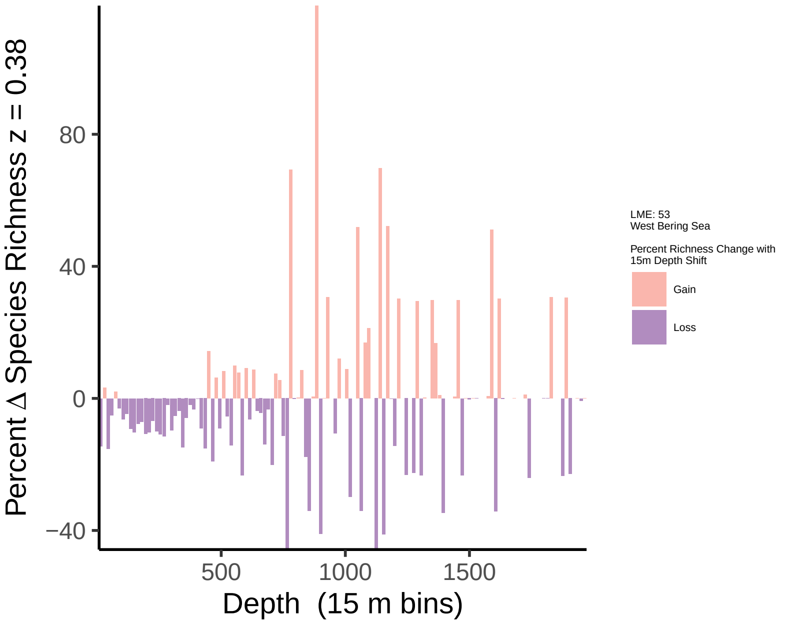

LME 51: Oyashio Current

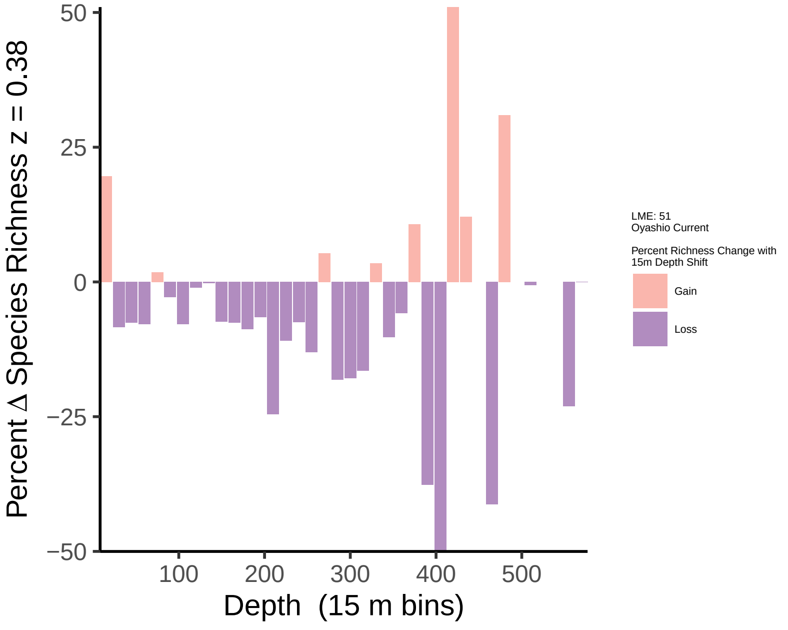

LME 54: Northern Bering − Chukchi Seas

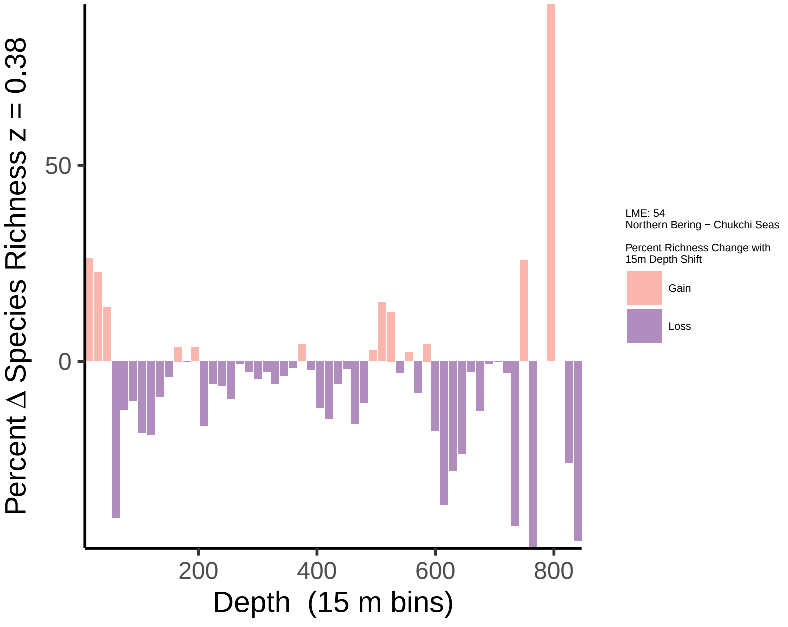

LME 55: Beaufort Sea

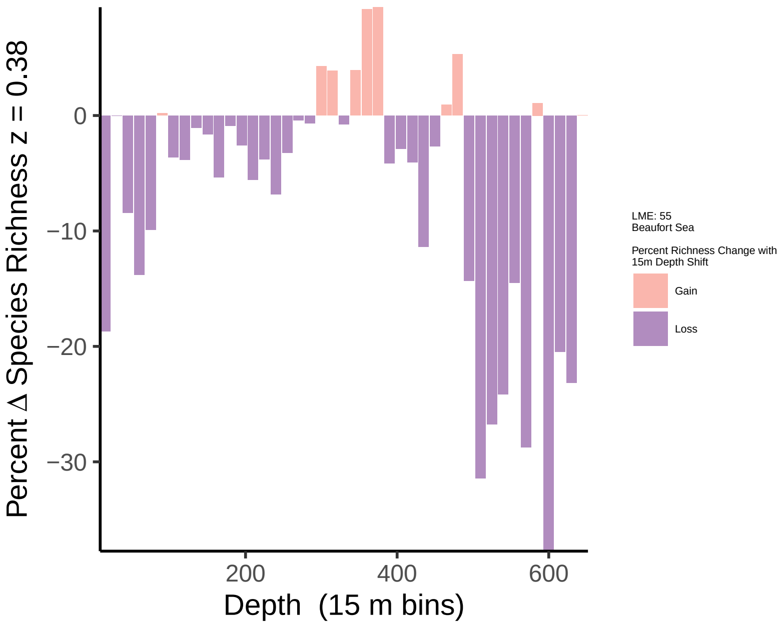

LME 58: Kara Sea

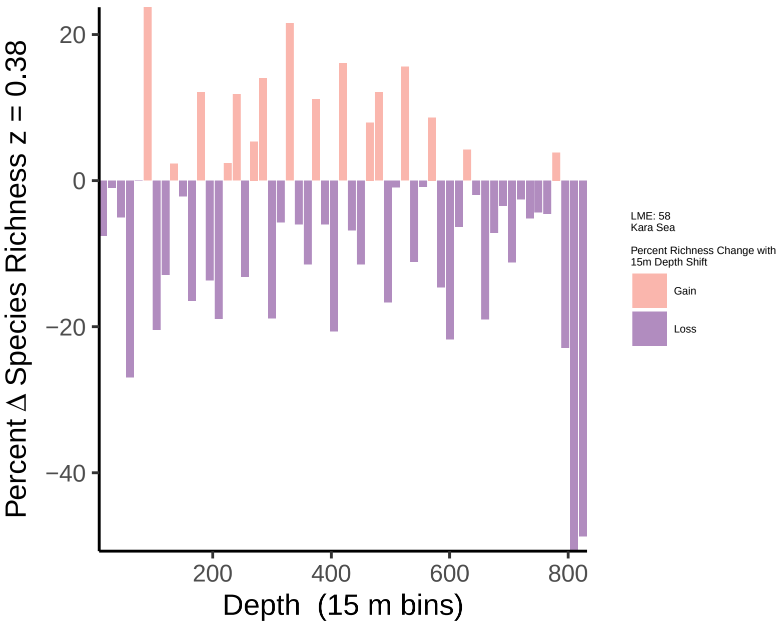

LME 56: East Siberian Sea

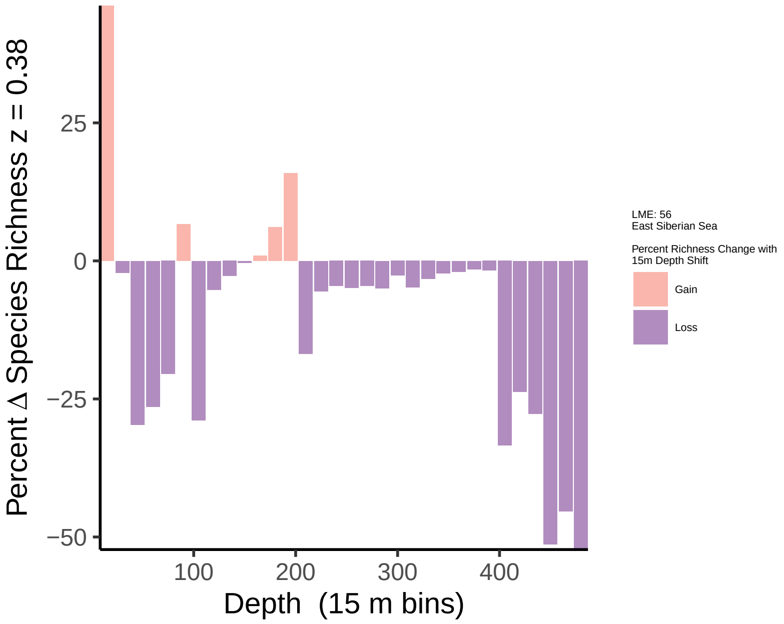

LME 59: Iceland Shelf and Sea

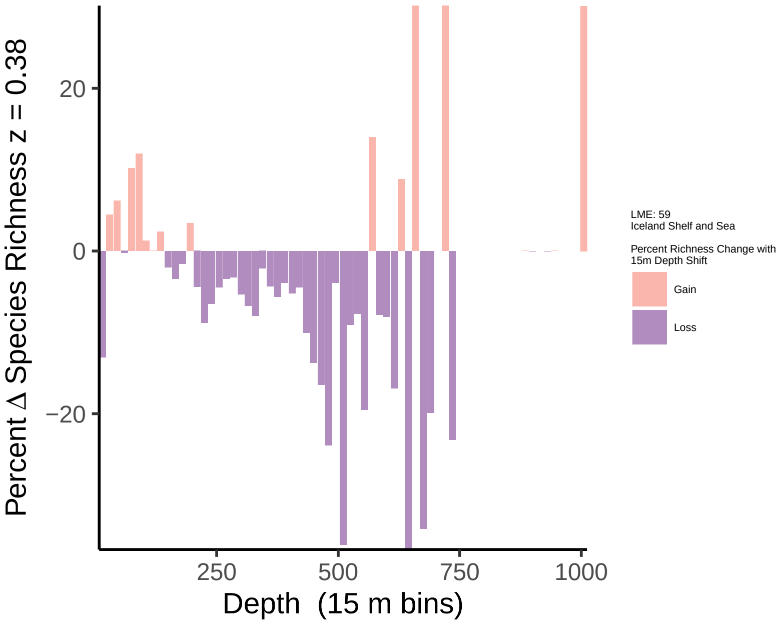

LME 57: Laptev Sea

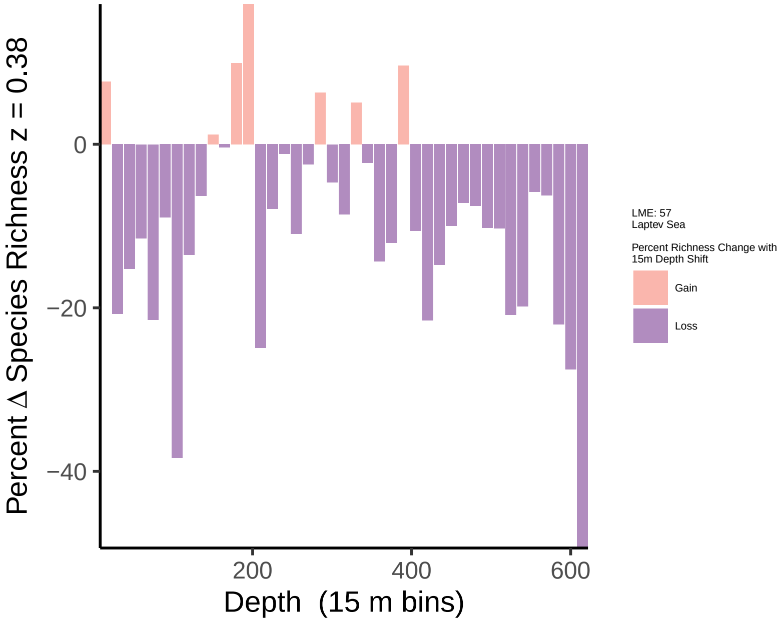

LME 60: Faroe Plateau

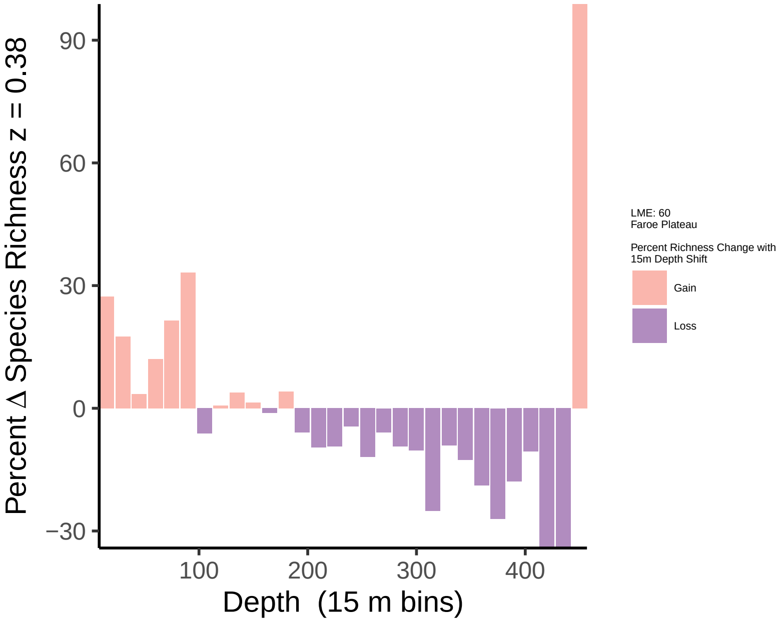

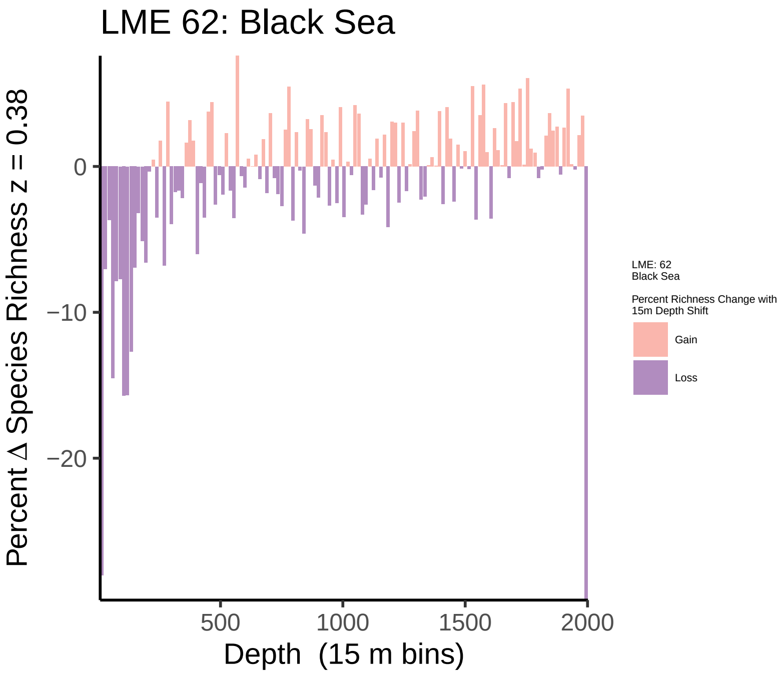
LME 62: Black Sea
Percent Δ Species Richness z = 0.38
0
−10
−20
500
1000
1500
2000
Depth (15 m bins)
LME: 62
Black Sea
Percent Richness Change with
15m Depth Shift
Gain
Loss

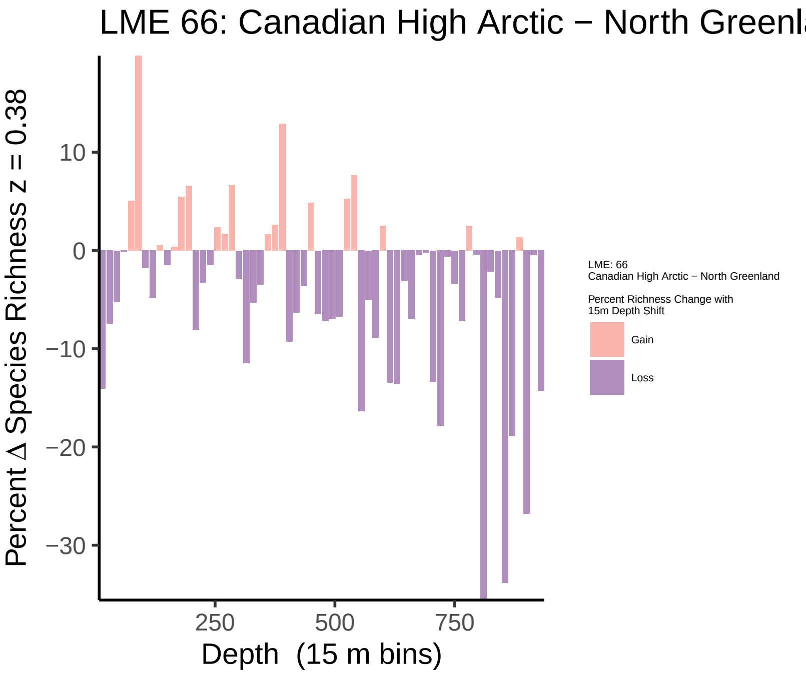
LME 66: Canadian High Arctic − North Greenla
Percent Δ Species Richness z = 0.38
10
0
−10
−20
−30
250
500
750
Depth (15 m bins)
LME: 66
Canadian High Arctic − North Greenland
Percent Richness Change with
15m Depth Shift
Gain
Loss

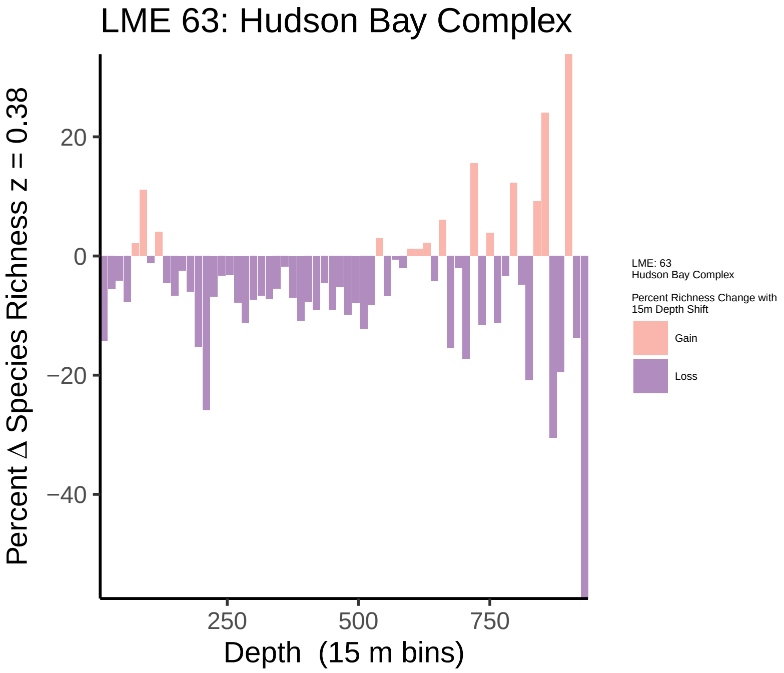
LME 63: Hudson Bay Complex
Percent Δ Species Richness z = 0.38
20
0
−20
−40
250
500
750
Depth (15 m bins)
LME: 63
Hudson Bay Complex
Percent Richness Change with
15m Depth Shift
Gain
Loss

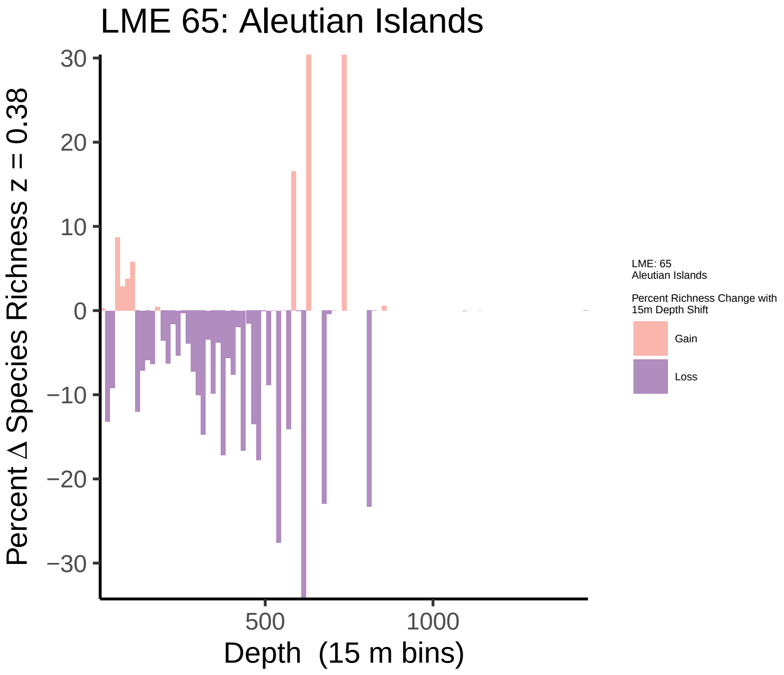
LME 65: Aleutian Islands
Percent Δ Species Richness z = 0.38
30
20
10
0
−10
−20
−30
500
1000
Depth (15 m bins)
LME: 65
Aleutian Islands
Percent Richness Change with
15m Depth Shift
Gain
Loss

Fig S10 c.

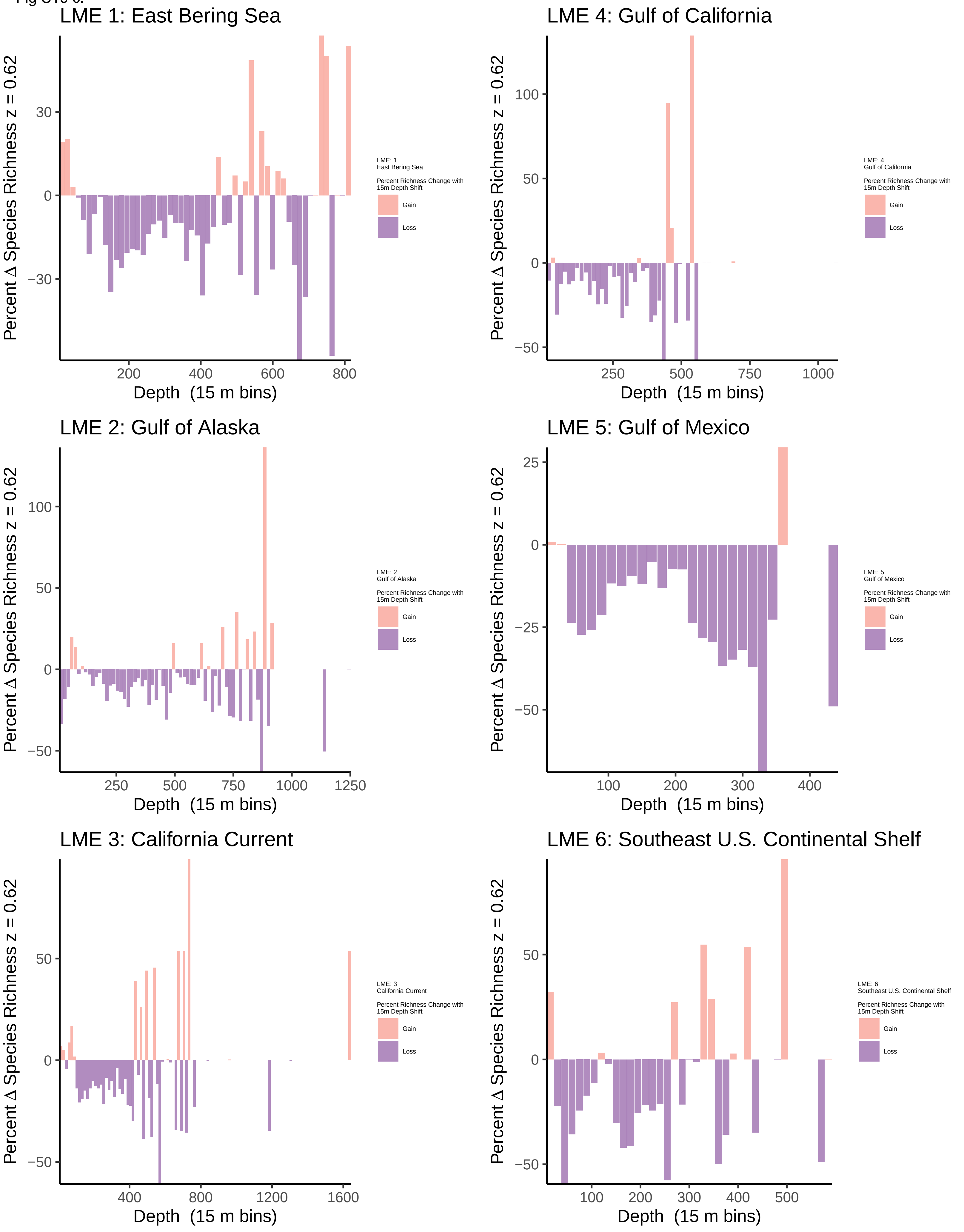

LME 1: East Bering Sea
Percent Δ Species Richness z = 0.62
Depth (15 m bins)
LME: 1
East Bering Sea
Percent Richness Change with
15m Depth Shift
Gain
Loss
LME 4: Gulf of California
Percent Δ Species Richness z = 0.62
Depth (15 m bins)
LME: 4
Gulf of California
Percent Richness Change with
15m Depth Shift
Gain
Loss
LME 2: Gulf of Alaska
Percent Δ Species Richness z = 0.62
Depth (15 m bins)
LME: 2
Gulf of Alaska
Percent Richness Change with
15m Depth Shift
Gain
Loss
LME 5: Gulf of Mexico
Percent Δ Species Richness z = 0.62
Depth (15 m bins)
LME: 5
Gulf of Mexico
Percent Richness Change with
15m Depth Shift
Gain
Loss
LME 3: California Current
Percent Δ Species Richness z = 0.62
Depth (15 m bins)
LME: 3
California Current
Percent Richness Change with
15m Depth Shift
Gain
Loss
LME 6: Southeast U.S. Continental Shelf
Percent Δ Species Richness z = 0.62
Depth (15 m bins)
LME: 6
Southeast U.S. Continental Shelf
Percent Richness Change with
15m Depth Shift
Gain
Loss

LME 7: Northeast U.S. Continental Shelf

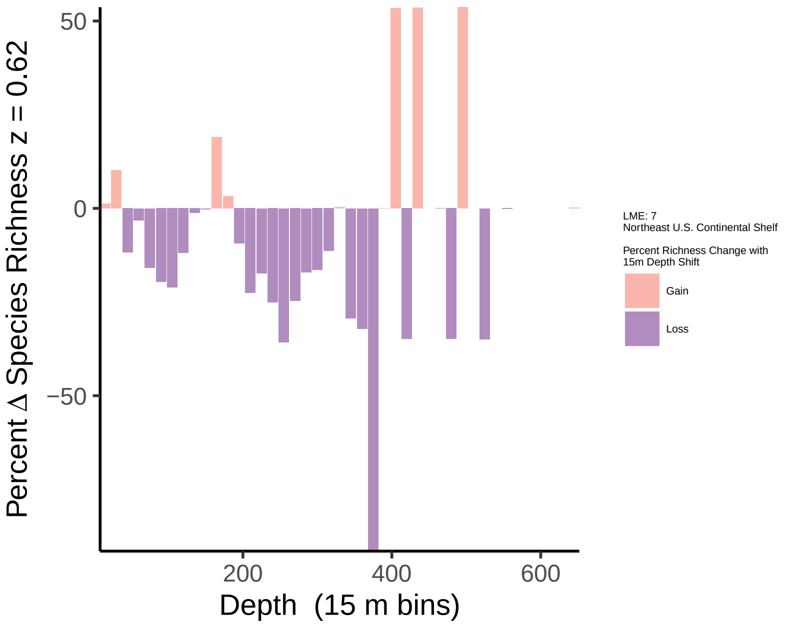

LME 10: Insular Pacific−Hawaiian

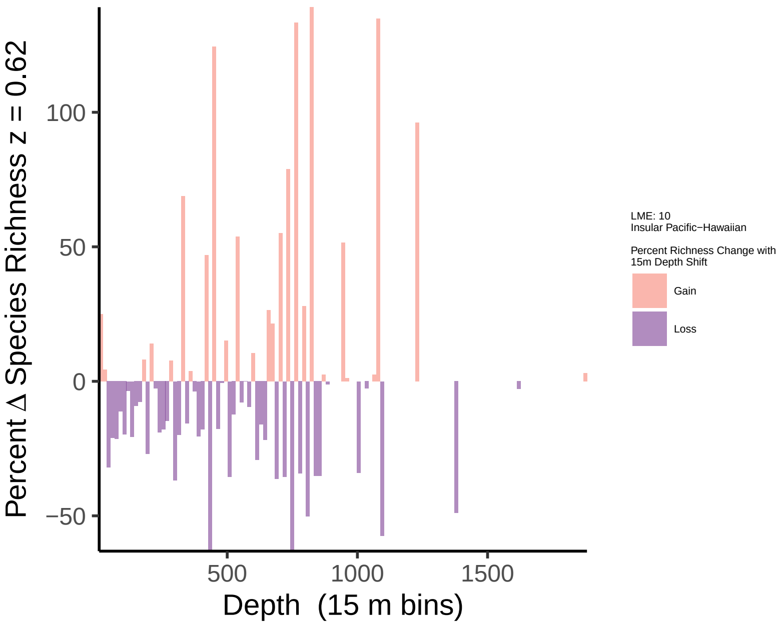

LME 8: Scotian Shelf

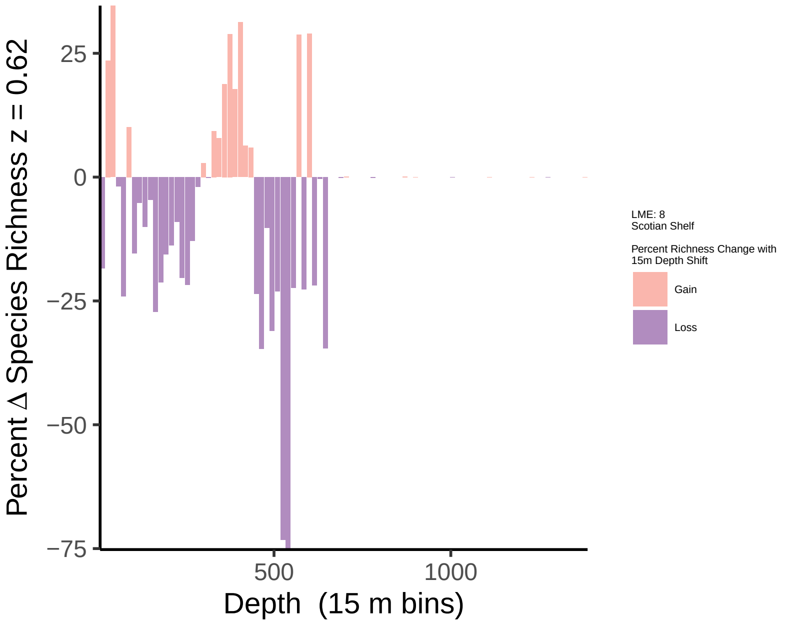

LME 11: Pacific Central−American Coastal

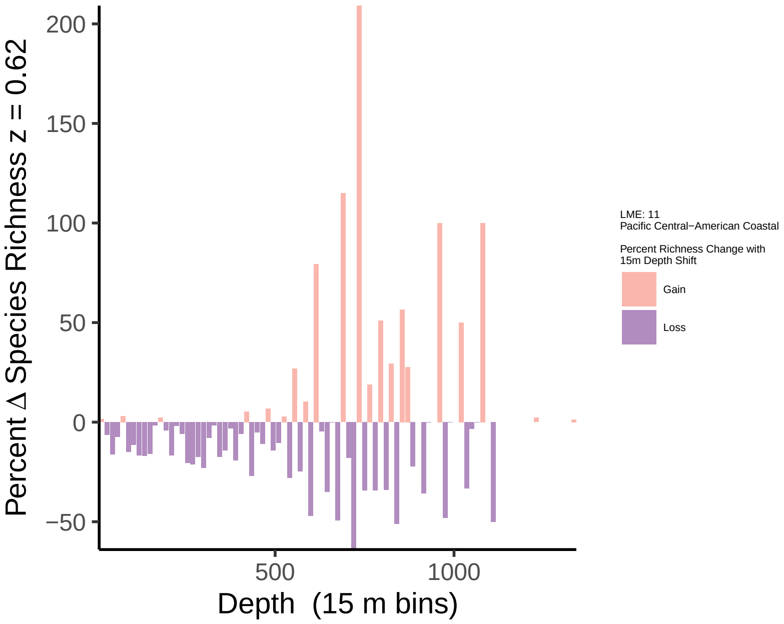

LME 9: Labrador − Newfoundland

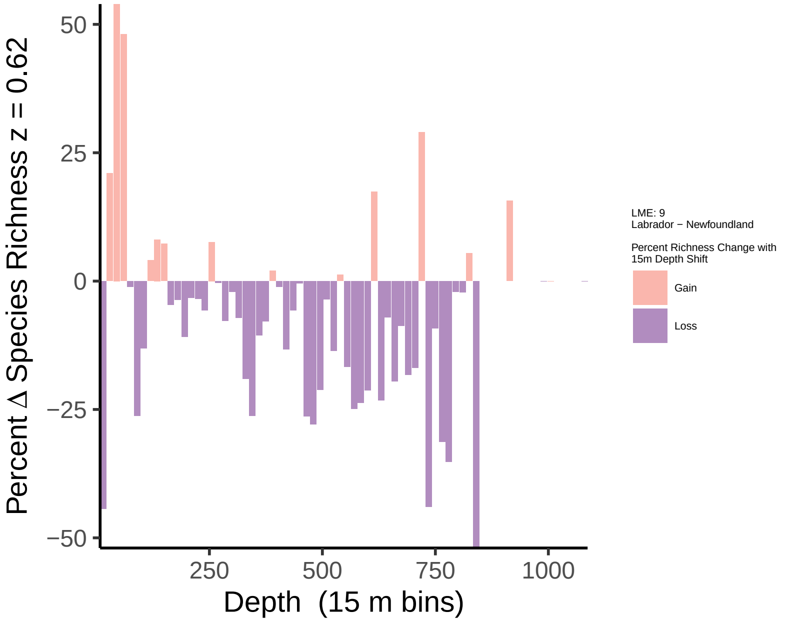

LME 12: Caribbean Sea

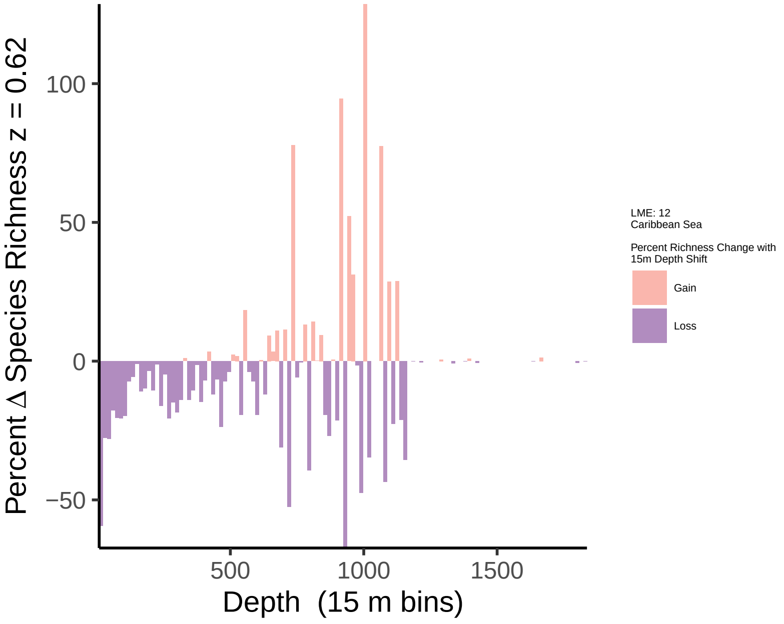

LME 13: Humboldt Current

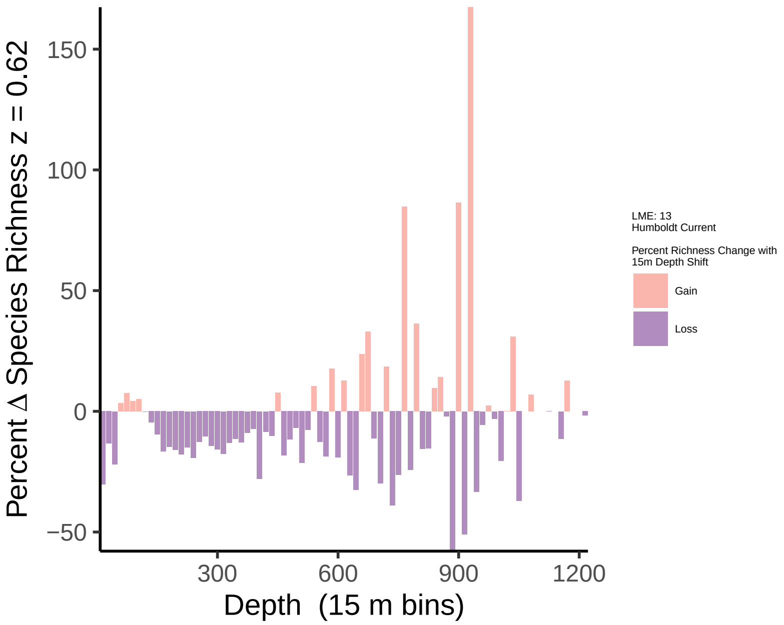

LME 16: East Brazil Shelf

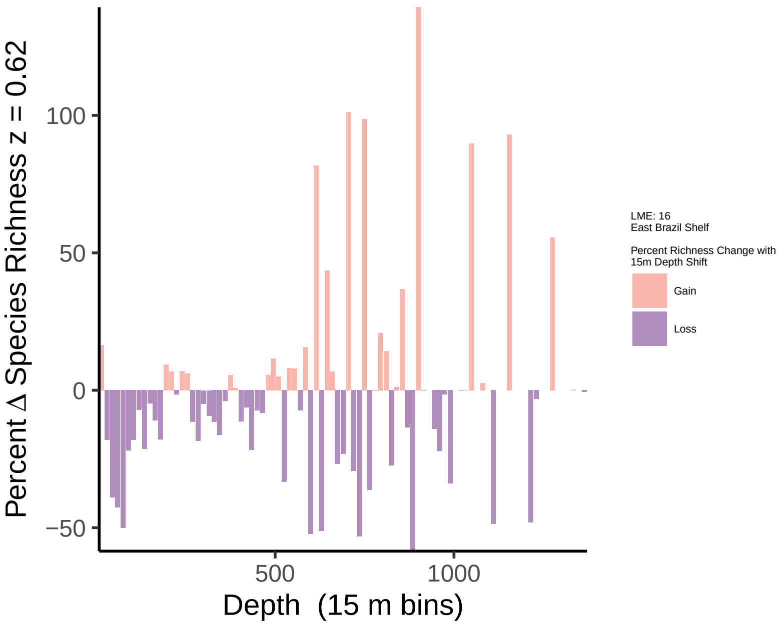

LME 14: Patagonian Shelf

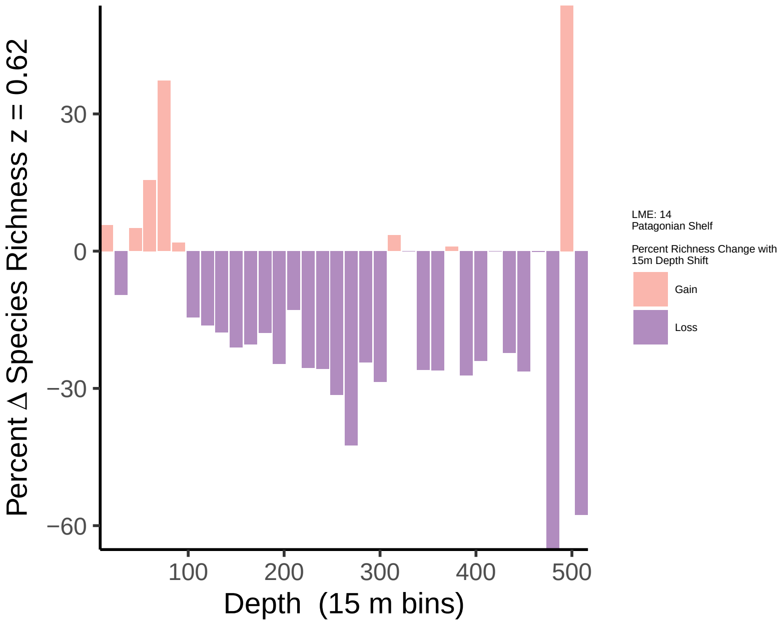

LME 17: North Brazil Shelf

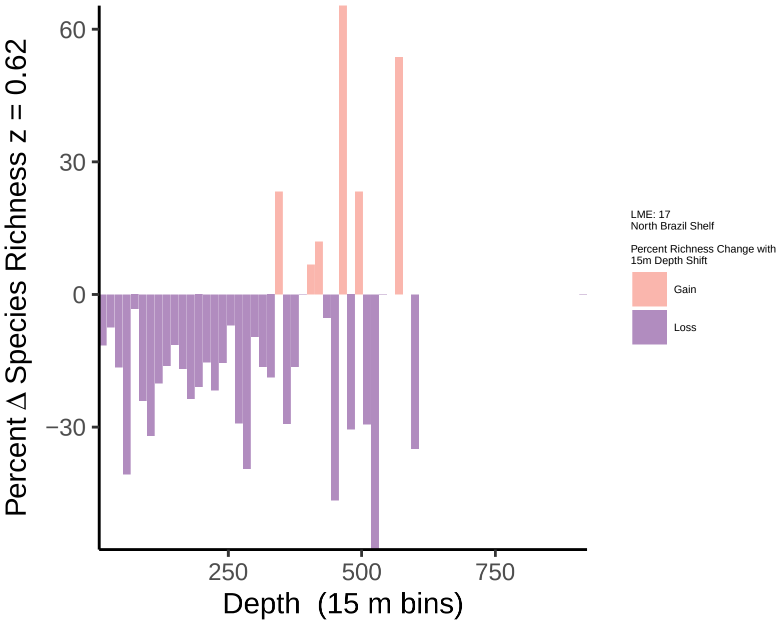

LME 15: South Brazil Shelf

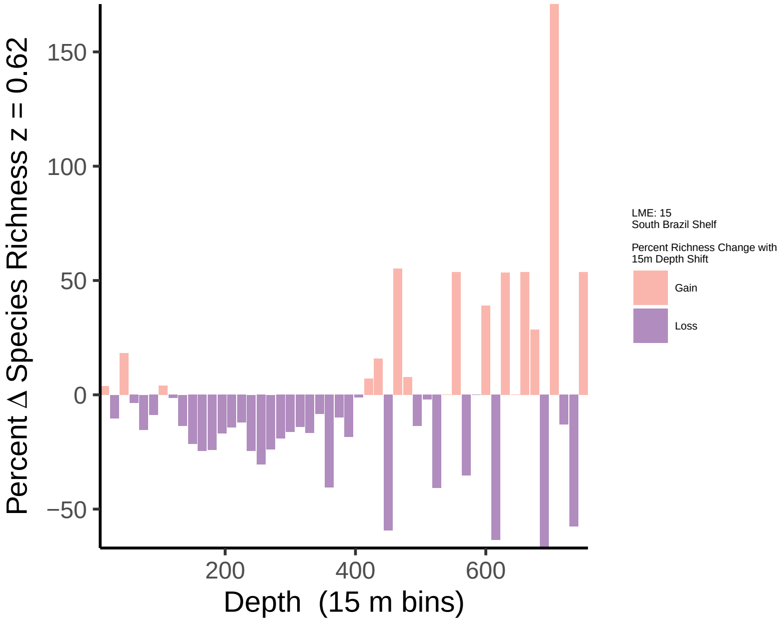

LME 18: Canadian Eastern Arctic − West Gree

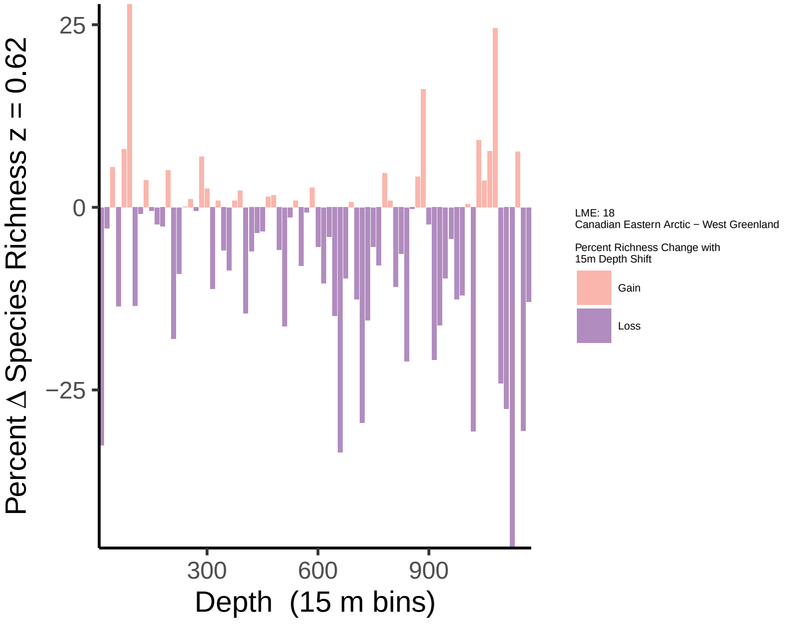

LME 19: Greenland Sea

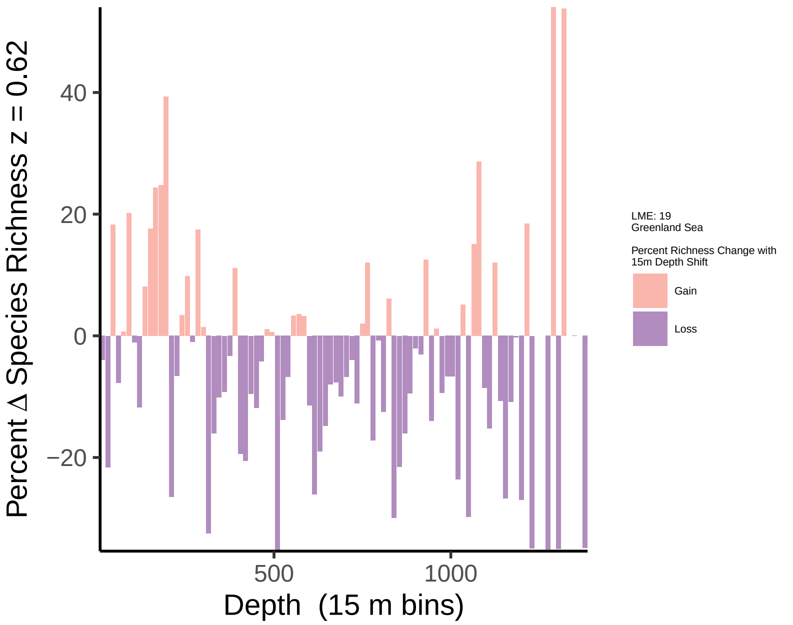

LME 22: North Sea

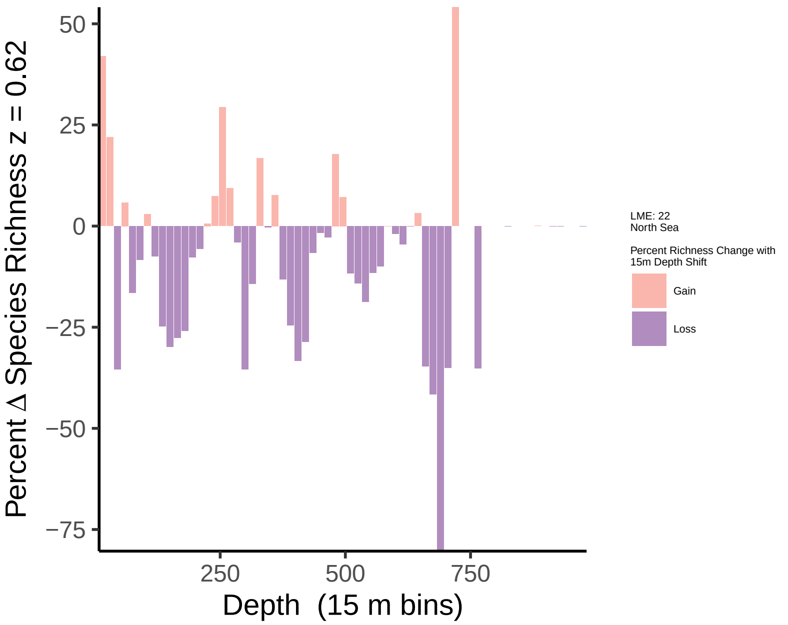

LME 20: Barents Sea

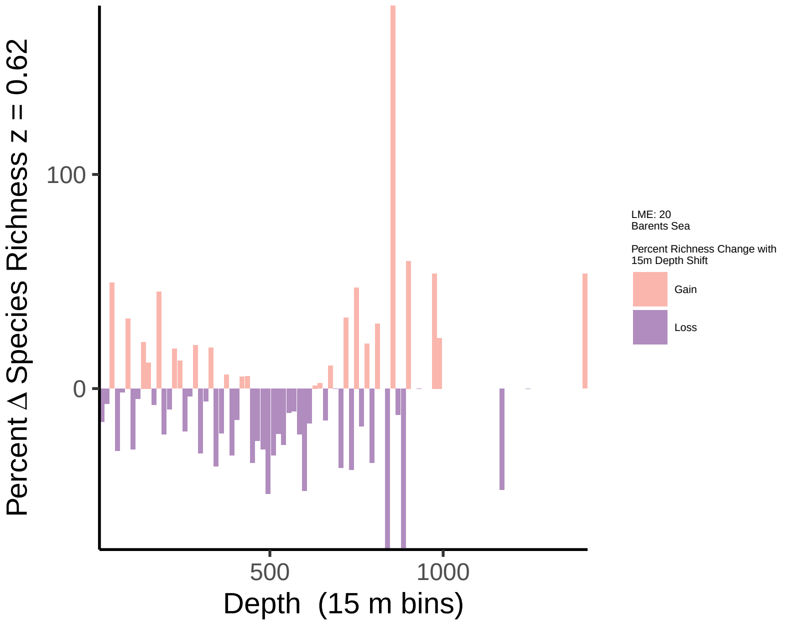

LME 23: Baltic Sea

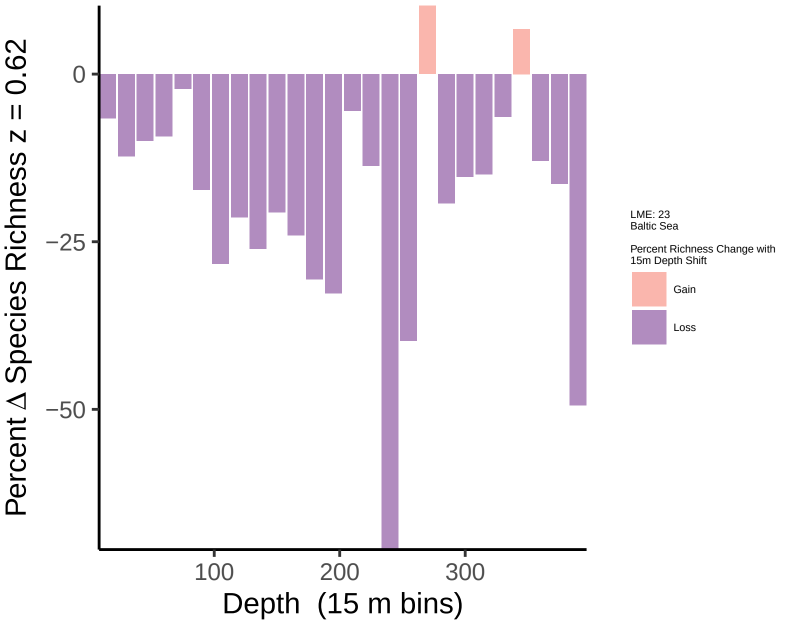

LME 21: Norwegian Sea

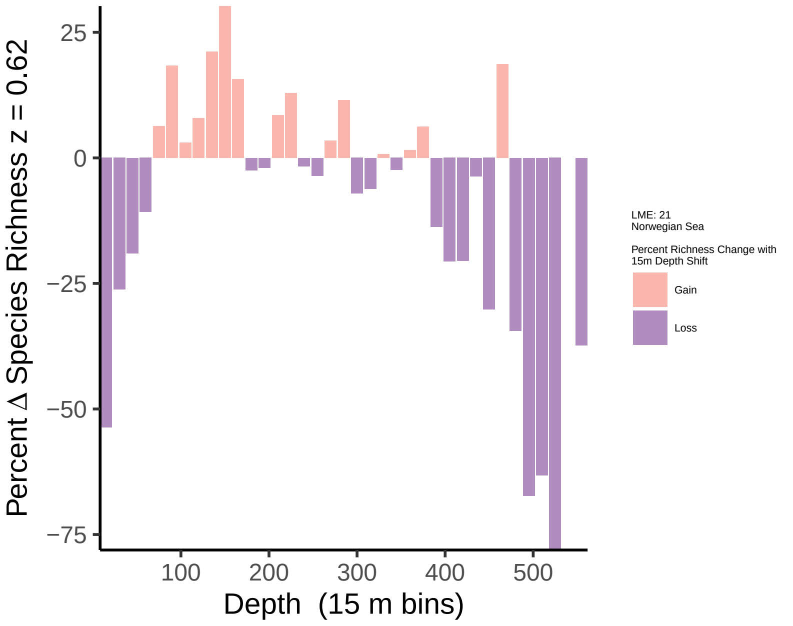

LME 24: Celtic−Biscay Shelf

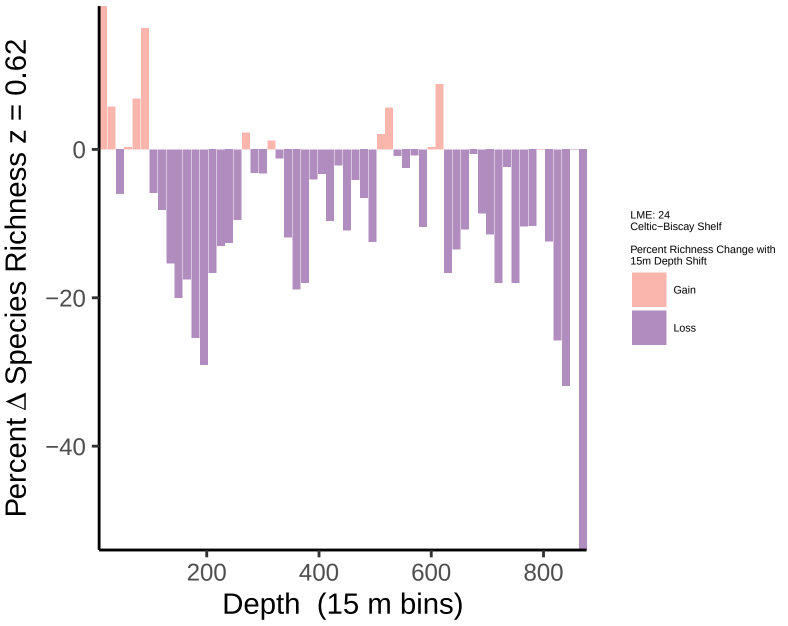

LME 25: Iberian Coastal

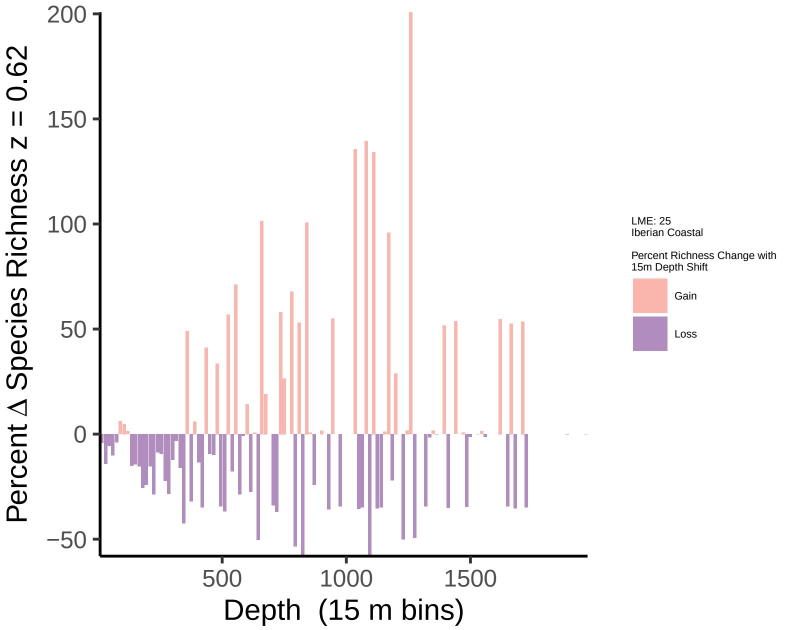

LME 28: Guinea Current

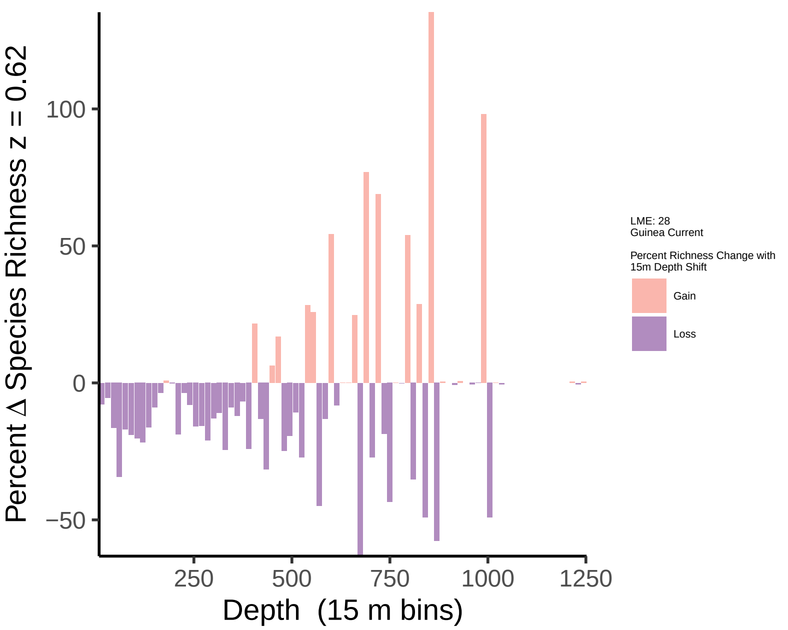

LME 26: Mediterranean Sea

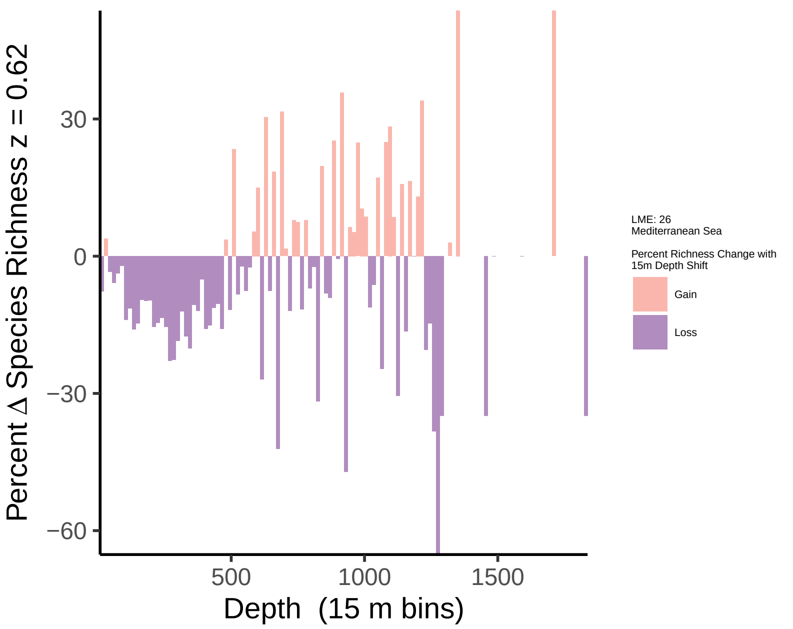

LME 29: Benguela Current

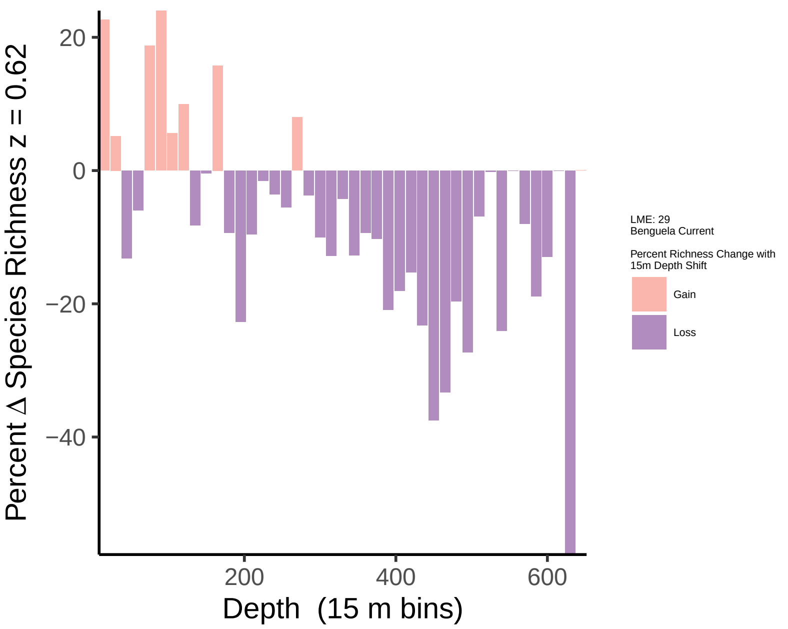

LME 27: Canary Current

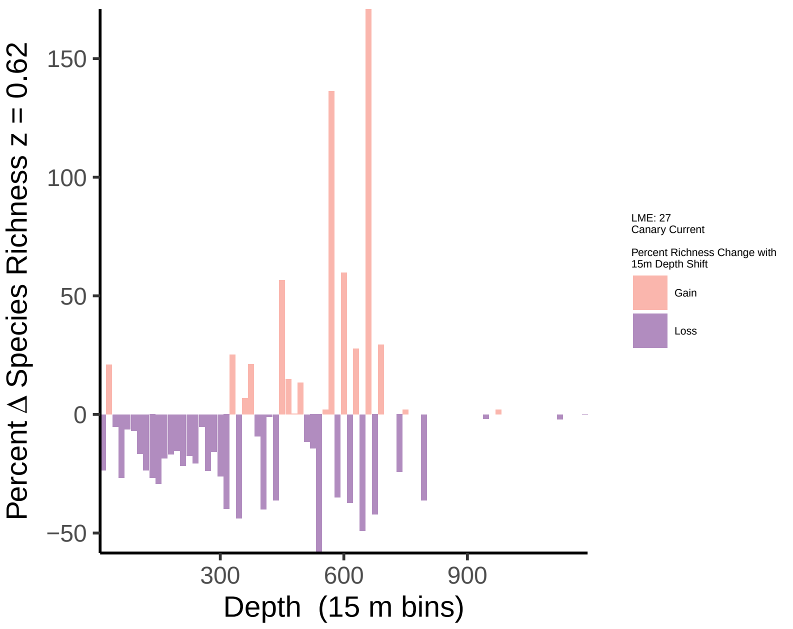

LME 30: Agulhas Current

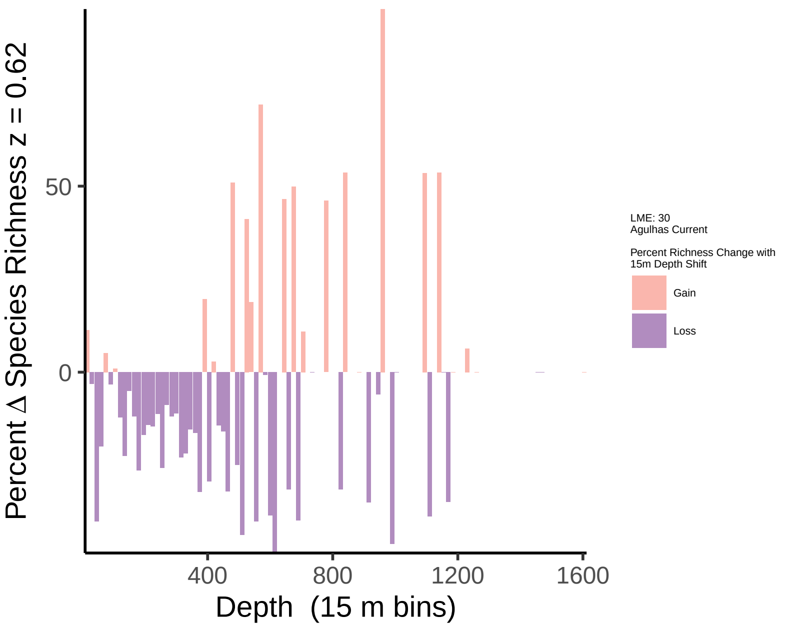

LME 31: Somali Coastal Current

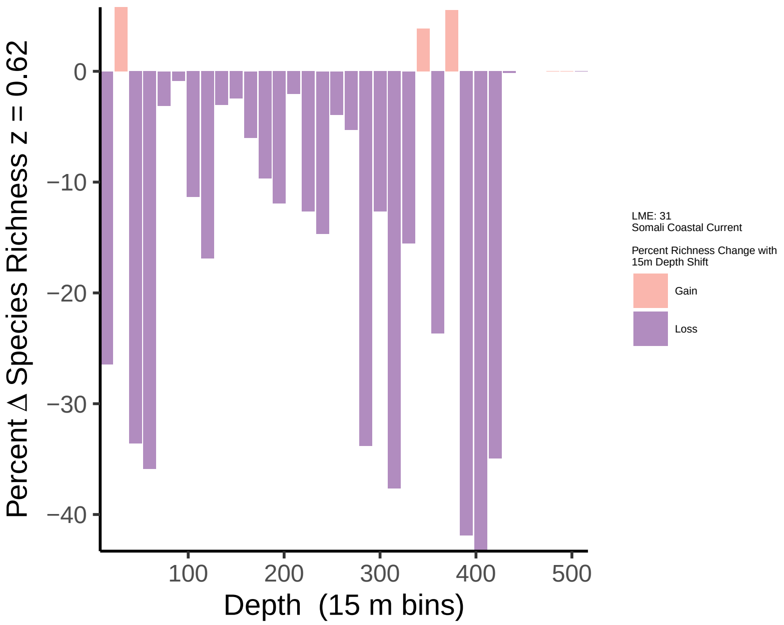

LME 34: Bay of Bengal

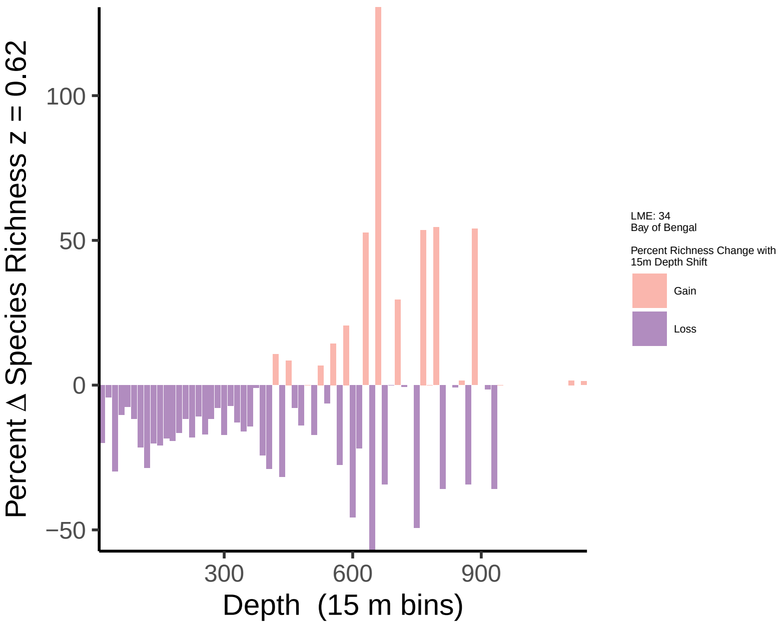

LME 32: Arabian Sea

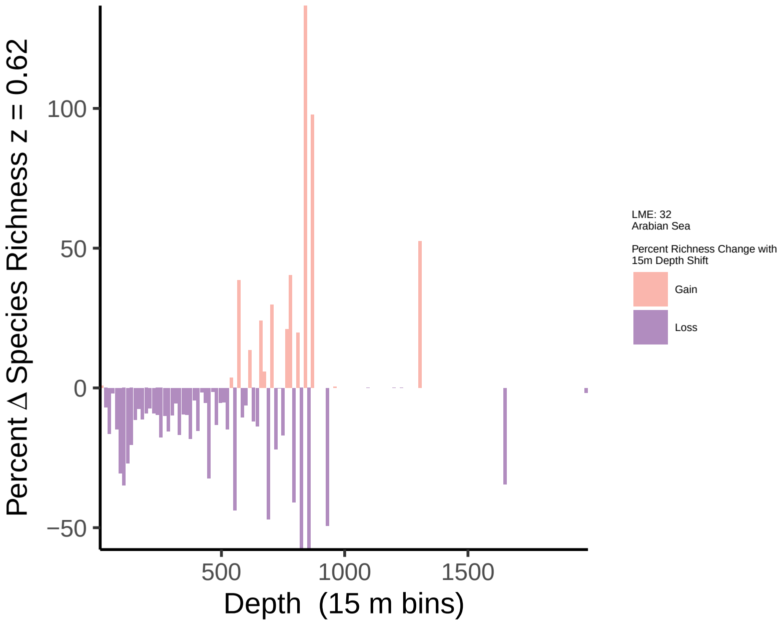

LME 35: Gulf of Thailand

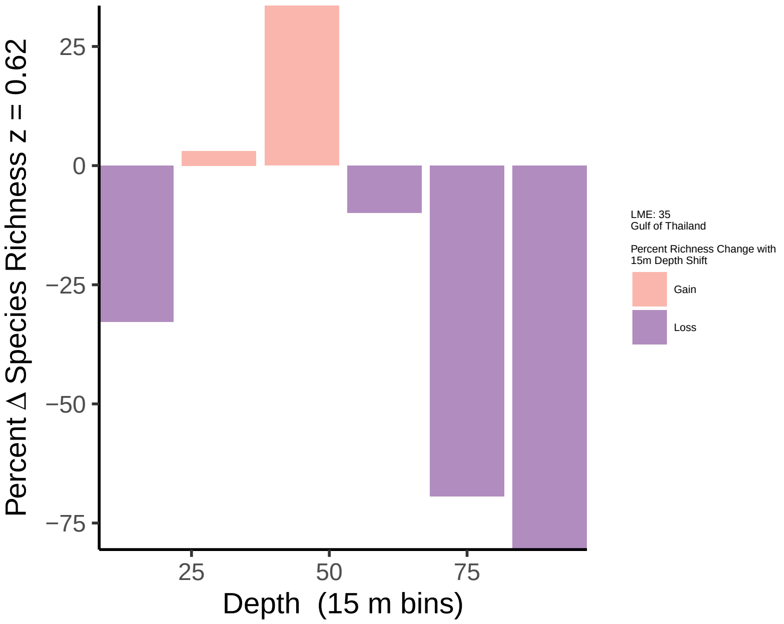

LME 33: Red Sea

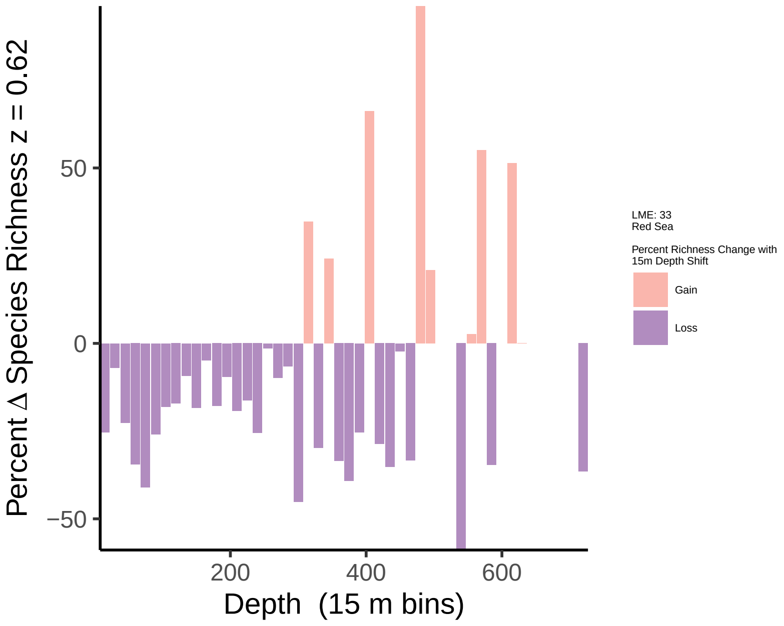

LME 36: South China Sea

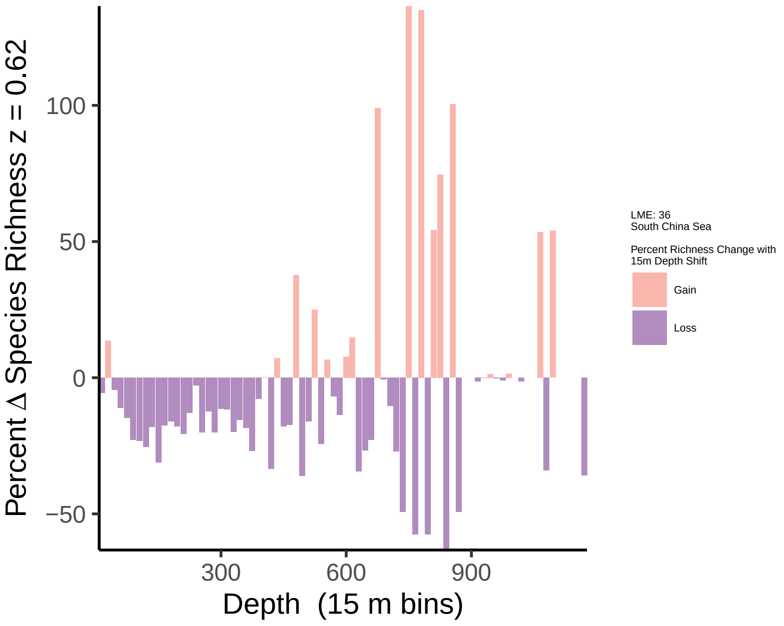

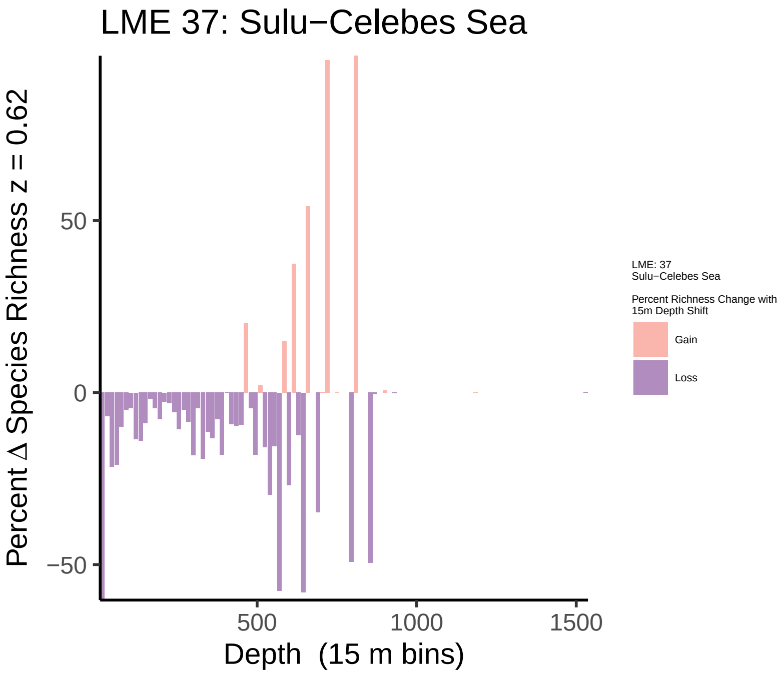
LME 37: Sulu−Celebes Sea
Percent Δ Species Richness z = 0.62
50
0
−50
500
1000
1500
Depth (15 m bins)
LME: 37
Sulu−Celebes Sea
Percent Richness Change with
15m Depth Shift
Gain
Loss

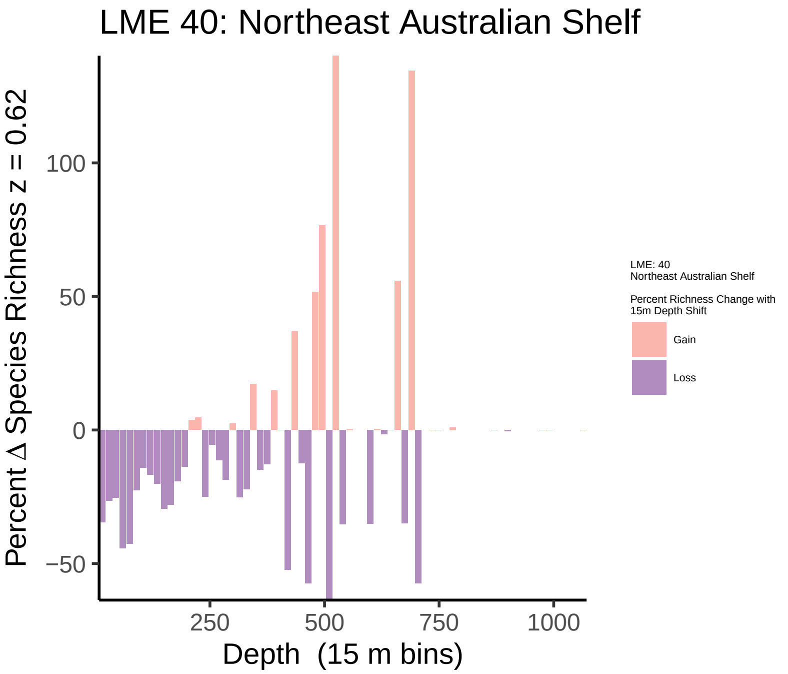
LME 40: Northeast Australian Shelf
Percent Δ Species Richness z = 0.62
100
50
0
−50
250
500
750
1000
Depth (15 m bins)
LME: 40
Northeast Australian Shelf
Percent Richness Change with
15m Depth Shift
Gain
Loss

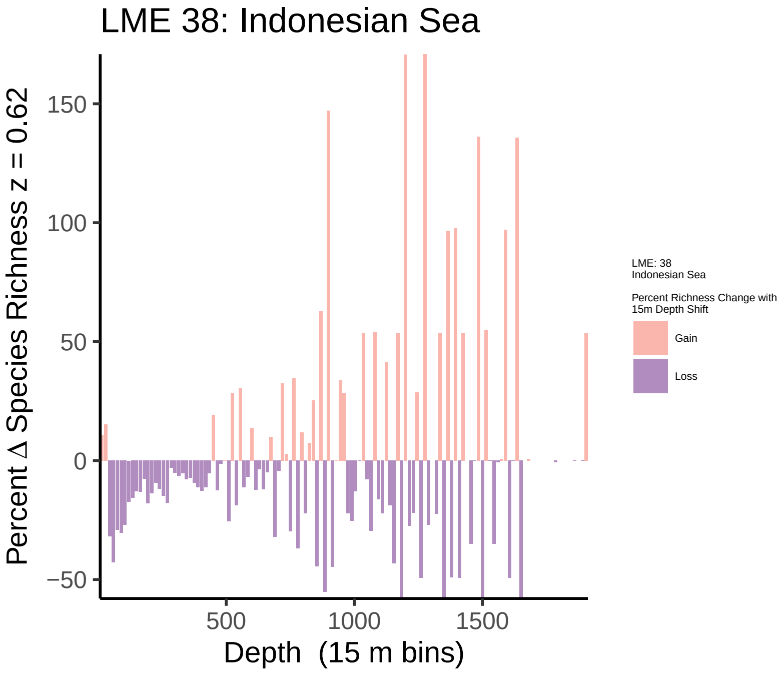
LME 38: Indonesian Sea
Percent Δ Species Richness z = 0.62
150
100
50
0
−50
500
1000
1500
Depth (15 m bins)
LME: 38
Indonesian Sea
Percent Richness Change with
15m Depth Shift
Gain
Loss

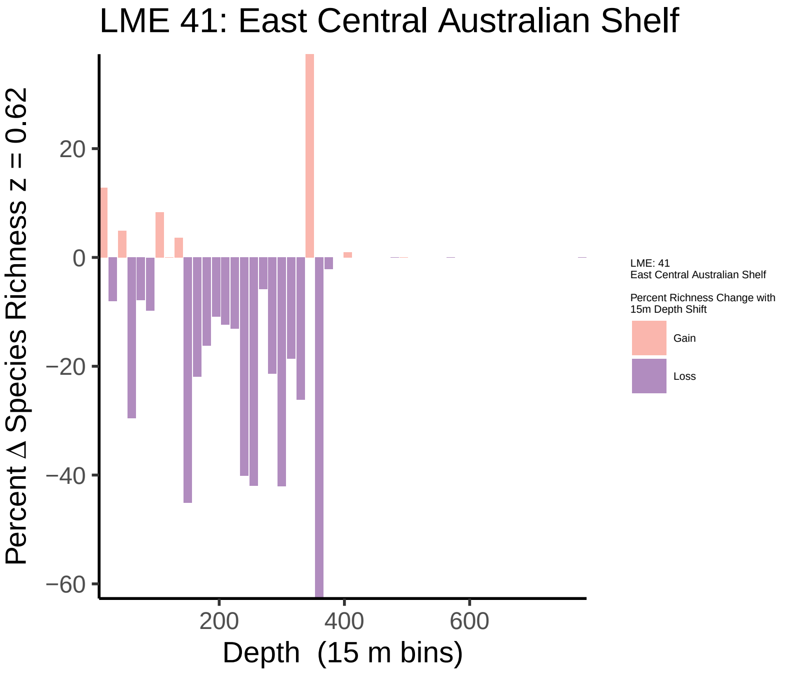
LME 41: East Central Australian Shelf
Percent Δ Species Richness z = 0.62
20
0
−20
−40
−60
200
400
600
Depth (15 m bins)
LME: 41
East Central Australian Shelf
Percent Richness Change with
15m Depth Shift
Gain
Loss

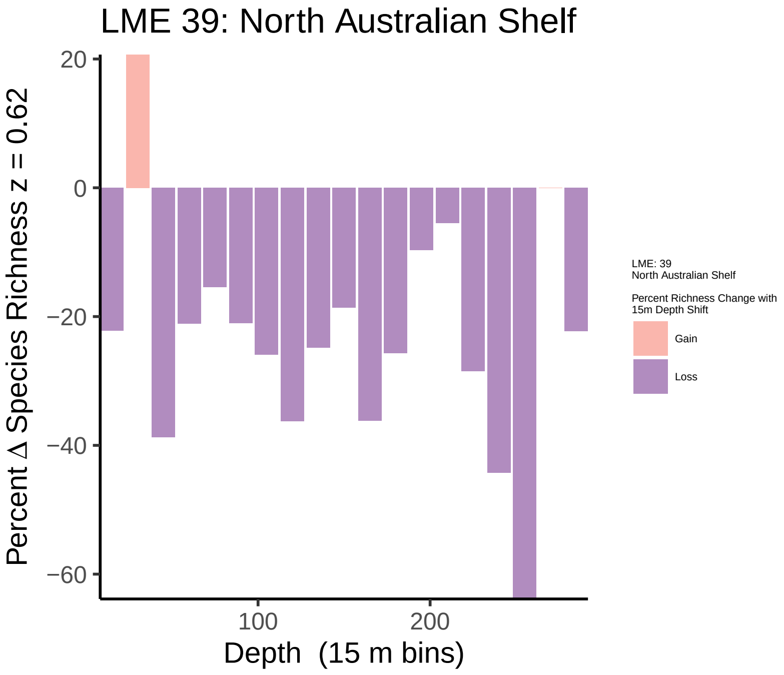
LME 39: North Australian Shelf
Percent Δ Species Richness z = 0.62
20
0
−20
−40
−60
100
200
Depth (15 m bins)
LME: 39
North Australian Shelf
Percent Richness Change with
15m Depth Shift
Gain
Loss

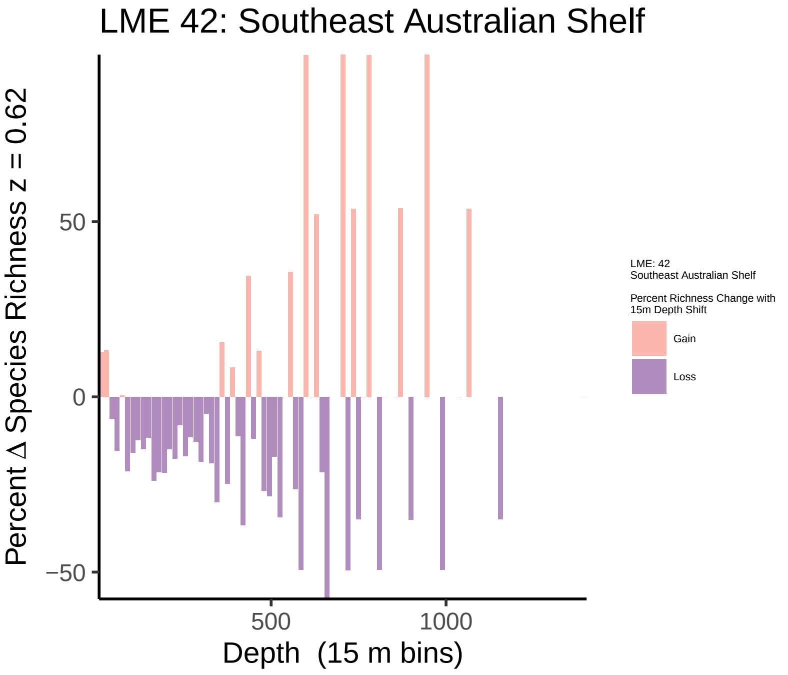
LME 42: Southeast Australian Shelf
Percent Δ Species Richness z = 0.62
50
0
−50
500
1000
Depth (15 m bins)
LME: 42
Southeast Australian Shelf
Percent Richness Change with
15m Depth Shift
Gain
Loss

LME 43: South West Australian Shelf

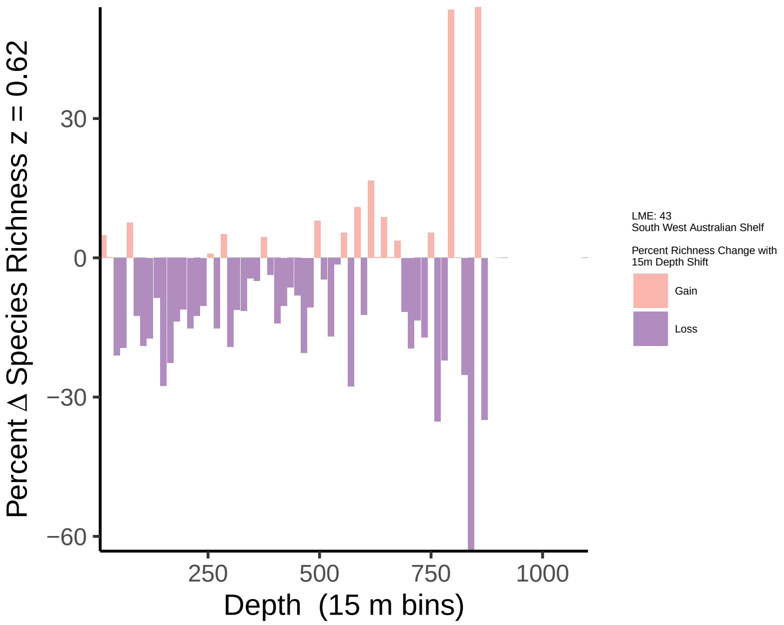

LME 46: New Zealand Shelf

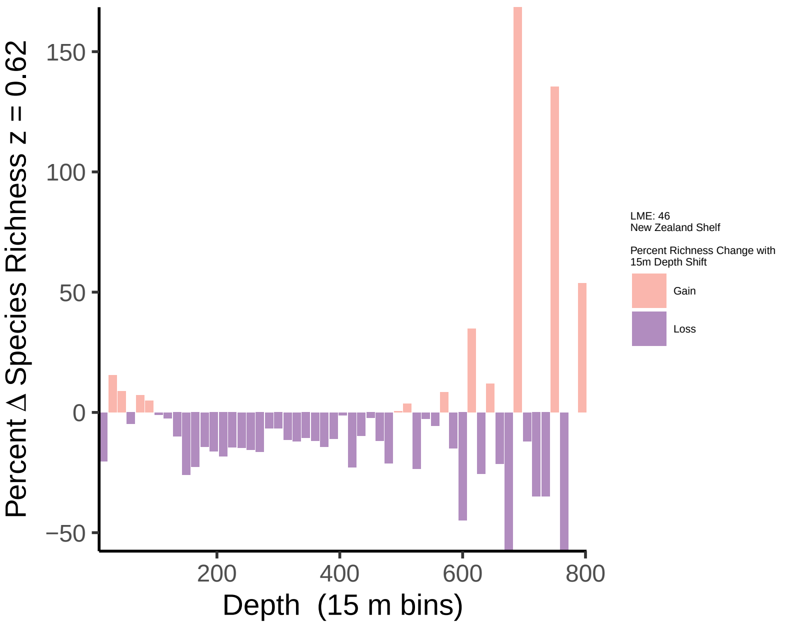

LME 44: West Central Australian Shelf

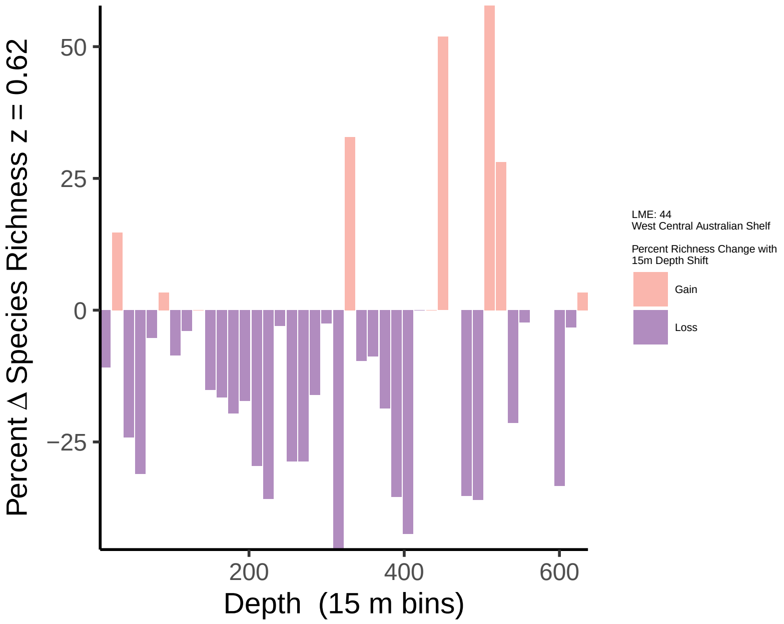

LME 47: East China Sea

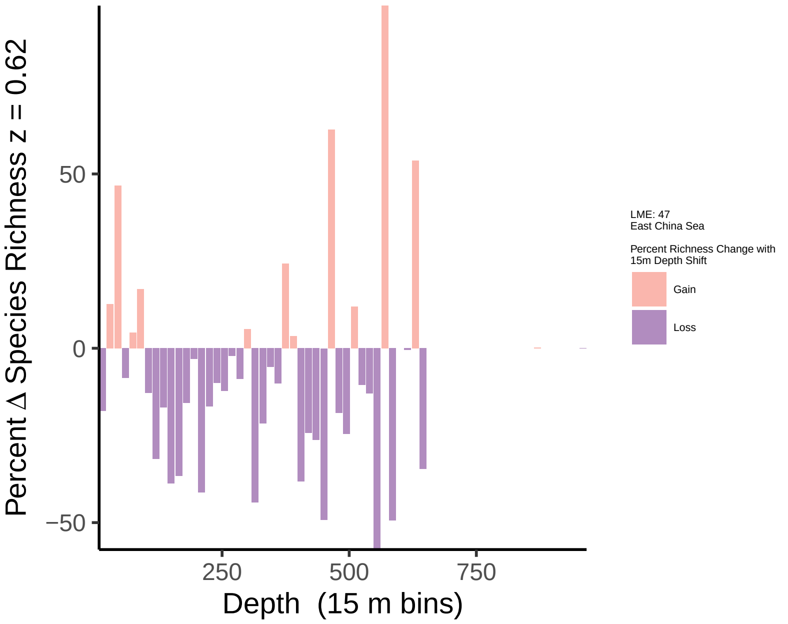

LME 45: Northwest Australian Shelf

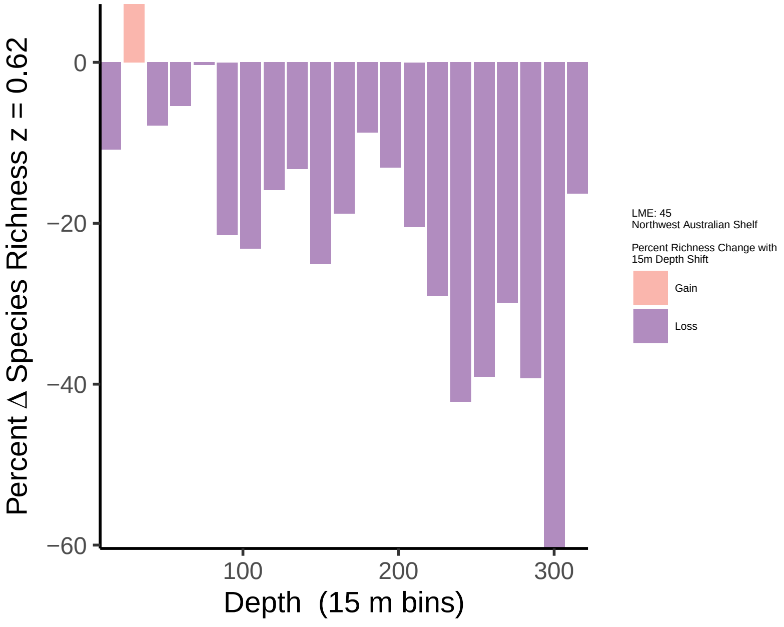

LME 48: Yellow Sea

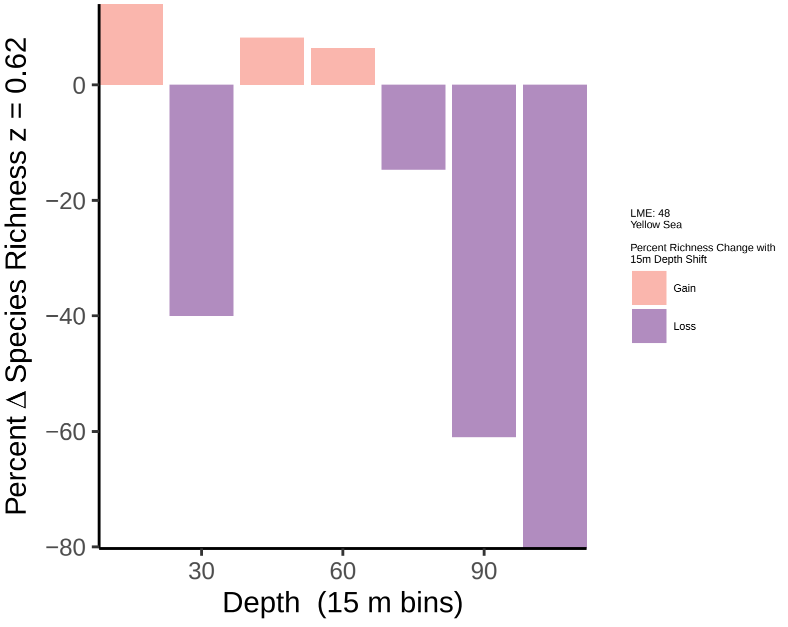

LME 49: Kuroshio Current

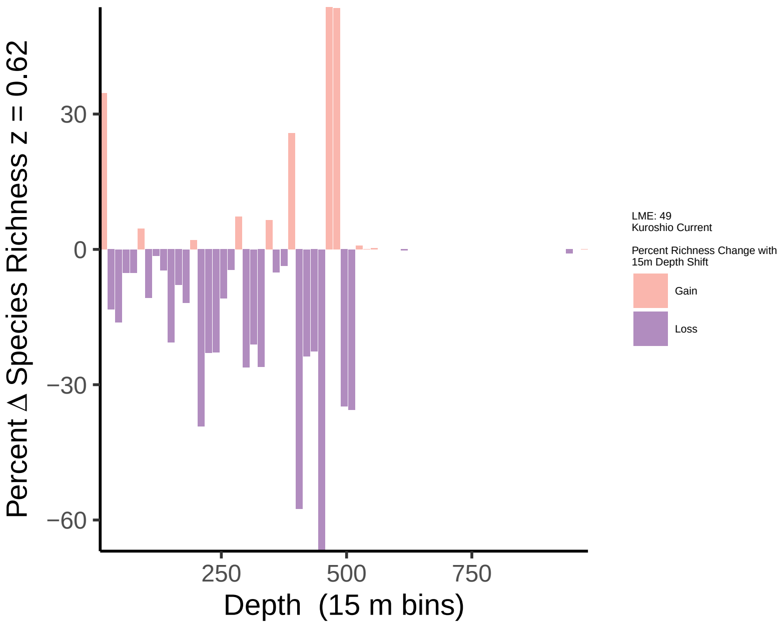

LME 52: Sea of Okhotsk

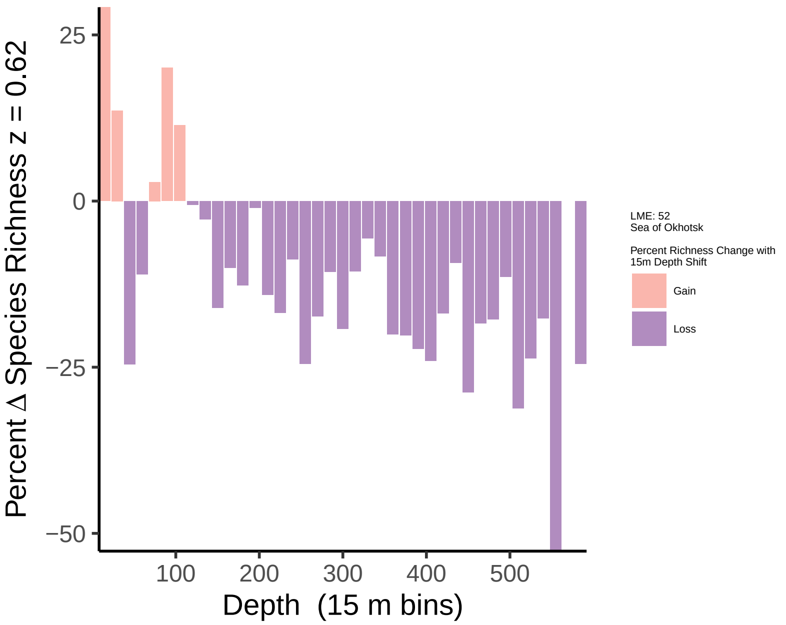

LME 50: Sea of Japan

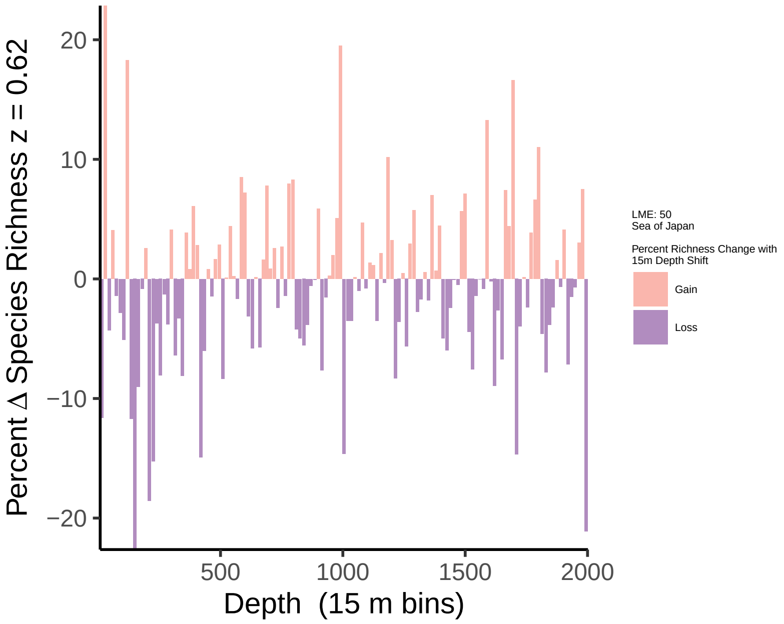

LME 53: West Bering Sea

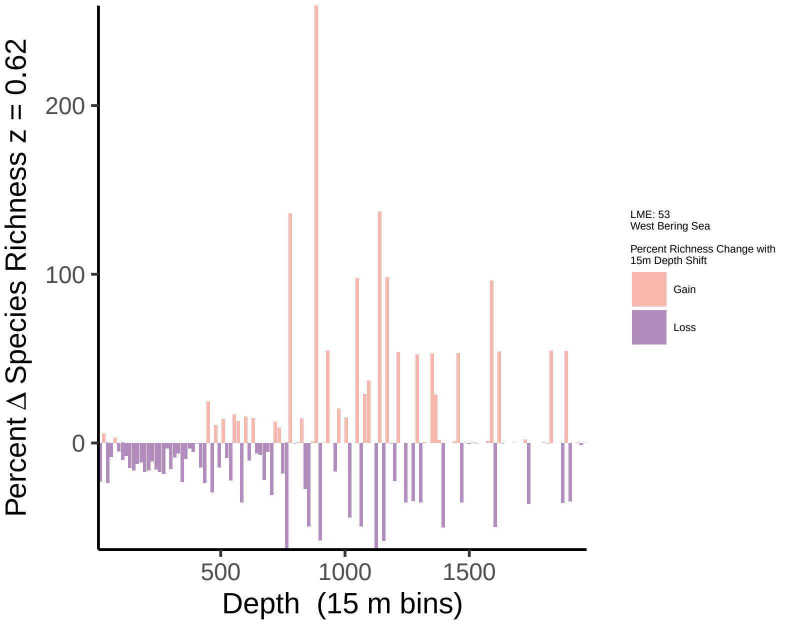

LME 51: Oyashio Current

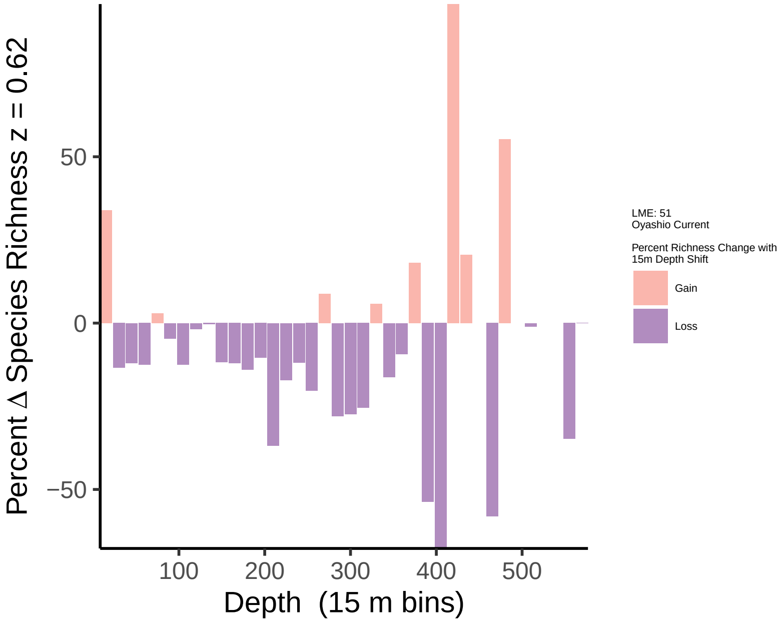

LME 54: Northern Bering − Chukchi Seas

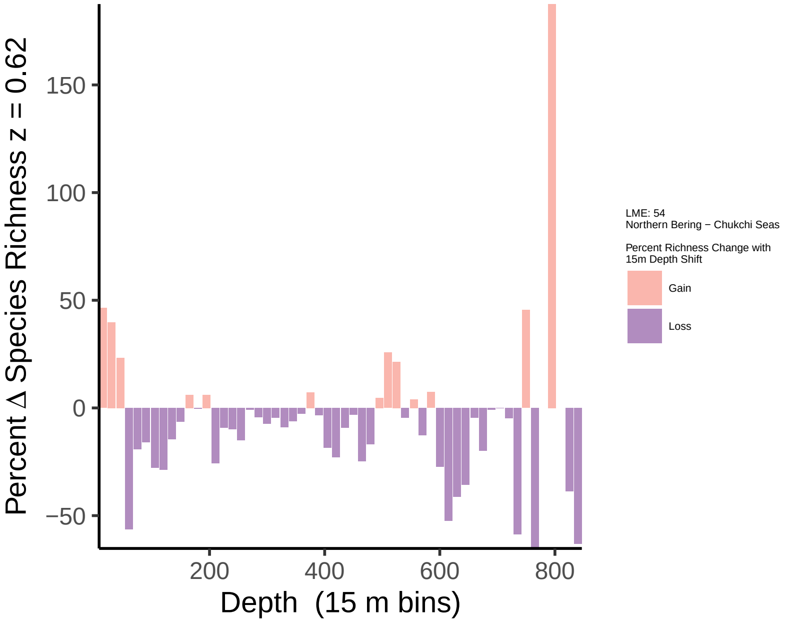

LME 55: Beaufort Sea

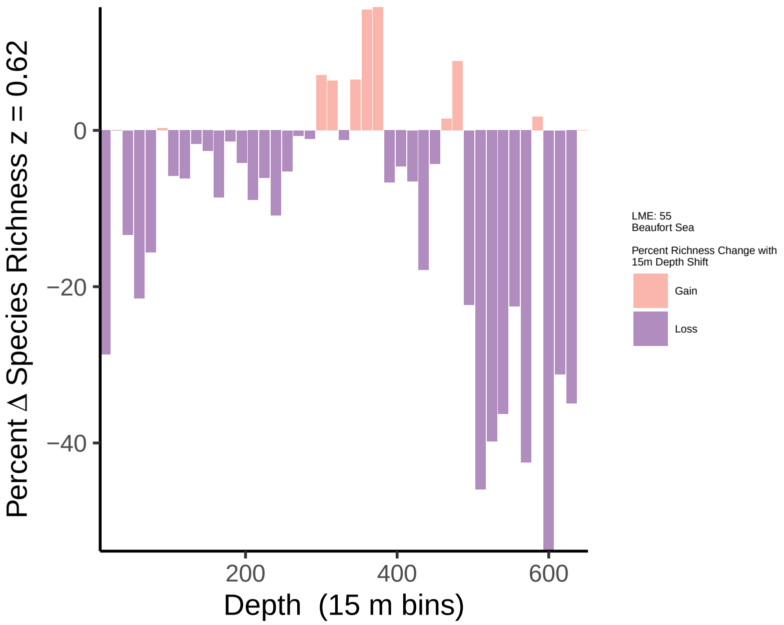

LME 58: Kara Sea

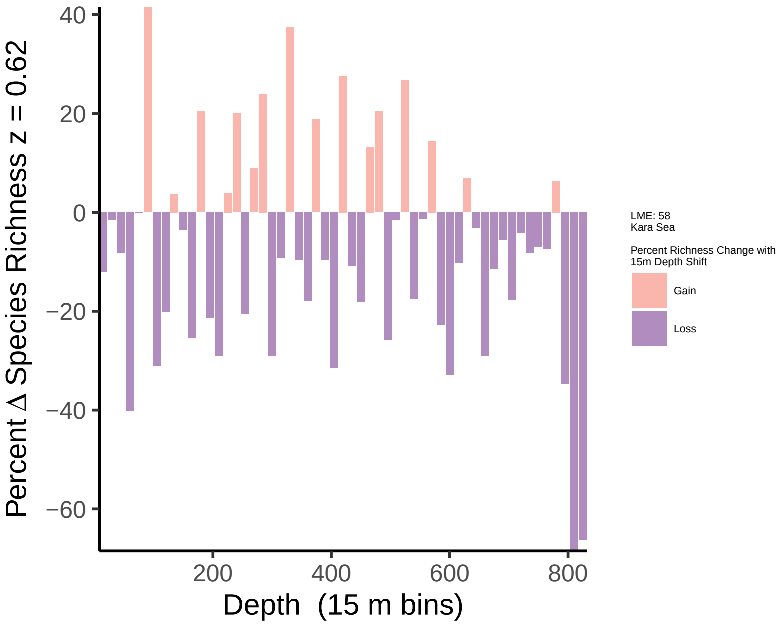

LME 56: East Siberian Sea

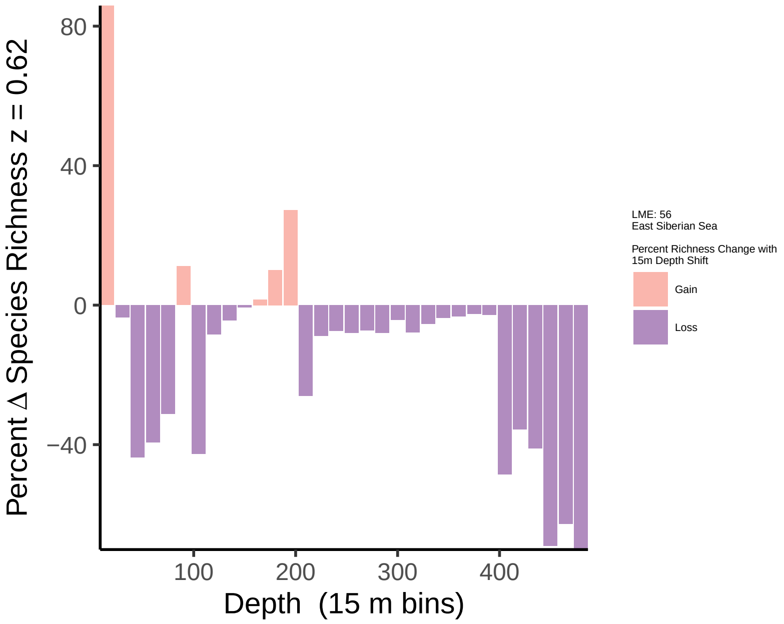

LME 59: Iceland Shelf and Sea

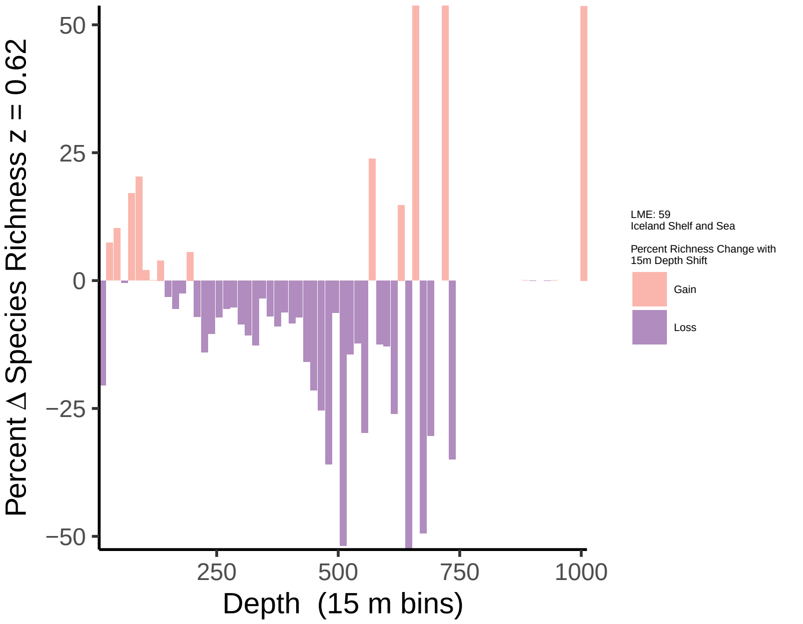

LME 57: Laptev Sea

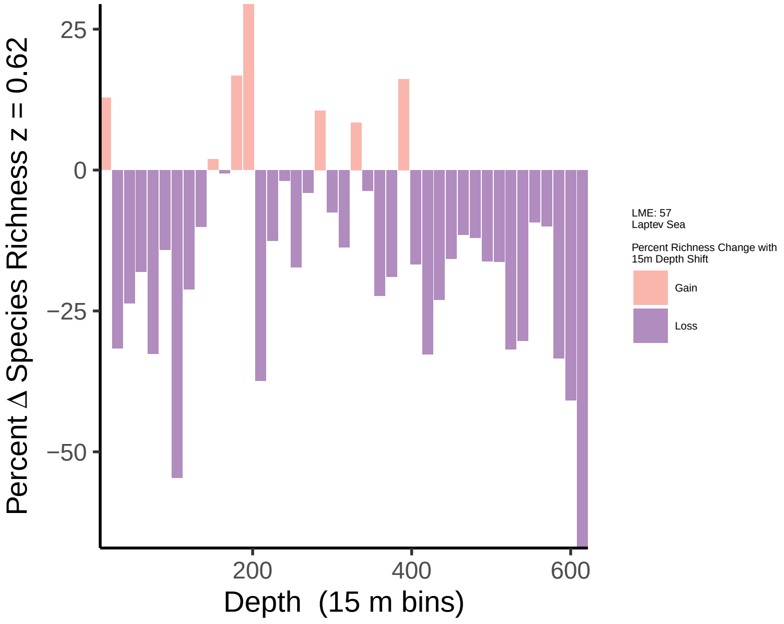

LME 60: Faroe Plateau

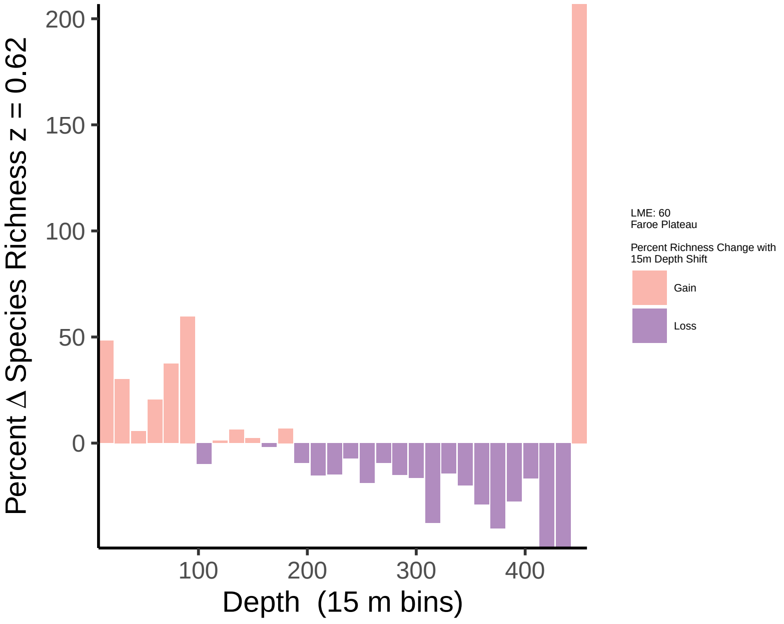

LME 62: Black Sea

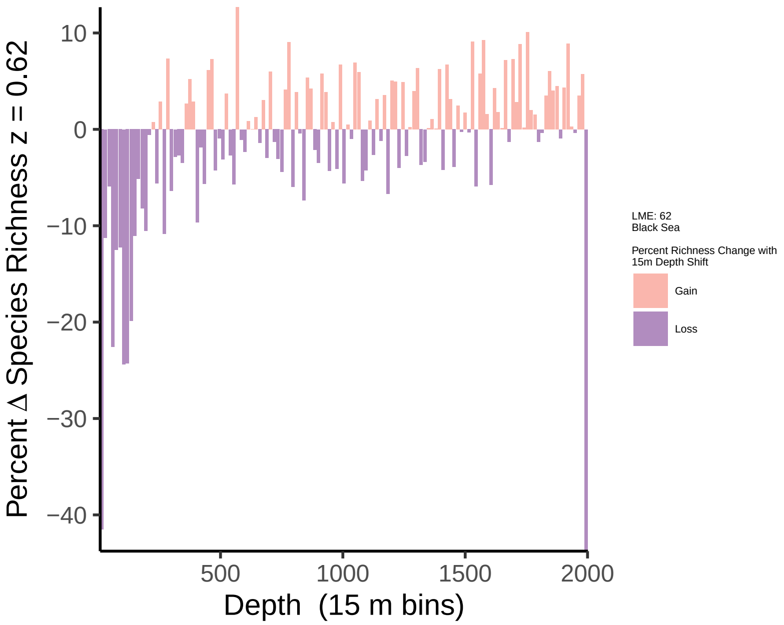

LME 66: Canadian High Arctic − North Greenla

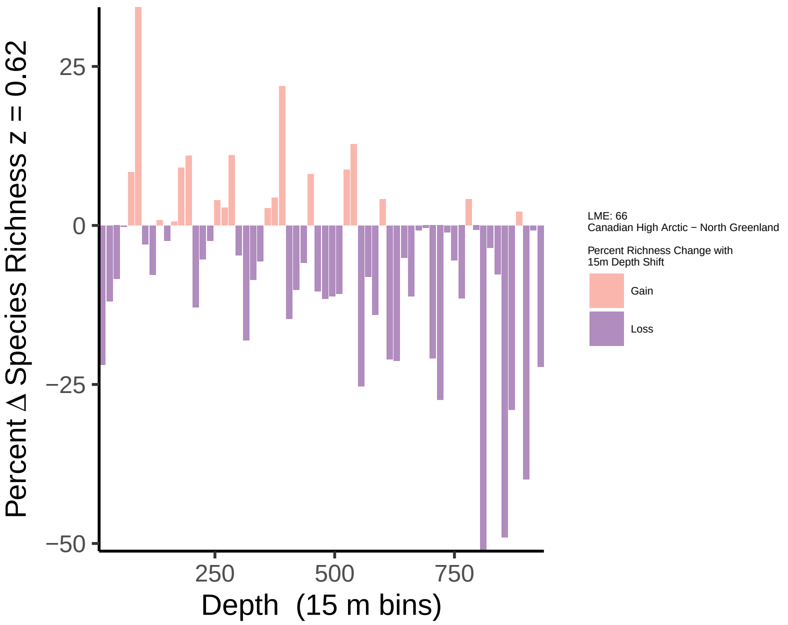

LME 63: Hudson Bay Complex

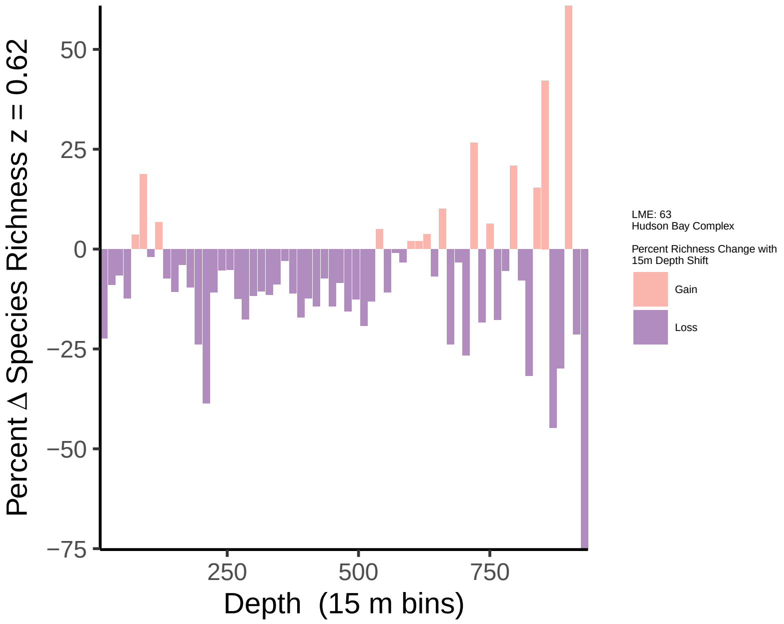

LME 65: Aleutian Islands

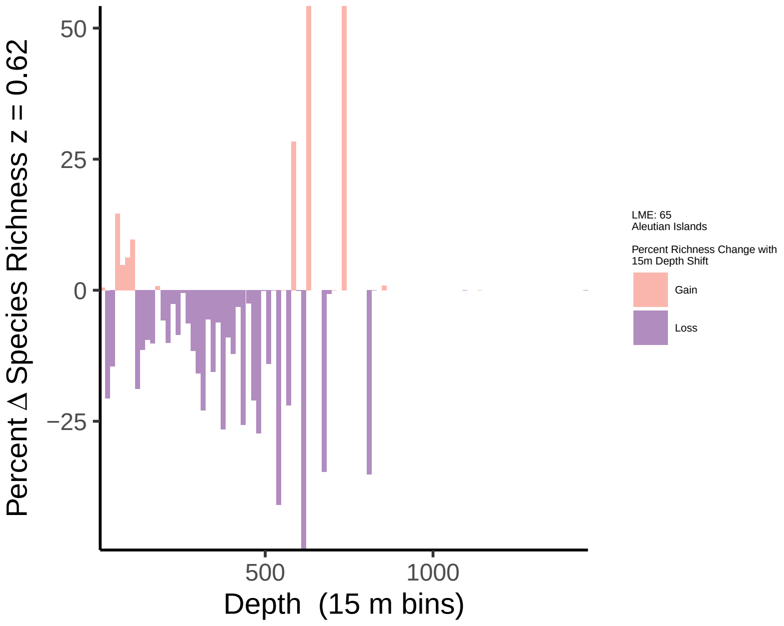

Supplement: Supplementary file 1 — Appendix S1 [file GCB-28-5185-s001.pdf]
